# Prognostic value of long non-coding RNA CCAT1 expression in patients with cancer: A meta-analysis

**DOI:** 10.1371/journal.pone.0179346

**Published:** 2017-06-08

**Authors:** Deyao Shi, Fashuai Wu, Feng Gao, Xiangcheng Qing, Zengwu Shao

**Affiliations:** Orthopaedic Hospital, Union Hospital, Tongji Medical College, Huazhong University of Science & Technology, Wuhan, P.R. China; University of South Alabama Mitchell Cancer Institute, UNITED STATES

## Abstract

**Background:**

LncRNA CCAT1 is significantly overexpressed in various types of cancers, suggesting that it might be associated with prognosis and clinicopathological features in patients with cancer.

**Methods:**

A comprehensive search was performed in Pubmed, Web of Science, OVID and CNKI databases. We also retrieved articles from other sources, such as retrieving from the reference lists of relevant articles. Eligible studies were included based on defined exclusion and inclusion criteria to perform a meta-analysis. STATA 14.0 was used to estimate pooled hazard ratios (HRs) with 95% confidence interval (95% CI), the heterogeneity among studies and publication bias to judge the prognostic value.

**Results:**

A total of 1587 patients from 11 eligible studies were included in the meta-analysis. The results showed that high expression level of CCAT1 was significantly associated with shorter overall survival in cancer patients (HR 2.335, 95% CI:1.551–3.517); in the subgroup analysis, region (China or UK), sample size (more or less than 100), type of cancer (digestive or non-digestive disease) and paper quality (score more or less than 7) did not alter the association between CCAT1 expression and cancer prognosis but preoperative treatment did. And CCAT1 expression was an independent prognostic marker for overall survival in patients with cancer (pooled HR 2.195, 95%CI:1.316–3.664) using Cox multivariate analyses. The clinicopathological parameters analysis further showed that increased expression level of CCAT1 was correlated with tumor size, lymph node metastasis, TNM stage, distant metastasis, microvascular invasion and capsular formation in relevant cancers.

**Conclusions:**

The meta-analysis results from present study suggested that increased expression level of CCAT1 was associated with poor prognosis and can serve as an independent biomarker. And the expression level of CCAT1 was associated with clinicopathological features in relevant cancers.

## Introduction

In 2012, an estimated 14.1 million new cancer cases and 8.2 million cancer deaths occurred worldwide[1]. Cancer is a leading cause of death worldwide in countries of all income levels[2]. The prognosis of most cancers is still poor. Early diagnosis and treatment is an important way to improve the prognosis of cancers. However, sensitivity and specificity of most of the cancer markers widely used now are not yet satisfactory[3]. Thus, identifying new molecular markers for early diagnosis and prognosis of cancers is highly needed.

Recent researches have shown us that the human genome encode a large number of long non-coding RNAs (lncRNAs), which in the past had been dismissed as simply transcriptional ‘junk’[4]. LncRNAs are a class of RNA molecules greater than 200 nucleotides in length with little or no protein coding capacity[5]. LncRNAs, which represent a new frontier in molecular biology, play important roles in regulating gene expression at epigenetic, transcriptional and post-transcriptional levels[6]. Diverse biological functions and pathological processes, including reprogramming of pluripotent stem cells, oncogenic progression and cell cycle regulation have been attributed to lncRNAs[7]. Studies have shown dysregulation of lncRNAs contribute to cancer progression through abnormal regulation of cancer-related cellular processes, such as proliferation, invasion, metastasis, apoptosis and multi-drug resistance[8–10], and lncRNAs have been implicated as promising markers for predicting the prognosis of cancers [11].

Colon cancer–associated transcript-1 (CCAT1,CARLo-5) is a lncRNA with 2628 base pairs in length and CCAT1 gene is located on chromosome 8q24.21 and in the vicinity of c-MYC, a well-known transcription factor[12].CCAT1 is aberrantly expressed in a variety of human cancers, including colon cancer, non-small cell lung cancer, hepatocellular carcinoma and esophageal squamous cell carcinoma etc., and has been shown to promote tumor cell proliferation, invasion and metastasis through various mechanisms[8, 9, 13–15]. Many studies have shown that high levels of CCAT1 expression may be associated with prognosis of human cancers[8, 9, 13–21]. However, most studies reported so far are limited in discrete outcome and sample size. Therefore, we conducted a quantitative meta-analysis to clarify the prognostic value of lncRNA CCAT1 expression in patients with cancer.

## Materials and methods

### Literature collection

The present review was performed in accordance with the standard guidelines for meta-analyses and systematic reviews of tumor marker prognostic studies[22, 23]. Two authors (DY Shi and FS Wu) independently used the following tools: Pubmed, Web of Science, OVID, and CNKI to obtain relevant articles on CCAT1 as a prognostic factor for the survival of patients with any cancer. The literature search language was limited to English in Pubmed, Web of Science and OVID and to Chinese in CNKI. The last search date was February 27, 2017. To increase the sensitivity of the search, both MeSH terms and free words were used. The search strategy was: “CCAT1 or colon cancer associated transcript 1 or CARLo-5” and “long non-coding RNA or lncRNA or non-coding RNA or RNA long non-coding” and “cancer or sarcoma or carcinoma or neoplasm or malignancy” and “prognosis or mortality of metastasis or progression or development or outcome or survival or recurrence or clinical significance”. The search strategy was correspondingly translated into Chinese in CNKI. We also retrieved articles from other sources, such as retrieving from the reference lists of relevant articles. A full electronic search strategy and procedure of our study for Pubmed was shown in S1 File.

### Study selection

The same two researchers independently assessed all the included studies and extracted the data. Studies were considered eligible if they met the following inclusion criteria: 1) Any type of human cancer was involved. 2) All tumors were confirmed through pathological or histological examinations. 3) CCAT1 expression was measured in human tissue or plasma. 4) The relationship between CCAT1 expression and survival was examined. 5) The patients had to be divided into two groups according to the expression level of CCAT1. 6) The survival curve or sufficient relevant data were provided to obtain hazard ratios (HR) for survival rates and their 95% confidence intervals.

Studies were excluded if they met the following criteria: 1) They were letters, case reports, reviews, conference reports or expert opinions.2) Neither English or Chinese language articles. 3) The required data could not be calculated or received from the original article or the authors. 4) Animal studies, cellular level studies or molecular level studies of CCAT1. 5) The article was not found in full or had been published repeatedly. We included the latest and the most informative article when overlapping studies were retrieved. Controversies were resolved through discussion with a third researcher (F Gao).

### Data extraction

Data extraction was repeated independently by the two researchers (DY Shi, FS Wu), and in the situation of a disagreement, a consensus was reached by a third researcher (F Gao). For each study, the following characteristics of the individual research articles were collected: author; journal name; year of publication; country of the population enrolled; ethnicity; sample size; study design; follow-up data; overall survival (OS); disease-free survival (DFS); progression-free survival (PFS); recurrence-free survival (RFS); survival analysis methodology; CCAT1 expression level; cut-off values; treatment information; HR values and their 95% confidence intervals; and patient data such as tumor size, TNM stages, lymph node metastasis and distant metastasis. We extracted HRs according to the following three methods. In method 1, we obtained the reported HRs and their 95% confidence intervals directly from the publication. However, there were still some HRs that could not be obtained directly through the above method. In method 2, by using the Engauge Digitizer version 9.8 we obtained necessary data from Kaplan-Meier Curves, then we inputted the extracted survival rates at specified times into the spreadsheet set up by Tierney JF et al[24] to calculate HRs and their 95% confidence intervals. If possible, we asked for original data directly from the authors of the relevant studies.

### Quality assessment of primary studies

For quality control of the paper, the assessment was performed by three authors (DY Shi, FS Wu, F Gao) according to the Newcastle-Ottawa Scale (NOS) oriented to cohort studies. NOS contains 3 categories including selection (4 items), comparability (2 items) and outcome (3 items). If a study met the following criteria, it can be awarded 1 score for each numbered item. Criteria were as below: In selection category, Item 1: exposed cohort truly represent the cancer patients with high expression level of CCAT1 in the community or somewhat represent those cancer patients in the community. Item 2: the non-exposed cohort, namely the cancer patients with low expression level of CCAT1, were drawn from the same community as the exposed cohort. Item 3: relative expression level of CCAT1 of patients with cancer was precise and exact. Item 4: demonstration that outcome of interest was not present at start of study. In comparability category, Item 1: Study controls for the most important factor: all patients had negative histories of exposure to either chemotherapy or radiotherapy before surgery. Item 2: study controls for any additional factor (This criterion could be modified to indicate specific control for a second important factor). In outcome category, Item 1: Assessment of outcome was independent blind assessment or reliable record. Item 2: Follow-up was long enough for outcomes to occur (> = 30 months). Item 3: Adequacy of follow up of cohorts, complete follow up or subjects lost to follow up unlikely to introduce bias, loss of follow-up was smaller than 20% or description provided of those lost. Disagreements were resolved through discussion with another researcher (XC Qing). The total scores ranges from 0 to 9. The study is considered high quality, if its score is greater than or equal to 7. A detailed table for quality assessment of included studies was shown in S1 Table.

### Statistical analysis

1) Pooled hazard ratios (HRs) and their associated 95% confidence intervals (CI) were merged using a fixed-effect model (Mantel-Haenszel). A random-effect model was applied if heterogeneity was observed. An HR>1 indicates that the patients with high CCAT1 expression have a poor prognosis and the patients with low CCAT1 expression have a good prognosis. And to explore the source of heterogeneity, subgroup analysis and meta regression by factors contributing to heterogeneity were performed. We also performed sensitivity analyses to examine the effect of each study on the overall pooled results. 2) For the studies from which we could obtain clinicopathological characteristics, we calculated the pooled odds ratios (ORs) or odds ratios values and performed heterogeneity tests to analyze the relationship between high CCAT1 expression and tumor size, TNM stages, lymph node metastasis and distant metastasis in different types of cancers. 3) The test for heterogeneity of combined HRs was carried out using a χ2 based Cochran Q test and Higgins I^2^ statistic. A p value of <0.05 or an I^2^ value of >50% was considered statistically significant. The presence of publication bias was evaluated by using funnel plots, Begg’s test and Egger’s test. A p value of less than 0.05 was considered statistically significant[25]. Statistical analysis and graphical representation were performed using Stata software statistical software version 14.0 (Stata Corporation, College Station, TX, USA).

## Results

### Included studies and characteristics

**Fig 1:**
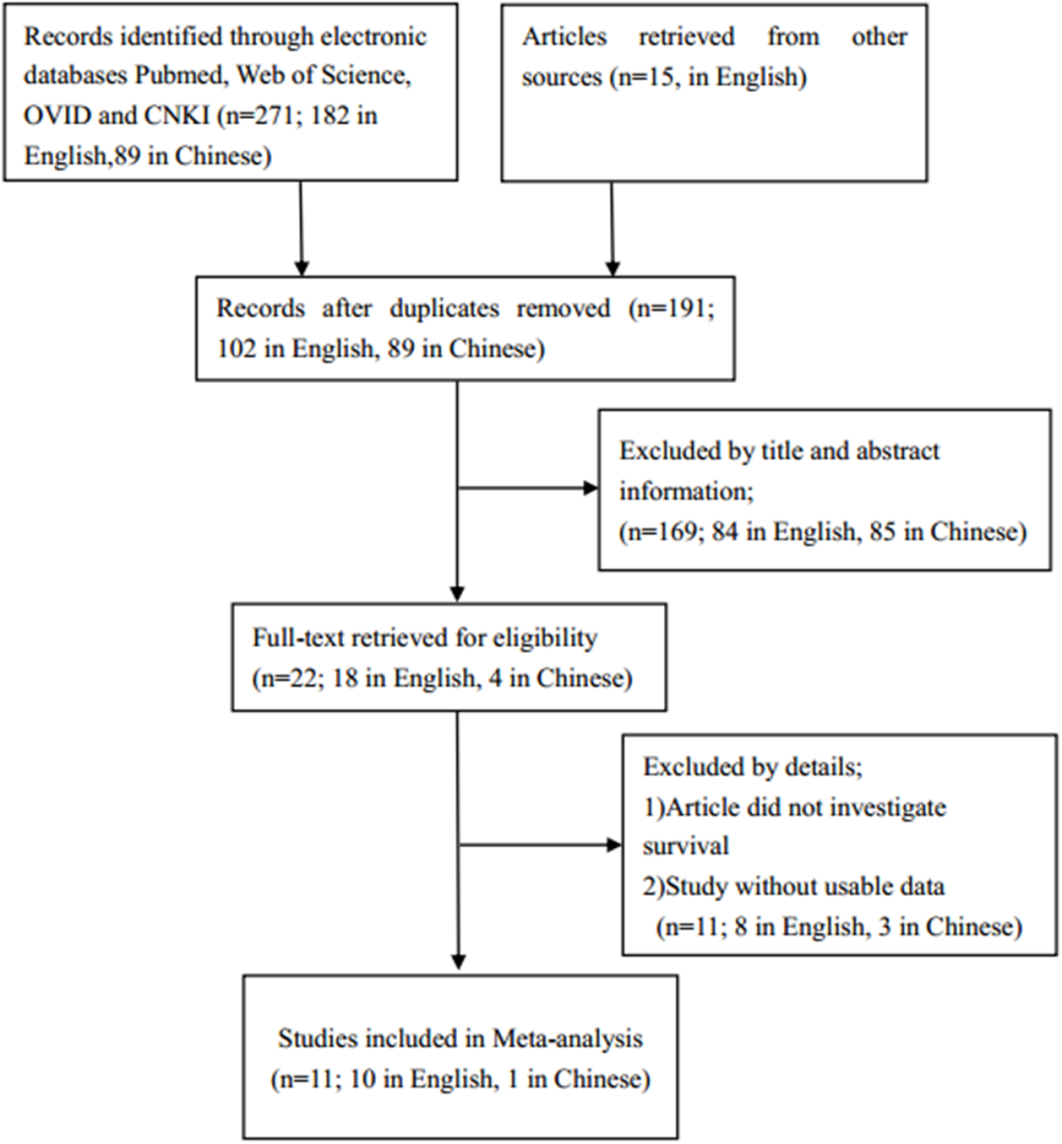
The flow diagram of the meta-analysis.

Our search strategy identified 271 articles in electronic databases. We got another 15 articles from other sources. After removing duplicates, there were 191 articles left. Then we reviewed the titles and abstracts of articles, and 169 irrelevant articles were excluded. After a more careful full-text reading, 11 of these articles were excluded[26–36]. Eventually, 11 published articles, of which 1 is in Chinese and the others are in English, were included in the current meta-analysis[8, 9, 13–21]. The detailed screening process is shown in **Fig 1**.

Among these 11 studies, a total of 1587 patients were represented, with mean sample size of 144.0 (range 48 to 638). Three studies included more than 100 participants. The accrual period of these studies ranged from 2015 to 2017. The regions represented in the studies include the United Kingdom (1) and China (10). Seven different types of cancer were evaluated with the greatest number being digestive system malignancies (3 colorectal cancers, 3 hepatocellular carcinomas, 1 gastric cancer and 1 esophageal squamous cell carcinoma). Other types of cancer were also included (1 breast cancer, 1 non-small cell lung cancer and 1 endometrial carcinoma). Ten studies analyzed the expression level of CCAT1 by qRT-PCR, while the other one study utilized the method of in situ hybridization. OS, RFS, DFS and PFS were estimated as survival outcome measures in 100% (11/11), 36% (4/11), 9% (1/11) and 9%(1/11) of the studies, respectively. Cox multivariable analyses were performed in 54.5% (6/11) of studies. The association between CCAT1 and clinicopathological characteristics of cancers (TNM stage, lymph node metastasis, distant metastasis, tumor size, microvascular invasion etc.) was estimated in 91%(10/11) of the studies. The main characteristics of each study are shown in **Table 1**.

**Table 1:** Characteristics of studies included in the meta-analysis.

Author	year	region	sample type	tumor type	sample size	preoperative treatment	clinical stage of tumor	method of CCAT1 expression	Elevated CCAT1	cut-off value	outcome measure	survival analysis	Method^*^	NOS scores
Deng et al.	2015	China	Tissue	Hepatocellular carcinoma	66	N/A	N/A	qRT-PCR	significant higher(p<0.001)	median	RFS,OS	No	2	7
Zhu et al.	2015	China	Tissue	Hepatocellular Carcinoma	86	No	N/A	qRT-PCR	significant higher(p<0.05)	median	RFS,OS	Univariate, multivariate analysis	1,2	7
Zhang et al.	2016	China	Tissue	Esophageal squamous cell carcinoma	90	N/A	TNM I-IV	qRT-PCR	80.7%, significant higher(p<0.001)	median	OS	Univariate, multivariate analysis	1	7
He et al.	2014	China	Tissue	Colon cancer	48	No	TNM I-IV	qRT-PCR	significant higher(p<0.05)	median	OS	No	2	6
Zhao et al.	2016	China	Tissue	Endometrial carcinoma	108	No	FIGO I-IV	qRT-PCR	4.77folds, significant higher(p<0.001)	median	OS	No	2	6
Cui et al.	2015	China	Plasma	Colorectal cancer	60	No	TNM I-IV	qRT-PCR	significant higher(p<0.001)	RQV	OS	No	2	6
Zhang et al.	2015	China	Tissue	Breast cancer	92	No	TNM I-III	qRT-PCR	significant higher(p<0.05)	median	OS,PFS	Multivariate	1	6
Luo et al.	2014	China	Tissue	Non-small cell lung cancer	62	No	TNM II-IV	qRT-PCR	significant higher(p<0.0001)	median	OS	No	2	6
Wang et al.	2015	China	Tissue	Hepatocellular carcinoma	97	No	BCLC 0-C	qRT-PCR	significant higher, more than 2 folds(p<0.05)	2-fold compared with ANT	OS,DFS,RFS	Univariate, multivariate analysis	1	7
McCleland et al.	2015	UK	Tissue	Colorectal cancer	638	N/A	TNM I-IV	ISH assay	significant higher(p<0.05)	ISH score = 1	OS	Multivariate	1	6
Liu et al.	2017	China	Tissue	Gastric cancer	240	No	TNM I-IV	qRT-PCR	significant higher(p<0.001)	0.041 times of 2^-ΔΔCt^	RFS,OS	Univariate, multivariate analysis	1	7

OS: overall survival. DFS: disease free survival. PFS: progression free survival. RFS: recurrence free survival. ΔCt = Ct (CCAT1)—Ct (GAPDH). ANT: adjacent non-tumor tissues. NOS: Newcastle-Ottawa Scale. qRT-PCR: quantitative reverse transcription PCR; ISH: In Situ Hybridization. N/A: not available. RQV: risk quotient value; BCLC: Barcelona-Clinic Liver Cancer; FIGO: International Federation of Gynecology and Obstetrics

*1 denoted as obtaining HRs directly from publications; 2 denoted as extracting HRs from Kaplan-Meier curves.

### Association between CCAT1 and survival in seven types of cancers

**Fig 2:**
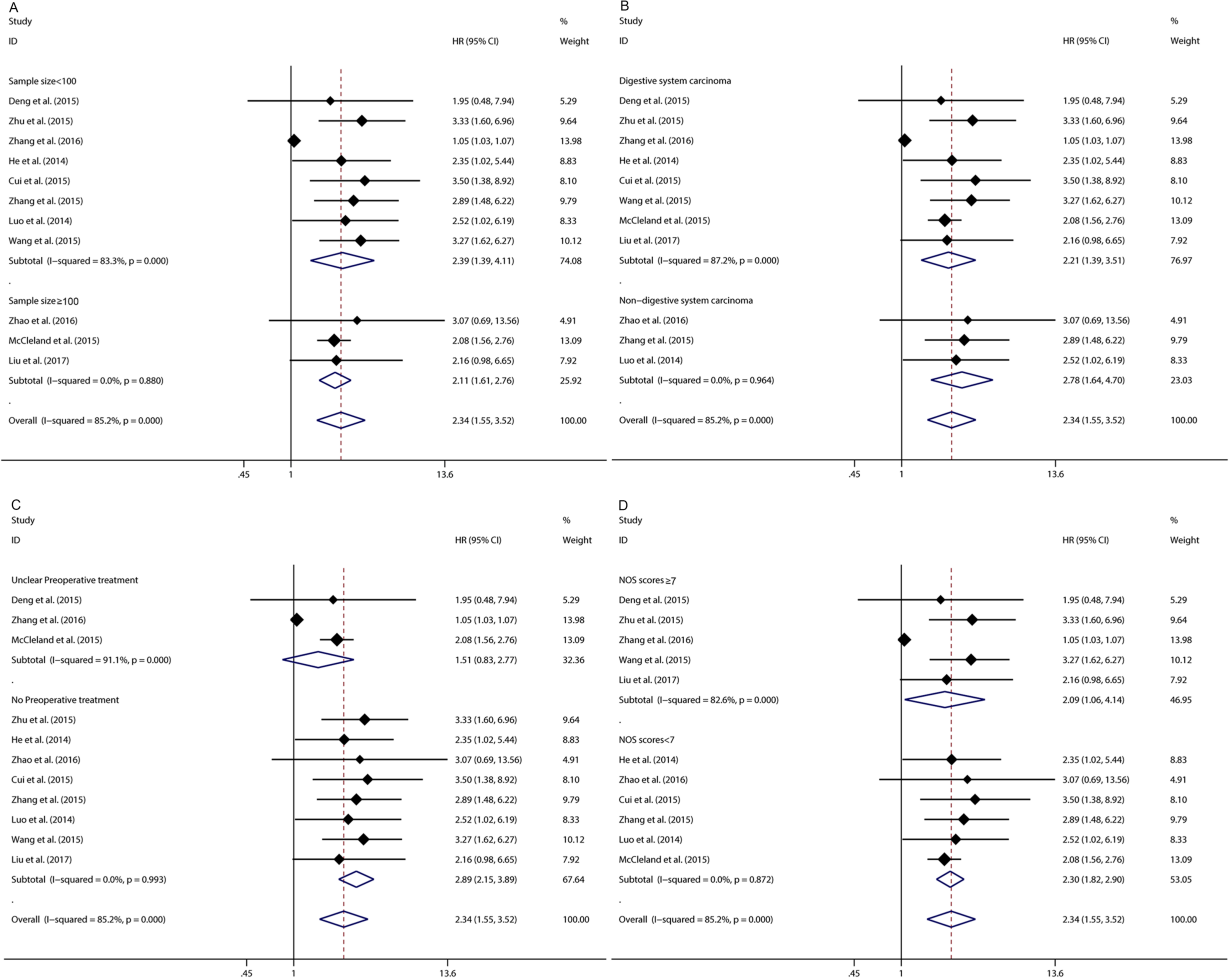
Meta-analysis of the pooled HRs of OS of different types of cancer with increased CCAT1 expression. (A) Subgroup analysis of HRs of OS by factor of sample size. (B) Subgroup analysis of HRs of OS by factor of type of cancer. (C) Subgroup analysis of HRs of OS by factor of preoperative treatment. (D) Subgroup analysis of HRs of OS by factor of paper quality.

Eleven studies reported the overall survival (OS) of seven types of cancer based on different CCAT1 expression levels in a total of 1587 patients. A significant association was found between elevated CCAT1 expression and poor OS in cancer patients (pooled HR 2.335, 95%CI: 1.551–3.517) (**Fig 2**). Significant heterogeneity existed across the studies (Tau^2^ = 0.3121; χ^2^ = 67.57, df = 10, *p* < 0.00001; I^2^ = 85.2%). In order to explore the source of heterogeneity, subgroups were analyzed by factors of the region (China or UK), sample size (more than 100 or fewer than 100), type of cancer (digestive system or non-digestive system malignancies), preoperative treatment (No or unclear) and paper quality (NOS scores ≥7 or <7) (**Fig 2**) (**Table 2**). The result of subgroup analysis showed that association between CCAT1 expression and OS of cancer patients were significant in all the factors above except preoperative treatment. As for the preoperative treatment, CCAT1 was found to be significantly associated with OS in patients without preoperative treatment (HR 2.889, 95% CI: 2.147–3.888) but not in those without clear preoperative treatment (HR 1.513, 95% CI: 0.826–2.772). Significant heterogeneity existed across studies in the subgroup of China by region, in the subgroup of patient number fewer than 100, in the subgroup of patients with digestive system malignancy, in the subgroup of studies without clear preoperative treatment and in the subgroup of paper quality with score more than 7.

**Table 2:** Results of subgroup analysis of pooled hazard ratios of overall survival of different types of cancer with increased CCAT1 expression.

Subgroup analysis	No. of studies	No. of patients	Pooled HR(95%CI)		Meta regression (*p* -value)	Heterogeneity	
			Fixed	Random		I^2^	*p* -value
**Region**							
China	10	949	1.057[1.038–1.076]	2.401[1.483–3.887]	0.832	80.4%	0.000
UK	1	638	2.080[1.564–2.767]	2.080[1.564–2.767]		-	-
**Sample size**							
<100	8	986	1.057[1.038–1.076]	2.393[1.394–4.106]	0.943	83.3%	0.000
≥100	3	601	2.113[1.615–2.765]	2.113[1.615–2.765]		0.0%	0.880
**Type of cancer**							
Digestive system carcinoma	8	1325	1.059[1.040–1.078]	2.212[1.394–3.510]	0.525	87.2%	0.000
Non-digestive system carcinoma	3	262	2.780[1.645–4.699]	2.780[1.645–4.699]		0.0%	0.964
**Preoperative treatment**							
Unclear	3	794	1.056[1.037–1.075]	1.513[0.826–2.772]	0.025	91.1%	0.000
No	8	793	2.889[2.147–3.888]	2.889[2.147–3.888]		0.0%	0.993
**NOS score**							
≥7	4	579	1.055[1.036–1.074]	2.095[1.061–4.136]	0.437	82.6%	0.000
<7	7	1008	2.296[1.819–2.897]	2.296[1.819–2.897]		0.0%	0.872

**Fig 3:**
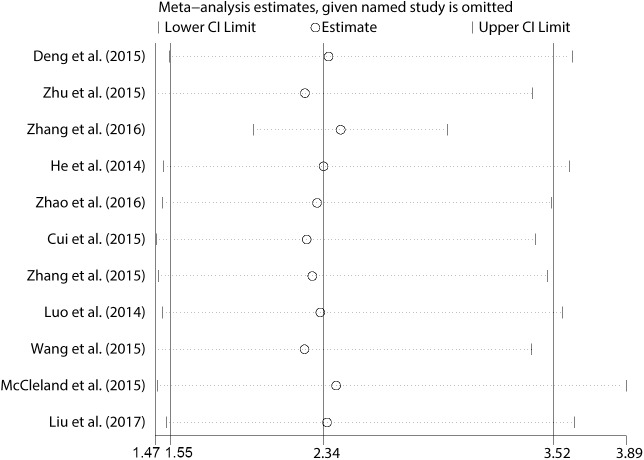
Sensitivity analysis (influence analysis) of the overall pooled study for OS. The study from Zhang et al., 2016 impacted the overall pooled results significantly. The 95% confidence interval of pooled HR and heterogeneity across studies changed notably after excluding that study.

**Fig 4:**
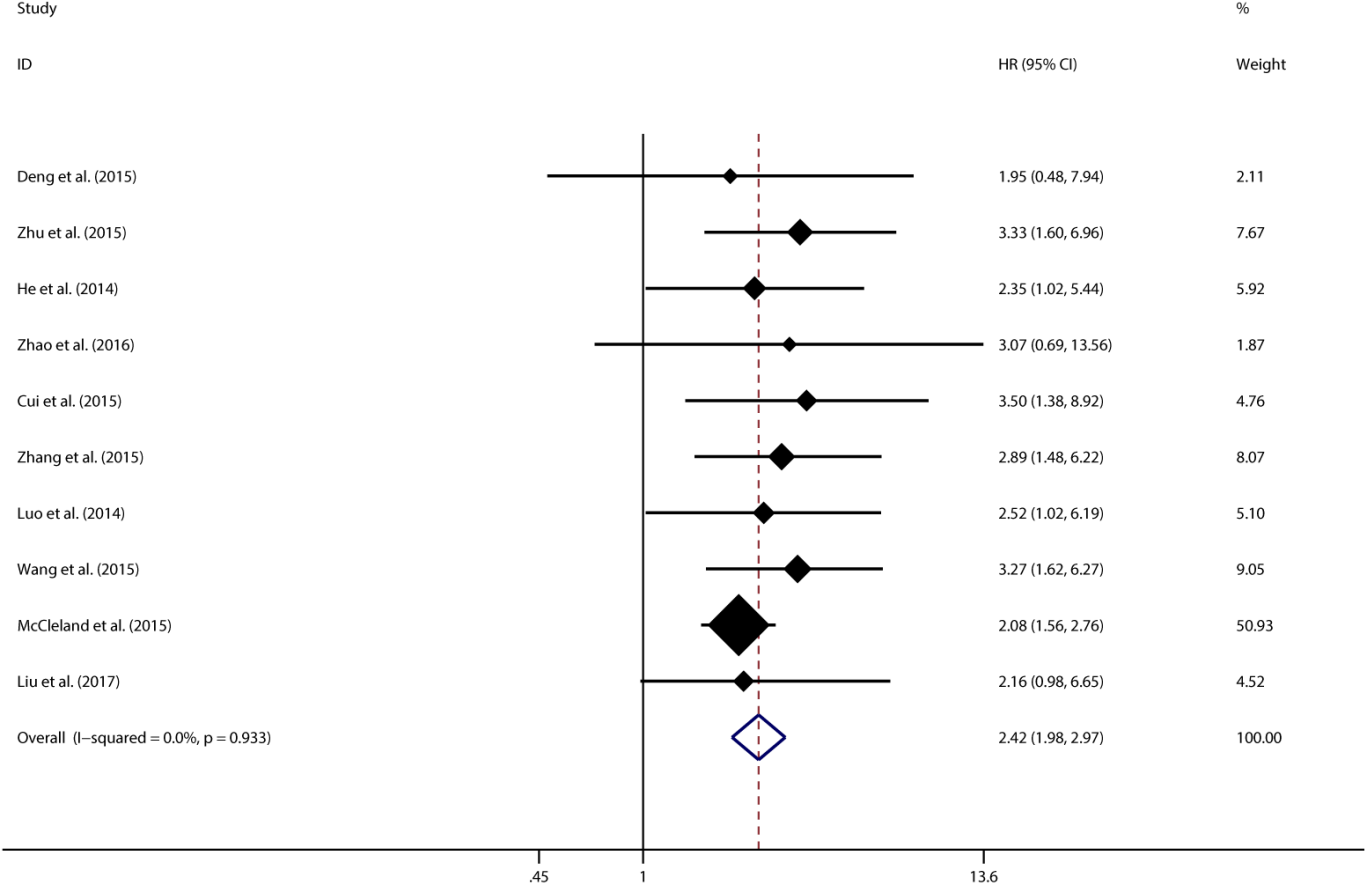
Meta-analysis of pooled HRs of OS of cancer with increased CCAT1 expression after excluding the outlier study.

**Fig 5:**
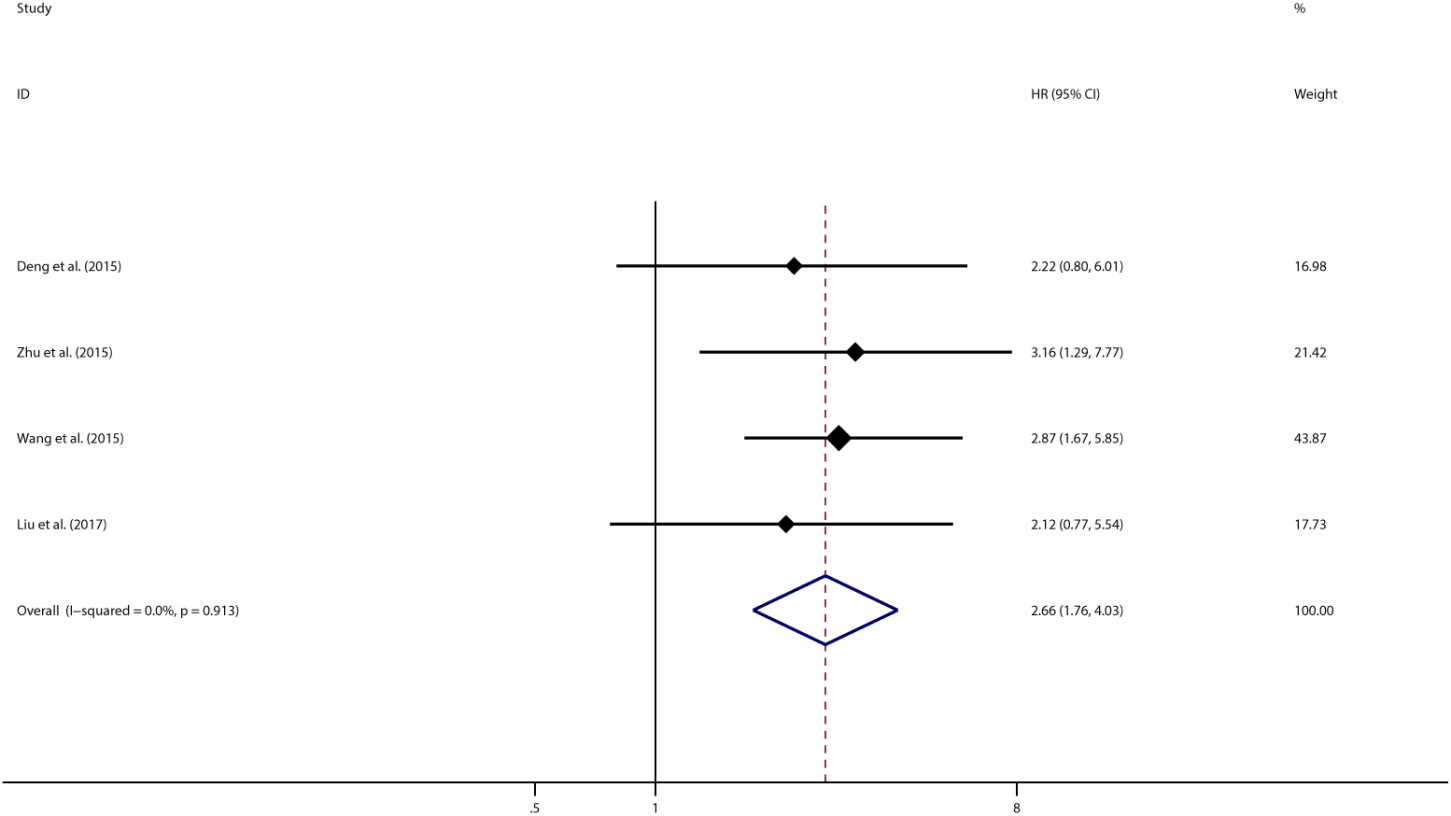
Meta-analysis of pooled HRs of RFS of cancer with increased CCAT1 expression.

In order to further explore the sources of heterogeneity, we performed meta-regression by the covariates including above factors. Meta-regression revealed p values less than 0.05 in the preoperative treatment covariate alone, indicating that preoperative treatment were likely to be the sources of heterogeneity. As shown in **Fig 3**, the sensitivity analysis identified that there was one study from Zhang et al., 2016 impacting the results greatly[15]. The 95% confidence interval of pooled HR change notably after excluding that study, indicating that the study was highly possible to be the main source of heterogeneity. After excluding this study, we still observed a significant association between CCAT1 and OS in cancer patients (pooled HR 2.424, 95%CI:1.977–2.971) (**Fig 4**) but with no significant heterogeneity existed across studies (χ^2^ = 3.65, df = 9, *p* = 0.933; I^2^ = 0.0%). In addition, Begg’s test and Egger’s linear regression test were conducted to evaluate publication bias. The funnel plot showed that there was no significant asymmetry. Begg’s test and Egger’s linear regression test also proved that there was no evidence of publication bias (*p* = 0.858, *p* = 0.135 respectively) (**S1 Fig)**. The prognostic significance of CCAT1 in recurrence-free Survival (RFS) was evaluated in 4 studies with 489 patients. CCAT1 was significantly associated with RFS (HR 2.659, 95%CI: 1.755–4.029) with little heterogeneity (χ^2^ = 0.53, df = 3, *p* = 0.913; I^2^ = 0.0%) (**Fig 5**). There was no significant publication bias across studies in analyzing CCAT1 and RFS (*p* = 0.734 in Begg’s test, *p* = 0.378 in Egger’s test and funnel plot was asymmetrical) **(S2 Fig)**.

**Fig 6:**
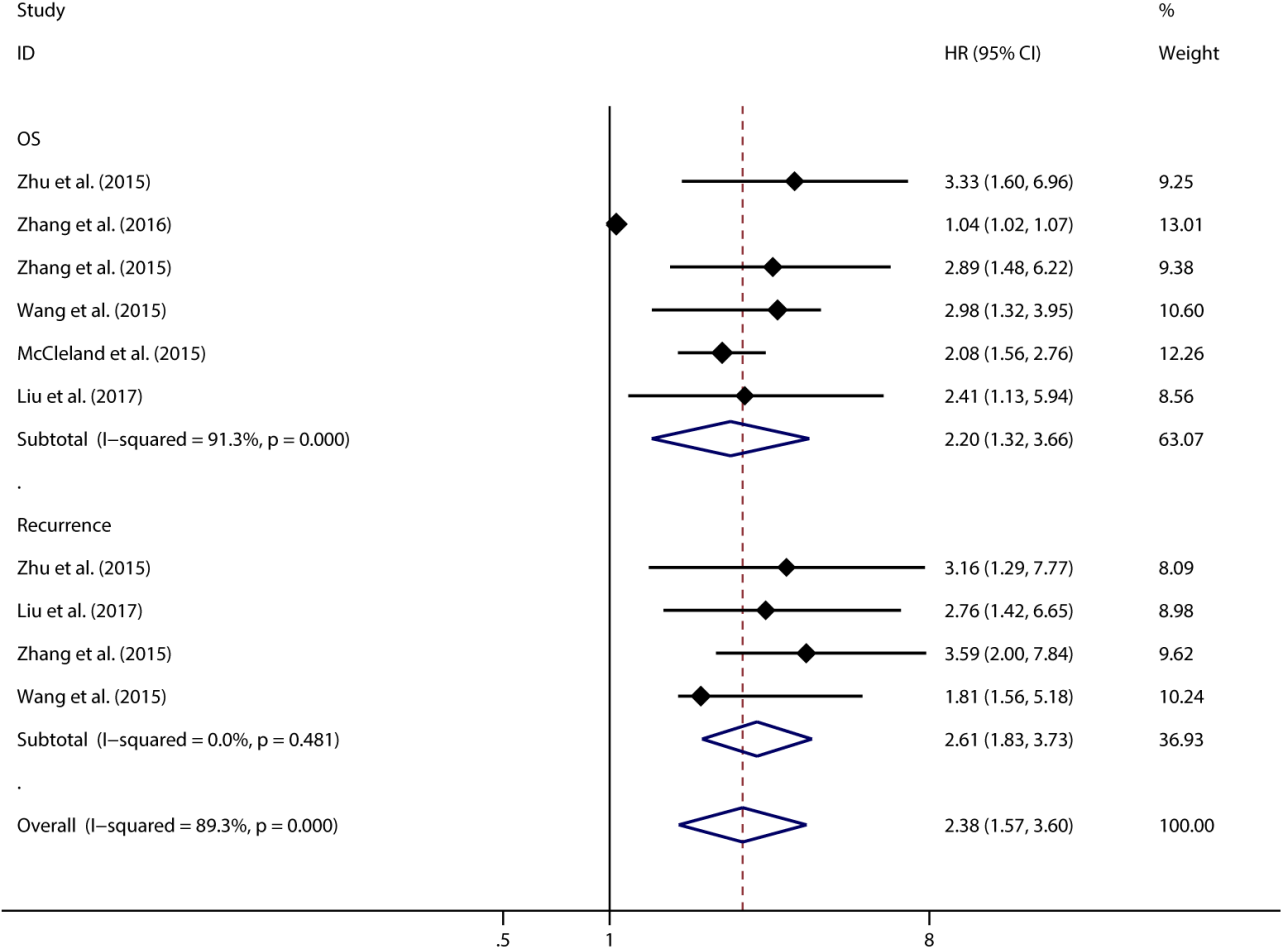
Meta-analysis of the independent role of CCAT1 in OS and recurrence of different types of cancer.

**Fig 7:**
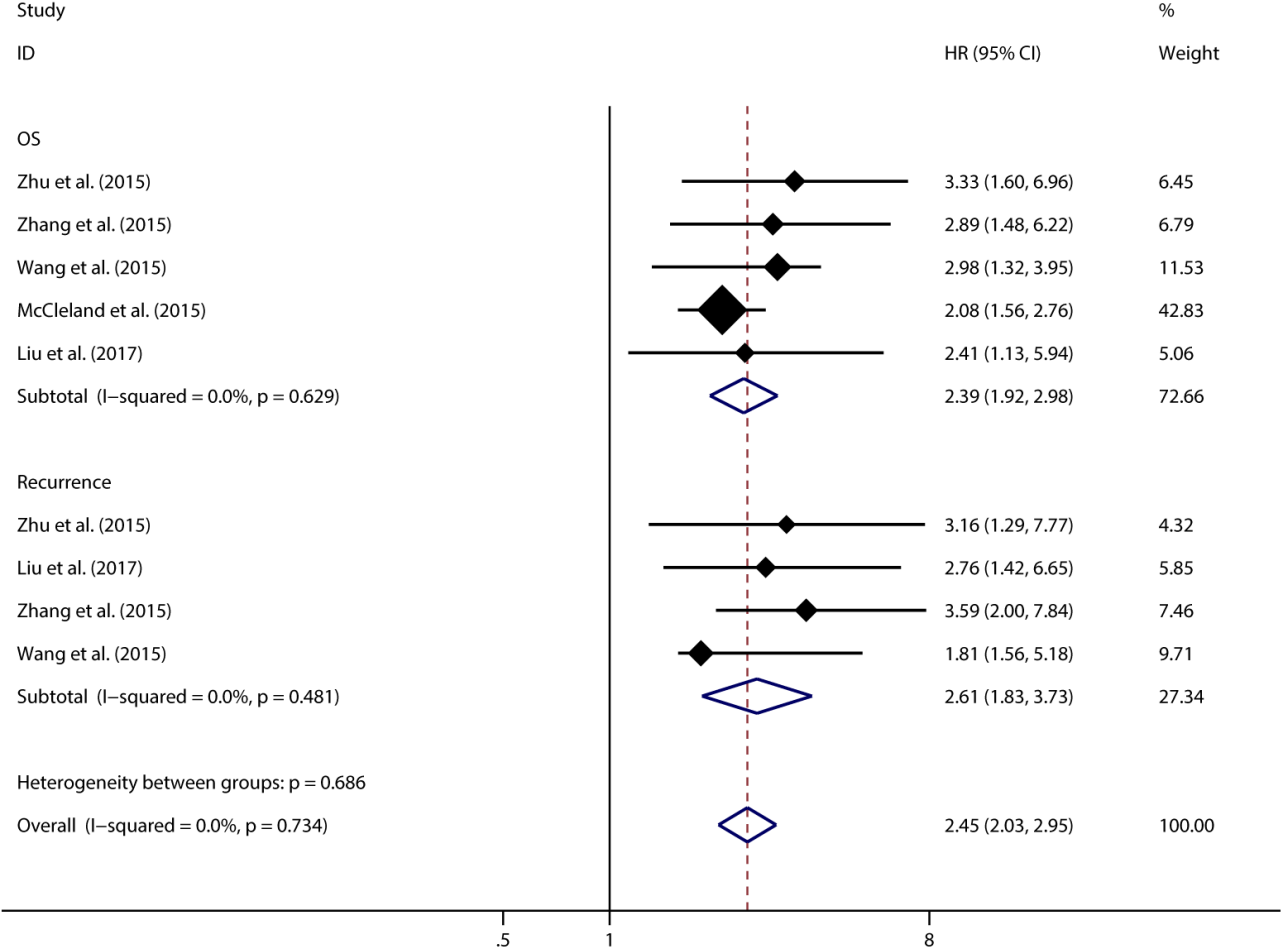
Meta-analysis of the independent role of CCAT1 in OS and recurrence of different types of cancer after excluding the outlier study.

Using Cox multivariate analyses in 6 studies including 1243 patients we found that CCAT1 expression was an independent prognostic factor for OS of cancer patients (pooled HR 2.195, 95%CI:1.316–3.664), but a significant heterogeneity was detected among studies (Tau^2^ = 0.3277; χ^2^ = 57.17, df = 5, *p* < 0.00001; I^2^ = 91.3%). In 4 studies respectively we found that CCAT1 was an independent factor for cancer recurrence (pooled HR 2.609, 95%CI: 1.825–3.728). There was no significant heterogeneity found in studies looking at the independent role of CCAT1 in recurrence (**Fig 6**). Subgroup analysis, sensitivity analysis and meta-regression were performed to explore the heterogeneity across studies concerning the independent role of CCAT1 in OS, but not in the recurrence (**Table 3**). We found that none of the examined factors were likely the source of heterogeneity across studies in meta-regression (*p* > 0.05). Sensitivity analysis showed the same outcome as above. The study from Zhang et al., 2016 was likely to be the main source of heterogeneity (**S3 Fig**)[15]. After excluding this study, we observed that CCAT1 was still an independent prognostic factor for OS of cancer (pooled HR 2.392, 95%CI: 1.922–2.978) with no significant heterogeneity existed between remain studies (χ^2^ = 2.58, df = 4, *p* = 0.629; I^2^ = 0.0%) (**Fig 7**). Begg’s test (*p* = 0.806) and Egger’s test (p = 0.082) showed no significant publication bias across studies, although the funnel plot was asymmetrical (**S4 Fig**).

**Table 3:** Results of subgroup analysis of the independent role of CCAT1 in overall survival/recurrence of different types of cancer.

Subgroup analysis	No. of studies	No. of patients	Pooled HR(95%CI)		Meta regression (*p* -value)	Heterogeneity	
			Fixed	Random		I^2^	*p* -value
**Overall survival**	6	1243	1.052 [1.030–1.073]	2.195[1.316–3.664]		91.3%	0.000
**Region**							
China	5	605	1.048[1.027–1.070]	2.262[1.165–4.392]	0.935	88.6%	0.000
UK	1	638	2.080[1.564–2.767]	2.080[1.564–2.767]		-	-
**Sample size**							
<100	4	365	1.047[1.026–1.069]	2.240[1.048–4.787]	0.958	90.4%	0.000
≥100	2	878	2.113[1.613–2.767]	2.113[1.613–2.767]		0.0%	0.742
**Type of cancer**							
Digestive system carcinoma	5	1151	1.051[1.029–1.073]	2.089[1.211–3.606]	0.611	91.9%	0.000
Non-digestive system carcinoma	1	92	2.891[1.412–5.918]	2.891[1.412–5.918]		-	-
**Preoperative treatment**							
Unclear	2	728	1.048[1.026–1.069]	1.451[0.739–2.850]	0.093	95.5%	0.000
No	4	515	2.925[2.078–4.116]	2.925[2.078–4.116]		0.0%	0.953
**NOS score**							
≥7	4	513	1.047[1.026–1.069]	2.137[1.018–4.486]	0.767	89.0%	0.000
<7	2	730	2.176[1.669–2.836]	2.176[1.669–2.836]		0.0%	0.403
**Recurrence**	4	515	2.609[1.825–3.728]	2.609[1.825–3.728]	-	0.0%	0.481

### Association between CCAT1 and clinicopathological characteristics of cancers

As shown in **Table 4**, eight studies examined the association between CCAT1 and the clinicopathological characteristics of six types of cancer. Four studies examined the association between TNM stage and CCAT1 in different cancers, including colorectal cancer (2), esophageal squamous cell carcinoma (1) and breast cancer (1). There was a significant association in colorectal cancer (pooled OR 1.924, 95%CI: 1.365–2.713) and breast cancer (OR 6.908, 95%CI: 2.647–18.028), while there was no significant association in esophageal squamous cell carcinoma (OR 2.175, 95% CI: 0.869–5.445). Four studies examined the association between tumor size and CCAT1 in hepatocellular carcinoma (2), colorectal cancer (1) and gastric cancer (1). There was a significant association in hepatocellular carcinoma (pooled OR 2.664, 95%CI: 1.399–5.072) and colorectal cancer (OR 5.464, 95%CI: 1.627–18.357), but there was no significant association in gastric cancer (OR 1.414, 95% CI: 0.838–2.387). There were five studies examining lymph node metastases from colorectal cancer (1), esophageal squamous cell carcinoma (1), endometrial carcinoma (1), breast cancer (1) and gastric cancer (1). We observed a significant association for all the five types of cancer. There was one study that examined distant metastases. CCAT1 was significantly associated with distant metastasis in gastric cancer (OR 2.345, 95%CI: 1.226–4.486). There were two studies examining microvascular invasion and capsular formation in hepatocellular carcinoma. We observed significant association between CCAT1 and these two clinicopathological characteristics (pooled OR 4.523, 95%CI: 2.157–9.480; pooled OR 0.419, 95%CI: 0.214–0.818 respectively). In addition, none of the studies demonstrated significant association between CCAT1 expression level with patients’ age or gender (*p* > 0.05). Subgroup analysis, sensitivity analysis and appraisal of publication bias was not performed due to the limited number and relative homogeneity of the studies.

**Table 4:** Results of meta-analysis of increased CCAT1 expression and clinicopathological features in various cancers.

Cancer types	No. of studies	No. of patients	Pooled OR		Heterogeneity	
			Fixed	Random	I^2^	*p* -value
**TNM stage**						
Colorectal cancer	2	686	1.924[1.365–2.713]	2.849[0.834–9.733]	72.1%	0.058
Esophageal squamous cell carcinoma	1	90	2.175[0.869–5.445]	2.175[0.869–5.445]	-	-
Breast cancer	1	92	6.908[2.647–18.028]	6.908[2.647–18.028]	-	-
**Tumor Size**						
Hepatocellular carcinoma	2	163	2.664[1.399–5.072]	2.663[1.396–5.080]	0.0%	0.480
Colorectal cancer	1	48	5.464[1.627–18.357]	5.464[1.627–18.357]	-	-
Gastric cancer	1	240	1.414[0.838–2.387]	1.414[0.838–2.387]	-	-
**Lymph node metastasis**						
Colorectal cancer	1	48	5.000[1.448–17.271]	5.000[1.448–17.271]	-	-
Esophageal squamous cell carcinoma	1	90	2.480[1.060–5.803]	2.480[1.060–5.803]	-	-
Endometrial Carcinoma	1	108	3.571[1.072–11.901]	3.571[1.072–11.901]	-	-
Breast cancer	1	92	5.882[1.569–22.047]	5.882[1.569–22.047]	-	-
Gastric cancer	1	240	2.349[1.394–3.956]	2.349[1.394–3.956]	-	-
**Distant metastasis**						
Gastric cancer	1	240	2.345[1.226–4.486]	2.345[1.226–4.486]	-	-
**Microvascular invasion**						
Hepatocellular carcinoma	2	163	4.523[2.157–9.480]	4.487[2.136–9.426]	0.0%	0.674
**Capsular formation**						
Hepatocellular carcinoma	2	163	0.419[0.214–0.818]	0.417[0.214–0.816]	0.0%	0.487

## Discussion

Cancer is a major public health problem worldwide and is one of the leading causes of death in the USA[37]. The 5-year survival of many types of human cancers is still pretty low. Therefore, it is necessary and significant for us to search and identify new potential biomarkers for early diagnosis and prognosis of cancers.

In recent years, mounting evidence has demonstrated that lncRNAs are important regulatory molecules in diverse biological and pathological processes, such as lncRNA UCA1 increases the cisplatin resistance of bladder cancer cells[38], lncRNA MALAT1 enhances the metastasis of osteosarcoma cells[39], LncRNA-ROR induces epithelial-to-mesenchymal transition of breast cancer cells[40] and lncRNA CCAT1 promotes the proliferation and migration of hepatocellular carcinoma cells[8]. Many lncRNAs are aberrantly expressed in various types of cancers and correlate with different pathophysiological features of tumor growth and with patient survival, thus making them a promising tool for the prognosis of cancers[11]. The lncRNAs such as MALAT1[41], GAS5[42], ANRIL[43], PVT1[44], CCAT2[45] and HOTAIR[46] etc. were found to be novel promising biomarkers to predict a poor prognosis in human cancers.

CCAT1 was initially found in colon cancer[47]. Recently, the function and role of CCAT1 has been extensively investigated in various types of cancer [48]. Ma et al. found that Long non-coding RNA CCAT1 promotes gallbladder cancer development via negative modulation of miRNA-218-5p[28]. Zhuang and Deng et al. revealed that CCAT1 promotes the proliferation and migration of hepatocellular carcinoma cells and laryngeal squamous cell carcinoma cells by functioning as a molecular sponge for let-7 and enhanced the expression of HMGA2 and Myc, the direct target genes of let-7[8, 29]. Study from Zhang et al. indicated that CARLo-5 might serve as a pro-oncogenic lncRNA promoting proliferation of gastric cancer and activating the ERK/MAPK pathway[27]. In colon cancer and pancreatic cancer, abnormally expressed CCAT1 promotes cell proliferation and migration[9, 49]. In the esophageal squamous cell carcinoma, Zhang et al. Showed that H3K27 acetylation activated-long non-coding RNA CCAT1 affects cell proliferation and migration by regulating SPRY4 and HOXB13 expression[15]. In non-small cell lung cancer cell line, inhibition of CARLo-5 by siRNA suppressed the proliferation, migration, and invasion of cells and reversed the epithelial-mesenchymal transition[13]. Based on these studies and owing to its functions, targeting CCAT1 may be beneficial to the outcome of cancer patients and CCAT1 may serve as a prognostic biomarker.

However, the sample sizes of these studies are not large enough. We examined 11 independent studies comprising data from a total of 1587 patients. Through systematic analysis, we found that CCAT1 was highly expressed in many types of tumors. By combining the HRs, we found that high CCAT1 expression was a poor prognostic marker for OS in tumor patients (pooled HR 2.335, 95%CI: 1.551–3.517). In addition, CCAT1 can be regarded as a prognostic risk factor (pooled HR 2.659, 95%CI: 1.755–4.029) for RFS in patients. It should be noted that among the included studies, only one study from Wang et al., 2015 reported the association between increased CCAT1 expression and DFS in hepatocellular carcinoma[14], and only one study from Zhang et al., 2015 reported the association between increased CCAT1 expression and PFS in breast cancer[16], thus these information was not performed in meta-analysis. While these studies indicated the prognosis predictive value of CCAT1 for DFS and PFS in patients with corresponding cancers to some extent.

Since significant heterogeneity observed across these studies, subgroup analysis, meta regression analysis and sensitivity analysis were performed to seek the source of heterogeneity. In the aspects of subgroup analysis, factors including region (China or UK), type of cancer (digestive or non-digestive disease), sample size (more or less than 100), and paper quality (NOS score more or less than 7) were found not altering the significant prognosis predictive value of CCAT1 expression in OS for different types of cancer. However, preoperative treatment (No or unclear) was found to alter the significance (HR 2.889, 95%CI: 2.147–3.888 vs. HR 1.513, 95%CI: 0.826–2.772). Meta regression analysis also found that only the preoperative treatment might be the major source of the significant heterogeneity (*p* = 0.025). Through a sensitivity analysis, we found that there was one study from Zhang et al., 2016 impacting the pooled HR and its 95%CI apparently[15]. After excluding this study, the results of both the fixed effect model and random effect model were found to present no observable difference (pooled HR 2.424, 95%CI:1.977–2.971) with no significant heterogeneity existing across remain studies, which indicated that our analysis became stable and overexpression of CCAT1 is associated with poor prognosis. Both Begg’s test and Egger’s test found no significant publication bias on the prognostic role of CCAT1 in different types of cancer. The reason why the study mentioned above caused obvious heterogeneity was analyzed in detail. We think it might be that biological types of carcinoma (esophageal squamous cell carcinoma) could cause notable influence and/or some patients in that study had received preoperative treatment. If the patients had received preoperative chemotherapy or radiotherapy, the true prognostic value of CCAT1 in survival might be impacted greatly. Furthermore, the prognostic significance of CCAT1 in RFS was evaluated in 4 studies with 489 patients. Meta-analysis showed that patients with high CCAT1 expression were more possible to have significantly poorer RFS (pooled HR 2.659, 95%CI: 1.755–4.029) with no significant heterogeneity. Because of the limited number of studies referring to the relationship between CCAT1 and RFS, we thought more studies with large sample size are necessary to draw a definite conclusion. In addition, by combining HRs from Cox multivariate analyses in a total of 6 studies, we found that CCAT1 was an independent prognostic factor in OS for cancer patients (pooled HR 2.195, 95%CI: 1.316–3.664). Subgroup analysis and meta regression showed that none of the examined factors were responsible for the significant heterogeneity. Sensitivity analysis showed definite significance change after excluding the study from Zhang et al., 2016 same as mentioned above. We obtained a more stable and significant result (pooled HR 2.392, 95%CI: 1.922–2.978) presenting CCAT1 as an independent prognostic factor for OS in cancer patients with nearly no heterogeneity. And CCAT1 was found to be an independent prognostic factor (pooled HR 2.609, 95%CI: 1.825–3.728) for cancer in recurrence through obtaining HRs in 4 studies with multivariate analysis method. It should be noted that Egger’s test found there was significant publication bias on the independent prognostic role of CCAT1 in different types of cancer.

In the aspect of association between CCAT1 and clinicopathological characteristics of cancers, our results showed that elevated CCAT1 expression was significantly associated with tumor size, lymph node metastasis, advanced TNM stage, distant metastasis, microvascular invasion and capsular formation in corresponding cancers, which suggests that increased CCAT1 may be associated with advanced features of cancer. However, the prognostic value of CCAT1 for clinicopathological characteristics could be different in diverse types of cancers. For instance, expression level of CCAT1 was found to be significantly associated with TNM stage in colorectal and breast cancer but not in esophageal cancer.

Considering many clinicopathological characteristics of various cancers may relate to patients’ gender and age, we would like to explore if there was any gender or age specific association between CCAT1 and features of different cancers. But information from the 11 included studies was not sufficient enough to conduct these analyses. We think future studies should pay more attention to these aspects. Besides, it’s a common viewpoint that different initiating molecular alterations drive different types or subtypes of cancer. The oncogene c-Myc, which is up regulated and contributes to tumorigenesis and development in various types of cancer, was demonstrated to be functionally related with lncRNA CCAT1 closely[31, 50, 51]. Thus we tried to found out whether the variation of association between CCAT1 and clinicopathological features of different cancers resulted from c-Myc expression level. But unfortunately none of the included studies carried out c-Myc expression detection in patients’ samples. We believe that future studies should research lncRNA CCAT1 along with c-Myc expression on the level of patients’ samples but not only in cell lines.

Although a lot of studies discussed certain mechanisms of CCAT1, it is worth mentioning that it’s quite difficult to figure out the functional importance of the significant association between CCAT1 expression and the tumorigenesis and development of different types of cancer. Therefore, more researches focusing on this filed should be conducted to fill the gap and promote the further study in the future.

Classic biomarkers widely used to monitor cancer now often show unsatisfactory sensitivity and specificity. Such as, alfa-fetoprotein (AFP) for monitoring HCC often shows a false-positive result during pregnancy, active liver disease, and many other tumors[52–54]. Therefore, identification of more sensitive and specific biomarkers for monitoring cancer is desirable and urgently needed. Compared to classic biomarkers for cancer, we think lncRNA CCAT1 could be a promising prognosis biomarker for several reasons: 1) Our study demonstrated overexpressed CCAT1 was associated significantly with poor prognosis of patients with different cancers, and it was further found to be an independent prognostic factor. 2) Many studies showed that lncRNAs played important roles in biological behavior of tumorigenesis, including proliferation, apoptosis, migration and metastasis[36, 49]. And through the interaction with protein coding genes and miRNAs, lncRNAs are more likely to reflect tumor biology features and phenotypes of cancer than other biomarkers. And by combination of certain proteins and miRNAs expression with lncRNAs, the predictive efficacy could be more powerful. For example, CCAT1 correlate with oncogene c-Myc, which is an important factor in tumorigenesis and progression in various types of cancers. 3) According to the current situation, high throughput sequencing technology are becoming affordable and popularized, lncRNA CCAT1 may become a novel prognosis biomarker for cancer.

In our study, a few limitations should be underlined. First, the cut-off values of high and low CCAT1 expression were different among studies, although most of them were set to median. Second, we only included English and Chinese language papers. And most studies are from China, the results may mainly represent Chinese cancer patients. Third, differences of paper quality and sample size across the studies might cause bias in the meta-analysis although subgroup analysis and meta regression did not show the paper quality or sample size as the resource of heterogeneity. Fourth, HRs of five studies could not be directly obtained from the publications. Thus, calculating them through survival curves might not be precise enough. Fifth, most of the included studies reported positive results so our results might overestimate the prognostic significance of CCAT1 in a variety of cancer to some degree. Sixth, because there were few studies on association between expression of CCAT1 with MFS (metastasis free survival), prognostic significance of CCAT1 in metastasis was not investigate. Therefore, larger-scale, multicenter, and high-quality studies are highly necessary to confirm our findings. Besides, The Cancer Genome Atlas (TCGA) and the GEO databases of the National Cancer Institute in the USA scheduled a large amount of patients in many types of cancer from around the world. Different techniques including RNA-sequencing were used to analyze the patient samples. Hence through re-analyzing the abundant RNA-sequencing raw data containing lncRNA CCAT1 expression and the corresponding information for the patients’ prognosis, a significant extension of our finding and re-analysis, which will include more patients from different region other than the Chinese group, could be accomplished in near future.

## Conclusion

Our study found that CCAT1 overexpression might be a promising predictive factor for assessing prognosis in various types of cancer. And the expression level of CCAT1 was associated with clinicopathological features such as TNM stage, lymph node metastasis, and distant metastasis in cancers. This meta-analysis is the first to demonstrate that high expression of the lncRNA CCAT1 is related to poor prognosis for cancer patients. In the future, more studies will be necessary to investigate the role of CCAT1 in human cancer.

## Supporting information

S1 Checklist PRISMA checklist. Each section was localized in the paper.
(DOC)

S1 Fig Publication bias for the analysis of the association between CCAT1 expression and OS. (A) Funnel plot/ (B) Begg’s test graph/ (C) Egger’s test graph.
(TIF)

S2 Fig Publication bias for the analysis of the association between CCAT1 expression and RFS. (A) Funnel plot/ (B) Begg’s test graph/ (C) Egger’s test graph.
(TIF)

S3 Fig Sensitivity analysis (influence analysis) of the independent role of CCAT1 expression for OS. (TIF)

S4 Fig Publication bias for the analysis of the independent prognostic role of CCAT1 expression for OS. (A) Funnel plot/ (B) Begg’s test graph/ (C) Egger’s test graph.
(TIF)

S1 File A full electronic search strategy and procedure for Pubmed. (DOC)

S1 Table A detailed table of quality assessment for included studies. (DOC)
