# Correction: Prognostic value of long non-coding RNA CCAT1 expression in patients with cancer: A meta-analysis

**DOI:** 10.1371/journal.pone.0186670

**Published:** 2017-10-12

**Authors:** Deyao Shi, Fashuai Wu, Feng Gao, Xiangcheng Qing, Zengwu Shao

There is an error in the authors’ affiliation. The affiliation should be: Department of Orthopaedics, Union Hospital, Tongji Medical College, Huazhong University of Science and Technology, Wuhan 430022, China.
