# Expression of Concern: Prognostic value of long non-coding RNA CCAT1 expression in patients with cancer: A meta-analysis

**DOI:** 10.1371/journal.pone.0284940

**Published:** 2023-04-20

**Authors:** The PLOS ONE Editors

After this article was published, similarities were noted between this article [1, 2] and submissions by other research groups which call into question the validity and provenance of the reported results.

In response to queries about these concerns, the first author provided the underlying data in S1 File.

The authors commented on aspects of how data were collected and analyzed for this study. The *PLOS ONE* Editors concluded that the data and information provided in post-publication discussions lent some assurance as to the results reported in the article, but did not address the concerns about similarities with other published and submitted work.

The *PLOS ONE* Editors issue this Expression of Concern to notify readers of the unresolved concerns discussed above, and to provide the data received from the authors.

## Supporting information

S1 File The underlying data used for the analysis in this article [1]. (ZIP)
