# Supplementary figures and images for: Prognostic value of long non-coding RNA CCAT1 expression in patients with cancer: A meta-analysis
Source: PLoS One. 2017 Jun 8;12(6):e0179346. doi: 10.1371/journal.pone.0179346 (PMC5464649; doi:10.1371/journal.pone.0179346)

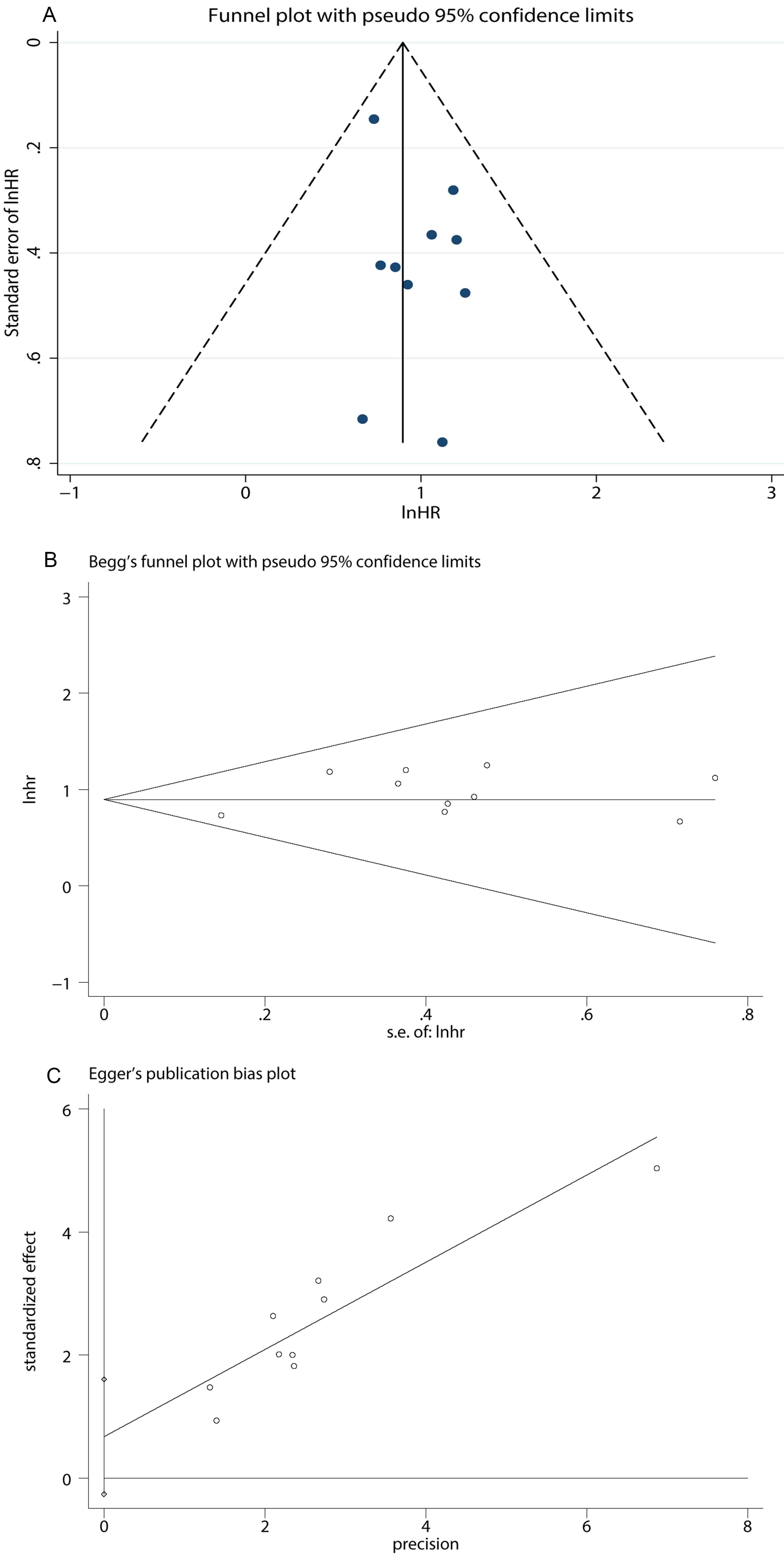

Supplement: S1 Fig — (A) Funnel plot/ (B) Begg’s test graph/ (C) Egger’s test graph. (TIF) [file pone.0179346.s002.tif]

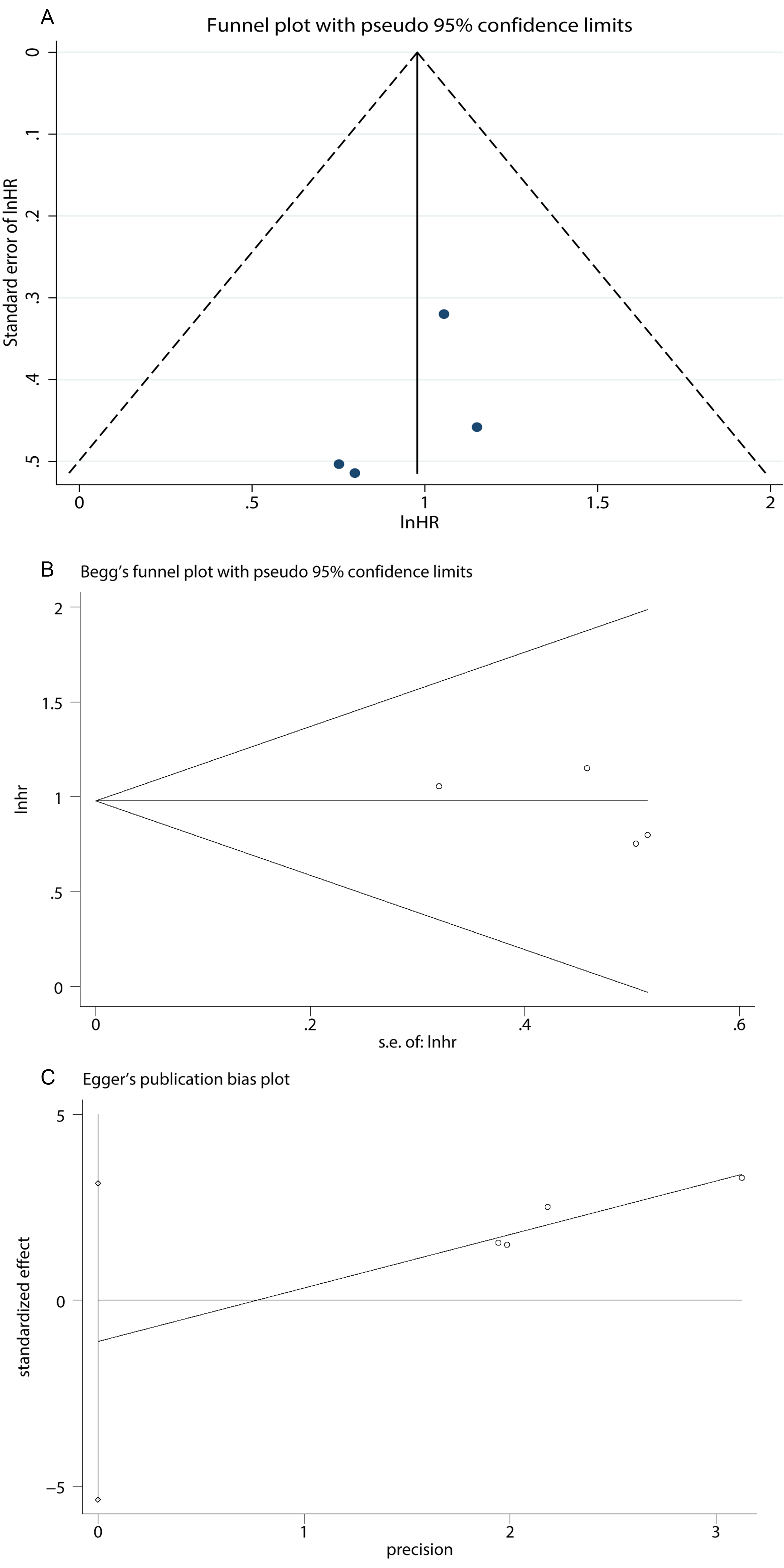

Supplement: S2 Fig — (A) Funnel plot/ (B) Begg’s test graph/ (C) Egger’s test graph. (TIF) [file pone.0179346.s003.tif]

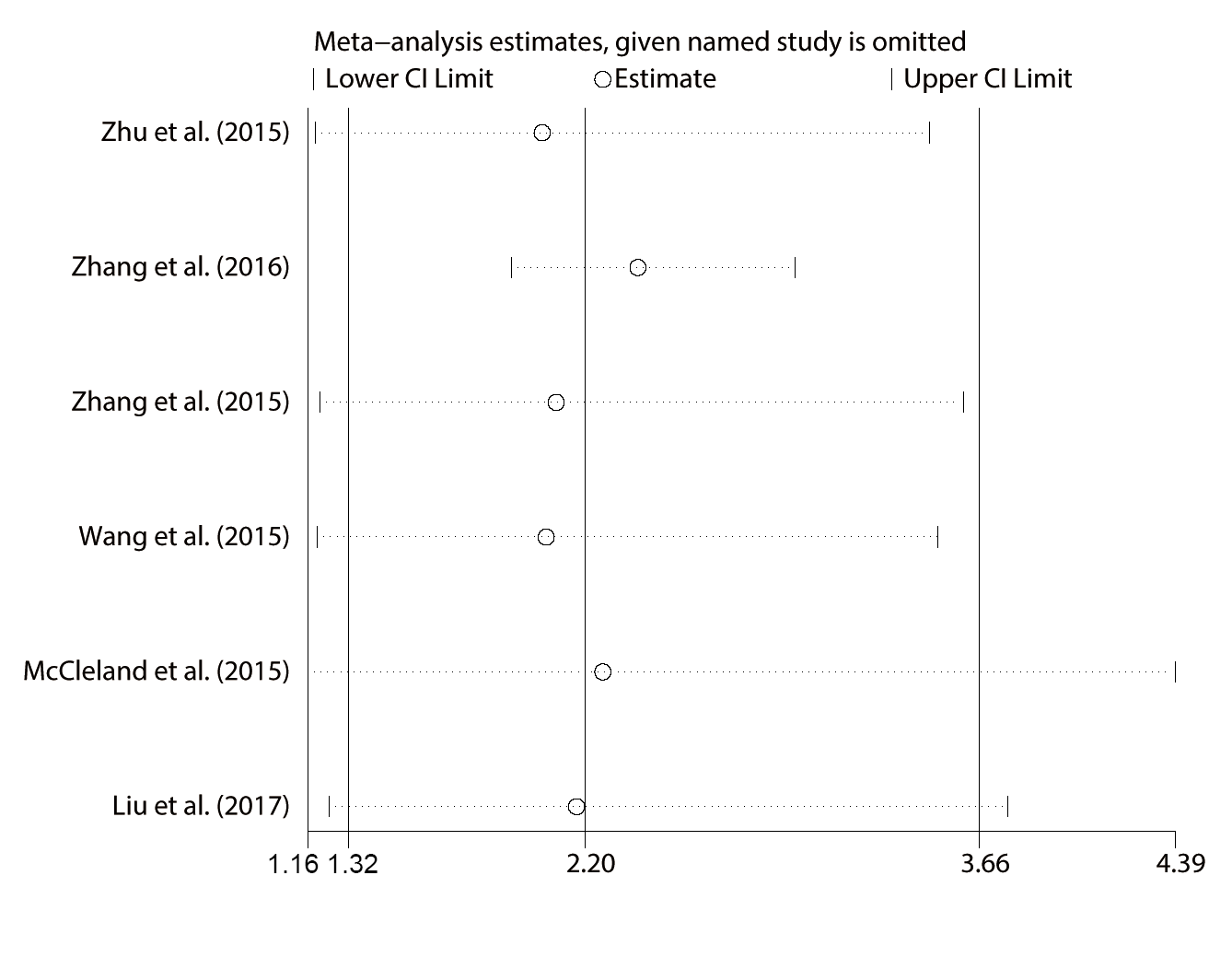

Supplement: S3 Fig — (TIF) [file pone.0179346.s004.tif]

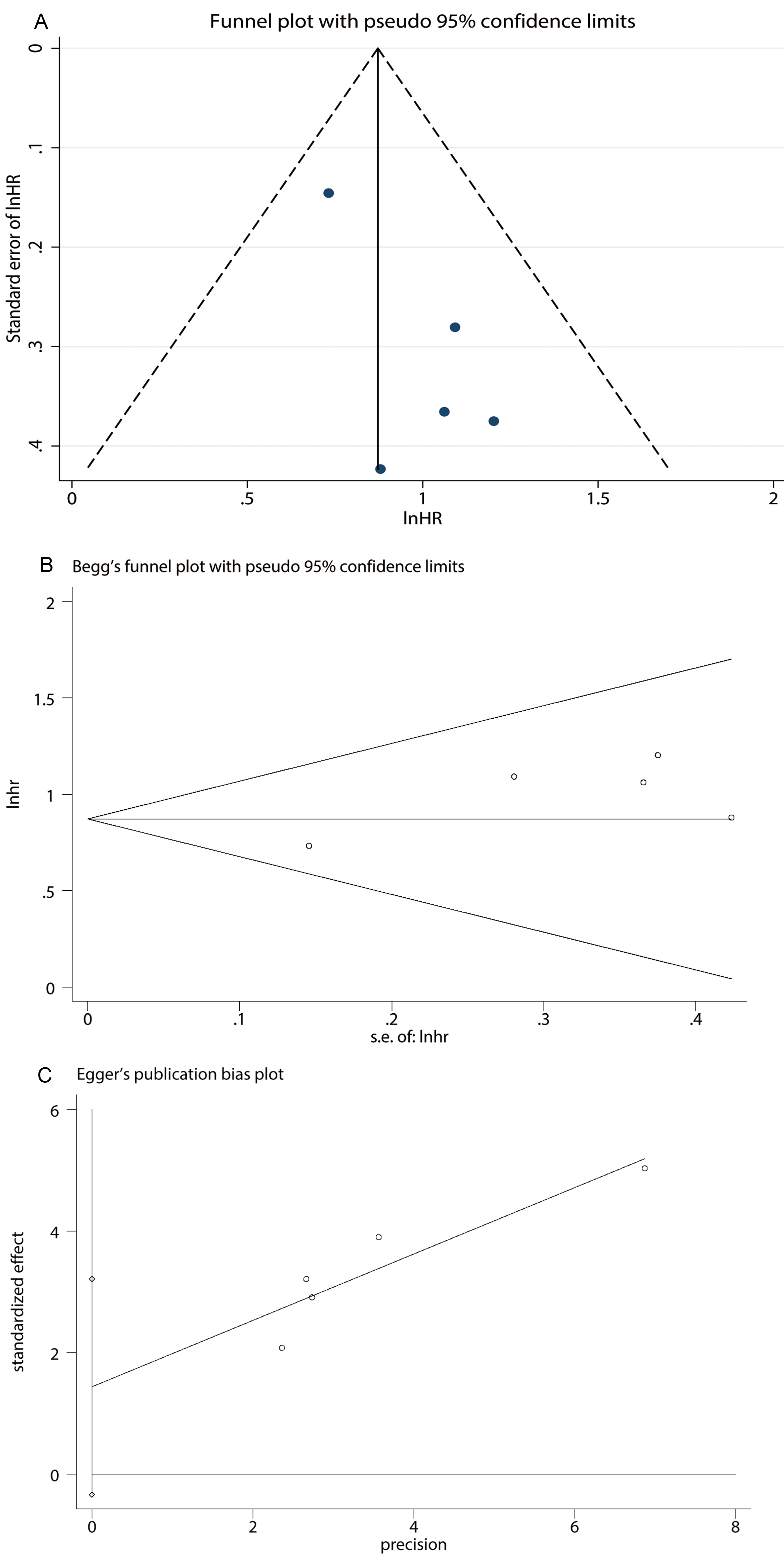

Supplement: S4 Fig — (A) Funnel plot/ (B) Begg’s test graph/ (C) Egger’s test graph. (TIF) [file pone.0179346.s005.tif]
