# Supplementary material for: Expression of Concern: Prognostic value of long non-coding RNA CCAT1 expression in patients with cancer: A meta-analysis
Source: PLoS One. 2023 Apr 20;18(4):e0284940. doi: 10.1371/journal.pone.0284940 (PMC10118116; doi:10.1371/journal.pone.0284940)
Supplement: S1 File — (ZIP) [file pone.0284940.s001.zip › 3 The 11 included studies in PDF/10.pdf]

Tumor Biol.
DOI 10.1007/s13277-014-2442-7

RESEARCH ARTICLE

Long non-coding RNA *CARLo-5* is a negative prognostic factor and exhibits tumor pro-oncogenic activity in non-small cell lung cancer

Jie Luo · Liang Tang · Jie Zhang · Jian Ni · Hai-ping Zhang · Ling Zhang · Jian-fang Xu · Di Zheng

Received: 24 June 2014 / Accepted: 4 August 2014
© International Society of Oncology and BioMarkers (ISOBM) 2014

Abstract Despite recent advances in the management of non-small cell lung cancer (NSCLC), the prognosis of NSCLC is still relatively poor. Thus, it is of great importance to identify novel effective diagnostic and prognostic biomarker of NSCLC. A growing volume of literature has demonstrated the vital roles of long non-coding RNAs (lncRNAs) in cancer biology. Cancer-associated region long non-coding RNA (*CARLo-5*), a recently identified lncRNA, was initially characterized in colon cancer. However, little is known about its role in NSCLC. In the present study, a great upregulation of *CARLo-5* was observed in cancer tissues compared to their adjacent normal tissues. Meanwhile, patients with high *CARLo-5* expression have significantly poorer prognosis than those with low expression. Inhibition of *CARLo-5* by siRNA suppressed the proliferation, migration, and invasion in NSCLC cell lines in vitro. In addition, silencing of *CARLo-5* reversed the epithelial–mesenchymal transition in NSCLC cell line. Our study suggests that *CARLo-5* may represent a prognostic marker and a potential therapeutic target of NSCLC.

Keywords Non-small cell lung cancer · Long non-coding RNA · *CARLo-5* · Prognosis · Epithelial–mesenchymal transition

Jie Luo and Liang Tang are equal contributors.

J. Luo · J. Ni · H.-p. Zhang · L. Zhang · J.-f. Xu · D. Zheng (✉)
Department of Medical Oncology, Shanghai Pulmonary Hospital, Tongji University School of Medicine, 507 Zhengmin Road, Shanghai 200433, People's Republic of China
e-mail: zhengdiok@aliyun.com

L. Tang · J. Zhang
Central Lab, Shanghai Pulmonary Hospital, Tongji University School of Medicine, Shanghai, China

Introduction

Non-small cell lung cancer (NSCLC), including adenocarcinoma and squamous cell carcinoma, is the most common type of cancer and a leading cause of cancer-related death worldwide [1]. The majority of NSCLC patients are diagnosed at advanced stages as they are usually asymptomatic at early stages [2]. Despite recent advances in surgical techniques and chemoradiation therapy, the prognosis of lung cancer is unfavorable, with a 5-year survival rate of around 11 % [3]. Therefore, it is vital to reveal the molecular mechanisms of the progression of NSCLC for the development of effective therapies.

Non-coding RNAs (ncRNAs), once regarded as "transcriptional noise," have recently been demonstrated to be functional molecules. These protein-non-coding sequences account for the majority of human genome while protein-coding genes only about 2 % [4]. The ncRNAs include not only well-characterized microRNAs and other non-coding transcripts less than 200 nucleotides (nt) but also a large class of long (>200 nt) ncRNAs (lncRNAs), which have emerged as a new layer of cell biology [4, 5]. A growing volume of literature has indicated the vital roles of lncRNAs in cancer biology. Alterations in lncRNAs have been shown to exhibit pro-oncogenic or tumor-suppressive activities [6–10].

A recently identified long non-coding RNA, named cancer-associated region long non-coding RNA (*CARLo-5*), is located in the 8q24.21 gene desert region [11]. It suggests that the cancer-associated variant rs6983267 in MYC enhancer region could regulate *CARLo*-5 expression through long-range interaction with the active regulatory region of its promoter. It was shown to play a role in cell cycle regulation in colon cancer [11]. However, the prognostic role of *CARLo-5* in cancer is elusive, and few studies have examined its molecular mechanism in NSCLC.

Published online: 17 August 2014

In the present study, we explored *CARLo-5* expression pattern and its correlation with clinicopathological factors in NSCLC. Then, its prognostic significance was assessed. The oncogenic role of *CARLo-5* was investigated in NSCLC cell lines.

Materials and methods

Cell culture

Three NSCLC adenocarcinoma cell lines (A549, SPC-A1, NCI-H1975), a NSCLC squamous carcinomas cell line (SK-MES-1), and a normal human bronchial epithelial cell line (16HBE) were purchased from the Institute of Biochemistry and Cell Biology of the Chinese Academy of Sciences (Shanghai, China). The cell lines were cultured in DMEM or RPMI 1640 (Gibco BRL), containing 10 % fetal bovine serum (FBS, HyClone) as well as 100 U/ml penicillin and 100 μg/ml streptomycin (Invitrogen). Cells were maintained in a humidified incubator at 37 °C in the presence of 5 % CO_2. All cell lines have been passaged for fewer than 6 months.

Patient samples

The study was undertaken with the understanding and written consent of each subject. The study methodologies conformed to the standards set by the Declaration of Helsinki. This study was approved by the Human Ethics Committee of Pulmonary Hospital at Tongji University (Shanghai, China). Sixty-two paired NSCLC and adjacent non-tumor lung tissues (≥3 cm away from tumor) were obtained from patients who underwent surgery at pulmonary hospital between 2008 and 2011 and were diagnosed with NSCLC (stages II, III, and IV) based on histopathological evaluation. Each sample was snap-frozen in liquid nitrogen and stored at −80 °C prior to RNA isolation and qRT-PCR analysis. All patients recruited to this study did not receive any pre-operative treatments. Complete clinicopathological data of the patients from which the specimens were collected were available. Overall survival (OS) was defined as the interval between the dates of surgery and death.

RNA extraction and quantitative real-time PCR

Total RNA from tissues and cells was extracted using TRIzol reagent (Invitrogen, CA) according to the manufacturer's protocol. RNA was reversed transcribed into cDNAs using the Primer-Script™ one step RT-PCR kit (TaKaRa, Dalian, China). The cDNA template was amplified by real-time RT-PCR using the SYBR® Premix Dimmer Eraser kit (TaKaRa, Dalian, China). The quantitative real-time polymerase chain reaction (qRT-PCR) was performed using the SYBR Select Master Mix (Applied Biosystems, cat. 4472908) on ABI 7500 system (Applied Biosystems, CA, USA) according to the manufacturer's instructions. Glyceraldehyde-3-phosphate dehydrogenase (GAPDH) was measured as an internal control for cell lines, and β-actin was measured as an internal control for paired tumor and normal tissues. The relative expression fold change of mRNAs was calculated by the $2^{-\Delta\Delta Ct}$ method. After the reverse transcription, 0.5 μl of the complementary DNA was used for subsequent qRT-PCR reaction. The primer sequences were as follows: GADPH: 5′-GTCAACGGATTT GGTCTGTATT-3′ (forward), 5′-AGTCTTCTGGGTGGCA GTGAT-3′ (reverse); β-actin: 5′-GAAATCGTGCGTGACA TTAA-3′ (forward), 5′-AAGGAAGGCTGGAAGAGTG-3′ (reverse); and *CARLo-5*: 5′-GCCACAAATCAACAACAA CAACAACAA-3′ (forward), 5′-AGAGTGATGCCAAGGC TGTTATTGTCAA-3′ (reverse). The qRT-PCR reaction was conducted under the following conditions: 95 °C for 30 s, 40 cycles of 95 °C for 5 s, and 60 °C for 60 s. For cell expression and tumor samples, each sample was run in triplicate. qRT-PCR results were analyzed and expressed relative to threshold cycle (CT) values and then converted to fold changes.

Plasmid and transfection

The *CARLo-5*-specific siRNA sequences were as follows: *CARLo-5* siRNA-1 (si*CARLo5*-1): GGAGGGUGCUUGAC AAUAAUU; CARLo-5 siRNA-2 (si*CARLo5*-2): GAGAAG ACCAUAAGAAGAU. Allstars Negative Control siRNA were purchased from Qiagen, Hilden, Germany. Cells were grown on six-well plates to 70 % confluency and transfected using Lipofectamine 2000 (Invitrogen) according to the manufacturer's instructions, and siRNAs were used at 50 nM final concentration. Forty-eight hours after transfection, cells were harvested for qRT-PCR or Western blot analyses.

Cell proliferation assay

Cell proliferation assays were conducted using the CCK-8 assay kits as described by the manufacturer.

Western blot analysis

The harvested cells were centrifuged at 6,000 rpm for 1 min. The total cellular proteins were prepared using RIPA cell lysis buffer (Cell Signaling Technology) supplemented with protease inhibitors. The lysates were then collected and subjected to ultrasonication and centrifugation. The supernatants were collected, and protein content was determined by Bradford assay. Equal amounts (30–50 μg) of proteins were applied to an 8–12 % SDS-polyacrylamide separating gel and transferred to a PVDF Immobilon-P membrane (Millipore). The membrane was blocked with 5 % skim milk in Tris-buffered saline with Tween 20 (TBST) and then probed with indicated primary

antibodies with gentle shaking at 4 °C overnight. The membranes were washed with TBST (3×10 min) and incubated in secondary antibodies at room temperature for 1 h. Antibody-bound proteins were detected by BeyoECL Plus kit.

The primary antibodies used in these experiments include E-cadherin (1:1,000) (Abcam), fibronectin (1:1,000) (Abcam), vimentin (1:500) (Abcam), Snail (1:1,000) (Abcam), Twist (1:500) (Abcam), Zeb1 (1:500) (Abcam), and Slug (1:500) (Abcam). HRP-conjugated goat anti-rabbit IgG antibody (Abcam) was used as the secondary antibody.

Flow cytometric analysis

Cells were seeded at a density of 1×10^6 cells/well in six-well plates. After 24 h, cells were washed with PBS and fixed in ice-cold 70 % ethanol for 1 h and then treated with 100 μL of 50 mg/L propidium iodide for 30 min at 4 °C in the dark. The cell cycle profiles were assayed using the Elite ESP flow cytometer at 488 nm, and data were analyzed with the CELL Quest software (BD Biosciences, San Jose, CA, USA).

Cell migration and invasion assay

For the migration assays, 48 h after transfection, 5×10^4 cells in serum-free media were placed into the upper chamber of an insert (8.0 μm, Millipore, MA). For the invasion assays, the upper chamber was pre-coated with Matrigel (Sigma, USA) and 1×105 cells were seeded. The chambers were then incubated for 24 h in culture medium with 10 % FBS in the bottom chambers before examination. The cells on the upper surface were scraped and washed away, whereas the cells on the lower surface were fixed and stained with 0.05 % crystal violet for 2 h. Finally, cells were counted under a microscope and the relative number was calculated. Experiments were independently repeated in triplicate.

Immunofluorescence microscopy

Cells were grown on glass chamber slides. For membrane staining (E-cadherin), cells were fixed by incubation with cold 100 % methanol for 10 min. For intracellular staining (vimentin, fibronectin), the cells were fixed with 4 % (wt/vol) paraformaldehyde in PBS and permeabilized by incubation with 0.5 % Triton X-100 in PBS for 1 min. The cells were incubated with 3 % bovine serum albumin in PBS for 30 min at room temperature. After washing with PBS, the cells were incubated with specific primary antibody at 4 °C overnight. The cells were then washed and incubated with Alexa Fluor 633-conjugated goat anti-rabbit IgG diluted in blocking solutions and incubated for 1 h. The nuclei were stained with 4,6-diamidino-2-phenylindole (DAPI). Sections were visualized by fluorescence microscopy.

Statistical analysis

All statistical analyses were performed using SPSS 17.0 (SPSS, Chicago, USA). The gene expression level of *CARLO-5* in tumors was compared with adjacent normal tissues utilizing the paired sample t test, whereas the association between *CARLO-5* expression and clinical characteristics was evaluated using the Mann–Whitney test. Survival curves were plotted by the Kaplan-Meier method, and the log-rank comparison was carried out to assess differences between stratified survival groups using the median value as the cutoff. A Cox proportional hazards analysis was performed to calculate the hazard ratio (HR) and the 95 % confidence interval (CI) to evaluate the association between *CARLO-5* expression and survival. The expression differences between cell lines, the expression changes after transfection, cell cycle, and cell migration assays were analyzed using independent samples t test. All data were presented as mean±standard error from three independent experiments with each measured in triplicate. A two-sided p value of less than 0.05 was considered to be statistically significant.

Results

Expression profile of *CARLo-5* in NSCLC cell lines

To determine whether *CARLo-5* is upregulated in lung cancer, we first determined its expression level in lung cancer cell lines including adenocarcinoma and squamous carcinoma subtypes utilizing qRT-PCR. When normalized to normal bronchial epithelial cell line (16HBE), the expression level of *CARLo-5* was upregulated in SPC-A1 and NCI-H1975 cell lines but downregulated in A549 cell lines (Fig. 1a).

CARLo-5 expression pattern and correlation between *CARLo-5* expression level and clinical characteristics

The expression level of *CARLo-5* in 62 pairs of human NSCLC and adjacent non-cancerous tissues was examined by quantitative real-time PCR. *CARLo-5* was significantly upregulated in cancerous tissues compared with adjacent normal tissues (Fig. 1b, $p<0.0001$), indicating that *CARLo-5* was frequently upregulated in NSCLC. Then, we identified the correlation between *CARLo-5* expression and clinicopathological parameters. As illustrated in Fig. 1b, c, high *CARLo-5* expression was associated with advanced pathological stage ($p<0.0001$) and lymph node metastasis ($p<0.0001$).

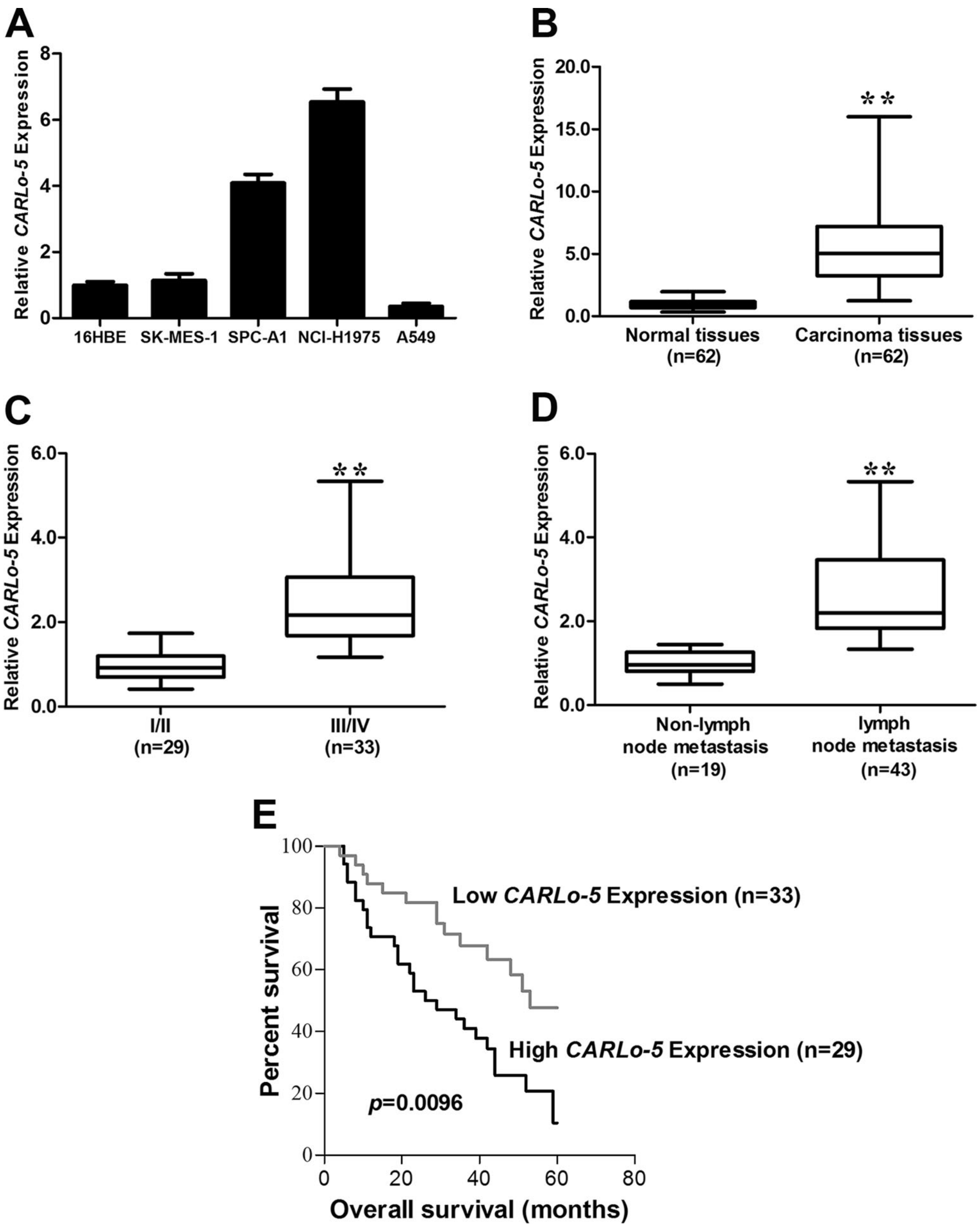

Fig. 1 CARLo-5 expression in NSCLC cell lines, cancer tissues, and its clinical significance. **a** qRT-PCR analysis of *CARLo-5* expression levels in NSCLC cell lines (SK-MES-1, SPC-A1, NCI-H1975, and A549) compared with the normal bronchial epithelial cell line (16HBE). **b** Difference in expression levels of *CARLo-5* expression levels between non-small cell lung cancer tissues and matched non-tumor NSCLC tissues. The expression of *CARLo-5* was normalized to β-actin. The statistical differences between samples were analyzed with paired samples *t* test (n=62, p<0.0001). **c** *CARLo-5* expression was significantly higher in patients at advanced pathological stages (p<0.0001). **d** *CARLo-5* expression was significantly higher in patients with lymph node metastasis than in patients with non-lymph node metastasis (p<0.0001). *Horizontal lines* in the box plots represent the medians, the *boxes* represent the interquartile range, and the *whiskers* represent the 2.5th and 97.5th percentiles. **e** Patients with high levels of *CARLo-5* expression showed reduced overall survival times compared with patients with low levels of *CARLo-5* expression (p=0.0096, log-rank test). p<0.05; **p<0.01

However, *CARLo-5* expression level was not associated with other parameters such as age and gender (Table 1).

Kaplan-Meier survival analysis and log-rank tests using patient postoperative survival were conducted to further evaluate the correlation between *CARLo-5* and prognosis of patients with NSCLC. According to the median ratio of relative *CARLo-5* expression (5.789) in tumor tissues, the 62 NSCLC patients were classified into two groups: high *CARLo-5* group (n=29), *CARLo-5* expression ratio≥median ratio, and low *CARLo-5* group (n=33), *CARLo-5* expression ratio≤median ratio. From the Kaplan-Meier survival curve, we observed that a high expression of *CARLo-5* is a significant predictor of shorter overall survival (p=0.0096, Fig. 1e). These results suggest that *CARLo-5* may play an important part in the progression of NSCLC.

Konckdown of *CARLo-5* inhibits proliferation of NSCLC cells in vitro

As shown in Fig. 1a, *CARLo-5* was most highly expressed in NCI-H1975 cells. To investigate the role of *CARLo-5* in NSCLC progression, we selected H1975 cells as our experimental model. We modulated the expression level of *CARLo-5* through RNA interference experiments. To avoid off-target effects, we designed two siRNAs targeting different regions of *CARLo-5*. qRT-PCR analysis of *CARLo-5* expression levels was performed 48 h after

Table 1 Correlation between CARLo-5 expression and clinicopathologic characteristics

Clinicopathologic characteristics	Number of patients (%)	Relative expression of *CARLo-5*[a]	*p* value[*]
Gender			
Male	37 (59.67 %)	6.23	0.656
Female	25 (40.32 %)	5.51	
Smoking			
Never	23 (37.09 %)	5.96	0.234
Ever	39 (62.90 %)	5.45	
Site of tumor			
Left lung	28 (45.16 %)	5.29	0.856
Right lung	34 (54.84 %)	5.97	
Histological grade			
Well and moderately	36 (58.06 %)	4.89	0.078
Poorly and others	26 (41.94 %)	6.33	
Lymph node metastasis			
Negative	43 (69.35 %)	2.51	<0.0001
Positive	19 (30.64 %)	6.59	
TNM stage			
I–II	29 (46.77 %)	2.94	<0.0001
III–IV	33 (53.23 %)	7.51	

[a] Median of relative expression

[*] $p<0.05$ was considered significant (Mann–Whitney *U* test)

transfection. As shown in Fig. 2a, *CARLo-5* was significantly knocked down by siCARLo-2, the most effective siRNA. Thus, siCARLo-2 was used in subsequent experiments.

Kim et al. [11] demonstrated that *CARLo-5* has a role in cell cycle regulation of colon cancer. We then explored the effects of *CARLo-5* on biological behaviors of NSCLC. Cell-counting kit-8 assays indicated that cell proliferation was reduced in H1975 cells when *CARLo-5* expression was knocked down (Fig. 2b). Consistent with reduced proliferation, H1975 cells, in which *CARLo-5* expression was attenuated, had markedly lower levels of proliferating cell nuclear antigen (PCNA) expression compared to control cells (Fig. 2c). These results suggest that *CARLo-5* may play a physiological role in regulation of cell proliferation, which are consistent with previous study [11]. Next, we examined whether the cell cycle would be affected by inhibition of *CARLo-5*. Knockdown of *CARLo-5* induced a significant increase in the percentage of cells in G0/G1 phase as determined by flow cytometric assay (Fig. 2d). These data suggest that knockdown of *CARLo-5* may inhibit the proliferation of H1975 cells through promoting G0/G1 arrest. To explore the underlying mechanism of *CARLo-5*'s role in G0/G1 arrest, we investigated the G0/G1 arrest markers, p16, p21, and p27, by Western blot. The results showed that p16, p21, and p27 proteins were increased with the knockdown of *CARLo-5* (Fig. 2e).

The effects of *CARLo-5* on the invasion of NSCLC cells

To investigate whether *CARLo-5* had a functional role in facilitating NSCLC cell migration and invasion, we performed migration and invasion assay. A significant decrease in cancer cell migration and invasion was observed in H1975 cells when *CARLo-5* expression was knocked down (Fig. 3a, b).

The effects of *CARLo-5* on epithelial–mesenchymal transition

The epithelial–mesenchymal transition (EMT) is a well-coordinated process during embryonic development and a pathological feature in tumorigenesis [12, 13]. During such a process, epithelial phenotype cells lose the expression of E-cadherin and other components of cell junctions and adopt a mesenchymal phenotype [14]. A growing number of studies suggest that the EMT contributes to cancer invasion, metastasis, and therapeutic resistance [15]. The EMT process is mediated by a set of transcription factors including the zinc-finger proteins Snail and Slug (also known as Snai2), the zinc-finger/homeodomain protein ZEB1, and the bHLH factor Twist [16].

We then explored whether direct inhibition of *CARLo-5* could reverse EMT in NSCLC cell lines. H1975 cells were transfected with siCARLo-2. As anticipated, the resulting cells acquired a more rounded, epithelial morphology (Fig. 4a). We then employed immunofluorescence to measure the expression of multiple EMT-associated genes. As illustrated in Fig. 4a, relative to cells transfected with control siRNA, H1975 cells transfected with siCARLo-2 exhibited a significant increase in the E-cadherin protein level and a significantly decreased expression of fibronectin and vimentin (Fig. 4a). Moreover, Western blot analyses revealed that knockdown of *CARLo-5* decreased the expression of some mesenchymal marker including vimentin and fibronectin while it upregulated the expression of epithelial marker E-cadherin (Fig. 4b). A series of transcription factors (TFs) are involved in EMT processes; we assessed the effect of *CARLo-5* silencing on the expression of following TFs known to promote EMT: Snail, Slug, ZEB1, and Twist. We found that H1975 cells expressed lower levels of Snail and Twist in response to *CARLo-5* silencing. In addition, while the expression of Snail decreased significantly, the expression of ZEB1 and Slug remained almost unchanged after *CARLo-5* knockdown (Fig. 4c).

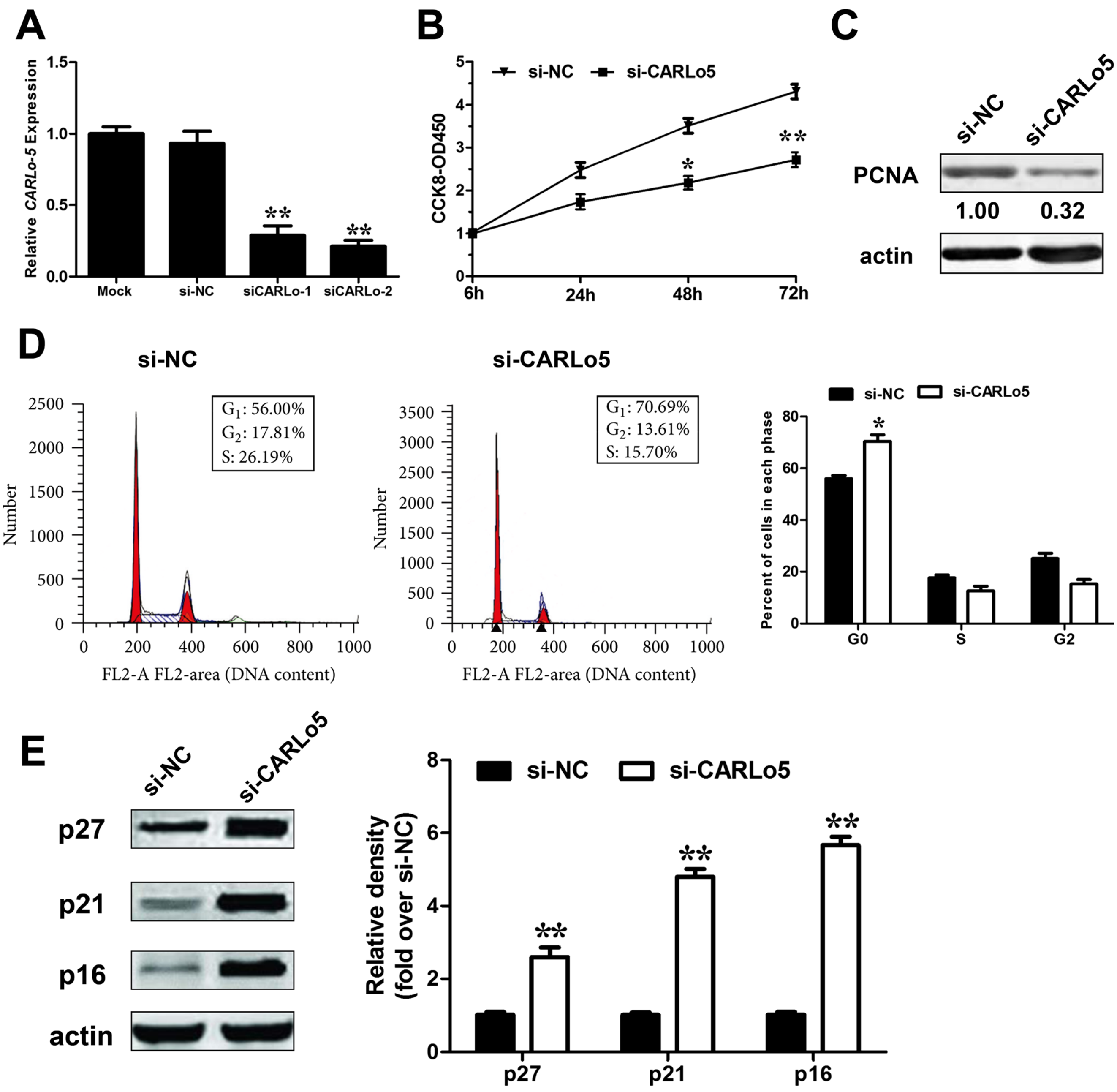

Fig. 2 Effect of *CARLo-5* on cell proliferation. **a** qRT-PCR analysis of *CARLo-5* expression following treatment of H1975 cells with two individual siRNAs targeting *CARLo-5*. **b** H1975 cells were transfected with si-*CARLo-2* or si-NC. CCK8 assays were performed to determine the proliferation of H1975 cells. Data represent the mean±S.D. from three independent experiments. **c** Changes in the proliferation marker, PCNA, were shown by Western blotting analysis and normalized to β-actin after si-*CARLo5* transfection. **d** Cell cycle analysis determined the relative cell numbers in each cell cycle phase after propidium iodide staining of *CARLo-5*-downregulated H1975 cells. *Numbers inside bars* represent percentages of cells in each phase. **e** H1975cells, treated as described in Fig. 2c, d, were collected for Western blotting analysis of the G0/G1 arrest markers, p16, p21, and p27. Relative protein expression was identified (n=3). Data represent the mean±S.D. from three independent experiments. $p<0.05$; $^{**}p<0.01$

Discussion

NSCLC is among the leading causes of cancer-related death. Our understanding of NSCLC pathogenesis has improved through the identification of activating mutations in and amplifications of oncogenes, including KRAS [17], EGFR [18], KARS [19], and inactivating mutations in tumor-suppressive genes, such as p53 [20]. However, the mechanism of NSCLC progression, including the role of cell proliferation, invasion, metastasis, and apoptosis resistance, has not been clarified. Accumulating evidence has highlighted the importance of lncRNAs in cancer [6–10]. Although literature about lncRNA

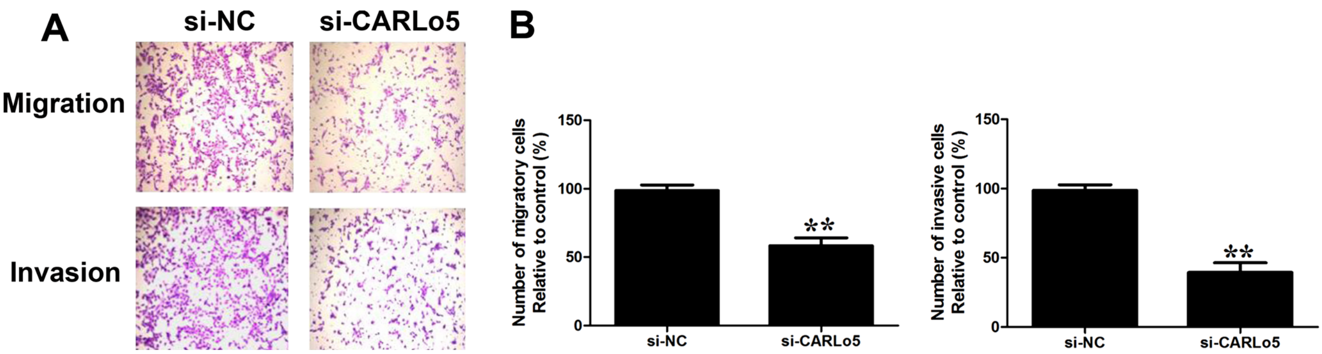

Fig. 3 Effect of *CARLo-5* on cell migration and invasion. **a**, **b** H1975 cells were transfected with *CARLo-5* siRNA or si-NC. Transwell assays were performed to investigate the migratory and invasive ability of NSCLC cells. Data represent the mean±S.D. from three independent experiments. $p<0.05$; $^{**}p<0.01$

is increasing, only a small proportion of lncRNAs have been characterized. Our study provides evidence that *CARLo-5*, a lncRNA, is clinically and functionally relevant to the progression of NSCLC.

As a new molecule in the lncRNAs world, *CARLo-5* was initially well-noted for its upregulation in colon cancer, wherein *CARLo-5* promotes tumor growth [11]. Inspired by the observation that *CARLo-5* is upregulated during tumor progression, we investigated the biological role of *CARLo-5* in NSCLC progression and analyzed its clinical significance.

In this study, we found that the expression of *CARLo-5* was dramatically upregulated in NSCLC tissues compared with

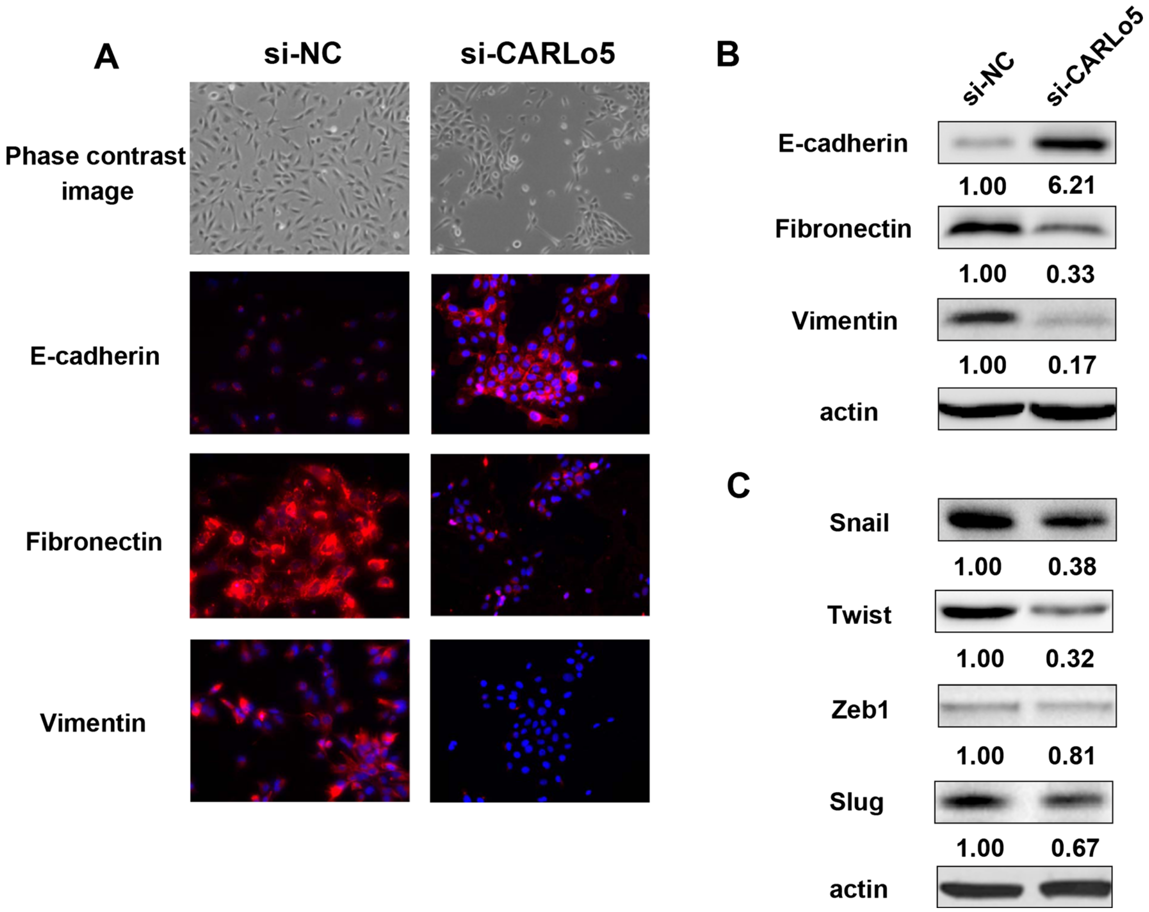

Fig. 4 Effect of *CARLo-5* on the EMT progress. **a** Phase-contrast images (*up*) and immunofluorescence images (*down*) of H1975 cells stained using antibodies against E-cadherin, fibronectin, or vimentin after transfected with si-*CARLo-2*. **b** Western blot analysis of phenotypic markers after *CARLo-5* knockdown in H1975 cells. **c** Western blot analysis of EMT-related TFs after *CARLo-5* knockdown in H1975 cells. Relative protein expression was identified ($n=3$) and normalized to β-actin. Data represent the mean±S.D.

adjacent normal tissues. Furthermore, high expression of *CARLo-5* was correlated with a less differentiated histology, metastasis, and clinical stage. These findings indicate that *CARLo-5* play a role in the modulation of cancer progression.

We demonstrated that inhibition of *CARLo-5* led to a significant reduction of cell proliferation and invasion. To investigate the mechanism through which *CARLo-5* contributes to the enhanced proliferation of NSCLC, we examined the effects of *CARLo-5* on cell cycle. We found that *CARLo-5* silencing induced a significant G0/G1 arrest. Furthermore, the Western blot analysis indicated that knockdown of *CARLo-5* significantly increased the expression of G0/G1 arrest markers, p16, p21, and p27. P16, p21, and p27 were used as the marker of G0/G1 arrest in cell research, and a reduction in the protein levels of these markers confirmed the flow cytometric results that knockdown of *CARLo-5* induced the G0/G1 arrest.

To explore the mechanism through which *CARLo-5* contributes to the migration and invasion of NSCLC, we investigated the effects of *CARLo-5* on EMT. Over the past decades, cell and tumor biologists have identified the vital role of EMT in cancer metastasis and invasion, a process where epithelial cells lose polarity and acquire a mesenchymal phenotype [12, 13]. Key hallmarks of EMT include the loss of E-cadherin expression and enhanced expression of fibronectin and vimentin [14]. Previous studies have identified that EMT can be triggered by various external signals such as hepatocyte growth factor, transforming growth factor, and epidermal growth factor and are mediated by a set of transcription factors including Snail, Slug, ZEB1, and Twist [16]. Recent studies have highlighted the importance of long non-coding RNAs in the regulation of EMT by controlling EMT inducers. For example, HOATIR promoted EMT by regulating Slug, Snail, and Twist expression [21]. MALAT-1 promoted EMT by activating Wnt signaling in vitro [22]. Similar regulation has been reported of BRAF-activated non-coding RNA [23]. Therefore, we determined the expression level of hallmarks of EMT following *CARLo-5* inhibition. Knockdown of *CARLo-5* induced a marked reduction of fibronectin and vimentin expression while upregulated E-cadherin expression, restoring the H1975 cells to more of an epithelial phenotype. What is more, we demonstrated that knockdown of *CARLo-5* downregulated the expression of a series of transcription factors that mediate the EMT. Our results indicated that the inhibitory effects on cell invasion of *CARLo-5* silencing were associated with EMT.

In summary, we demonstrate that the expression of *CARLo-5* was significantly upregulated in NSCLC tissues. We also showed that *CARLo-5* promoted the proliferation and invasion of NSCLC cells, suggesting that *CARLo-5* may play a functional role in NSCLC development. Our study may add our understanding to the molecular mechanisms through which *CARLo-5* contributes to the tumor progression, which may facilitate the development of lncRNA-directed diagnostics and therapeutics against cancers.

Acknowledgments This work was supported by Shanghai Municipal Natural Science Foundation (grant no. 10ZR1424900). The funding sources had no role in the study design, in the collection, analysis, and interpretation of data; in the writing of the manuscript; and in the decision to submit the manuscript for publication.

Conflicts of interest None

References

1. Siegel R, Naishadham D, Jemal A. CA Cancer J Clin. 2012;62(1):10–29.
2. Smith RA, Manassaram-Baptiste D, Brooks D, Cokkinides V, Doroshenk M, Saslow D, et al. Cancer screening in the United States, 2014: a review of current American Cancer Society guidelines and current issues in cancer screening. CA Cancer J Clin. 2014;64(1):30–51.
3. Verdecchia A, Francisci S, Brenner H, Gatta G, Micheli A, Mangone L, et al. EUROCARE-4 Working Group: recent cancer survival in Europe: a 2000-02 period analysis of EUROCARE-4 data. Lancet Oncol. 2007;8(9):784–96.
4. Kugel JF, Goodrich JA. Non-coding RNAs: key regulators of mammalian transcription. Trends Biochem Sci. 2012;37:144–51.
5. Fachel AA, Tahira AC, Vilella-Arias SA, Maracaja-Coutinho V, Gimba ER, Vignal GM, et al. Expression analysis and in silico characterization of intronic long noncoding RNAs in renal cell carcinoma: emerging functional associations. Mol Cancer. 2013;12:140.
6. Yang F, Huo XS, Yuan SX, Zhang L, Zhou WP, Wang F, et al. Repression of the long noncoding RNA-LET by histone deacetylase 3 contributes to hypoxia-mediated metastasis. Mol Cell. 2013;49:1083–96.
7. Huang JF, Guo YJ, Zhao CX, Yuan SX, Wang Y, Tang GN, et al. Hepatitis B virus X protein (HBx)-related long noncoding RNA (lncRNA) down-regulated expression by HBx (Dreh) inhibits hepatocellular carcinoma metastasis by targeting the intermediate filament protein vimentin. Hepatology. 2013;57:1882–92.
8. Gupta RA, Shah N, Wang KC, Kim J, Horlings HM, Wong DJ, et al. Long non-coding RNA HOTAIR reprograms chromatin state to promote cancer metastasis. Nature. 2010;464:1071–6.
9. Prensner JR, Chen W, Iyer MK, Cao Q, Ma T, Han S, et al. PCAT-1, a long noncoding RNA, regulates BRCA2 and controls homologous recombination in cancer. Cancer Res. 2014;74(6):1651–60.
10. Yang F, Xue X, Zheng L, Bi J, Zhou Y, Zhi K, et al. Long non-coding RNA GHET1 promotes gastric carcinoma cell proliferation by increasing c-Myc mRNA stability. FEBS J. 2014;281(3):802–13.
11. Kim T, Cui R, Jeon YJ, Lee JH, Lee JH, Sim H, et al. Long-range interaction and correlation between MYC enhancer and oncogenic long noncoding RNA CARLo-5. Proc Natl Acad Sci U S A. 2014;111(11):4173–8.
12. Tam WL, Weinberg RA. The epigenetics of epithelial-mesenchymal plasticity in cancer. Nat Med. 2013;19(11):1438–49.
13. Nieto MA. Epithelial plasticity: a common theme in embryonic and cancer cells. Science. 2013;342(6159):1234850.
14. Pinto CA, Widodo E, Waltham M, Thompson EW. Breast cancer stem cells and epithelial mesenchymal plasticity—implications for chemoresistance. Cancer Lett. 2013;341(1):56–62.

15. Rhim AD. Epithelial to mesenchymal transition and the generation of stem-like cells in pancreatic cancer. Pancreatology. 2013;13(2):114–7.
16. Ouyang G, Wang Z, Fang X, Liu J, Yang CJ. Molecular signaling of the epithelial to mesenchymal transition in generating and maintaining cancer stem cells. Cell Mol Life Sci. 2010;67(15):2605–18.
17. Kim M, Chen X, Chin LJ, Paranjape T, Speed WC, Kidd KK, et al. Extensive sequence variation in the 3′ untranslated region of the KRAS gene in lung and ovarian cancer cases. Cell Cycle. 2014;3(6):1030–40.
18. Antonicelli A, Cafarotti S, Indini A, Galli A, Russo A, Cesario A, et al. EGFR-targeted therapy for non-small cell lung cancer: focus on EGFR oncogenic mutation. Int J Med Sci. 2013;10(3):320–30.
19. Wang L, Hu H, Pan Y, Wang R, Li Y, Shen L, et al. PIK3CA mutations frequently coexist with EGFR/KRAS mutations in non-small cell lung cancer and suggest poor prognosis in EGFR/KRAS wildtype subgroup. PLoS One. 2014;9(2):e88291.
20. Yamaguchi F, Kugawa S, Tateno H, Kokubu F, Fukuchi K. Analysis of EGFR, KRAS and P53 mutations in lung cancer using cells in the curette lavage fluid obtained by bronchoscopy. Lung Cancer. 2012;78(3):201–6.
21. Xu ZY, Yu QM, Du YA, Yang LT, Dong RZ, Huang L, et al. Knockdown of long non-coding RNA HOTAIR suppresses tumor invasion and reverses epithelial-mesenchymal transition in gastric cancer. Int J Biol Sci. 2013;9(6):587–97.
22. Ying L, Chen Q, Wang Y, Zhou Z, Huang Y, Qiu F. Upregulated MALAT-1 contributes to bladder cancer cell migration by inducing epithelial-to-mesenchymal transition. Mol BioSyst. 2012;8(9):2289–94.
23. Sun M, Liu XH, Wang KM, Nie FQ, Kong R, Yang JS, et al. Downregulation of BRAF activated non-coding RNA is associated with poor prognosis for non-small cell lung cancer and promotes metastasis by affecting epithelial-mesenchymal transition. Mol Cancer. 2014;13:68.
