# Supplementary material for: Expression of Concern: Prognostic value of long non-coding RNA CCAT1 expression in patients with cancer: A meta-analysis
Source: PLoS One. 2023 Apr 20;18(4):e0284940. doi: 10.1371/journal.pone.0284940 (PMC10118116; doi:10.1371/journal.pone.0284940)
Supplement: S1 File — (ZIP) [file pone.0284940.s001.zip › 3 The 11 included studies in PDF/11.pdf]

Clin Exp Med
DOI 10.1007/s10238-015-0395-9

ORIGINAL ARTICLE

Long non-coding RNA CARLo-5 expression is associated with disease progression and predicts outcome in hepatocellular carcinoma patients

Fuqiang Wang[1] · Chengrong Xie[2] · Wenxing Zhao[2] · Zhigang Deng[3] · Huili Yang[4] · Qinliang Fang[1]

Received: 17 June 2015 / Accepted: 14 September 2015
© Springer International Publishing Switzerland 2015

Abstract Recently, many studies show that long non-coding RNAs (lncRNAs) play important roles in cancer biology. Although its expression was reported dysregulated during tumorigenesis, the contributions of lncRNAs to hepatocellular carcinoma (HCC) are still largely unknown. In particular, the lncRNA CARLo-5 has a functional role in cell-cycle regulation in colon cancer, while the clinical significance and biological function of CARLo-5 in HCC remain unelucidated. In order to fill those study blanks, the expression level of CARLo-5 in human HCC specimens was tested, and its correlation with clinicopathologic features as well as the prognosis for patients with HCC was analyzed. Additionally, MTT, wound healing and transwell assays were employed to investigate the biological function of CARLo-5. The results showed that CARLo-5 levels were significantly overexpressed in HCC tissues compared to ANLT. Besides, high expression of CARLo-5 was associated with liver cirrhosis ($P = 0.001$), tumor number ($P < 0.001$), vascular invasion ($P = 0.001$), capsular formation ($P = 0.014$) and Edmondson–Steiner grade ($P < 0.001$), which proved that CARLo-5 was an independent risk factor for overall survival and disease-free survival. In addition, in highly metastatic HCC cell lines (HCCLM3 and MHCC97-L), CARLo-5 was up-regulated, but in lowly metastatic HCC cell lines (HepG2, SNU387), it showed down-regulated. Besides, by using gain and loss of function experiments in HCC cell lines (HCCLM3 and HepG2), the results showed that CARLo-5 overexpression significantly enhanced cell proliferation, migration and invasion in vitro. Our study also revealed that CARLo-5 was prominently up-regulated in HCC specimens and its high expression was associated with poor prognosis of HCC patients. Totally, those findings together indicate that CARLo-5 promotes proliferation and metastasis of HCC and potentially emerged as a novel therapeutic target.

Keywords CARLo-5 · Hepatocellular carcinoma · lncRNA · Metastasis · Prognosis

Electronic supplementary material The online version of this article (doi:10.1007/s10238-015-0395-9) contains supplementary material, which is available to authorized users.

✉ Huili Yang
lili_yeung@sina.com

✉ Qinliang Fang
qinliang_fang@163.com

1 Department of Hepatobiliary Surgery, Zhongshan Hospital of Xiamen University, Fujian Provincial Key Laboratory of Chronic Liver Disease and Hepatocellular Carcinoma, No. 201-209 Hubin South Road, Xiamen 361000, Fujian, China

2 Medical College of Xiamen University, Fujian Provincial Key Laboratory of Chronic Liver Disease and Hepatocellular Carcinoma, Xiamen 361000, Fujian, China

3 Department of General Surgery, MianYang Central Hospital, Mianyanng 621000, Sichuan, China

4 Department of Neurology, Zhongshan Hospital of Xiamen University, Fujian Provincial Key Laboratory of Chronic Liver Disease and Hepatocellular Carcinoma, Xiamen 361000, Fujian, China

Introduction

Hepatocellular carcinoma (HCC) is the fifth most common tumor and the third most common cause of cancer mortality in the world [1]. Over the past decades, there has been an explosion in understanding of the molecular alterations occurring in HCC, and a series of molecular biomarkers that have potential prognostic and therapeutic significance have been identified [2, 3]. Although significant advance has

Published online: 03 October 2015

been made for treatment of HCC, knowledge concerning the long-term prognosis, however, still remains little because of postoperational recurrence and metastasis. Since the biology of HCC remains poorly understood [3, 4], dissecting the mechanisms underlying recurrence and metastasis of HCC is of great value for developing effective therapeutic and prognostic strategies.

Long non-coding RNAs (lncRNAs), non-protein-coding transcripts longer than 200 bp, have recently attracted interesting due to their functions in a variety of biological process [5, 6], and the alternation of lncRNA can result in abnormal expression of gene products, even lead to the occurrence of tumor [7]. Since the lncRNAs HULC was reported to be specifically up-regulated in HCC [8], more and more lncRNAs have been found to play critical role in HCC, such as MALAT1 [9], PVT1 [10], HOTTIP [11] and PCNA-AS1 [12]. However, these lncRNAs lack the capability to fully explain the mechanism of the recurrence and metastasis of HCC, and the overall pathophysiological function of lncRNAs on HCC remains largely unknown.

CARLo-5, known as a long non-coding RNA, which identified to be significantly correlated with the rs6983267 allele, was associated with increased cancer susceptibility [13]. It was also demonstrated that CARLo-5 had a functional role in cell-cycle regulation and tumor development [13]. However, its role in HCC still has not basic research yet. In this study, we detected the expression levels of CARLo-5 in HCC specimens and investigated its function in HCC progression according to in vitro assays. Our study highlights the significance of CARLo-5 in predicting patients' clinical outcome.

Materials and methods

Patient specimens

All patients were recruited between 2007 and 2012 in Zhongshan Hospital of Xiamen University (Xiamen, China). Matched HCC and the adjacent non-tumor liver tissue (ANLT) specimens were obtained from 97 HCC patients. All of the HCC patients with viral hepatitis had HBV infection. Hepatitis C infection and alcohol use were ruled out in these patients. Non-neoplastic liver tissues were obtained from 38 patients with hemangioma or viral hepatitis B. Prior informed consent was obtained from all patients, and the study was approved by the Ethics Committee of Zhongshan Hospital of Xiamen University. All the samples were snap-frozen in liquid nitrogen and then stored at −80 °C for quantitative real-time RT-PCR (qRT-PCR). Histopathology was verified by at least two pathologists in the Department of Pathology at Zhongshan Hospital, and no anticancer treatments were given before biopsy collection.

Cell lines

Liver cancer cell lines HepG2, Hep3B, SK-HEP1, SMMC7721, MHCC97-L, MHCC97-H, PLC/PRF/5 and HCCLM3 were purchased from Shanghai Institute of Cell Biology, Liver Cancer Institute of Fudan University. SNU387, SNU449 and the immortalized human normal liver cells THLE-2 and THLE-3 were obtained from American Type Culture Collection (ATCC). HuH-6 and HuH-7 were derived from RIKEN Cell Bank (Ibaraki, Japan), and HLE from the Health Science Research Resources Bank (Osaka, Japan). Above cell lines were routinely maintained in the high-glucose DMEM supplemented with 10 % fetal bovine serum, 100 U/ml penicillin and 100 mg/ml streptomycin at 37 °C in a humidified incubator under 5 % CO_2.

RNA isolation and quantitative real-time PCR

Total RNAs from HCC tissues and cells were extracted using TRIzol reagent (Invitrogen, Carlsbad, CA) according to the instructions. cDNA was synthesized using the universal cDNA synthesis kit (TaKaRa Bio, Shiga, Japan). Real-time PCR was performed using the SYBR Green Real-time PCR Master Mix (TaKaRa Bio, Shiga, Japan) as described. GAPDH was used as an internal control for mRNA. The primers of CARLo-5 and GAPDH were all bought from Invitrogen. The primer sequences tested in this study were listed as follows: 5′-CAACCCTGACGCTCT TTCTG-3′ (sense) and 5′-GCTTTGGATTTGAGACACTC TG-3′ (antisense) for CARLo-5; 5′-AACGGATTTGGTCG TATTGG-3′ (sense) and 5′-TTGATTTTGGAGGGATCT CG-3′ (antisense) for GAPDH.

Follow-up and prognostic study

Patients' follow-up was terminated on June 26, 2014. All 97 HCC patients were regularly followed up by the same experienced team, with surveillance for the recurrence and metastasis by clinical examination, serial monitoring of alpha-fetoprotein levels and ultrasonography in a 3-month interval. For patients who suspects recurrence and metastasis, computed tomography (CT) scan and/or magnetic resonance imaging (MRI) was used to validate recurrent lesions. Twelve conventional variables together with CARLo-5 expression were tested in all 97 patients. The related clinicopathologic data are listed in Table 1 in detail.

Immunohistochemistry (IHC)

The paraffin-embedded tissue samples from postoperative patients were cut into 4-μm sections. Then, the samples

Table 1 Correlations between CARLo-5 and clinicopathologic variables of HCC

Clinicopathologic variable	No.	CARLo-5		*P* value
		Low	High	
Gender				
Female	39	19	20	0.079
Male	58	18	40	
Age (years)				
≤60	52	21	31	0.625
>60	45	16	29	
Etiology				
Virus	65	23	42	0.425
No virus	32	14	18	
Liver cirrhosis				
Presence	57	14	43	0.001
Absence	40	23	17	
AFP				
≥400 μg/l	61	26	35	0.123
<400 μg/l	36	11	25	
Tumor number				
Solitary	35	22	13	<0.001
Multiple	62	15	47	
Tumor size (cm)				
≤5	46	22	24	0.062
>5	51	15	36	
Vascular invasion				
Presence	31	6	35	0.001
Absence	66	31	35	
Capsular formation				
Presence	35	19	16	0.014
Absence	62	18	44	
Child–Pugh				
A	70	30	40	0.124
B	27	7	20	
Edmondson–Steiner grade				
Low grade (I and II)	44	28	16	<0.001
High grade (III and IV)	53	9	44	
BCLC stage				
0/A	43	21	22	0.053
B/C	54	16	38	

were deparaffinized in xylene and rehydrated using a series of graded alcohols. After microwave antigen retrieval, the slides were incubated overnight with a rabbit monoclonal antibody Ki67 (1:100) (Santa Cruz, CA, USA) at 4 °C and subsequent secondary antibody followed by DAB. Slides were blocked with 10 % goat serum before incubating with primary antibody. The samples were incubated overnight with a primary antibody and subsequent secondary antibody followed by DAB. The expression levels of Ki67 were according to the percentage of positive cells.

Vector construction and transfection

Knockdown and overexpression lentivirus as well as the relative negative control (NC) lentivirus was purchased from GenePharma (Shanghai, China). The three candidate hairpin sequences were as follows: sequence-1: sense, 5′-CCAUUCCAUUCAUUUCUCUUUCCUA-3′, antisense, 5′-UAGGAAAGAGAAAUGAAUGGAAUGG-3′; sequence-2: sense, 5′-CCAUUCAUUUCUCUUUCCUACCACA-3′, antisense, 5′-UGUGGUAGGAAAGAGAAAUGAAUGG-3′; sequence-3: sense, 5′-UGGAGUCAGACUGCUUGAACUUGAA-3′; antisense, 5′-UUCAAGUUCAAGCAGUCUGACUCCA-3′. CARLo-5 overexpression CDS area address is http://www.ncbi.nlm.nih.gov/nuccore/ NR.108049.1. HCCLM3 and HepG2 cells were transfected with the shRNA or overexpression lentivirus according to the manufacturer's instructions. 1×10^5 cells were infected with 1×10^8 lentivirus in the presence of 1 μl polybrene (GenePharma, Shanghai, China). In the present study, the infection efficiency of lentivirus was over 90 % (Supplementary Figure 1 and Supplementary Figure 2). There was no obviously cell death between CARLo-5 group and vector group after virus infection. Stable transfection lentivirus cell lines were used for subsequent assays.

MTT assay

The proliferation of HCC cells in vitro was measured using the MTT assay. 1×10^4 stably infected cells were seeded into each well of 96-well plates. Six wells of each group were detected every day. Hundred microliter fresh medium containing MTT 0.5 mg/ml was put into each cell and incubated at 37 °C for 4 h; then, the medium was replaced by 100 ml of DMSO and shaken at room temperature for 10 min. The absorbance was measured at 570 nm.

Wound healing and transwell assay in vitro

Wound healing assay was used to assess the ability of cell migration, and appropriate HCC cells were seeded into 55-mm dishes and cultured for about 1 day. When cells nearly reached 100 % confluence, a scratch line was created with a 100-μl pipette tip. And then, the cells were cultured for 24 h, and the rate of closure was assessed through imaged with an inverted microscope TE-2000S (Nikon). For the transwell invasion assay, about 1×10^5 cells were seeded into the upper chamber of the insert with Matrigel-coated membrane (BD Biosciences, Franklin Lakes, NJ). After culture for 24 h, cells and gel in the

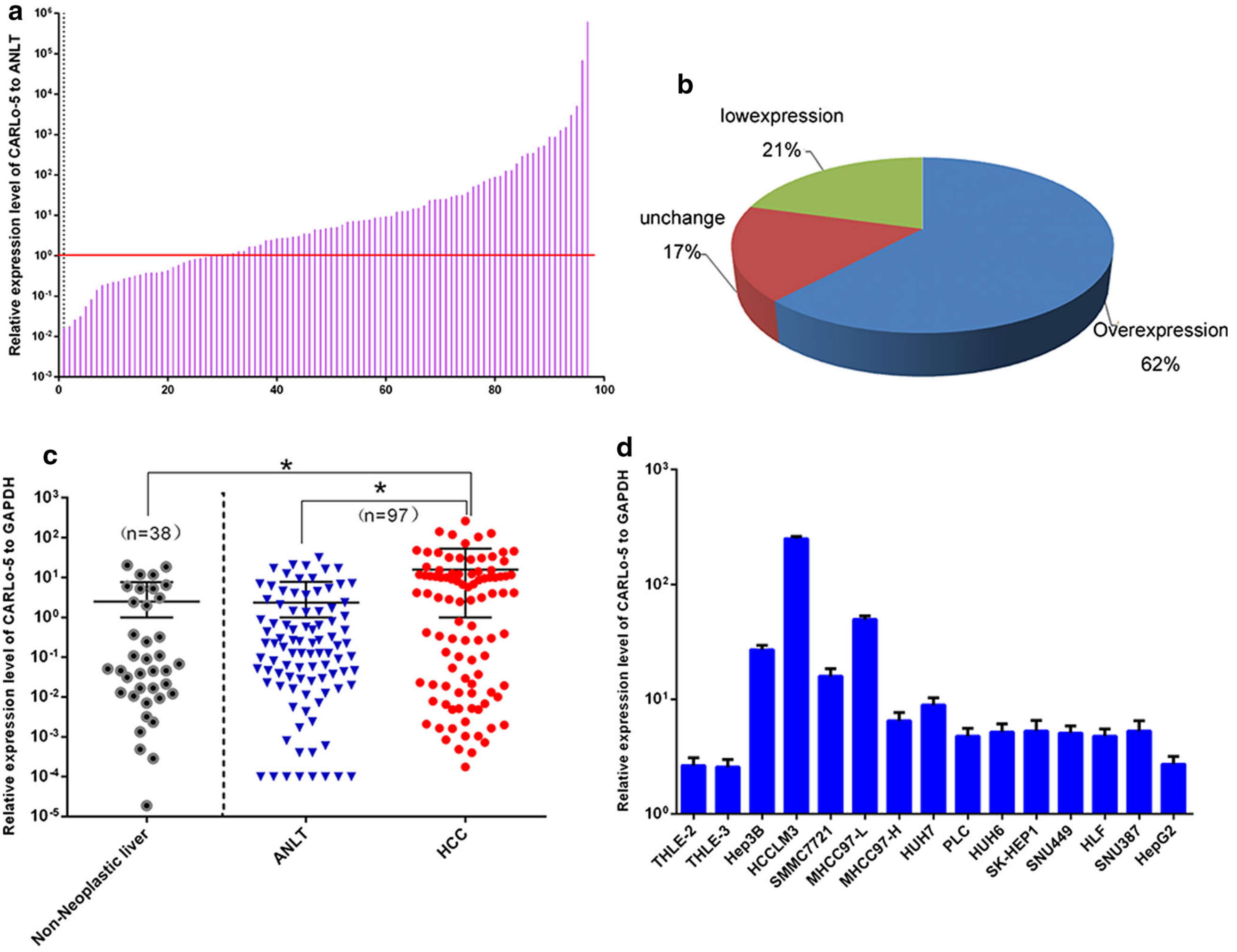

Fig. 1 CARLo-5 was frequently overexpressed in HCC tissues and cells. **a** Expression of lncRNAs CARLo-5 in 97 pairs of HCC tissues and their corresponding adjacent non-tumorous liver tissues (ANLTs). Expression level of CARLo-5 was determined by qRT-PCR and normalized to GAPDH. Fold change was analyzed using the formula $2^{-(\Delta\Delta C_t[\mathrm{HCC/ANLT}])}$. *Red line* indicates fold change of CARLo-5 equal to 2. **b** The distribution of CARLo-5 in 97 cases of HCC and their corresponding ANLTs. 62 % up-regulated in HCC (more than twofold), 17 % unchanged (between 0.5- and 2-fold), 21 % low expression (lower than 0.5-fold). **c** CARLo-5 was also up-regulated in HCC samples compared to non-neoplastic liver specimens. Fold change was calculated using the formula $2^{-\Delta C_t}$. **d** qRT-PCR data reveal a broad spectrum of expression among the 15 tested cell lines. Non-tumoral liver cell lines THLE-2 and THLE-3 display the lowest expression of CARLo-5. HCCLM3 cells had the highest CARLo-5 expression level among the 15 cell lines. Data are presented as mean ± SEM. GAPDH was used as the reference gene. $^*P < 0.05$

upper chamber were removed carefully and cells adhering to underside of the membrane were stained with 0.1 % crystal violet (Bogoo, Shanghai, China) and 20 % methanol. The numbers of cells were counted under an inverted microscope (Nikon). For each experimental group, the assay was performed in triplicate.

Statistical analysis

All data were analyzed by SPSS 21.0 software. Data were presented as mean ± SEM from at least three independent experiments. The differences between two groups were analyzed by Student's *t* test, and the Pearson χ^2 was used for analysis of variables data. Overall survival and disease-free survival curves were obtained using the Kaplan–Meier method, and differences in survival between the high CARLo-5 expression group and low expression group were evaluated using the log-rank test. Univariate analysis and multivariate analysis were analyzed with Cox proportional hazards regression model to verify the independent risk factors. The relationship between the CARLo-5 and Ki67 expression levels was evaluated using the Spearman rank correlation test. *P* value <0.05 was considered statistically significant.

Table 2 Univariable analysis and multivariable analysis of overall survival (OS) and CARLo-5 by Cox proportional hazards regression model

Variables	No.	Univariable analysis		Multivariable analysis	
		HR (95 % CI)	*P* value	HR (95 % CI)	*P* value
Gender					
Female	39	1			
Male	58	0.715 (0.250–1.645)	0.341		NA
Age (years)					
≤60	52	1			
>60	45	0.260 (0.142–1.031)	0.071		NA
HBsAg					
Positive	65	1			
Negative	32	0.655 (0.286–1.720)	0.348		NA
AFP					
≥400 μg/l	61	1			
<400 μg/l	36	2.752 (0.966–6.945)	0.417		NA
Tumor number					
Solitary	35	1		1	
Multiple	62	2.323 (1.175–3.232)	0.032	2.417 (0.478–10.743)	0.310
Tumor size (cm)					
≤5	46	1			
>5	51	1.854 (0.694–5.279)	0.403		NA
Vascular invasion					
Absent	31	1		1	
Present	66	3.486 (1.025–6.900)	0.028	3.033 (1. 692–7.541)	0.034
Capsular formation					
Presence	35	1		1	
Absence	62	3.455 (1.530–8.464)	0.008	2.700 (0.890–10.573)	0.079
Liver cirrhosis					
Absent	57	1		1	
Present	40	3.896 (1.531–6.947)	0.031	4.533 (1.204–13.230)	0.028
Child–Pugh					
A	70	1			
B	27	0.955 (0.358–2.680)	0.862		NA
Edmondson–Steiner grade					
Low grade (I and II)	44	1		1	
High grade (III and IV)	53	3.643 (1.537–8.668)	0.010	3.916 (1.235–17.599)	0.020
BCLC stage					
0/A	43	1			
B/C	54	2.847 (0.860–7.061)	0.090		NA
CARLo-5 expression					
Low	37	1		1	
High	60	3.267 (1.620–6.271)	0.014	2.981 (1.316–3.952)	0.011

Results

Expression of CARLo-5 was prominently up-regulated in HCC samples and cell lines

To investigate CARLo-5 expression levels in HCC, we compared its expression between 97 HCC tissues and their corresponding ANLTs by qRT-PCR. The result showed that CARLo-5 was significantly up-regulated (more than two-fold) in 60 HCC cases (62 %), unchanged (between half-fold and twofold) in 17 HCC cases (17 %) and down-regulated (lower than half-fold) in 20 HCC cases (21 %) (Fig. 1a, b). Remarkably, the expression levels of CARLo-5 were also increased in HCC samples compared with that of ANLTs as

Table 3 Univariable analysis and multivariable analysis of recurrence-free disease (RFS) and CARLo-5 by Cox proportional hazards regression model

Variables	No.	Univariable analysis		Multivariable analysis	
		HR (95 % CI)	*P* value	HR (95 % CI)	*P* value
Gender					
Female	39	1			
Male	58	0.938 (0.167–1.481)	0.174		NA
Age (years)					
≤60	52	1			
>60	45	0.671 (0.218–1.332)	0.079		NA
HbsAg					
Positive	65	1			
Negative	32	0.891 (0.279–1.380)	0.217		NA
AFP					
≥400 μg/l	61	1			
<400 μg/l	36	0.995 (0.957–3.915)	0.065		NA
Tumor number					
Solitary	35	1		1	
Multiple	62	4.537 (1.668–9.620)	0.030	2.173 (1.010–6.227)	0.016
Tumor size (cm)					
≤5	46	1			
>5	51	1.613 (0.571–2.768)	0.672		NA
Capsular formation					
Presence	35	1		1	
Absence	62	1.988 (1.003–3.939)	0.049	1.824 (0.784–3.675)	0.107
Liver cirrhosis					
Absent	57	1			
Present	40	2.268 (1.029–4.999)	0.042	1.728 (0.720–4.415)	0.140
Vascular invasion					
Absent	31	1		1	
Present	66	1.995 (1.408–4.312)	0.022	1.877 (1.612–4.210)	0.027
Child–Pugh					
A	70	1			
B	27	0.785 (0.316–3.710)	0.691		NA
Edmondson–Steiner grade					
Low grade (I and II)	44	1			
High grade (III and IV)	53	1.302 (0.809–2.754)	0.514		NA
BCLC stage					
A	43	1			
B/C	54	0.449 (0.281–1.363)	0.501		NA
CARLo-5 expression					
Low	37	1		1	
High	60	2.873 (1.669–5.852)	0.005	1.810 (1.562–5.177)	0.010

well as to non-neoplastic liver disease tissues (Fig. 1c). Furthermore, the CARLo-5 expression in non-tumoral liver cell lines (THLE-2, THLE-3) was examined and a panel of HCC cell lines (HepG2, Hep3B, SK-HEP1, SMMC7721, MHCC97-L, MHCC97-H, PLC/PRF/5, HCCLM3, SNU387, SNU449) by qRT-PCR. The results indicated that CARLo-5 was overexpressed in all liver cancer cell lines but not in THLE-2 and THLE-3 cell lines, consistent with the data derived from patient specimens (Fig. 1d). Finally, HCCLM3 (the highest expression of CARLo-5) and HepG2 (the relatively lower expression of CARLo-5) were chosen as representatives for further research.

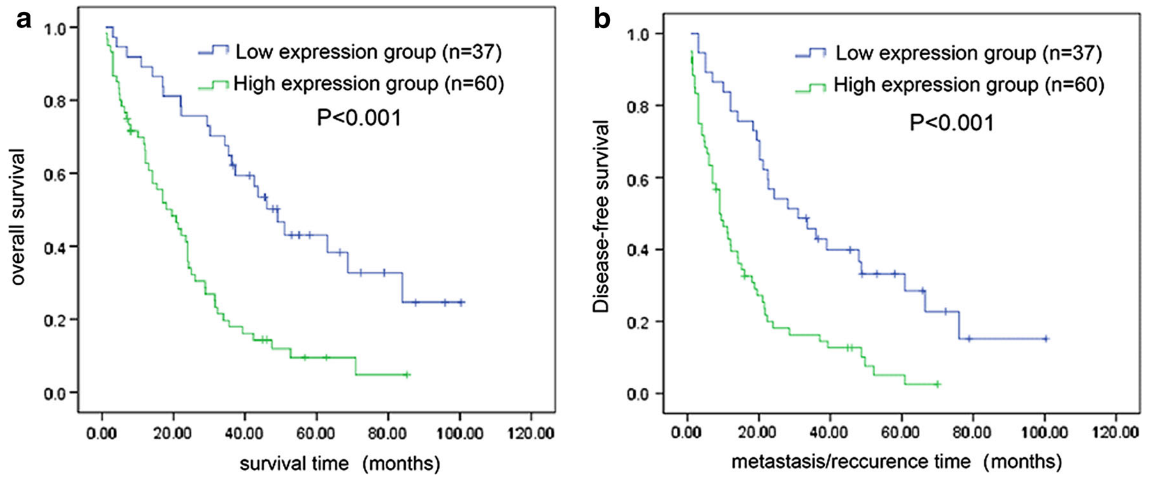

Fig. 2 High CARLo-5 expression was significantly associated with overall survival and disease-free survival. In total, 97 patients were divided into low expression group (fold change of CARLo-5 < 2) and high expression group (fold change of CARLo-5 ≥ 2), respectively, according to the result of qRT-PCR. Survival of 97 HCC patients analyzed using the Kaplan–Maier method. **a** The overall survival time of CARLo-5 high expression group was significantly poorer compared to CARLo-5 low expression group ($P < 0.001$). **b** CARLo-5 high expression group also had poorer disease-free survival than CARLo-5 low expression group ($P < 0.001$)

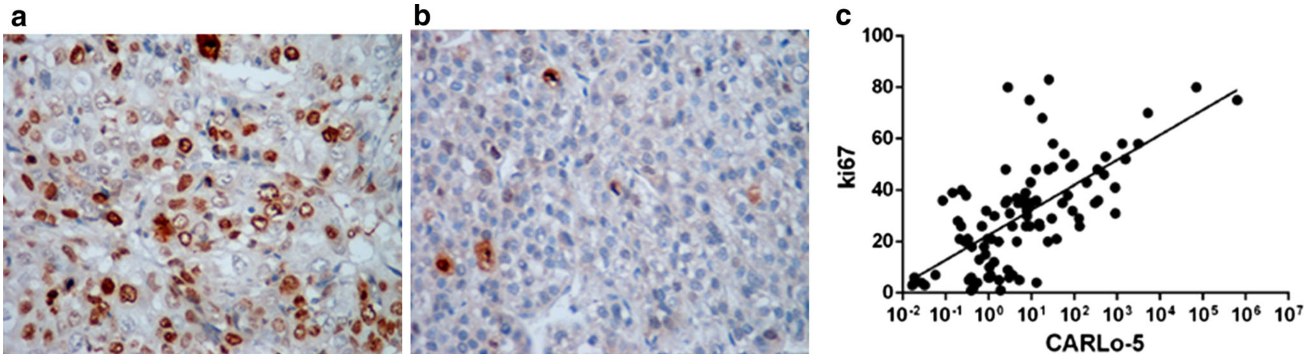

Fig. 3 CARLo-5 expression was positively correlated with Ki67. Immunohistochemistry was used to analyze Ki67 expression in 97 HCC tissues. Paraffin-embedded tissue sections were stained with antibodies against Ki67 and counterstained with hematoxylin. **a** High expression of CARLo-5 and high expression of Ki67. **b** Low expression of CARLo-5 and low expression of Ki67. **c** *Scatterplot* of CARLo-5 expression versus Ki67 expression; the *regression line* indicates a correlation based on the Spearman correlation coefficient ($R = 0.67$, $P < 0.01$)

High CARLo-5 expression is associated with poor clinicopathologic characteristics and prognosis of HCC

To examine the correlation of CARLo-5 expression levels with the clinicopathologic features of HCC, we divided patients (97 cases) into two groups based on the levels of CARLo-5 expression that were either increased (60 cases) or not increased (37 cases). The results showed that CARLo-5 expression was significantly associated with poor clinicopathologic features including liver cirrhosis ($P = 0.001$), tumor number ($P < 0.001$), vascular invasion ($P = 0.001$), capsular formation ($P = 0.014$) and Edmondson–Steiner grade ($P < 0.001$) (Table 1). In addition, we analyzed the relationship between CARLo-5 expression and the patients' prognosis. The vascular invasion [hazard ratios (HR) 3.033, $P = 0.034$], liver cirrhosis (HR 4.533, $P = 0.028$), Edmondson–Steiner grade (HR 3.916, $P = 0.020$) and CARLo-5 expression (HR 2.981, $P = 0.011$) were recognized as independent risk factors for overall survival (OS) by both the univariate analysis and subsequent multivariate survival analysis (Table 2). Furthermore, the tumor number (HR 2.173, $P = 0.016$), vascular invasion (HR 1.877, $P = 0.027$) and CARLo-5 expression (HR 1.810, $P = 0.010$) were verified as independent risk factors for disease-free survival (DFS) by both univariate analysis and multivariate survival analysis (Table 3).

And then, Kaplan–Maier analysis was used to detect the patient OS and DFS rates. The survival curves showed that HCC patients with high levels of CARLo-5 expression had shorter OS and DFS time than those with low levels of

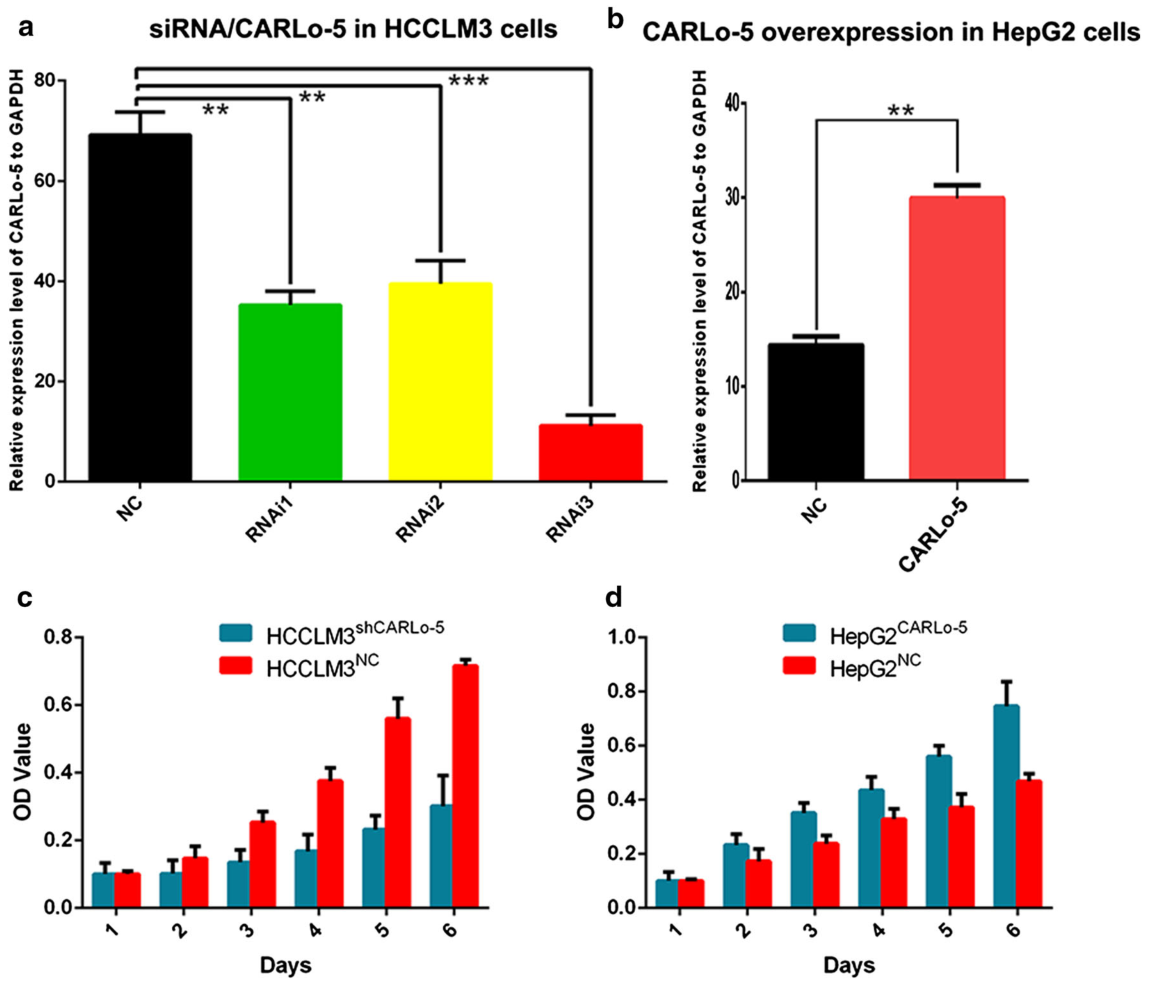

Fig. 4 CARLo-5 promoted proliferation HCC cells in vitro. CARLo-5 expression levels were detected in HCC cells infected with CARLo-5 inhibition lentivirus or CARLo-5 overexpression lentivirus or negative control lentivirus. CARLo-5 expression levels in these cells were evaluated by qRT-PCR. The data were normalized to the expression level of CARLo-5 in NC HCC cells. **a** HCCLM3 cells were treated with three siRNAs targeting CARLo-5 RNA and negative control. The most efficiency of CARLo-5 knockdown was RNAi3. **b** HepG2 cells were transfected with CARLo-5 overexpression lentivirus. Efficiency of CARLo-5 overexpression was more than twofold. GAPDH was used as the reference gene. **c** Cell growth of HCC cells was examined with MTT assays. The results showed that $HCCLM3^{shCARLo-5}$ grew slower than $HCCLM3^{NC}$. **d** $HepG2^{CARLo-5}$ grew faster than $HepG2^{NC}$. $^{**}P < 0.01$; $^{***}P < 0.001$. *NC* negative control

CARLo-5 expression (Fig. 2a, b). Meanwhile, 1-, 3-, 5-year overall survival rate in CARLo-5 high expression group was significantly lower than the CARLo-5 low expression group (68 vs. 89 %; 18 vs. 65 %; 9 vs. 42 %, respectively, $P < 0.001$), and 1-, 3-, 5-year disease-free survival rate in CARLo-5 high expression group was also lower than the low expression group (43 vs. 84 %; 16 vs. 46 %; 5 vs. 32 %, $P < 0.001$).

To determine the role of CARLo-5 in HCC development, we first investigated the expression profile of Ki67 in HCC via immunohistochemical analyses, which indicated that Ki67 expression was low even undetectable in most CARLo-5 low expression HCC tissues and was highly expressed in most CARLo-5 high expression tissues (Fig. 3a, b). Furthermore, very importantly, there was a positive correlation between CARLo-5 and Ki67 expression ($R = 0.67$; Fig. 3c). These findings suggested that CARLo-5 up-regulation might contribute to HCC progression.

CARLo-5 promotes proliferation and migration of HCC cells

Having observed CARLo-5 expression was associated with the tumor number and vascular invasion in HCC patients (Table 1). To further investigate the functionally characterizing CARLo-5 in HCC cell proliferation and migration, shRNA was employed to inhibit the expression of CARLo-5 in HCCLM3 and overexpression CARLo-5 in HepG2. Then, the efficiency of CARLo-5 knockdown or

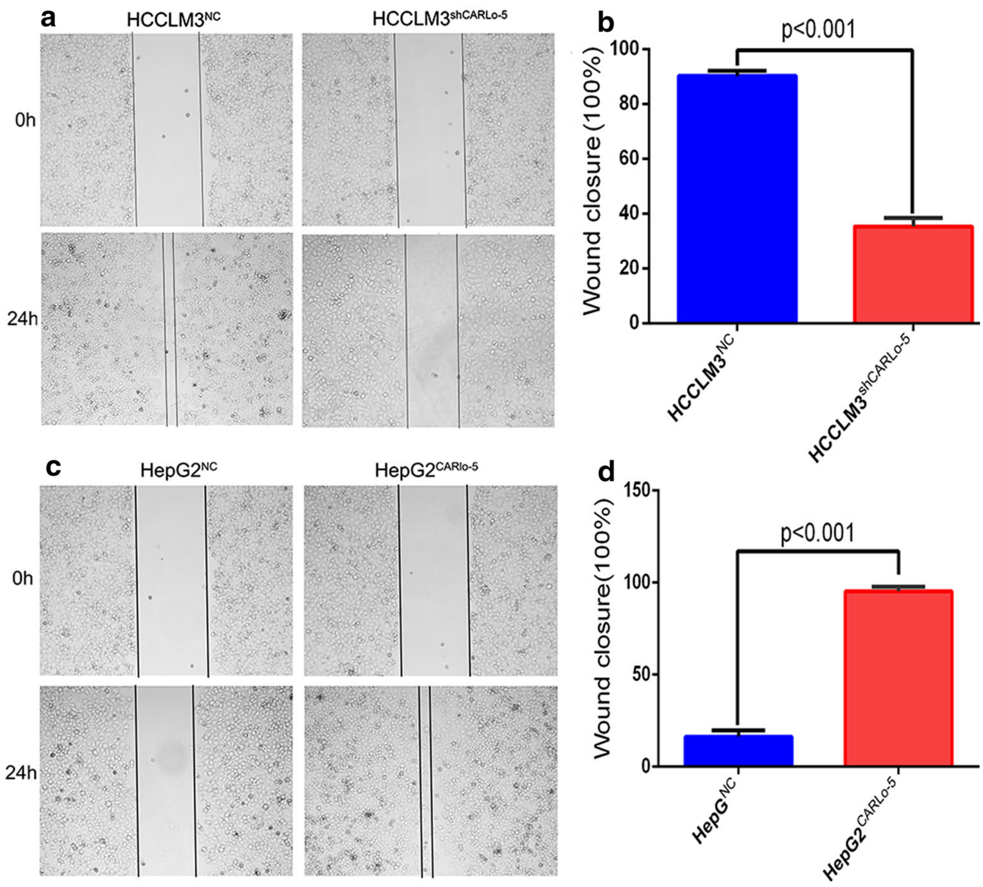

Fig. 5 CARLo-5 significantly promoted migration of HCC cells in vitro. Migration potentials of HCCLM3^{NC}, HCCLM3$^{shCARLo-5}$ and HepG2^{NC}, HepG2$^{CARLo-5}$ cells were measured with wound healing assays. **a**, **b** The results showed that the closure of HCCLM3^{NC} was significantly faster than that of HCCLM3$^{shCARLo-5}$ (96 vs. 38 %, $P < 0.001$). **c**, **d** The wound healing assay indicated that the migration ability of HepG2^{NC} was slower than HepG$^{CARLo-5}$ cells (16 vs. 95 %, $P < 0.001$)

overexpression in HCC cells was assessed by qRT-PCR. The results showed that the sequence-3 could significantly reduce CARLo-5 expression (more than 90 %) (Fig. 4a), and the overexpression efficiency was more than twofold (Fig. 4b). MTT assay was performed to assess the effect of CARLo-5 on cell proliferation. HCCLM3 cells expressing anti-CARLo-5 showed lower proliferation than the control cells (Fig. 4c). In contrast, HepG2 cells expressing CARLo-5 exhibited higher proliferation rate (Fig. 4d).

Furthermore, wound healing and transwell assays were performed to analyze the function of CARLo-5. It was remarkable that inhibition of CARLo-5 expression significantly suppressed wound healing of HCCLM3 cells ($P < 0.001$; Fig. 5a, b). On the contrary, overexpressing CARLo-5 in HepG2, the capacity of healing about HepG2 significantly increased ($P < 0.001$; Fig. 5c, d). Similarly, transwell assays with Matrigel revealed that HCCLM3 expressing anti-CARLo-5 exhibited significantly reduced rate of invasion compared with control cells ($P < 0.001$; Fig. 6a, c, e), while HepG2-overexpressed CARLo-5 migrated faster than vector-transduced control cells ($P < 0.001$; Fig. 6b, d, f). In summary, these results revealed that CARLo-5 enhances HCC cell proliferation and invasion potentialities.

Discussion

More and more evidences have shown that many HCC patients die from recurrence and metastases after curative resection [3, 14, 15]. Thus, it is important to gain a better understanding of the mechanisms that HCC metastasis. In recent years, numerous lncRNAs are found to play critical roles in metastasis and invasion of HCC cells [6, 16]. The lncRNA CARLo-5 gene locates in the 8q24.21 region, which has a function in cell-cycle regulation and tumor progression [13]. However, the clinical significance and biological function of CARLo-5 in HCC remain unclear. In our study, we reported that CARLo-5 was prominently up-regulated in HCC tissues and liver cancer cell lines. We also found that CARLo-5 expression was associated with poor clinicopathologic characteristics, and it was one of the independent risk factors for OS and DFS. Our results showed that CARLo-5 may pave a critical role in the metastasis of HCC.

To get an insight into the functional role of CARLo-5 in HCC, we employed shRNA to knockdown the CARLo-5 expression in HCCLM3 cells and overexpression CARLo-5 in HepG2 cells. The results showed that CARLo-5

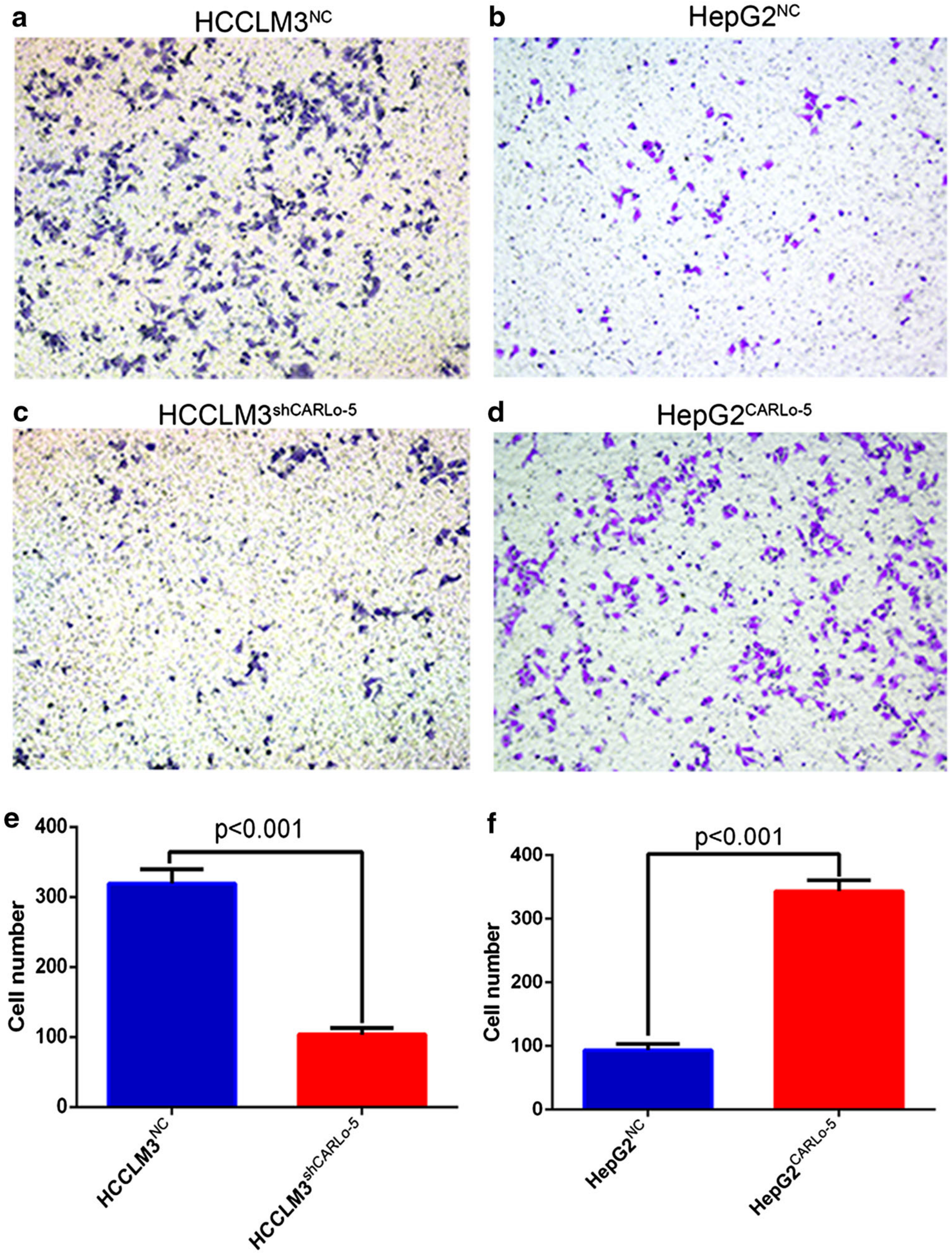

Fig. 6 CARLo-5 significantly promoted invasion of HCC cells in vitro. Invasion potentials of HCCLM3^{NC}, HCCLM3$^{shCARLo\text{-}5}$, HepG2^{NC} and HepG2$^{CARLo\text{-}5}$ cells were measured with transwell assays. **a**, **c**, **e** Our data showed that the numbers of HCCLM3^{NC} cells passed through the Matrigel were much more than that of HCCLM3$^{shCARLo\text{-}5}$ (319 ± 12 vs. 104 ± 6, $P < 0.001$). **b**, **d**, **f** The transwell assay indicated that the invasion ability of HepG2^{NC} was weaker than HepG$^{CARLo\text{-}5}$ cells (94 ± 6 vs. 343 ± 10, $P < 0.001$)

expression resulted in a significant acceleration of cell proliferation and migration in these two HCC cells.

Numerous studies have reported that genetic variants at 8q24 are associated with the risk of many kinds of tumors including hepatocellular carcinoma [17–19]. Interestingly, the lncRNA CARLo-5 is known to be located in the 8q24.21 gene desert [13]. In addition, the MYC enhancer could regulate CARLo-5 expression by enhancing its transcription through direct interaction [13]. Many evidences showed that the genomic region containing MYC enhancer is strongly associated with tumor pathogenesis [20–22]. Therefore, according to the foundations, it is demanded to investigate whether CARLo-5 promotes proliferation and metastasis of HCC. Our results showed that up-regulation of CARLo-5 in HepG2 could promote proliferation and migration. Meanwhile, CARLo-5 depletion dramatically attenuated the proliferation and migration ability in HCCLM3. These results suggested that CARLo-5 could be a potential target of hepatocellular carcinoma.

In conclusion, we demonstrate, for the first time, that the lncRNAs CARLo-5 is prominently up-regulated in HCC specimens and its overexpression is significantly associated with poor prognosis for HCC patients. Furthermore, the results indicate that CARLo-5 plays a functional role in

HCC progression. Totally, the data together indicate that CARLo-5 promotes proliferation and migration of HCC and lncRNA CARLo-5 may be emerged as a novel therapeutic target.

Acknowledgments This work was supported by medical innovation subject of Fujian Province (2009-CXB-58).

Compliance with ethical standards

Conflict of interest There were no financial disclosures from any authors.

References

1. Jemal A, Bray F, Center MM, Ferlay J, Ward E, Forman D. Global cancer statistics. CA Cancer J Clin. 2011;61:69–90.
2. Feng GS. Conflicting roles of molecules in hepatocarcinogenesis: paradigm or paradox. Cancer Cell. 2012;21:150–4.
3. Forner A, Llovet JM, Bruix J. Hepatocellular carcinoma. Lancet. 2012;379:1245–55.
4. El-Serag HB. Hepatocellular carcinoma. N Engl J Med. 2011;365:1118–27.
5. Cheng W, Zhang Z, Wang J. Long noncoding RNAs: new players in prostate cancer. Cancer Lett. 2013;339:8–14.
6. Huang JL, Zheng L, Hu YW, Wang Q. Characteristics of long non-coding RNA and its relation to hepatocellular carcinoma. Carcinogenesis. 2014;35:507–14.
7. Maass PG, Luft FC, Bahring S. Long non-coding RNA in health and disease. J Mol Med (Berl). 2014;92:337–46.
8. Panzitt K, Tschernatsch MM, Guelly C, et al. Characterization of HULC, a novel gene with striking up-regulation in hepatocellular carcinoma, as noncoding RNA. Gastroenterology. 2007;132:330–42.
9. Lai MC, Yang Z, Zhou L, et al. Long non-coding RNA MALAT-1 overexpression predicts tumor recurrence of hepatocellular carcinoma after liver transplantation. Med Oncol. 2012;29:1810–6.
10. Wang F, Yuan JH, Wang SB, et al. Oncofetal long noncoding RNA PVT1 promotes proliferation and stem cell-like property of hepatocellular carcinoma cells by stabilizing NOP2. Hepatology. 2014;60:1278–90.
11. Quagliata L, Matter MS, Piscuoglio S, et al. Long noncoding RNA HOTTIP/HOXA13 expression is associated with disease progression and predicts outcome in hepatocellular carcinoma patients. Hepatology. 2014;59:911–23.
12. Yuan SX, Tao QF, Wang J, et al. Antisense long non-coding RNA PCNA-AS1 promotes tumor growth by regulating proliferating cell nuclear antigen in hepatocellular carcinoma. Cancer Lett. 2014;349:87–94.
13. Kim T, Cui R, Jeon YJ, et al. Long–range interaction and correlation between MYC enhancer and oncogenic long noncoding RNA CARLo-5. Proc Natl Acad Sci USA. 2014;111:4173–8.
14. Farazi PA, DePinho RA. Hepatocellular carcinoma pathogenesis: from genes to environment. Nat Rev Cancer. 2006;6:674–87.
15. Bruix J, Gores GJ, Mazzaferro V. Hepatocellular carcinoma: clinical frontiers and perspectives. Gut. 2014;63:844–55.
16. Yuan JH, Yang F, Wang F, et al. A long noncoding RNA activated by TGF-beta promotes the invasion-metastasis cascade in hepatocellular carcinoma. Cancer Cell. 2014;25:666–81.
17. Malz M, Bovet M, Samarin J, et al. Overexpression of far upstream element (FUSE) binding protein (FBP)-interacting repressor (FIR) supports growth of hepatocellular carcinoma. Hepatology. 2014;60:1241–50.
18. Pedica F, Ruzzenente A, Bagante F, et al. A re-emerging marker for prognosis in hepatocellular carcinoma: the add-value of fishing c-myc gene for early relapse. PLoS ONE. 2013;8:e68203.
19. Ding J, Huang S, Wu S, et al. Gain of miR-151 on chromosome 8q24.3 facilitates tumour cell migration and spreading through downregulating RhoGDIA. Nat Cell Biol. 2010;12:390–9.
20. Pomerantz MM, Ahmadiyeh N, Jia L, et al. The 8q24 cancer risk variant rs6983267 shows long–range interaction with MYC in colorectal cancer. Nat Genet. 2009;41:882–4.
21. Ahmadiyeh N, Pomerantz MM, Grisanzio C, et al. 8q24 prostate, breast, and colon cancer risk loci show tissue-specific long–range interaction with MYC. Proc Natl Acad Sci USA. 2010;107:9742–6.
22. Sur IK, Hallikas O, Vaharautio A, et al. Mice lacking a Myc enhancer that includes human SNP rs6983267 are resistant to intestinal tumors. Science. 2012;338:1360–3.
