# Supplementary material for: Expression of Concern: Prognostic value of long non-coding RNA CCAT1 expression in patients with cancer: A meta-analysis
Source: PLoS One. 2023 Apr 20;18(4):e0284940. doi: 10.1371/journal.pone.0284940 (PMC10118116; doi:10.1371/journal.pone.0284940)
Supplement: S1 File — (ZIP) [file pone.0284940.s001.zip › 3 The 11 included studies in PDF/2.pdf]

RESEARCH ARTICLE

Aberrant Expression of CCAT1 Regulated by c-Myc Predicts the Prognosis of Hepatocellular Carcinoma

Hua-Qiang Zhu[&], Xu Zhou[&], Hong Chang, Hong-Guang Li, Fang-Feng Liu, Chao-Qun Ma, Jun Lu*

Abstract

Background: CCAT1 has been reported to be linked with pathogenesis of malignancies including colon cancer and gastric cancer. However, the regulatory effect of CCAT1 in hepatocellular carcinoma (HCC) remains unclear. The purpose of this research was to identify any role of CCAT1 in the progression of HCC. Materials and Methods: Real time-PCR was performed to test the relative expression of CCAT1 in HCC tissues. A computation screen of CCAT1 promoter was conducted to search for transcription-factor-binding sites. The association of c-Myc with CCAT1 promoter *in vivo* was tested by Pearson correlation analysis and chromatin immunoprecipitation assay. Additionally, Kaplan-Meier analysis and Cox proportional hazards analyses were performed. Results: c-Myc directly binds to the E-box element in the promoter region of CCAT, and when ectopically expressed increases promoter activity and expression of CCAT1. Moreover, Kaplan-Meier analysis demonstrated that the patients with low expression of CCAT1 demonstrated better overall and relapse-free survival compared with the high expression group. Cox proportional hazards analyses showed that CCAT1 expression was an independent prognostic factor for HCC patients. Conclusions: The findings demonstrated CCAT1, acting as a potential biomarker in predicting the prognosis of HCC, is regulated by c-Myc.

Keywords: Long non-coding RNA - biomarker - HCC

Asian Pac J Cancer Prev, **16 (13)**, 5181-5185

Introduction

As one of the most common malignancies in the world, hepatocellular carcinoma (HCC) has a very high morbidity and mortality (Cao et al., 2015). It is a major global health challenge that affects almost 500000 people worldwide every year (Flemming, Yang, Vittinghoff, Kim, and Terrault, 2014). Despite recent improvements in surgery and chemotherapy, the prognosis for HCC remains grim. Therefore, there is a pressing requirement to identify new prognostic biomarkers and therapeutic targets for HCC. Additionally, the underlying pathophysiological mechanisms of HCC development remain unclear.

In recent years, long non-coding RNAs (lncRNAs), which are defined as transcripts of greater than 200 nucleotides with no or little protein coding function, have drawn more and more attention in many research fields (Spizzo et al., 2012; Fang et al., 2014). The long non-coding RNA Colon Cancer Associated Transcript 1 (CCAT1) with abnormal expressions in many malignancies (Alaiyan et al., 2013; Yang et al., 2013; He et al., 2014; Kam et al., 2014; Xiang et al., 2014) have been documented to be involved in the pathogenesis of malignant tumors including colon cancer and gastric cancer. However, the role of CCAT1 in the development of HCC still remains unclear.

In our study, we found the un-normal up-regulation of CCAT1 in the tumor tissues of patients with HCC compared with corresponding adjacent. The HCC cell lines confirmed the aberrant expression of CCAT1 compared the normal liver cell line. In addition, our results validated that CCAT1 acted as a crucial role in the development of HCC. The promoting-cancer effect of CCAT1 was demonstrated *in vitro*. We also found that c-Myc directly binds to the E-box element in the promoter region of CCAT1, and when ectopically expressed increased promoter activity and expression of CCAT1. Nucleotide substitutions in the E-box element in the promoter region abrogated c-Myc-dependent promoter activation. The expression of CCAT1 and c-Myc shows strong association in HCC. Moreover, Kaplan-Meier analyses and Cox proportional regression analysis suggest that CCAT1 may be a potential biomarker for predicting the survival of HCC patients.

Materials and Methods

Clinical samples and cell culture

86 cases of patients who underwent liver cancer

Department of Hepatobiliary Surgery, Provincial Hospital Affiliated to Shandong University, Jinan, Shandong Province, China
*[&]Equal contributors *For correspondence: lujunmdjnyy@163.com*

radical resection during December 2010 to May 2012 were recruited from the Provincial Hospital Affiliated to Shandong University (Jinan, China). Specimens were obtained immediately after surgical resection and stored at -80°C for further analysis. All patients had negative histories of exposure to either chemotherapy or radiotherapy before surgery, and there was no other co-occurrence of diagnosed cancers. This study was approved by the Ethical Committee of Provincial Hospital Affiliated to Shandong University and every patient had written informed consent.

The human HCC cell lines, HepG2 and 97H, were purchased from Liver cancer institute, Fudan University, Shanghai. They were maintained in an atmosphere of 5% CO2 in DMEM medium (Thermo, San Jose, CA, USA) supplemented with 10% fetal bovine serum (Thermo, San Jose, CA, USA). Cell line authentication was performed by STR profiling before initiation of this project.

Real-time PCR

Total RNA from frozen samples and cell lines was extracted by Invitrogen (Invitrogen, California, USA) according to the manufacturer's protocol. cDNAs from all samples were synthesized from 1μg of total RNA by Prime Script RT Master Mix kit (Takara, Japan). The expression of CCAT1 was analyzed by qRT-PCR using Quantifast SYBR Green PCR Kit (Qiagen, Dusseldorf, Germany) at 95°C for 5 min, followed by 40 cycles of 95°C for 10 s and 60°C for 30 s. Primer sequences were as follows: CCAT1 forward primer: 5´ CATTGGGAAAGGTGCCGAGA 3´, reverse primer: 5´ ACGCTTAGCCATACAGAGCC 3´; GAPDH, forward primer: 5´ GGGAGCCAAAAGGGTCAT 3´, and reverse primer: 5´ GAGTCCTTCCACG ATACCAA 3´. Data analyses for the gene expression were performed using the $2^{-\Delta\Delta Ct}$ method.

RNA interference

Short interfering RNA specifically targeting c-Myc and a corresponding scrambled siRNA control (Santa Cruz Biotechnology, Santa Cruz, USA) were transfected into cells in 6-well plates using Lipofectamine 2000 reagent (Invitrogen, Carlsbad, USA) according to the manufacturer's instructions.

Western blotting

Whole cells were washed in PBS and lysed in RIPA lysis buffer supplemented with protease inhibitor cocktail (Roche, Mannheim, Germany). The proteins in each sample were resolved with sodium dodecyl sulfate–polyacrylamide gel electrophoresis, transferred onto polyvinylidene difluoride membranes. The blots were blocked in BSA (5% w/v in PBS + 0.1% Tween 20) for 30min at room temperature. The following primary antibodies were used according to the manufacturer's instructions. Antibodies against c-Myc, and GAPDH were purchased from Cell Signaling Technology (CST, Beverly, MA, USA). The appropriate secondary antibodies (Santa Cruz Biotechnology, Santa Cruz, USA) were used at 1:1,000-1:2,000 (v/v) dilutions in PBS + 0.1% Tween 20 for 1 h at room temperature, and the signals were revealed using ECL kit(Thermo Scientific Pierce, Thermo Fisher Scientific, Rockford, USA).

Chromatin immunoprecipitation

HepG2 and 97H cells were treated with formaldehyde and incubated for 10 min to generate DNA-protein cross-links. Cell lysates were then sonicated to generate chromatin fragments of 200-300 bp and immunoprecipitated with c-Myc (CST, Beverly, MA, USA) or IgG as control. Precipitated chromatin DNA was recovered and analyzed by PCR.

Statistical methods

All experiments were independently repeated at least triplicate. Data were expressed as mean±SD. Differences between two independent groups were tested with the student t test. All statistical analyses were carried out using SPSS version 18.0 and presented with Graph Pad prism software. Kaplan-Meier survival curves were plotted and log rank test was done. The significance of various variables for survival was analyzed by Cox proportional hazards model in a multivariate analysis. The results were considered to be statistically significant at $P<0.05$.

Results

CCAT1 was significantly associated with prognosis of HCC

We performed Kaplan-Meier analysis to test whether the CCAT1 expression levels were associated with overall survival and relapse-free survival of the HCC patients. 86 HCC samples were divided into CCAT1 low expression group (n=43) and high expression group (n=43), median was used as cut off. Patients with high CCAT1 expression had a worse relapse-free survival (RFS) than the low expression group. The difference was statistically significant (P=0.002) (Figure 1A). Similarly, a statistically significant association of CCAT1 with overall survival (OS) was also obtained (P=0.003) (Figure 1B). Additionally, Cox proportional regression analysis suggested that CCAT1 were independent prognostic factor for the HCC patients (adjusted hazard ratio (AHR): 7.17 [95% Confidence Interval (CI): 3.20-16.05]; P=0.001) (Table 1).

c-Myc promotes CCAT1 transcription and upregulates its expression in HCC cells

Recently, many important transcript factors are found to be involved in regulating lncRNA transcription. To explore which transcript factors would activate CCAT1 expression, we analyze the potential transfect factor binding sites in the promoter region of CCAT1 and found that there is one E-box element that could be recognized by c-Myc. To further determine whether c-Myc could be directly binding to CCAT1 promoter regions and lead to the upregulation of CCAT1, we assessed CCAT1 expression after HCC cells transfected with c-Myc siRNA. The results demonstrated that c-Myc expression levels were significantly down regulated in HCC cells after c-Myc siRNA transfection (Figure 2A). Additionally, results showed that CCAT1 expression was obviously

Table 1. Cox proportional Hazards Analyses

A

Clinical factor	*P* value	Univariable analysis	
		HR	95% CI
CCAT1	0.002*	3.33	1.60-6.96
AGE	0.58	1.21	0.62-2.34
GENDER	0.96	1.02	0.52-1.97
CIRHOSIS	0.51	1.80	0.41-2.55
ALT	0.94	1.03	0.51-2.07
AFP	0.19	1.63	0.78-3.38
GRADE	0.001	3.27	1.65-6.52

B

Clinical factor	*P* value	Multivariable analysis	
		HR	95% CI
CCAT1	0.002*	3.33	1.60-6.96
AGE	0.58	1.21	0.62-2.34
GENDER	0.96	1.02	0.52-1.97
CIRHOSIS	0.51	1.80	0.41-2.55
ALT	0.94	1.03	0.51-2.07
AFP	0.19	1.63	0.78-3.38
GRADE	0.001	3.27	1.65-6.52

*Indicates *P* <0.05; HR: Hazard ratio; AHR: Adjusted hazard ratio; CI: Confidence interval

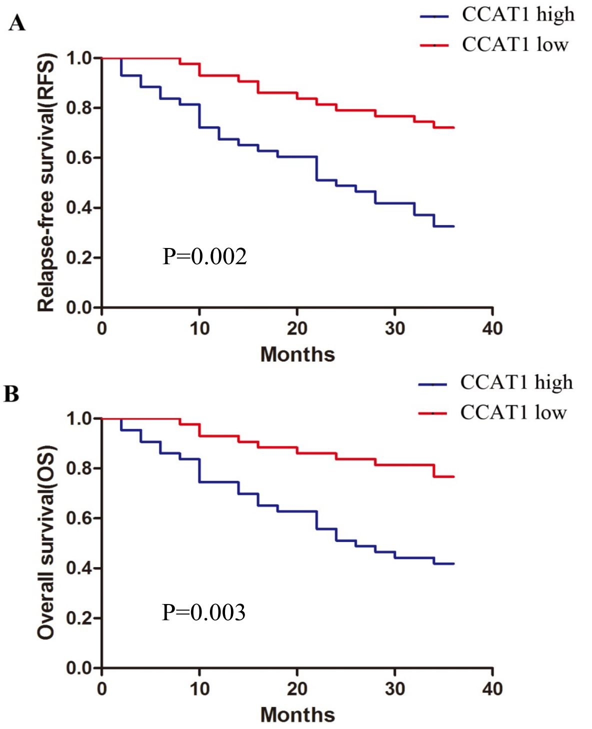

Figure 1. Association of CCAT1 Expression with Relapse-Free Survival and Overall Survival of the Patients with HCC. Kaplan-Meier curves for (A) RFS, and (B) OS stratified according to low or high expression of CCAT1 in HCC. *P* values were calculated using the log-rank test

decreased in c-Myc siRNA transfected HCC cells (Figure 2B). Moreover, chromatin immunoprecipitation assays indicated that c-Myc could directly bind to the promoter of CCAT1 (Figure 2C). Our data indicated that c-Myc was involved in CCAT1 regulation and contributed to HCC development.

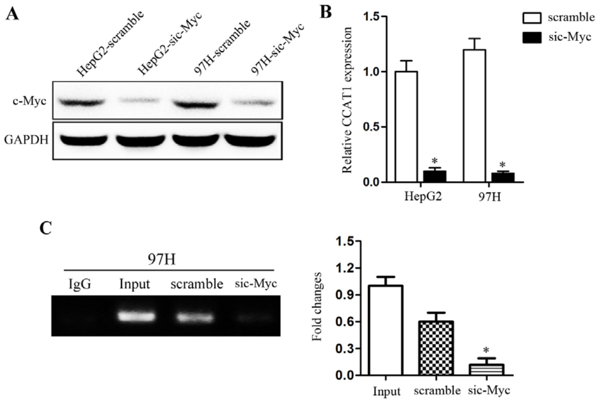

Figure 2. c-Myc Promotes CCAT1 Transcription and Upregulates its Expression in HCC Cells. (A) Both HepG2 and 97H cells were transfected with siRNA/c-Myc. The siRNA/scramble cells were used as the control. The transfection efficiency was verified by using western blotting assays. (B) The mRNA expression levels of CCAT1 in HepG2 and 97H cells transfected with siRNA/c-Myc and siRNA/scramble were measured by qRT-PCR. The mRNA expression level of GAPDH was served as control. Data are represented as mean±SD. * indicates *P*<0.05. Every independent experiment was performed three times. (C) Immunoprecipitation of chromatin from 97H either siRNA/scramble treated or siRNA/c-Myc treated using anti-c-Myc antibodies. As control, no antibody was used. Precipitated chromatin was PCR-amplified using CCAT1 primers. Data are represented as mean±SD. * indicates *P*<0.05. Every independent experiment was performed three times

Discussion

HCC is a worldwide disease with a high incidence, especially in China. The five-year survival rate for HCC is very low, and more than 500,000 people die of it each year despite advanced therapeutic treatments such as surgery, chemotherapy and radiotherapy (Chen, Therneau, Orsini, and Qiao, 2011; Cai et al., 2014). Therefore, it is essential to thoroughly explore the pathogenesis and develop novel and effective means for the treatment of HCC (G. J. Li et al., 2013). Lots of evidences have indicated that long non-coding RNAs played an vital role in carcinogenesis and cancer progression (Gibb, Brown, and Lam, 2011; Zhu et al., 2012; Uva et al., 2013; Arase et al., 2014; Hu et al., 2014; Z. Li and Rana, 2014; Xing et al., 2014; Haerty and Ponting, 2015). It has been well documented that CCAT1 was involved in pathogenesis of several malignancies via its interaction with c-Myc (Alaiyan et al., 2013; Yang et al., 2013; He et al., 2014; Kam et al., 2014; Xiang et al., 2014; Ma et al., 2015; Mizrahi et al., 2015) , but the role of CCAT1 in development of HCC still remains unknown.

Our results indicated that the expression pattern of CCAT1 was significantly associated with clinical features of HCC patients. However, the regulators that contribute to misregulated CCAT1 and the biological function of CCAT1 in HCC is not well documented. Recently, more and more evidence indicated that the lncRNA transcription can be regulated by both some key transcript factors and epigenetic modifications (Wu, Kallin, Zhang, 2010; Zhu et al., 2012; Engreitz et al., 2013; Kallen et al., 2013; Uva et al., 2013; Flemming et al., 2014; Xiang et al., 2014; Xing et al., 2014; Haerty and Ponting, 2015). For example, p53 could promote lncRNA-p21 transcription

and E2F1 regulates lncRNA ERIC expression (Bao et al., 2015), while the core catalytic subunit of polycomb repressive complex 2 (PRC2) EZH2 could repress lncRNA SPRY4-IT1 transcription via epigenetic maintenance of the H3K27me3 (Sun et al., 2014). In the present study, we demonstrated that there are some transcription factor c-Myc-binding sites in the promoter of CCAT1, and up-regulation of c-Myc could promote CCAT1 expression in HCC cell lines. Additionally, the ChIP assays further indicated that c-Myc could bind to the CCAT1 promoter region directly, which suggested that c-Myc could activate lncRNA CCAT1 expression in HCC cells and contribute to HCC development. Moreover, we performed Kaplan-Meier analysis to determine whether the CCAT1 expression levels were associated with overall survival and relapse-free survival of the HCC patients. 86 HCC samples were divided into CCAT1 low expression group and high expression group, median was used as cut off. Patients with high CCAT1 expression had a worse relapse-free survival (RFS) than the low expression group. The difference was statistically significant. Similarly, a statistically significant association of CCAT1 with overall survival (OS) was also obtained. Additionally, Cox proportional regression analysis suggested that CCAT1 were independent prognostic factor for the HCC patients.

In conclusion, our results also illuminated that CCAT1 could be a potential biomarker for predicting the survival of HCC patients. Additionally, our results indicated that c-Myc contribute to the upstream regulation of CCAT1 in HCC. These findings may attribute to a better understand of the upstream regulation mechanism of CCAT1. Taken together, our data indicated a c-Myc/CCAT1 axis signaling might be involved in HCC development, which may play a vital role in the tumorgenesis of HCC and could also serve as potential prognosis markers and potential targets for therapy in the future.

Acknowledgements

This study was supported by Natural Science Foundation of Shandong Province in China (Y2008C22 and ZR2014HM099), Excellent Youth Scientist Foundation of Shandong Province in China (2007BS03038), and others of Shandong Province (2014WS0093, 2014GGB14041, 2014WS0095 and 2014WS0096).

References

Alaiyan B, Ilyayev N, Stojadinovic A, et al (2013). Differential expression of colon cancer associated transcript1 (CCAT1) along the colonic adenoma-carcinoma sequence. *BMC Cancer*, **13**, 196.

Arase M, Horiguchi K, Ehata S, et al (2014). Transforming growth factor-beta-induced lncRNA-Smad7 inhibits apoptosis of mouse breast cancer JygMC(A) cells. *Cancer Sci*, **105**, 974-82.

Bao X, Wu H, Zhu X, et al (2015). The p53-induced lincRNA-p21 derails somatic cell reprogramming by sustaining H3K9me3 and CpG methylation at pluripotency gene promoters. *Cell Res*, **25**, 80-92.

Cai JB, Shi GM, Dong ZR, et al (2014). Ubiquitin-specific protease 7 accelerates p14 degradation by deubiquitinating thyroid hormone receptor-interacting protein 12 and promotes hepatocellular carcinoma progression. *Hepatol*, [Epub ahead of print].

Cao C, Sun J, Zhang D, et al (2015). The Long intergenic noncoding RNA UFC1, a target of MicroRNA 34a, interacts with the mRNA stabilizing protein HuR to increase levels of beta-catenin in HCC cells. *Gastroenterol*, **148**, 415-26 e18.

Chen M, Therneau T, Orsini LS, et al (2011). Design and rationale of the HCC BRIDGE study in China: a longitudinal, multicenter cohort trial in hepatocellular carcinoma. *BMC Gastroenterol*, **11**, 53.

Engreitz JM, Pandya-Jones A, McDonel *P*, et al (2013). The Xist lncRNA exploits three-dimensional genome architecture to spread across the X chromosome. *Science*, **341**, 1237973.

Fang TT, Sun XJ, Chen J, et al (2014). Long non-coding RNAs are differentially expressed in hepatocellular carcinoma cell lines with differing metastatic potential. *Asian Pac J Cancer Prev*, **15**, 10513-24.

Flemming JA, Yang JD, Vittinghoff E, et al (2014). Risk prediction of hepatocellular carcinoma in patients with cirrhosis: the ADRESS-HCC risk model. *Cancer*, **120**, 3485-93.

Gibb EA, Brown CJ, Lam WL (2011). The functional role of long non-coding RNA in human carcinomas. *Mol Cancer*, **10**, 38.

Haerty W, Ponting CP (2015). Unexpected selection to retain high GC content and splicing enhancers within exons of multiexonic lncRNA loci. *RNA*, [Epub ahead of print].

He X, Tan X, Wang X, et al (2014). C-Myc-activated long noncoding RNA CCAT1 promotes colon cancer cell proliferation and invasion. *Tumour Biol*, **35**, 12181-8.

Hu X, Feng Y, Zhang D, et al (2014). A functional genomic approach identifies FAL1 as an oncogenic long noncoding RNA that associates with BMI1 and represses p21 expression in cancer. *Cancer Cell*, **26**, 344-57.

Kallen AN, Zhou XB, Xu J, et al (2013). The imprinted H19 lncRNA antagonizes let-7 microRNAs. *Mol Cell*, **52**, 101-12.

Kam Y, Rubinstein A, Naik S, et al (2014). Detection of a long non-coding RNA (CCAT1) in living cells and human adenocarcinoma of colon tissues using FIT-PNA molecular beacons. *Cancer Lett*, **352**, 90-6.

Li GJ, Harrison TJ, Yang JY, et al (2013). Combined core promoter mutations and pre-S deletion of HBV may not increase the risk of HCC: a geographical epidemiological study in Guangxi, China. *Liver Int*, **33**, 936-43.

Li Z, Rana TM (2014). Decoding the noncoding: prospective of lncRNA-mediated innate immune regulation. *RNA Biol*, **11**, 979-85.

Ma MZ, Chu BF, Zhang Y, et al (2015). Long non-coding RNA CCAT1 promotes gallbladder cancer development via negative modulation of miRNA-218-5p. *Cell Death Dis*, **6**, 1583.

Mizrahi I, Mazeh H, Grinbaum R, et al (2015). Colon Cancer Associated Transcript-1 (CCAT1) Expression in Adenocarcinoma of the Stomach. *J Cancer*, **6**, 105-10.

Spizzo R, Almeida MI, Colombatti A, et al (2012). Long non-coding RNAs and cancer: a new frontier of translational research? *Oncogene*, **31**, 4577-87.

Sun M, Liu XH, Lu KH, et al (2014). EZH2-mediated epigenetic suppression of long noncoding RNA SPRY4-IT1 promotes NSCLC cell proliferation and metastasis by affecting the epithelial-mesenchymal transition. *Cell Death Dis*, **5**, 1298.

Uva *P*, Da Sacco L, Del Corno M, et al (2013). Rat mir-155 generated from the lncRNA Bic is 'hidden' in the alternate genomic assembly and reveals the existence of novel mammalian miRNAs and clusters. *RNA*, **19**, 365-79.

Wu SC, Kallin EM, Zhang Y (2010). Role of H3K27 methylation in the regulation of lncRNA expression. *Cell Res*, **20**, 1109-16.

Xiang JF, Yin QF, Chen T, et al (2014). Human colorectal cancer-specific CCAT1-L lncRNA regulates long-range chromatin interactions at the MYC locus. *Cell Res*, **24**, 513-31.

Xing Z, Lin A, Li C, et al (2014). lncRNA directs cooperative epigenetic regulation downstream of chemokine signals. *Cell*, **159**, 1110-25.

Yang F, Xue X, Bi J, et al (2013). Long noncoding RNA CCAT1, which could be activated by c-Myc, promotes the progression

of gastric carcinoma. *J Cancer Res Clin Oncol*, **139**, 437-45.
Zhu Z, Gao X, He Y, et al (2012). An insertion/deletion polymorphism within RERT-lncRNA modulates hepatocellular carcinoma risk. *Cancer Res*, **72**, 6163-72.
