# Supplementary material for: Expression of Concern: Prognostic value of long non-coding RNA CCAT1 expression in patients with cancer: A meta-analysis
Source: PLoS One. 2023 Apr 20;18(4):e0284940. doi: 10.1371/journal.pone.0284940 (PMC10118116; doi:10.1371/journal.pone.0284940)
Supplement: S1 File — (ZIP) [file pone.0284940.s001.zip › 3 The 11 included studies in PDF/4.pdf]

Tumor Biol. (2014) 35:12181–12188
DOI 10.1007/s13277-014-2526-4

RESEARCH ARTICLE

# C-Myc-activated long noncoding RNA CCAT1 promotes colon cancer cell proliferation and invasion

**Xiaolu He · Xueming Tan · Xiang Wang · Heiying Jin · Li Liu · Limei Ma · Hong Yu · Zhining Fan**

Received: 29 July 2014 / Accepted: 20 August 2014 / Published online: 4 September 2014
© International Society of Oncology and BioMarkers (ISOBM) 2014

**Abstract** Recently, more and more evidence are rapidly accumulating that long noncoding RNAs (lncRNAs) are involved in human tumorigenesis and misregulated in many cancers, including colon cancer. LncRNA could regulate essential pathways that contribute to tumor initiation and progression with their tissue specificity, which indicates that lncRNA would be valuable biomarkers and therapeutic targets. Colon cancer-associated transcript 1 (CCAT1) is a 2628 nucleotide-lncRNA and located in the vicinity of a well-known transcription factor c-Myc. CCAT1 has been found to be upregulated in many cancers, including gastric carcinoma and colonic adenoma-carcinoma. However, its roles in colon cancer are still not well documented and need to be investigated. In this study, we aim to investigate the prognostic value and biological function of CCAT1 and discover which factors may contribute to the deregulation of CCAT1 in colon cancer. Our results revealed that CCAT1 was significantly overexpressed in colon cancer tissues when compared with normal tissues, and its increased expression was correlated with patients' clinical stage, lymph nodes metastasis, and survival time after surgery. Moreover, c-Myc could promote CCAT1 transcription by directly binding to its promoter region, and upregulation of CCAT1 expression in colon cancer cells promoted cell proliferation and invasion. These data suggest that c-Myc-activated lncRNA CCAT1 expression contribute to colon cancer tumorigenesis and the metastatic process and could predict the clinical outcome of colon cancer and be a potential target for lncRNA direct therapy.

**Keywords** Colon cancer · c-Myc · CCAT1 · Cell proliferation · Invasion

## Introduction

Colon cancer is one of the leading causes of cancer-related deaths in both eastern and western countries [1]. The colon cancer carcinogenesis involving multistep progression and is associated with genetic or epigenetic abnormalities, including mutations of the k-ras and Apc genes, as well as hypermethylation of DNA mismatch repair genes [2–4]. Recently, lots of evidence highlight that noncoding RNAs (ncRNAs) are also closely associated with colon cancer tumorigenesis [5, 6]. As an important member of ncRNA, microRNAs have been found to play key roles in colon cancer development and progression. For example, miR-18a could induce the apoptosis of colon cancer cells through directly binding to oncogenic hnRNP A1 mRNA and leads to the autophagolysosomal degradation of the protein [7]. Moreover, miR-139-5p involved in colon cancer development through inhibiting cell proliferation, metastasis, and inducing apoptosis and cell cycle arrest by directly targeting NOTCH1 [8]. However, the role of long noncoding RNAs (lncRNAs), another newly known member of ncRNA family, in colon cancer tumorigenesis are still not well documented and needed to be investigated.

With the advanced development of whole genome and transcriptome sequencing technologies and the ENCODE project, it is more and more clear that only a minuscule fraction of the human genome encodes proteins, and most of

Xiaolu He and Xueming Tan contributed equally to this work.

X. He · X. Tan · L. Ma · H. Yu
The Second Affiliated Hospital of Nanjing Medical University, Nanjing, China

X. Wang · L. Liu · Z. Fan (✉)
Department of Endoscopy Center, The First Affiliated Hospital of Nanjing Medical University, Nanjing 210029, People's Republic of China
e-mail: tgzy111@126.com

H. Jin
National Medical Center of Anorectal Surgery, Nanjing Hospital of Traditional Chinese Medicine, Nanjing, China

the genome DNA is represented in processed transcripts without or lacking of protein-coding capacity [9]. In the past decade, particular attention has been focused on the exploding class of transcripts referred to as lncRNAs, which are arbitrarily defined as being longer than 200 nucleotides [10, 11]. Although only a small number of lncRNAs were characterized, steadily growing evidence revealed that lncRNAs have authentic biological roles. For example, lncRNAs have been reported to have been implicated in regulating imprinting, cell cycle and apoptosis regulation, pluripotency, meiotic entry, retrotransposon silencing and telomere length, etc. [12–14]. To date, a lot of lncRNAs are found to be misregulated in multiple cancers, and it is anticipated that better understanding of the roles of lncRNAs in cancer will promote the development of novel and effective therapeutic strategies [15].

LncRNA colon cancer-associated transcript 1 (CCAT1) is a recently discovered 2628 nucleotide-lncRNA, which is located in the vicinity of a well-known transcription factor c-Myc. Previous study showed that CCAT1 is upregulated in tissues obtained from colon cancer patients compared with the normal human tissues [16]. Moreover, studies in human tissues revealed minimal CCAT1 expression in normal liver and small bowel tissue; however, there is no CCAT1 expression in many other human tissues tested. In addiction, the chromosome 8q24.21 location where CCAT1 is transcripted was described as a “hot spot” with many genetic alternations in colon cancer [17, 18]. However, the factors involved in CCAT1 upregulation and the biological roles of CCAT1 in colon cancer are still unknown. In this study, we found that CCAT1 was significantly overexpressed in colon cancer tissues, and its increased expression was correlated with patients clinical stage, lymph nodes metastasis, and survival time. Moreover, c-Myc could promote CCAT1 transcription and upregulation of CCAT1 expression in colon cancer cells, while CCAT1 overexpression promoted colon cancer cell proliferation and invasion. These data suggest that c-Myc-activated lncRNA CCAT1 expression contribute to colon cancer tumorigenesis and the metastatic process and could predict the clinical outcome of colon cancer and be a potential target for lncRNA direct therapy.

## Materials and methods

### Tissue samples

Colon cancer and normal tissues were obtained between 2009 and 2011 from patients who underwent primary surgical resection of colon cancer with informed consent at the Nanjing Hospital of Traditional Chinese Medicine, China. No local or systemic treatments were conducted in these patients before the operation. All these tissue samples were immediately snap-frozen in liquid nitrogen and stored at −80 °C until total RNA was extracted. This study was approved by the Research Ethics Committee of Nanjing Medical University. Informed consent was obtained from all patients.

### Cell lines and culture conditions

Human colon cancer cell lines SW480 and SW620 were purchased from the Institute of Biochemistry and Cell Biology of the Chinese Academy of Sciences (Shanghai, China). Cells were grown in high-glucose Dulbecco’s modified Eagle’s medium (HT-29) or Roswell Park Memorial Institute 1640 (SW480 and SW620) supplemented with 10 % fetal bovine serum (10 % FBS), 100 U/ml penicillin, and 100 mg/ml streptomycin (Invitrogen, Shanghai, China) in humidified air at 37 °C with 5 % $CO_2$.

### qPCR analyses

Total RNA of tissues and cells was isolated with TRIzol reagent (Invitrogen, Carlsbad, CA, USA) according to the manufacturer’s protocol. For analysis of CCAT1 and c-MYC mRNA expression, 1 μg total RNA was reverse transcribed in a final volume of 20 μl using random primers under standard conditions using the PrimeScript RT Reagent Kit. SYBR Premix Ex Taq (TaKaRa, Dalian, China) was used to detect CCAT1 and c-MYC according to the manufacturer’s instructions, and glyceraldehyde 3-phosphate dehydrogenase (GAPDH) was used as an internal control. The primers were designed as follows: CCAT1, forward primer: 5′ CATTGG GAAAGGTGCCGAGA 3′, reverse primer: 5′ ACGCTTAG CCATACAGAGCC 3′; c-Myc, forward primer: 5′ CCACAG CAAACCTCCTCACA 3′, reverse primer: 5′ TCCAACTT GACCCTCTTGGC 3′; GAPDH, forward primer: 5′ GGGA GCCAAAAGGGTCAT 3′, and reverse primer: 5′ GAGTCC TTCCACG ATACCAA 3′. The relative expression levels of RNA were calculated based on the difference between amplification of target genes and GAPDH mRNA using the 2-Δct method.

### Plasmid constructs

The sequence of c-Myc and CCAT1 was synthesized and subcloned into pcDNA3.1 (Invitrogen, Shanghai, China). Ectopic expression of c-Myc or CCAT1 was achieved by using the pcDNA-c-Myc or pcDNA-CCAT1 transfection and empty pcDNA vector was used as control. The expression levels of c-MYC and CCAT1 were detected by quantitative PCR (qPCR).

### Transfection of colon cancer cells

All plasmid vectors (pcDNA-c-Myc, pcDNA-CCAT1, and empty vector) for transfection were extracted by DNA Midiprep kit (Qiagen, Hilden, Germany). SW480 and SW620 cells cultured on six-well plate were transfected with the pcDNA-c-Myc, pcDNA-CCAT1, or empty vector using Lipofectamine 2000 (Invitrogen, Shanghai, China) according to the manufacturer's instructions. Cells were harvested after 48 h for qPCR and Western blot analyses.

### Cell proliferation assays

Colon cancer cells proliferation was monitored using Cell Proliferation Reagent Kit I (MTT) (Roche Applied Science). pcDNA-CCAT1 and empty vector-transfected SW480 and SW620 cells (3,000/well) were allowed to grow in 96-well plates. Cell proliferation was measured every 24 h following the manufacturer's protocol. For colony formation assay, a total of 500 pcDNA-CCAT1 and empty vector cells were placed in a fresh six-well plate and maintained in media containing 10 % FBS, replacing the medium every 5 days. After 14 days, cells were fixed with methanol and stained with 0.1 % crystal violet (Sigma-Aldrich). Visible colonies were manually counted. Triplicate wells were measured for each treatment group.

### Cell migration and invasion assays

In migration assays, $3\times10^4$ cells at 48 h after transfection were placed into the upper chamber of an insert in serum-free medium (8-μm pore size; Millipore); for the invasion assays, $1\times10^5$ cells in serum-free medium were placed into the upper chamber coated with Matrigel (Sigma-Aldrich). Medium containing 10 % FBS was added to the lower chamber. The cells remaining on the upper membrane were removed after incubation for 24 h, and cells that had migrated or invaded through the membrane were stained with 0.1 % crystal violet, imaged, and counted using an IX71 inverted microscope (Olympus, Tokyo, Japan). Experiments were independently repeated three times.

### Chromatin immunoprecipitation

SW480 and SW620 cells were treated with formaldehyde and incubated for 10 min to generate DNA-protein cross-links. Cell lysates were then sonicated to generate chromatin fragments of 200–300 bp and immunoprecipitated with c-Myc (CST) or IgG as control. Precipitated chromatin DNA was recovered and analyzed by qPCR.

### Statistical analysis

Student's *t* test (two-tailed), one-way ANOVA, and the Mann–Whitney *U* test were used to analyze data, along with SPSS 17.0 (IBM, IL, USA). *P* values of less than 0.05 were considered statistically significant.

## Results

### CCAT1 expression is upregulated in human colon cancer tissues

To investigate the CCAT1 expression in colon cancer tissues, we performed qPCR analysis in 48 colon cancer tissues and normal counterparts. The results showed that expression of CCAT1 was significantly upregulated in colon cancer tissues (Fig. 1a). Furthermore, we investigated the increased CCAT1 expression with clinical pathological features of colon cancer patients and revealed a significant association between CCAT1 upregulation and advanced pathological stage and lymph nodes metastasis (Fig. 1b, c). However, CCAT1 expression was not correlated with patient age and gender (Table 1).

Furthermore, to evaluate the correlation between CCAT1 expression and colon cancer patient prognosis, Kaplan–Meier survival analyses using patient postoperative survival were performed. According to the median ratio of relative CCAT1 expression, the 48 colon cancer patients were classified into two groups: high-CCAT1 group ($n$=24, CCAT1 expression ratio≥mean ratio) and low-CCAT1 group ($n$=24, CCAT1 expression ratio≤ mean ratio). The results of Kaplan–Meier survival curve revealed that patients with increased CCAT1 expression levels had significantly shorter survival times than those with lower CCAT1 expression levels (Fig. 1d). These findings indicated that increased CCAT1 expression plays a key role in colon cancer development and progression.

### c-Myc promotes CCAT1 transcription and upregulates its expression in colon cancer cells

Recently, many important transcript factors are found to be involved in regulating lncRNA transcription. To investigate which transcript factors would activate CCAT1 expression, we analyze the potential transfect factor binding sites in the promoter region of CCAT1 (http://jaspar.genereg.net) and found that there is one E-box element that could be recognized by c-Myc. To further determine whether c-Myc could be directly binding to CCAT1 promoter regions and lead to the upregulation

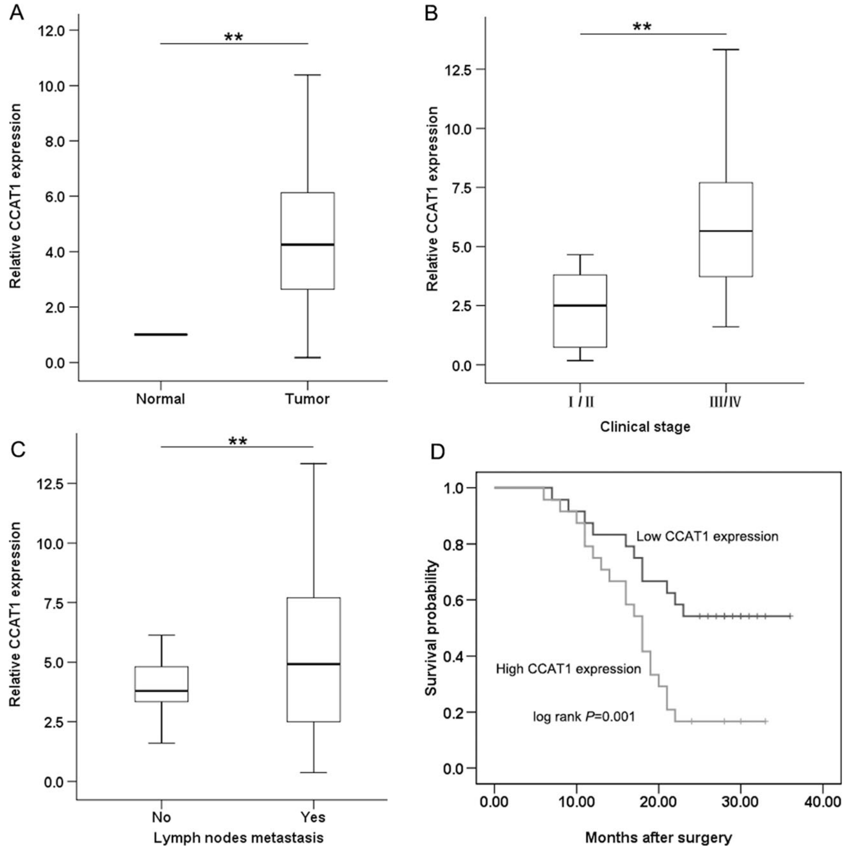


**Fig. 1** Relative CCAT1 expression in colon cancer tissues and its clinical significance. **a** Relative expression of CCAT1 was examined by qPCR and normalized to GAPDH expression in colon cancer tissues ($n$=48) in comparison with corresponding nontumor normal tissues ($n$=48). **b** CCAT1 expression was significantly higher in patients with advanced clinical stage. **c** CCAT1 expression was significantly higher in patients with lymph nodes metastasis. **d** Kaplan–Meier overall survival curves according to CCAT1 expression level. The overall survival of the high-CCAT1 group ($n$=24: CCAT1 expression ratio≥median ratio) was significantly lower than that of low-CCAT1 group ($n$=24: CCAT1 expression ratio≤median ratio). ($P$<0.001, log-rank test). *$P$<0.05; **$P$<0.01

**Table 1** Correlation between CCAT1 expression and clinicopathological characteristics in patients with colon cancer

| Clinical parameter | CCAT1 | | Chi-squared test $P$ value |
|---|---|---|---|
| | Low-CCAT1 group (no. of cases) | High-CCAT1 group (no. of cases) | |
| Age (years) | | | |
| <50 | 11 | 14 | 0.564 |
| >50 | 13 | 10 | |
| Gender | | | |
| Male | 9 | 14 | 0.248 |
| Female | 15 | 10 | |
| Size | | | |
| >5 cm | 8 | 17 | 0.02 |
| <5 cm | 16 | 7 | |
| TNM stage | | | |
| I/II | 19 | 9 | 0.008 |
| III/IV | 5 | 15 | |
| Lymphatic metastasis | | | |
| Yes | 6 | 15 | 0.019 |
| No | 18 | 9 | |
| Distant metastasis | | | |
| Yes | 0 | 4 | 0.055 |
| No | 24 | 20 | |

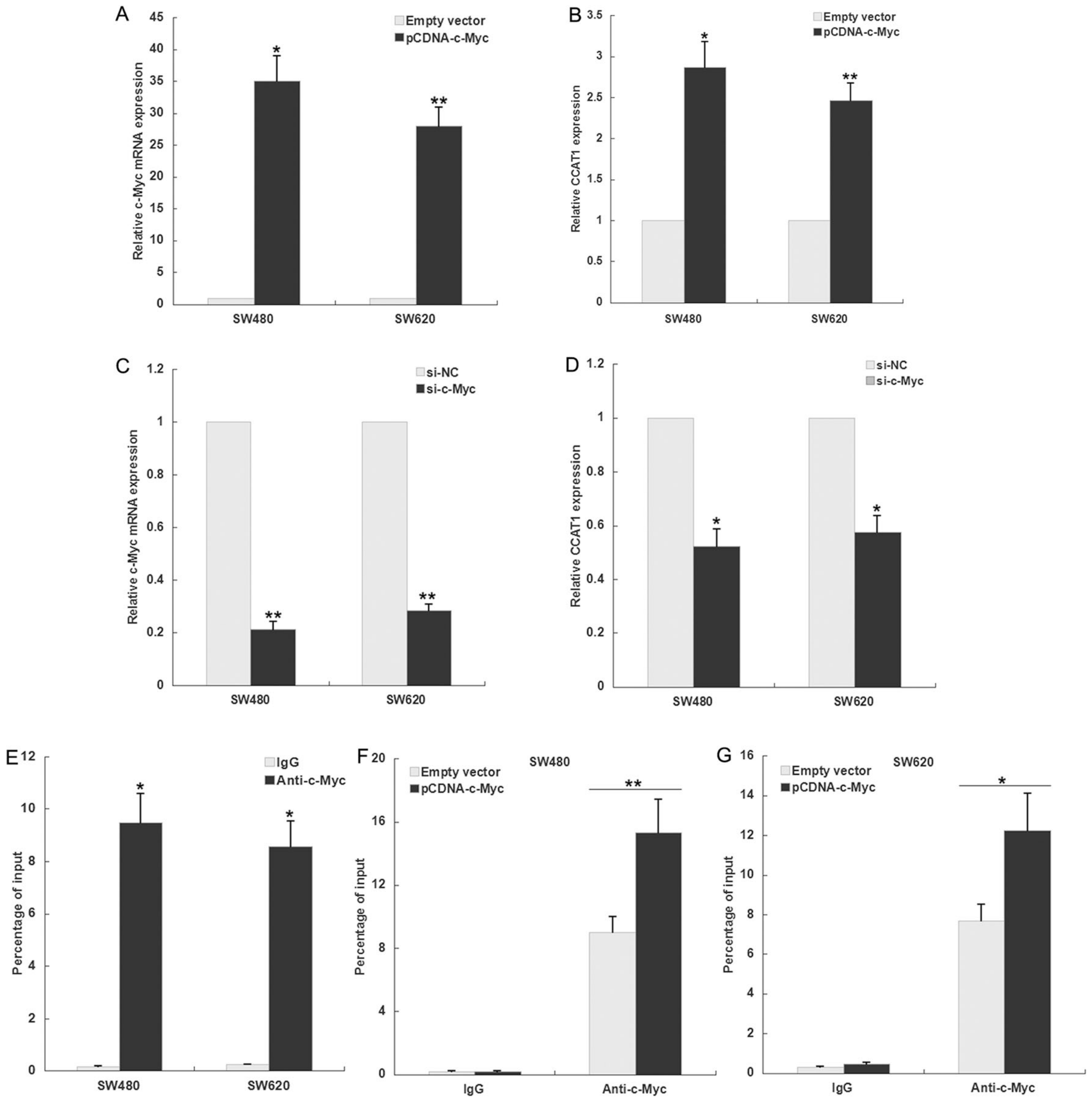


**Fig. 2** c-Myc promotes CCAT1 transcription through binding to its promoter regions. **a** QPCR analysis of c-Myc levels after the transfection of the pcDNA-c-Myc or the control vector into SW480 and SW620 cells. **b** Expression levels of the CCAT1 after the transfection of pcDNA-c-Myc or empty vector into SW480 and SW620 cells. **c** QPCR assays of the CCAT1 levels after the transfection of c-Myc siRNA or the control siRNA into SW480 and SW620. **d** Expression levels of the CCAT1 after the transfection of c-Myc siRNA or the control siRNA into SW480 and SW620 cells. **e** Chromatin immunoprecipitation (ChIP) assays were used to assess c-Myc binding at the promoter region of CCAT1 containing the E-box element. The ChIP-derived DNA was amplified by qPCR with specific primers and expressed as a percentage of input DNA. Data are shown as the mean±standard error, based on at least three independent experiments

of CCAT1, we assessed CCAT1 expression after colon cancer cells transfected with pcDNA-c-Myc vector. The results showed that c-Myc expression levels were significantly upregulated or downregulated in colon cancer cells after pcDNA-c-Myc or si-c-Myc transfection (Fig. 2a, c). Moreover, qPCR results showed that CCAT1 expression was increased or decreased in pcDNA-c-Myc or si-c-Myc-transfected colon cancer cells (Fig. 2b, d). Additionally, chromatin immunoprecipitation assays indicated that c-Myc could directly bind to CCAT1 promoter (Fig.2e, f, g). Their data suggest that c-Myc is involved in CCAT1 upregulation and contributed to colon cancer development.

## CCAT1 overexpression promotes colon cancer cells proliferation

CCAT1 was overexpressed in SW480 and SW620 cells by transfecting them with pcDNA-CCAT1. The results of qPCR showed that CCAT1 expression was increased by 22- or

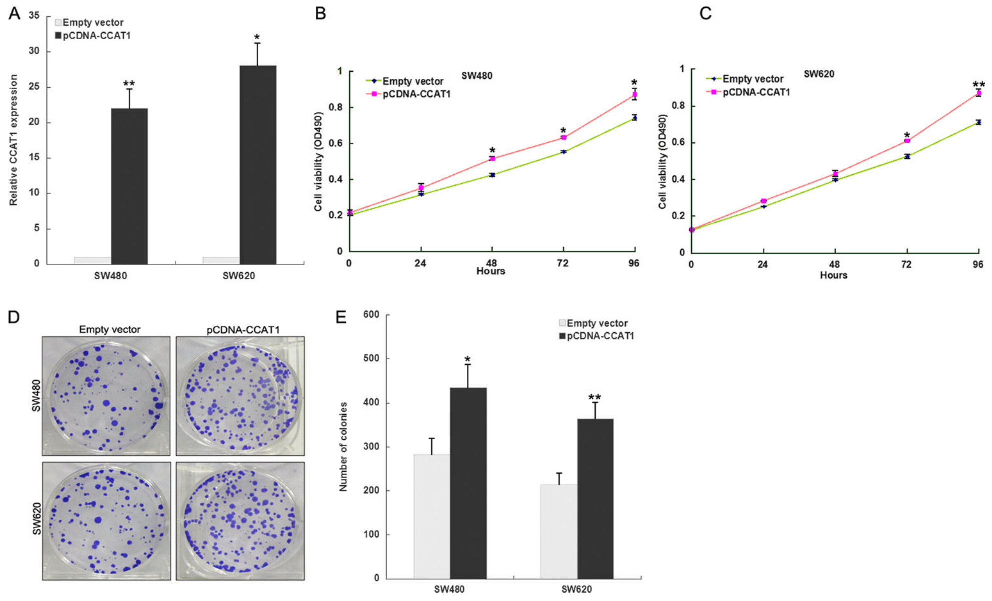


**Fig. 3** The effect of CCAT1 overexpression on colon cancer cells proliferation in vitro. SW480 and SW620 cells were transfected with pcDNA-CCAT1 or empty vector, respectively. **a** QPCR analyses of CCAT1 expression level following treatment SW480 and SW620 cells with pcDNA-CCAT1 or empty vector. **b**, **c** MTT assay was performed to determine the proliferation of pcDNA-CCAT1-transfected SW480 and SW620 cells. Data represent the mean±s.d. from three independent experiments. **d**,**e** Colony-forming growth assay was performed to determine the colony formation ability of pcDNA-CCAT1 transfected SW480 and SW620 cells. The colonies were counted and captured. *$P<0.05$; **$P<0.01$

28-fold in SW480 and SW620 cells following transfection with pcDNA-CCAT1 compared with control cells (Fig. 3a). To assess the biological function of CCAT1 in colon cancer cells, we investigated the effects of

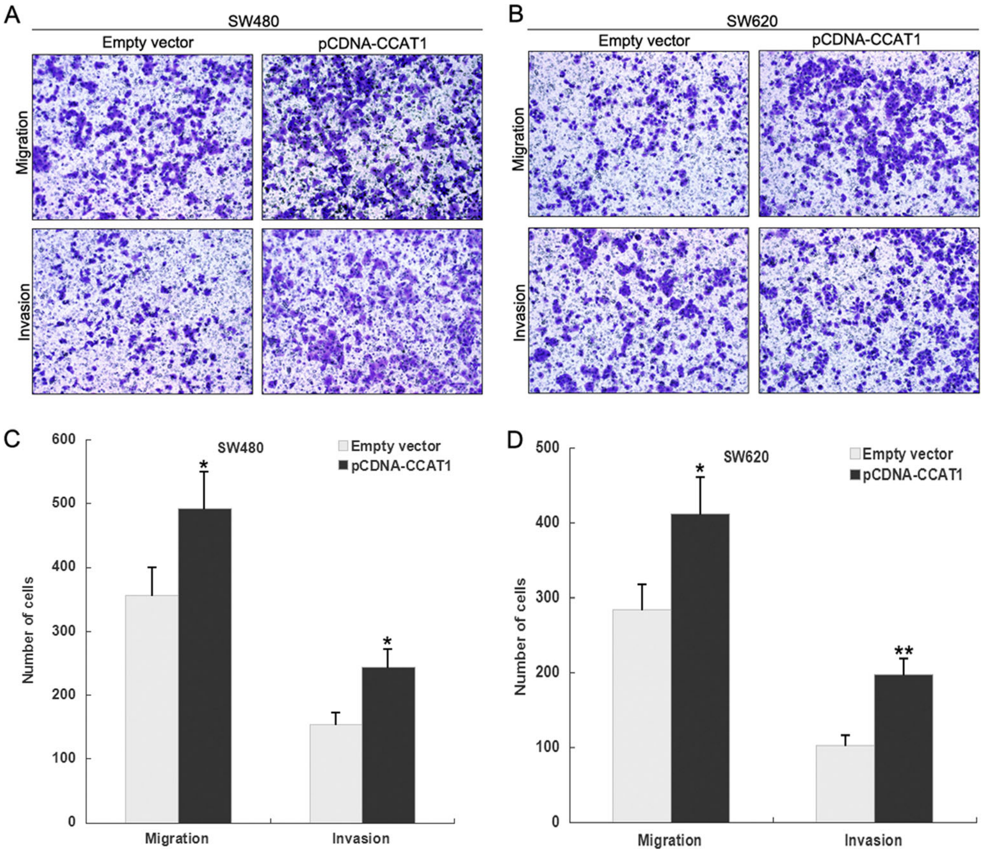


**Fig. 4** The effect of CCAT1 overexpression on colon cancer cells migration and invasion in vitro. SW480 and SW620 cells were transfected with pcDNA-CCAT1 or empty vector, respectively. **a**, **b** Transwell assays were used to investigate the changes in migratory and invasive abilities of SW480 and SW620 cells. *$P<0.05$; **$P<0.01$

CCAT1 overexpression on cell proliferation. MTT assays revealed that increased CCAT1 expression could promote SW480 and SW620 cell growth (Fig. 3b, c). Similarly, the results of colony-formation assays showed that clonogenic survival was increased following enhanced CCAT1 expression in SW480 and SW620 cells (Fig. 3d, e).

Upregulated CCAT1 expression promotes the migration and invasion of colon cancer cells

Tumor cells invasion is a significant aspect of cancer progression, including the migration of tumor cells into contiguous tissues. Therefore, to investigate whether CCAT1 has a direct functional role in regulating colon cancer cell migration and invasion, we evaluated colon cancer cell invasion through Matrigel and migration through transwell. The results showed that upregulation of CCAT1 significantly promoted the migration of SW480 and SW620 cells compared with the control. Similarly, invasion of SW480 and SW620 cells was increased following overexpression of CCAT1 (Fig. 4a, b). These results indicate that CCAT1 could promote the migratory and invasive phenotype of colon cancer cells.

## Discussion

Recently, many large-scale gene expression studies of tumor samples have revealed that lots of lncRNAs are misregulated in multiple cancers, including colon cancer [19, 20]. For example, lncRNA HOTAIR is a powerful predictor of metastasis and poor prognosis via regulating cell epithelial-mesenchymal transition in colon cancer [21]. Moreover, lncRNA CCAT2, mapping to 8q24, contributes to metastatic progression and chromosomal instability in colon cancer by regulating MYC and WNT expression through TCF7L2-mediated transcriptional regulation [6]. In this study, we found that lncRNA CCAT1 is significantly overexpressed in colon cancer tissues and correlated with patients' survival time, indicating that CCAT1 could be an important role in colon cancer and implicate the potential application of CCAT1 in the treatment of colon cancer.

CCAT1, identified by Nissan et al., was highly expressed in the vast majority of primary colorectal cancer (CRC) tumors, in precancerous polyps (adenomas), lymph nodes, blood, and distant CRC metastasis, but not in normal tissues [16]. Meanwhile, CCAT1 was found to be upregulated in colon adenoma-carcinoma sequence, and this overexpression is evident in both premalignant conditions and advanced metastatic disease [22]. In addition, another CCAT1 transcript CCAT1-L was identified, which is also transcribed specifically in human CRC from a locus 515 kb upstream of Myc. CCAT1-L could regulate long-range interactions between the MYC promoter and its enhancers by localizing to its transcription site and interact with CTCF to modulate chromatin conformation at these loop regions [23]. Furthermore, increased CCAT1 expression also involved in the gastric cancer tumorigenesis, and upregulation of its expression promoted gastric cancer cell proliferation and invasion [24]. However, the regulators that contribute to misregulated CCAT1 and the biological function of CCAT1 in colon cancer is not well documented.

Recently, more and more evidence indicated that the lncRNA transcription can be regulated by both some key transcript factors and epigenetic modifications. For example, p53 could promote lncRNA-p21 transcription and E2F1 regulates lncRNA ERIC expression, while the core catalytic subunit of polycomb repressive complex 2 (PRC2) EZH2 could repress lncRNA SPRY4-IT1 transcription via epigenetic maintenance of the H3K27me3 [25–27]. In the present study, we found that there are some transcription factor c-Myc-binding sites in the promoter region of CCAT1, and overexpression of c-Myc could increase CCAT1 expression in colon cancer cells. More importantly, the ChIP assays further determined that c-Myc could directly bind to the CCAT1 promoter region, which indicated that c-Myc could activate lncRNA CCAT1 expression in colon cancer cells and contribute to colon cancer development.

To further investigate the potential function role of CCAT1 in colon cancer cells, we performed gain of function assays and found that upregulation of CCAT1 expression promoted colon cancer cell proliferation, migration, and invasion. Taken together, our results showed that lncRNA CCAT1 is significantly upregulated in colon cancer tissues, and this overexpression is partly mediated by transcript factor c-Myc. Moreover, increased CCAT1 expression is correlated with colon cancer patients' clinical stage, lymph nodes metastasis, and survival time. These findings implicate that lncRNA CCAT1 may be an important target of colon cancer therapy.

**Acknowledgments** This study was supported by the National Natural Science Foundation of China (81172266 to ZNF), the Life Health Technology Foundation of Jiangsu province (BL2012031), and the Natural Science Foundation of Jiangsu province (BK2011859 to ZNF).

**Conflicts of interest** None

## References

1. Siegel R, Naishadham D, Jemal A. Cancer statistics, 2013. CA Cancer J Clin. 2013;63(1):11–30. doi:10.3322/caac.21166.
2. Capon DJ, Seeburg PH, McGrath JP, Hayflick JS, Edman U, Levinson AD, et al. Activation of Ki-ras2 gene in human colon and lung carcinomas by two different point mutations. Nature. 1983;304(5926):507–13.
3. Forrester K, Almoguera C, Han K, Grizzle WE, Perucho M. Detection of high incidence of K-ras oncogenes during human colon

tumorigenesis. Nature. 1987;327(6120):298–303. doi:10.1038/327298a0.
4. Powell SM, Zilz N, Beazer-Barclay Y, Bryan TM, Hamilton SR, Thibodeau SN, et al. APC mutations occur early during colorectal tumorigenesis. Nature. 1992;359(6392):235–7. doi:10.1038/359235a0.
5. Wu WK, Law PT, Lee CW, Cho CH, Fan D, Wu K, et al. MicroRNA in colorectal cancer: from benchtop to bedside. Carcinogenesis. 2011;32(3):247–53. doi:10.1093/carcin/bgq243.
6. Ling H, Spizzo R, Atlasi Y, Nicoloso M, Shimizu M, Redis RS, et al. CCAT2, a novel noncoding RNA mapping to 8q24, underlies metastatic progression and chromosomal instability in colon cancer. Genome Res. 2013;23(9):1446–61. doi:10.1101/gr.152942.112.
7. Fujiya M, Konishi H, Mohamed Kamel MK, Ueno N, Inaba Y, Moriichi K, et al. MicroRNA-18a induces apoptosis in colon cancer cells via the autophagolysosomal degradation of oncogenic heterogeneous nuclear ribonucleoprotein A1. Oncogene. 2013. doi:10.1038/onc.2013.429.
8. Zhang L, Dong Y, Zhu N, Tsoi H, Zhao Z, Wu CW, et al. MicroRNA-139-5p exerts tumor suppressor function by targeting NOTCH1 in colorectal cancer. Mol Cancer. 2014;13(1):124. doi:10.1186/1476-4598-13-124.
9. Djebali S, Davis CA, Merkel A, Dobin A, Lassmann T, Mortazavi A, et al. Landscape of transcription in human cells. Nature. 2012;489(7414):101–8. doi:10.1038/nature11233.
10. Mercer TR, Dinger ME, Mattick JS. Long non-coding RNAs: insights into functions. Nat Rev Genet. 2009;10(3):155–9. doi:10.1038/nrg2521.
11. Ponting CP, Oliver PL, Reik W. Evolution and functions of long noncoding RNAs. Cell. 2009;136(4):629–41. doi:10.1016/j.cell.2009.02.006.
12. Geisler S, Coller J. RNA in unexpected places: long non-coding RNA functions in diverse cellular contexts. Nat Rev Mol Cell Biol. 2013;14(11):699–712. doi:10.1038/nrm3679.
13. Nagano T, Fraser P. No-nonsense functions for long noncoding RNAs. Cell. 2011;145(2):178–81. doi:10.1016/j.cell.2011.03.014.
14. Lee JT. Epigenetic regulation by long noncoding RNAs. Science. 2012;338(6113):1435–9. doi:10.1126/science.1231776.
15. Cheetham SW, Gruhl F, Mattick JS, Dinger ME. Long noncoding RNAs and the genetics of cancer. Br J Cancer. 2013;108(12):2419–25. doi:10.1038/bjc.2013.233.
16. Nissan A, Stojadinovic A, Mitrani-Rosenbaum S, Halle D, Grinbaum R, Roistacher M, et al. Colon cancer associated transcript-1: a novel RNA expressed in malignant and pre-malignant human tissues. Int J Cancer. 2012;130(7):1598–606. doi:10.1002/ijc.26170.
17. Yeager M, Orr N, Hayes RB, Jacobs KB, Kraft P, Wacholder S, et al. Genome-wide association study of prostate cancer identifies a second risk locus at 8q24. Nat Genet. 2007;39(5):645–9. doi:10.1038/ng2022.
18. Zanke BW, Greenwood CM, Rangrej J, Kustra R, Tenesa A, Farrington SM, et al. Genome-wide association scan identifies a colorectal cancer susceptibility locus on chromosome 8q24. Nat Genet. 2007;39(8):989–94. doi:10.1038/ng2089.
19. Chen H, Xu J, Hong J, Tang R, Zhang X, Fang JY. Long noncoding RNA profiles identify five distinct molecular subtypes of colorectal cancer with clinical relevance. Mol Oncol. 2014. doi:10.1016/j.molonc.2014.05.010.
20. Zhu YP, Bian XJ, Ye DW, Yao XD, Zhang SL, Dai B, et al. Long noncoding RNA expression signatures of bladder cancer revealed by microarray. Oncol Lett. 2014;7(4):1197–202. doi:10.3892/ol.2014.1843.
21. Wu ZH, Wang XL, Tang HM, Jiang T, Chen J, Lu S, et al. Long noncoding RNA HOTAIR is a powerful predictor of metastasis and poor prognosis and is associated with epithelial-mesenchymal transition in colon cancer. Oncol Rep. 2014;32(1):395–402. doi:10.3892/or.2014.3186.
22. Alaiyan B, Ilyayev N, Stojadinovic A, Izadjoo M, Roistacher M, Pavlov V, et al. Differential expression of colon cancer associated transcript1 (CCAT1) along the colonic adenoma-carcinoma sequence. BMC Cancer. 2013;13(1):196. doi:10.1186/1471-2407-13-196.
23. Xiang JF, Yin QF, Chen T, Zhang Y, Zhang XO, Wu Z, et al. Human colorectal cancer-specific CCAT1-L lncRNA regulates long-range chromatin interactions at the MYC locus. Cell Res. 2014;24(5):513–31. doi:10.1038/cr.2014.35.
24. Yang F, Xue X, Bi J, Zheng L, Zhi K, Gu Y, et al. Long noncoding RNA CCAT1, which could be activated by c-Myc, promotes the progression of gastric carcinoma. J Cancer Res Clin Oncol. 2013;139(3):437–45. doi:10.1007/s00432-012-1324-x.
25. Huarte M, Guttman M, Feldser D, Garber M, Koziol MJ, Kenzelmann-Broz D, et al. A large intergenic noncoding RNA induced by p53 mediates global gene repression in the p53 response. Cell. 2010;142(3):409–19. doi:10.1016/j.cell.2010.06.040.
26. Feldstein O, Nizri T, Doniger T, Jacob J, Rechavi G, Ginsberg D. The long non-coding RNA ERIC is regulated by E2F and modulates the cellular response to DNA damage. Mol Cancer. 2013;12(1):131. doi:10.1186/1476-4598-12-131.
27. Sun M, Liu XH, Lu KH, Nie FQ, Xia R, Kong R, et al. EZH2-mediated epigenetic suppression of long noncoding RNA SPRY4-IT1 promotes NSCLC cell proliferation and metastasis by affecting the epithelial-mesenchymal transition. Cell Death Dis. 2014;5:e1298. doi:10.1038/cddis.2014.256.
