# Supplementary material for: Expression of Concern: Prognostic value of long non-coding RNA CCAT1 expression in patients with cancer: A meta-analysis
Source: PLoS One. 2023 Apr 20;18(4):e0284940. doi: 10.1371/journal.pone.0284940 (PMC10118116; doi:10.1371/journal.pone.0284940)
Supplement: S1 File — (ZIP) [file pone.0284940.s001.zip › 3 The 11 included studies in PDF/5.pdf]

Environmental and Molecular Mutagenesis 57:508–515 (2016)

Research Article

The rs6983267 SNP and Long Non-Coding RNA *CARLo-5* Are Associated With Endometrial Carcinoma

Xiwa Zhao,[1] Xurui Wei,[1] Lianmei Zhao,[2] Li Shi,[1] Jianxin Cheng,[1]* Shan Kang,[1] Hui Zhang,[1] Jun Zhang,[1] Li Li,[1] Haibo Zhang,[1] and Wei Zhao[1]

[1]*Department of Obstetrics and Gynecology, Fourth Hospital, Hebei Medical University, Shijiazhuang, China*

[2]*Tumor Research Institute, Fourth Hospital, Hebei Medical University, Shijiazhuang, China*

The single nucleotide polymorphism (SNP) rs6983267 and cancer-associated region long non-coding RNA (*CARLo-5*) are associated with various human cancers. This study aimed to investigate the expression of *CARLo-5* in endometrial carcinoma (EC) and its relationship with clinicopathological features and patient survival. The association of the rs6983267 SNP with EC risk and its involvement in the regulation of *CARLo-5* expression in EC were investigated. The rs6983267 SNP was genotyped by polymerase chain reaction (PCR) and ligase detection reaction in 543 EC patients and 584 controls. The expression of *CARLo-5* in 108 EC tissues and 66 normal endometrial tissues (NETs) was determined using quantitative real-time PCR. The genotype and allele distributions of the rs6983267 SNP differed significantly between patients and controls. There was a significant correlation between the rs6983267 genotypes and lymph node metastasis of EC patients ($P = 0.026$). *CARLo-5* expression was significantly higher in EC tissues than in NETs ($P < 0.001$) and significantly associated with FIGO stage ($P = 0.029$) and lymph node metastasis ($P = 0.030$). Patients with high *CARLo-5* expression had significantly shorter overall survival than those with low *CARLo-5* expression ($P = 0.003$). The rs6983267 genotype was significantly correlated with *CARLo-5* expression ($P < 0.05$). In conclusion, *CARLo-5* was identified as a pro-oncogenic lncRNA that may play an important role in EC progression and represent a prognostic marker for EC. The expression of *CARLo-5* was significantly correlated with the rs6983267 genotype associated with increased susceptibility to EC. Environ. Mol. Mutagen. 57:508–515, 2016. © 2016 Wiley Periodicals, Inc.

Key words: endometrial carcinoma; rs6983267; CARLo-5; susceptibility; prognosis

INTRODUCTION

Endometrial carcinoma (EC) is one of the most common cancers of the female reproductive system and the third most frequent cause of gynecologic cancer-related death. The morbidity and mortality of EC are increasing despite advances in diagnostic techniques, surgical management, and chemoradiotherapy, and a large number of EC patients have a poor prognosis [Vale et al., 2012; Siegel et al., 2013]. Furthermore, although our understanding of the molecular mechanisms underlying EC carcinogenesis has improved, there is currently no molecular biomarker that is universally used for the identification of patients with EC [Slomovitz and Coleman, 2012]. Therefore, elucidating the molecular mechanisms underlying the progression of EC is essential for the development of effective therapies and the identification of diagnostic and prognostic biomarkers for patients with EC.

Several previous studies showed a significant association between the G allele of rs6983267, a single nucleotide polymorphism (SNP) at 8q24.21, and increased risk of various types of cancer, such as colorectal and prostate cancers [Haiman et al., 2007; Tomlinson et al., 2007; Sahasrabudhe et al., 2015]. However, the role of rs6983267 in EC has not been reported to date. In addition, the biological function of

*Correspondence to: Jianxin Cheng, Department of Obstetrics and Gynecology, Fourth Hospital, Hebei Medical University, Jiankanglu 12, Shijiazhuang 050011, Hebei, People's Republic of China. E-mail: jxcheng2014@163.com

Received 28 February 2016; provisionally accepted 14 June 2016; and in final form 17 June 2016

DOI 10.1002/em.22031

Published online 19 July 2016 in Wiley Online Library (wileyonlinelibrary.com).

© 2016 Wiley Periodicals, Inc.

the rs6983267 SNP remains unclear because it is located in a gene desert that does not contain protein-coding genes. Several studies reported a correlation between rs6983267 and proto-oncogene MYC expression; however, there is little agreement among the different studies [Prokunina-Olsson and Hall, 2009; Takatsuno et al., 2013].

Genome sequencing projects revealed that protein non-coding sequences account for the majority of the human genome, whereas protein-coding genes account for ~2% of the genome [Shi et al., 2013]. Long non-coding RNAs (lncRNAs), which are non-protein-coding RNA transcripts longer than 200 nucleotides, recently attracted interest because of their involvement in various cellular processes, development, and human diseases [Fatica and Bozzoni, 2014]. Increasing evidence indicates that lncRNAs play vital roles in cancer biology, and they are regarded as drivers of tumor suppressive and oncogenic functions in various types of cancer [Gibb et al., 2011; He et al., 2014; Chen et al., 2015; Luo et al., 2015].

Cancer-associated region lncRNA (*CARLo-5*), a recently identified lncRNA located in the 8q24.21 region, has been investigated in numerous solid tumors, including colon [Kim et al., 2014], gastric [Zhang et al., 2014], and lung cancers [Luo et al., 2014]. In these tumors, *CARLo-5* is upregulated and can serve as a modular scaffold. The oncogenic SNP rs6983267 in the MYC enhancer region regulates *CARLo-5* expression through long-range interaction with the active regulatory region of its promoter [Kim et al., 2014]. However, to the best of our knowledge, the role and molecular mechanism of *CARLo-5* in EC have not been reported to date.

The objective of the present study was to investigate the expression of *CARLo-5* in EC and its relationship with the carcinoma's clinicopathological features and patient survival. The association of the rs6983267 SNP with the risk of EC and its potential involvement in the regulation of *CARLo-5* expression in EC were also investigated.

MATERIALS AND METHODS

Study Participants

Blood was obtained from the following two groups and DNA was extracted for genotyping: (1) 584 female controls and (2) 543 EC patients treated at the Fourth Hospital, Hebei Medical University between 2010 and 2015. All patients underwent an initial hysterectomy and were pathologically diagnosed with EC. Pathological diagnoses were performed by the Department of Pathology of the same hospital according to the classification system of the International Federation of Gynecology and Obstetrics (FIGO 2009). The clinical information of the 543 EC patients and its relationship with clinicopathological features are listed in Table II. None of the patients enrolled in this study received preoperative radiotherapy, chemotherapy, or hormone therapy. The control group consisted of 584 women without any malignant disease confirmed by surgical exploration and with normal endometrial tissues (NETs) pathologically confirmed after hysterectomy for benign diseases, such as myoma or adenomyosis. The mean ages of patients and controls were 56 years (range, 29–70 years) and 53 years (range, 31–67 years), respectively. There was no statistically significant difference in age distribution between the two groups ($P > 0.05$). All subjects were women of the Han nationality in Northern China. The study was approved by the ethics committee of Hebei Cancer Institute, and informed consent was obtained from all recruited subjects.

Of the above-mentioned study subjects (247 EC patients and 233 controls) selected between 2010 and 2012, 108 EC patients and 66 controls were selected for subsequent experiments on the basis of fresh-frozen tissues (EC and NETs) collected during hysterectomy for EC and benign diseases, respectively. Following surgical excision, all EC samples and NETs were immediately frozen in liquid nitrogen and stored at −80°C until RNA extraction. The clinical information on the 108 EC patients is listed in Table II. Overall survival was defined as the interval between the date of surgery and death.

DNA Extraction and Genotyping

Venous blood (5 mL) was collected from patients and controls in Vacutainer tubes containing EDTA and stored at 4°C. Genomic DNA was extracted within 1 week after sampling using proteinase K digestion followed by a salting out procedure according to a previously described method [Miller et al., 1988]. Genotypes of rs6983267 SNP were analyzed by the Shanghai Generay Biotech Co., Ltd. (http://www.generay.com.cn) using the polymerase chain reaction (PCR)/ligase detection reaction method. After the PCR/ligase detection reaction, the products were analyzed using an ABI 3730XL DNA sequencer (Applied Biosystems, CA). To confirm the accuracy of this method, randomly selected PCR products were subjected to direct DNA sequencing, and the reproducibility was 100%.

RNA Extraction and Quantitative Real-Time PCR

Total RNA from tissues was extracted using the TRIzol reagent (Invitrogen, CA) according to the manufacturer's protocol. RNA (2 μg) was reverse transcribed into cDNA using the Primer-Script™ one step real-time PCR kit from Promega Corp (Madison, WI). The cDNA template was amplified by real-time PCR using the SYBR® Premix Dimmer Eraser kit (TaKaRa, Dalian, China). The quantitative real-time PCR was performed on an ABI 7500 system (Applied Biosystems, CA) according to the manufacturer's instructions. The amplified fragments were detected on a 1.5% (w/v) agarose gel and analyzed using an IS1000 image analysis system (Alpha Innotech, San Leandro, CA). The level of *CARLo-5* expression in each sample was normalized to the respective GAPDH expression level. The primer sequences were synthesized by Sangon Biotech Co., Ltd (Shanghai, China) and were as follows: GAPDH, 5′-GTCAACGGATTTGGTCTGTATT-3′ (forward), 5′-AGTCT TCTGGGTGGCAGTGAT-3′ (reverse); and *CARLo-5*, 5′-GCCACAAA TCAACAACAACAACAACAA-3′ (forward), 5′-AGAGTGATGCCAAG GCTGTTATTGTCAA-3′ (reverse). The real-time PCR reaction was performed under the following conditions: 95°C for 5 min, 40 cycles of 95°C for 15 s, and 60°C for 60 s. The specificity of each PCR reaction was confirmed by melting curve analyses. The comparative Ct method (ΔΔCt) was used for quantification of gene expression. The relative expression amount of *CARLo-5* to GAPDH was calculated using the equation $2^{-\Delta\Delta Ct}$, where $\Delta Ct = Ct\ CARLo{-}5 - Ct$ GAPDH. The gene expression levels of *CARLo-5* were compared between tumors and normal tissues. To minimize experimental variability, each sample was analyzed in triplicate and the mean expression level was calculated.

Statistical Analysis

Statistical analysis was performed using the SPSS17.0 software package (SPSS Company, Chicago, Illinois, USA). Hardy-Weinberg analysis was performed to compare the observed and expected genotype frequencies using the χ^2 test. Comparison of the genotype distributions in the

TABLE I. Association of the rs6983267 SNP With the Risk of Developing Endometrial Carcinoma

SNP	Controls, *n* (%)	Patients, *n* (%)	*P* value	OR (95% CI)
Genotypes				
GG	127 (21.8)	148 (27.2)	0.021	1.00
GT	291 (49.8)	274 (50.5)		0.81 (0.61–1.08)
TT	166 (28.4)	121 (22.3)		0.63 (0.45–0.87)
GT+TT	457 (78.2)	395 (72.8)		0.74 (0.57–0.97)
Alleles				
G	545 (46.7)	570 (52.5)	0.006	1.00
T	623 (53.3)	516 (47.5)		0.79 (0.67–0.93)

patients and controls was performed using the χ^2 test. The odds ratio (OR) and 95% confidence interval (CI) were calculated using an unconditional logistic regression model. The comparison of *CARLo-5* expression between two groups was performed using the Student's *t* test, whereas the association of the rs6983267 genotypes and *CARLo-5* expression with clinicopathological features was evaluated using Pearson's $\chi 2$ test. Survival curves were plotted by the Kaplan-Meier method, and the log-rank test was used to assess differences between stratified survival groups using the median value as the cutoff. A two-sided *P* value of <0.05 was considered to be statistically significant.

RESULTS

Association of the rs6983267 SNP With Susceptibility to EC

The distribution of the rs6983267 genotype in the control group did not significantly deviate from Hardy-Weinberg equilibrium ($P > 0.05$). The genotype and allele frequencies for the rs6983267 SNP are summarized in Table I. The genotype and allele distributions of the rs6983267 SNP differed significantly between patients and controls. The frequency of the *G* allele in patients (52.5%) was significantly higher than that in the controls (46.7%) ($P = 0.006$). The frequencies of the *GG*, *GT*, and *TT* genotypes in patients were significantly different from those in the controls ($P = 0.021$). Women carrying the *T* allele (*GT* + *TT* genotype) had a lower risk of developing EC than those carrying the *GG* genotype (OR = 0.74; 95% CI = 0.57–0.97).

Association between the rs6983267 Genotypes and Clinicopathological Features

To determine the clinical relevance of the rs6983267 genotypes in EC, the association between the rs6983267 genotypes and clinicopathological features such as age, histology, FIGO stage, histological grade, myometrial invasion, lymphovascular space invasion, and lymph node metastasis was examined. The rs6983267 genotypes were not associated with age, histology, FIGO stage, histological grade, myometrial invasion and lymphovascular space invasion, but there was a significant correlation between the distribution of genotype frequency and lymph node metastasis (Table II, $P = 0.026$).

Overexpression of *CARLo-5* in EC Tissues

The expression levels of *CARLo-5* were examined in 108 EC samples and 66 NETs by real-time PCR. *CARLo-5* expression was significantly higher in EC tissues than in NETs (Figs. 1A and 1B, $P < 0.001$), indicating that *CARLo-5* was frequently upregulated in EC.

Association between *CARLo-5* Expression Levels and Clinicopathological Features

To determine the clinical relevance of *CARLo-5* expression in EC, the association between *CARLo-5* expression and clinicopathological features such as age, histology, FIGO stage, histological grade, myometrial invasion, lymphovascular space invasion, and lymph node metastasis was examined. The median level of *CARLo-5* expression in EC tissues was selected as the cutoff point and used to assign the 108 patients with EC to the high *CARLo-5* group (n = 54) or the low *CARLo-5* group (n = 54). As shown in Table II, high *CARLo-5* expression was associated with advanced FIGO stage (Fig. 1C, $P = 0.029$) and lymph node metastasis (Fig. 1D, $P = 0.030$). However, *CARLo-5* expression was not associated with other features such as age, histology, histological grade, myometrial invasion and lymphovascular space invasion (Table II).

Association between *CARLo-5* Expression Levels and Prognosis of EC Patients

Kaplan-Meier survival analysis and log-rank tests using patient postoperative survival were performed to further evaluate the correlation between *CARLo-5* expression and the prognosis of EC patients. The overall survival curves in the high and low *CARLo-5* groups are shown in Figure 2. Patients with high *CARLo-5* expression showed significantly poorer overall survival than those with low *CARLo-5* expression ($P = 0.003$). These results indicate that *CARLo-5* may play an important role in the progression of EC.

Correlation between *CARLo-5* Expression and Genotypes of rs6983267

To determine whether the rs6983267 SNP is involved in the regulation of *CARLo-5* expression, *CARLo-5* expression levels were assessed in 108 EC tissues classified according to the three genotypes of rs6983267. As shown in Figures 3A and 3B, there was a significant correlation between rs6983267 genotypes and *CARLo-5* expression levels ($P < 0.05$). The expression level of *CARLo-5* was highest in EC tissues with the *GG* genotype ($n = 34$), followed by the *GT* and *TT* genotypes ($n = 51$ and 23, respectively).

TABLE II. Association of the rs6983267 Genotypes and *CARLo-5* Expression With Clinicopathological Features

	Genotype			*CARLo-5* expression		
Clinicopathological features	*GG* (%)	*GT+TT* (%)	*P*	High (%)	Low (%)	*P*
Age (years)						
≥55	92 (28.5)	231 (71.5)	0.437	37 (55.2)	30 (44.8)	0.165
<55	56 (25.4)	164 (74.6)		17 (41.5)	24 (58.5)	
Histology						
Endometrioid	125 (27.1)	336 (72.9)	0.861	45 (47.4)	50 (52.6)	0.139
Non-endometrioid	23 (28.0)	59 (72.0)		9 (69.2)	4 (30.8)	
FIGO stage						
I–II	117 (26.4)	327 (73.6)	0.316	39 (44.8)	48 (55.2)	0.029
III–IV	31 (31.3)	68 (68.7)		15 (71.4)	6 (28.6)	
Histological grade						
G1	35 (21.0)	132 (79.0)	0.082	13 (39.4)	20 (60.6)	0.200
G2	86 (30.6)	195 (69.4)		31 (51.7)	29 (48.3)	
G3	27 (28.4)	68 (71.6)		10 (66.7)	5 (33.3)	
Myometrial invasion						
<50%	84 (24.5)	259 (75.5)	0.058	29 (44.6)	36 (55.4)	0.169
≥50%	64 (32.0)	136 (68.0)		25 (58.1)	18 (41.9)	
Lymphovascular space invasion						
Positive	29 (31.5)	63 (68.5)	0.313	10 (62.5)	6 (37.5)	0.279
Negative	119 (26.4)	332 (73.6)		44 (47.8)	48 (52.2)	
Lymph node metastasis						
Positive	35 (36.5)	61 (63.5)	0.026	12 (75.0)	4 (25.0)	0.030
Negative	113 (25.3)	334 (74.7)		42 (45.7)	50 (54.3)	

These results suggest that the rs6983267 SNP plays a role in the regulation of *CARLo-5* expression.

DISCUSSION

Despite recent advances in diagnostic techniques and treatment modalities, EC remains associated with a poor prognosis. Although the involvement of oncogenes and tumor suppressor genes has been suggested, the molecular mechanism of EC progression remains unclear. Further investigation of the pathogenesis of EC and the identification of accurate prognostic markers are vital to improve the prognosis of EC patients. In the present study, we identified a significant association between the *G* allele of the rs6983267 SNP and increased risk of developing EC. In addition, we found that *CARLo-5* levels were higher in EC tissues than in NETs, and *CARLo-5* upregulation was associated with advanced FIGO stage, lymph node metastasis, and poor prognosis. In addition, a significant correlation between rs6983267 genotypes and the expression of *CARLo-5* was detected. To the best of our knowledge, this is the first molecular epidemiologic study investigating the association of the rs6983267 SNP and *CARLo-5* with EC.

LncRNAs, initially thought to represent spurious transcriptional noise, are emerging as new regulators in the cancer paradigm [Nagano and Fraser, 2011]. LncRNAs regulate the expression of genes at the epigenetic, transcriptional, and post-transcriptional levels, and play an important role in physiological processes [Krishnan and Mishra, 2014]. Accumulating evidence indicates that lncRNAs may play complex and extensive roles in promoting the development and progression of cancer [Huarte et al., 2010; Gibb et al., 2011; Sahu et al., 2015], and recent studies showed that lncRNAs play an important role in the development of EC [He et al., 2014, Huang et al., 2014; Zhai et al., 2015]. For example, HOTAIR, a well-characterized lncRNA, is upregulated in EC and correlates with poor prognosis [He et al., 2014]. However, a small number of lncRNAs have been characterized and their role in cancer remains to be explored.

As a new molecule in the lncRNA field, *CARLo-5* was initially identified because of its upregulation in colon cancer, wherein *CARLo-5* functions in cell-cycle regulation and tumor development [Kim et al., 2014]. In addition, the expression of *CARLo-5* is significantly correlated with the rs6983267 allele associated with increased cancer susceptibility. On the basis of these findings, we investigated the biological role of the rs6983267 SNP and *CARLo-5* in EC progression, and analyzed the potential involvement of the rs6983267 SNP in the regulation of *CARLo-5* expression in EC.

In the present study, we investigated, for the first time, the expression pattern of *CARLo-5* in EC tissues and analyzed the clinical significance of *CARLo-5* in patients with EC. We found that the expression of *CARLo-5* was dramatically upregulated in EC tissues compared with its expression in NETs, which was consistent with recent

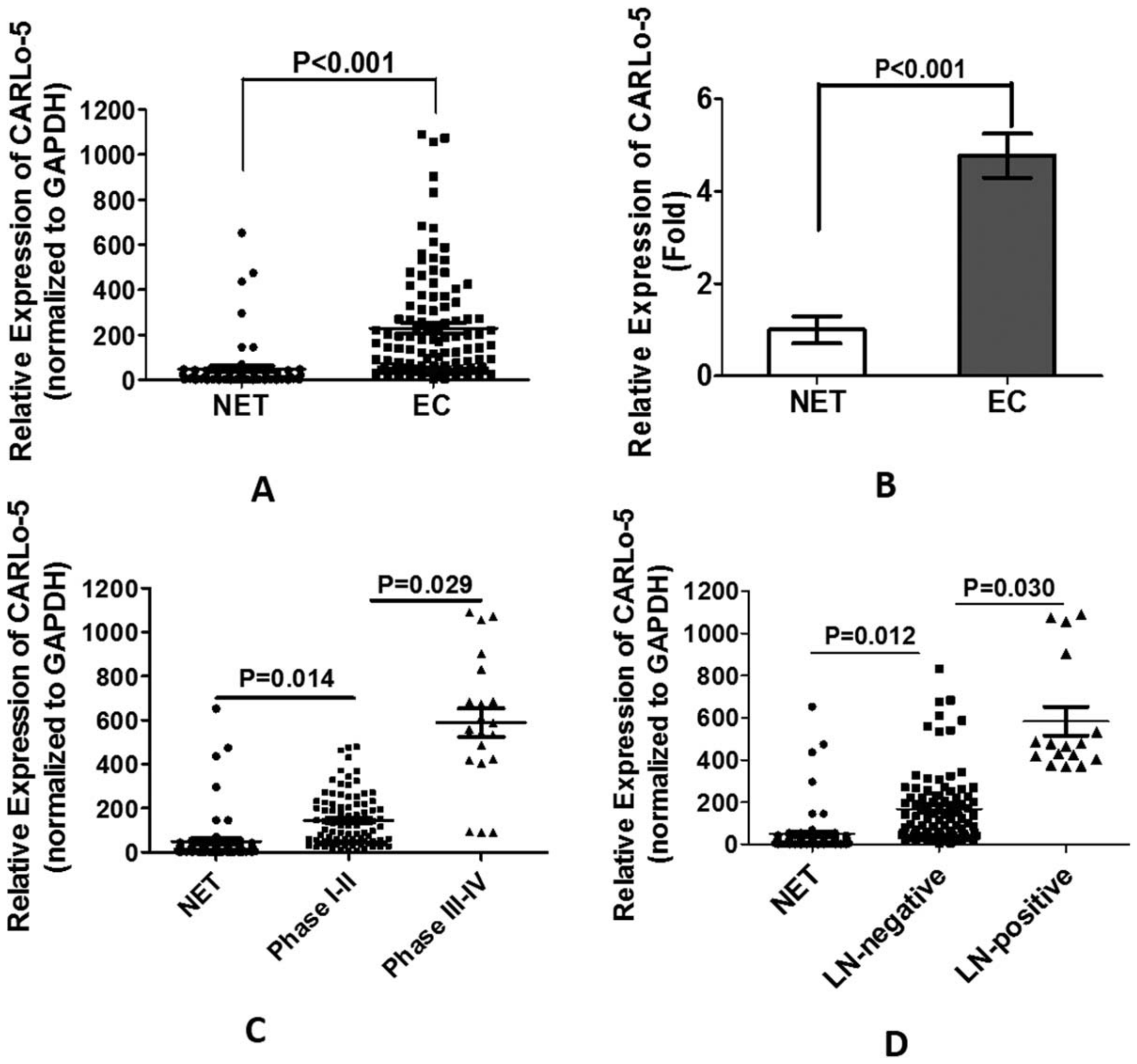

Fig. 1. *CARLo-5* expression and clinicopathological features. **A**: *CARLo-5* expression was significantly upregulated in endometrial carcinoma (EC) tissues compared with normal endometrial tissues (NETs). **B**: Difference in expression levels of *CARLo-5* between EC tissues and NETs. The fold change was 4.77, and the expression of *CARLo-5* was normalized to GAPDH. **C**: *CARLo-5* expression was significantly higher in patients with advanced FIGO stages (phases III–IV) ($P = 0.029$). **D**: *CARLo-5* expression was significantly higher in patients with lymph node metastasis (LN-positive) than in patients with non-lymph node metastasis (LN-negative) ($P = 0.030$).

studies showing the upregulation of *CARLo-5* in a variety of cancers, such as colon, gastric, and lung cancers [Kim et al., 2014; Luo et al., 2014; Zhang et al., 2014]. Our results also revealed that the upregulation of *CARLo-5* expression was associated with advanced FIGO stage and lymph node metastasis, indicating that *CARLo-5* overexpression might play an important role in EC progression. Furthermore, high expression of *CARLo-5* was correlated with poor survival. These findings were consistent with those of a study showing that *CARLo-5* upregulation was correlated with advanced FIGO stage, lymph node metastasis, and poor prognosis of patients with non-small cell lung cancer [Luo et al., 2014]. These findings indicated that *CARLo-5* may exhibit oncogenic activity and play an important role in the modulation of EC progression.

The rs6983267 at 8q24.21 has been established as a significant cancer-related SNP, as it shows a strong association with carcinogenesis [Ishimaru et al., 2012]. A previous study reported that the homozygous *G* allele of rs6983267 is associated with a high risk of colorectal cancer, while the homozygous T allele is a non-risk allele [Ishimaru et al., 2012]. Furthermore, several other studies showed that the rs6983267 SNP is related to increased risk in various types of cancer, such as breast, bladder, ovarian, and prostate cancers in several populations [Haiman et al., 2007; Wokolorczyk et al., 2008]. These previous findings were consistent with those of the present study. The frequency of the *G* allele of rs6983267 was significantly higher in EC patients (53.4%) than in the controls (45.3%). Women carrying the *G* allele of rs6983267 had a

high risk of developing EC. These findings suggest that the rs6983267 SNP is a multicancer susceptibility marker. Furthermore, this study showed that there was a significant correlation between the distribution of genotype frequency and lymph node metastasis, and EC patients with the *GG* genotype might have a high risk of lymph node metastasis.

Although rs6983267 at 8q24.21 is considered a significant cancer-associated SNP, the biological function of rs6983267 remains unclear because of the lack of protein-coding genes at 8q24.21. Several studies demonstrated a long-range interaction between the cancer-associated variant rs6983267 and MYC expression [Pomerantz et al., 2009; Takatsuno et al., 2013]. TCF4/LEF binding to the homozygous *G* allele of rs6983267 promotes MYC transcription; no direct binding is observed in patients with the homozygous T allele [Tuupanen et al., 2008; Tuupanen et al., 2009]. However, other studies found no clear correlation between the rs6983267 SNP and MYC expression [Prokunina-Olsson and Hall, 2009; Sur et al., 2012]. Further studies are required to verify whether MYC is involved in the function of the rs6983267 SNP and its role in cancer susceptibility. A previous study showed a strong association between the cancer-associated variant rs6983267 in the MYC enhancer region and *CARLo-5* expression [Kim et al., 2014]. Moreover, the physical interaction between the MYC enhancer and the active regulatory region of the *CARLo-5* promoter provides strong evidence of the mechanism of the association [Kim et al., 2014]. These results are consistent with the findings of the present study showing that the expression level of *CARLo-5* was highest in EC tissues with the *GG* genotype of rs6983267 and lowest in EC tissues with the *TT* genotype of rs6983267. The cancer-associated variant rs6983267 in the MYC enhancer region may regulate the expression of *CARLo-5* through long-range interaction with the active regulatory region of its promoter, which further promotes progression and worsens the prognosis of EC.

In conclusion, the present study reported for the first time that the rs6983267 variant at chromosome 8q24 is a cancer-associated SNP in EC. Our study indicated that the rs6983267 SNP may regulate the expression of *CARLo-5*, which could function as a pro-oncogenic lncRNA with an important role in EC progression and

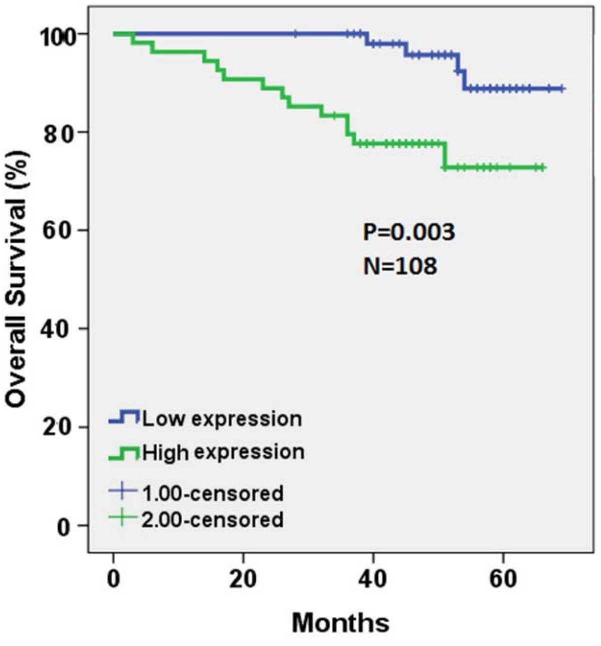

Fig. 2. Association between *CARLo-5* expression level and the prognosis of endometrial carcinoma patients. Kaplan-Meier survival curves show that high expression of *CARLo-5* was significantly associated with a poor overall survival in 108 endometrial carcinoma patients ($P = 0.003$).

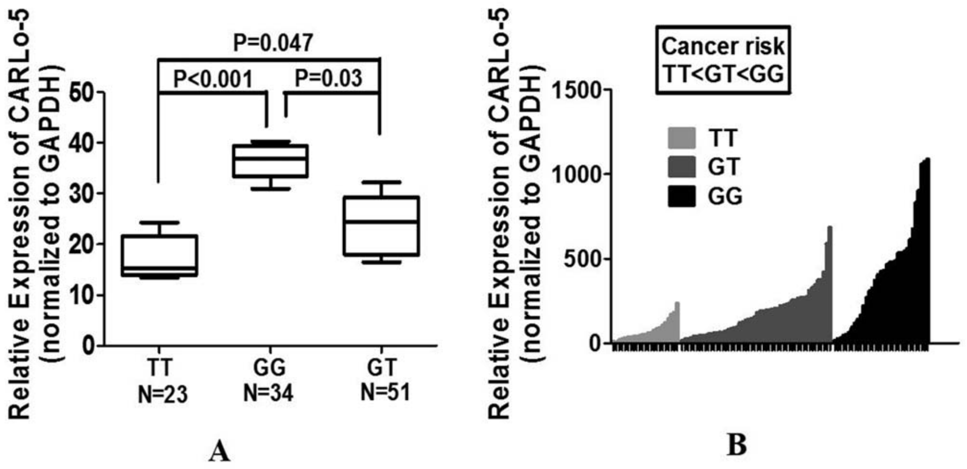

Fig. 3. Correlation between *CARLo-5* expression and genotypes of rs6983267. **A**: Box-and-whisker plot showing differential expression levels of *CARLo-5* in endometrial carcinoma tissues classified by the three genotypes of rs6983267. **B**: Relative expression levels of *CARLo-5* in 108 endometrial carcinoma tissues classified by the three genotypes of rs6983267.

represent a prognostic marker for EC. This study contributes to our understanding of the molecular mechanisms of EC by clarifying the association of the rs6983267 SNP and *CARLo-5* with EC. However, it is unclear whether the rs6983267 SNP or *CARLo-5* can be used as a universal molecular biomarker for the identification of patients with EC on the basis of the findings of the present study. Thus, the underlying molecular mechanisms of the rs6983267 SNP and *CARLo-5* and their involvement in EC need to be further studied.

ACKNOWLEDGMENTS

The authors greatly acknowledge several doctors in the Department of Obstetrics and Gynecology, the Fourth Affiliated Hospital of Hebei Medical University, China, for their assistance in recruiting study subjects.

AUTHOR CONTRIBUTIONS

Xiwa Zhao, Jianxin Cheng, and Shan Kang designed the study and applied for Research Ethics Board approval. Li Shi, Hui Zhang, and Wei Zhao recruited the patients and collected the data. Jun Zhang, Li Li, and Haibo Zhang conducted the experiments. Xurui Wei and Lianmei Zhao analyzed the data and prepared draft figures and tables. Xiwa Zhao drafted and completed the manuscript. All authors approved the final manuscript.

REFERENCES

Chen X, Liu L, Zhu W. 2015. Up-regulation of long non-coding RNA CCAT2 correlates with tumor metastasis and poor prognosis in cervical squamous cell cancer patients. Int J Clin Exp Pathol 8: 13261–13266.

Fatica A, Bozzoni I. 2014. Long non-coding RNAs: New players in cell differentiation and development. Nat Rev Genet 15:7–21.

Gibb EA, Brown CJ, Lam WL. 2011. The functional role of long non-coding RNA in human carcinomas. Mol Cancer 10:38

Haiman CA, Le Marchand L, Yamamato J, Stram DO, Sheng X, Kolonel LN, Wu AH, Reich D, Henderson BE. 2007. A common genetic risk factor for colorectal and prostate cancer. Nat Genet 39:954–956.

He X, Bao W, Li X, Chen Z, Che Q, Wang H, Wan XP. 2014. The long non-coding RNA HOTAIR is upregulated in endometrial carcinoma and correlates with poor prognosis. Int J Mol Med 33:325–332.

Huang J, Ke P, Guo L, Wang W, Tan H, Liang Y, Yao S. 2014. Lentivirus-mediated RNA interference targeting the long noncoding RNA HOTAIR inhibits proliferation and invasion of endometrial carcinoma cells in vitro and in vivo. Int J Gynecol Cancer 24: 635–642.

Huarte M, Guttman M, Feldser D, Garber M, Koziol MJ, Kenzelmann-Broz D, Khalil AM, Zuk O, Amit I, Rabani M, Attardi LD, Regev A, et al. 2010. A large intergenic noncoding RNA induced by p53 mediates global gene repression in the p53 response. Cell 142:409–419.

Ishimaru S, Mimori K, Yamamoto K, Inoue H, Imoto S, Kawano S, Yamaguchi R, Sato T, Toh H, Iinuma H, Suzuki S, Tokudome S, et al. 2012. Increased risk for CRC in diabetic patients with the nonrisk allele of SNPs at 8q24. Ann Surg Oncol 19:2853–2858.

Kim T, Cui R, Jeon YJ, Lee JH, Lee JH, Sim H, Park JK, Fadda P, Tili E, Nakanishi H, Huh MI, Kim SH, et al. 2014. Long-range interaction and correlation between MYC enhancer and oncogenic long noncoding RNA CARLo-5. Proc Natl Acad Sci USA 111: 4173–4178.

Krishnan J, Mishra RK. 2014. Emerging trends of long non-coding RNAs in gene activation. Febs J 281:34–45.

Luo G, Wang M, Wu X, Tao D, Xiao X, Wang L, Min F, Zeng F, Jiang G. 2015. Long non-coding RNA MEG3 inhibits cell proliferation and induces apoptosis in prostate cancer. Cell Physiol Biochem 37:2209–2220.

Luo J, Tang L, Zhang J, Ni J, Zhang HP, Zhang L, Xu JF, Zheng D. 2014. Long non-coding RNA CARLo-5 is a negative prognostic factor and exhibits tumor pro-oncogenic activity in non-small cell lung cancer. Tumor Biol 35:11541–11549.

Miller SA, Dybes DD, Polesky HF. 1988. A simple salting out procedure for extracting DNA from human nucleated cells. Nucleic Acid Res 16:1215

Nagano T, Fraser P. 2011. No-nonsense functions for long noncoding RNAs. Cell 145:178–181.

Pomerantz MM, Ahmadiyeh N, Jia L, Herman P, Verzi MP, Doddapaneni H, Beckwith CA, Chan JA, Hills A, Davis M, Yao K, Kehoe SM, et al. 2009. The 8q24 cancer risk variant rs6983267 shows long-range interaction with MYC in colorectal cancer. Nat Genet 41:882–884.

Prokunina-Olsson L, Hall JL. 2009. No effect of cancer-associated SNP rs6983267 in the 8q24 region on co-expression of MYC and TCF7L2 in normal colon tissue. Mol Cancer 8:96–100.

Sahasrabudhe R, Estrada A, Lott P, Martin L, Polanco Echeverry G, Velez A, Neta G, Takahasi M, Saenko V, Mitsutake N, JTCMS Consortium, Jaeguer E, et al. 2015. The 8q24 rs6983267G variant is associated with increased thyroid cancer risk. Endocr Relat Cancer 22:841–849.

Sahu A, Singhal U, Chinnaiyan AM. 2015. Long noncoding RNAs in cancer: From function to translation. Trends Cancer 1:93–109.

Shi X, Sun M, Liu H, Yao Y, Song Y. 2013. Long non-coding RNAs: a new frontier in the study of human diseases. Cancer Lett 339: 159–166.

Siegel R, Naishadham D, Jemal A. 2013. Cancer statistics, 2013. CA Cancer J Clin 63:11–30.

Slomovitz BM, Coleman RL. 2012. The PI3K/AKT/mTOR pathway as a therapeutic target in endometrial cancer. Clin Cancer Res 18: 5856–5864.

Sur IK, Hallikas O, Vaharautio A, Yan J, Turunen M, Enge M, Taipale M, Karhu A, Aaltonen LA, Taipale J. 2012. Mice lacking a Myc enhancer that includes human SNP rs6983267 are resistant to intestinal tumors. Science 338:1360–1363.

Takatsuno Y, Mimori K, Yamamoto K, Sato T, Niida A, Inoue H, Imoto S, Kawano S, Yamaguchi R, Toh H, Iinuma H, Ishimaru S, et al. 2013. The rs6983267 SNP Is associated with MYC transcription efficiency, which promotes progression and worsens prognosis of colorectal cancer. Ann Surg Oncol 20:1395–1402.

Tomlinson I, Webb E, Carvajal-Carmona L, Broderick P, Kemp Z, Spain S, Penegar S, Chandler I, Gorman M, Wood W, Barclay E, Lubbe S, et al. 2007. A genome-wide association scan of tag SNPs identifies a susceptibility variant for colorectal cancer at 8q24.21. Nat Genet 39:984–988.

Tuupanen S, Niittymaki I, Nousiainen K, Vanharanta S, Mecklin JP, Nuorva K, Jurvinen H, Hautaniemi S, Karhu A, Aaltonen LA. 2008. Allelic imbalance at rs6983267 suggests selection of the risk allele in somatic colorectal tumor evolution. Cancer Res 68: 14–17.

Tuupanen S, Turunen M, Lehtonen R, Hallikas O, Vanharanta S, Kivioja T, Bjorklund M, Wei G, Yan J, Niittymaki I, Mecklin JP, Järvinen H, et al. 2009. The common colorectal cancer

Environmental and Molecular Mutagenesis. DOI 10.1002/em

predisposition SNP rs6983267 at chromosome 8q24 confers potential to enhanced Wnt signaling. Nat Genet 41:885–890.

Vale CL, Tierney J, Bull SJ, Symonds PR. 2012. Chemotherapy for advanced, recurrent or metastatic endometrial carcinoma. Cochrane Database Syst Rev 8:CD003915

Wokolorczyk D, Gliniewicz B, Sikorski A, Zlowocka E, Masojc B, Debniak T, Matyjasik J, Mierzejewski M, Medrek K, Oszutowska D, Suchy J, Gronwald J, et al. 2008. A range of cancers is associated with the rs6983267 marker on chromosome 8. Cancer Res 68:9982–9986.

Zhai W, Li X, Wu S, Zhang Y, Pang H, Chen W. 2015. Microarray expression profile of lncRNAs and the upregulated ASLNC04080 lncRNA in human endometrial carcinoma. Int J Oncol 46:2125–2137.

Zhang Y, Ma M, Liu W, Ding W, Yu H. 2014. Enhanced expression of long noncoding RNA CARLo-5 is associated with the development of gastric cancer. Int J Clin Exp Pathol 7:8471–8479.
