# Supplementary material for: Expression of Concern: Prognostic value of long non-coding RNA CCAT1 expression in patients with cancer: A meta-analysis
Source: PLoS One. 2023 Apr 20;18(4):e0284940. doi: 10.1371/journal.pone.0284940 (PMC10118116; doi:10.1371/journal.pone.0284940)
Supplement: S1 File — (ZIP) [file pone.0284940.s001.zip › 3 The 11 included studies in PDF/6.pdf]

European Review for Medical and Pharmacological Sciences 2017; 21: 530-534

# Long non-coding RNA CARLo-5 upregulation associates with poor prognosis in patients suffering gastric cancer

J.-N. LIU[1], Y.-M. SHANGGUAN[2]

[1]Department of Thyroid Surgery, [2]Department of Outpatient; The Second Hospital of Shandong University, Jinan, Shandong, China

**Abstract.** – **OBJECTIVE:** Accumulating evidence has demonstrated that microRNAs (miRNAs) play critical roles in cancer initiation and development. The present study aims to explore the clinical significance of long noncoding RNA CARLo-5 (CARLo-5) in gastric cancer.

**PATIENTS AND METHODS:** CARLo-5 expression in normal gastric tissue, gastric cancer were quantified by Quantitative reverse transcription PCR. The relationship between CARLo-5 expression and the clinical features of gastric cancer was assessed. Kaplan-Meier method with log-rank test was applied to compare survival curves. The univariate and multivariate Cox regression models were employed to evaluate the prognostic value of the CARLo-5 expression in overall survival (OS) and relapse-free survival (RFS).

**RESULTS:** We observed that the relative expression of CARLo-5 in gastric cancer tissues was significantly higher than that of their matched adjacent normal tissues ($p < 0.001$). High CARLo-5 expression was found to be closely correlated with advanced T stage ($p = 0.003$), positive distant metastasis ($p = 0.009$), lymph node involvement ($p = 0.001$), and poor differentiation ($p = 0.001$). Furthermore, Kaplan-Meier analysis demonstrated that gastric cancer patients with high CARLo-5 expression had poorer OS ($p < 0.001$) and RFS ($p < 0.001$). Finally, univariate and multivariate Cox analysis indicated that high CARLo-5 expression were independent predictors for both OS and RFS.

**CONCLUSIONS:** Our findings implicated that CARLo-5 could be considered promising biomarkers for prognosis of gastric cancer.

*Key Words:*
Long noncoding RNA, CARLo-5, Overall survival, Relapse-free survival.

## Introduction

Cancer has become a major health problem and a large economic burden in the world[1]. Gastric cancer (GC) is one of the most common malignancies and the third leading cause of cancer-related deaths worldwide[2]. Despite advancements in the treatment of GC, the 5-year overall survival rate is still very low[3]. Early detection and treatment as well as blocking or slowing GC malignancy progression are necessary to improve GC prognosis.

Emerging evidence indicate that long noncoding RNAs (lncRNAs) is defined as transcripts with a minimum length of 200 nucleotides in size and limited protein-coding potential[4,5]. LncRNAs have recently gained significant attention in delineating the complex mechanisms underlying malignant processes[6]. More and more studies report the effect of lncRNA in tumors. For instance, Zhan et al[7] found that lncRNA PANDAR was significantly up-regulated in bladder cancer tissues and over expression of PANDAR promoted the proliferation/migration and suppressed apoptosis. Li et al[8] reported that knockdown of lncRNA could inhibit epithelial-mesenchymal transition (EMT) phenotype in glioma cells. Zhang et al[9] found high expression of lncRNA Sox2ot was a statistically significant risk factor affecting overall survival in gastric cancer patients. These results revealed that lncRNA may function not only as diagnostic and prognostic markers, but also as potential therapeutic targets of human tumors.

CARLo-5 was a newly identified lncRNA and its function in tumors remained largely unknown. The current study aimed to determine CARLo-5 expression in GC tissues and to explore the relationship between CARLo-5 level and clinicopathological features and patients survival.

## Patients and Methods

### *Ethics Statement*

The study was approved by the Institute of The Second Hospital of Shandong University, Jinan,



*Corresponding Author:* Yu-meng Shangguan, MD; e-mail: sggm19@163.com

Shandong, People's Republic of China. All specimens were handled and identified according to ethical and legal standards. Written informed consent was obtained from every patient to participate in this study.

### Patients and Tissue Samples

GC tissue samples and matched normal tissues were obtained from 240 patients at the Second Hospital of Shandong University between July 2008 and July 2011. None of the patients had received chemotherapy, radiotherapy, or endocrine therapy before surgery. The diagnosis was based on clinical examination and histopathological analysis of the tissue specimens. All tissue specimens were immediately snap-frozen in liquid nitrogen and then stored at -80°C. Clinical data of all the patients is listed in Table I.

### Quantitative Real-time PCR

Total RNA was extracted from cells or tissues using RNAisoPlus (Takara, Dalian, Niaoning, China), and was reverse-transcribed into cDNA using HiFiScript cDNA Kit (Invitrogen, Carlsbad, CA, USA). Its synthesis was conducted at 37 °C for 15 min, then 85 °C for 5 s according to the experimental protocols. Quantitative real-time PCR was performed with SYBR Green (Takara, Dalian, Niaoning, China) and the data collection were carried out on the Applied Biosystems® 7500 Real-Time PCR Systems (Thermo Fisher Scientific, Franklin, MA, USA). The primer sequences were as follows: GADPH: 5'-GTCAACGGATTTGGTCTGTATT-3' (forward), 5'-AGTCTTCT-GGGTGGCAGTGAT-3' (reverse); CARLo-5: 5'-GCCACAAATCAACAACAACAACAACAA-3' (forward), 5'-AGAGTGATGCCAAGGCTGTTATTGTCAA-3' (reverse).

CARLo-5 values were normalized to GAPDH. Comparative quantification was determined using the method of $2^{-\Delta\Delta CT}$.

### Statistical Analysis

Data are expressed as means ± standard errors of at least 3 independent experiments The significance of differences was analyzed using two-tailed Student's t-test using Prism 6 software (GraphPad Software, Inc., La Jolla, CA, USA). The Kaplan-Meier method was used to plot overall survival and relapse-free survival curves. The univariate and multivariate analyses were performed using log-rank tests and Cox's proportional hazard model, respectively. Differences were considered statistically significant when $p$ was less than 0.05.

## Results

### CARLo-5 is Upregulated in Human GC Tissues

We assessed CARLo-5 expression by qRT-PCR in the 240 GC tissues and matched adjacent normal tissues. The relative expression of CARLo-5 in GC tissues was significantly higher than that of their matched adjacent normal tissues ($p <$ 0.001, Figure 1).

### Correlations Between the Levels of CARLo-5 and the Clinicopathological Factors in GC Patients

According to the set cutoff value of 0.041, this cohort of 240 GC patients was divided into two groups: low CARLo-5 expression and high CARLo-5 expression. The clinicopathological parameters of CARLo-5 were showed in Table I. We found that high CARLo-5 expression was found to be closely correlated with advanced T stage ($p$ = 0.003), positive distant metastasis ($p$ = 0.009), lymph node involvement ($p$ = 0.001), and poor differentiation ($p$ = 0.001). However, CARLo-5 expression was not significantly related to gender, age, or tumor size (all $p > 0.05$) (Table I).

### Increased Expression of CARLo-5 Predicts a Poor Prognosis

Furthermore, the prognostic value of the CARLo-5 expression in malignant GC patients was evaluated. Kaplan-Meier method and log-rank test were used to evaluate the differences of OS

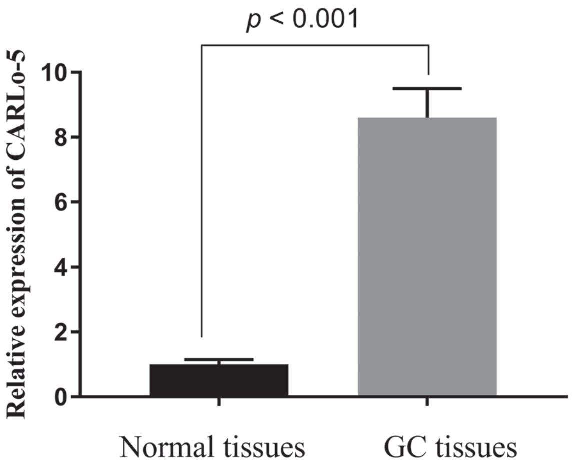


**Figure 1.** Expression levels of CARLo-5 in GC tissues. The expression levels of CARLo-5 were significantly upnregulated in GC tissue samples, as compared with adjacent normal tissue samples ($p < 0.001$).

**Table I.** CARLo-5 level and clinicopathological parameters in patients with GC.

| Characteristics | n | High expression (%) | Low expression (%) | *p* |
|---|---|---|---|---|
| Gender | | | | 0.445 |
| Male | 95 | 45 (47.4) | 50 (52.6) | |
| Female | 145 | 76 (52.4) | 69 (47.6) | |
| Age | | | | 0.191 |
| < 60 | 109 | 60 (55) | 49 (45) | |
| ≥ 60 | 131 | 61 (46.6) | 70 (53.4) | |
| T stage | | | | 0.003 |
| T1-T2 | 118 | 48 (40.7) | 70 (59.3) | |
| T3-T4 | 122 | 73 (60) | 49 (40) | |
| Lymph node metastasis | | | | 0.001 |
| No | 106 | 41 (38.7) | 65 (61.3) | |
| Yes | 134 | 80 (59.7) | 54 (40.3) | |
| Distant metastasis | | | | 0.009 |
| No | 189 | 87 (46) | 102 (54) | |
| Yes | 51 | 34 (66.7) | 17 (33.3) | |
| Differentiation | | | | 0.001 |
| Well/moderate | 141 | 58 (41.1 | 83 (58.9) | |
| Poor | 99 | 63 (63.6) | 36 (36.4) | |
| Tumor size (cm) | | | | 0.194 |
| < 5 | 91 | 41 (44.1) | 50 (55.9) | |
| ≥ 5 | 149 | 80 (53.7) | 69 (46.3) | |

and RFS between the low-expression group and high-expression group. The results indicated that patients with high CARLo-5 expression had shorter mean months of OS and RFS than did patients with low CARLo-5 expression ($p < 0.001$, respectively) (Figures 2, 3).

The univariate analyses showed that T stage ($p = 0.012$), Lymph node metastasis ($p = 0.007$), distant metastasis ($p = 0.004$), differentiation ($p = 0.009$) and CARLo-5 level ($p = 0.002$) were significantly associated with OS (Table II). Multivariate analysis using Cox's regression model was performed and identified that CARLo-5 was independent prognostic factors (RR=2.41; 95% CI 1.13-5.94; $p = 0.005$, Table II). The similar results were also found in RFS (Table III). Our

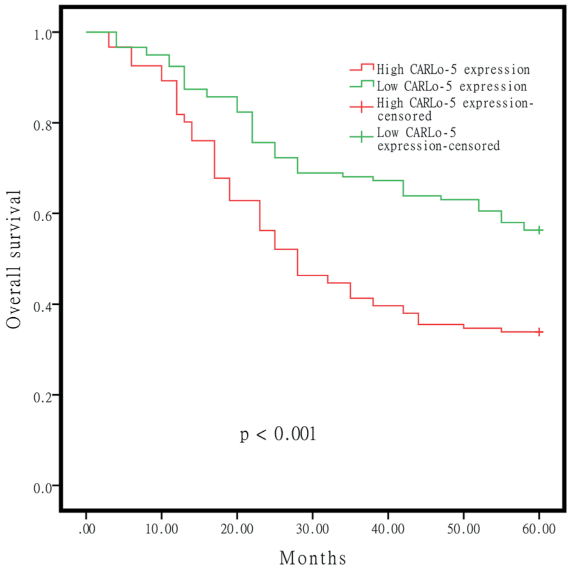


**Figure 2.** Overall survival rate in patients with high CARLo-5 expression was significantly lower than that in patients with low CARLo-5 expression ($p < 0.001$).

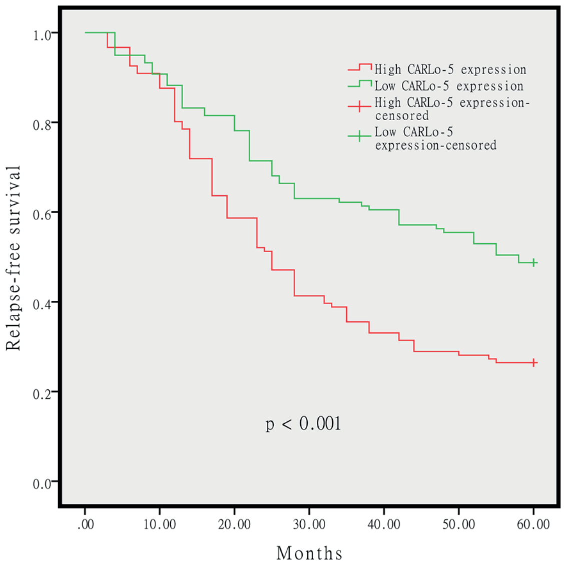


**Figure 3.** Relapse-free survival rate in patients with high CARLo-5 expression was significantly lower than that in patients with low CARLo-5 expression ($p < 0.001$).

**Table II.** Univariate and multivariable Cox regression analysis of OS.

| Viable | Univariate analysis | | | Multivariate analysis | | |
|---|---|---|---|---|---|---|
| | RR | 95 % CI | *p*-value | RR | 95 % CI | *p*-value |
| T stage | 2.56 | 0.83-4.14 | 0.012 | 2.02 | 0.63-3.65 | 0.018 |
| Lymph node metastasis | 3.72 | 1.14-4.73 | 0.007 | 3.13 | 0.88-4.16 | 0.009 |
| Distant metastasis | 2.86 | 1.24-4.21 | 0.004 | 2.21 | 1.03-3.99 | 0.007 |
| Differentiation | 3.55 | 1.73-5.56 | 0.009 | 3.11 | 1.31-4.77 | 0.011 |
| CARLo-5 expression | 2.16 | 0.98-6.65 | 0.002 | 2.41 | 1.13-5.94 | 0.005 |

results revealed that CARLo-5 was a poor independent prognostic factor for both OS and RFS in GC patients.

## Discussion

To the best of our knowledge, this is the first report of the analysis of the prognostic value of CARLo-5 in GC. We found that high CARLo-5 expression in GC tissues, providing the first evidence that CARLo-5 up-regulation is closely associated with GC. Then, we analyzed the association between CARLo-5 expression and clinicopathologic features in GC. The results showed that high CARLo-5 expression were closely correlated with advanced T stage, positive distant metastasis, lymph node involvement, and poor differentiation. Furthermore, Kaplan-Meier analysis showed that patients with high CARLo-5 expression had worse OS and RFS than patients with low CARLo-5 expression. Moreover, both univariate and multivariate analyses revealed that the CARLo-5 expression was a predictor of poor RFS and OS. These findings implicated CARLo-5 as a potentially important contributing factor in GC progression.

Mounting evidence indicates that lncRNAs are associated with a diverse range of functions in cell biology[10,11]. The prognostic potential of lncRNA has been demonstrated for several types of cancer, including gastric cancer[12,13]. For example, Peng et al[14] found that lncRNA SPRY4-IT1 up-regulation occurred more frequently in GC patients with multiple tumor nodes and advanced tumor stage. Moreover, SPRY4-IT1 may serve as a promising biomarker for predicting the prognosis of GC. Fei et al[15] showed that lncRNA LINC00982 expression was downregulated in tumor tissues in patients with gastric cancer (GC) compared with those in the adjacent normal tissues. Furthermore, they identified lncRNA LINC00982 as a poor prognostic biomarker in GC.

Previous studies revealed that CARLo-5 served as a tumor promoter in several tumors. Luo et al[16] showed that a great up-regulation of CARLo-5 was observed in cancer tissues compared to their adjacent normal tissues, and knockdown of CARLo-5 inhibits proliferation of NSCLC cells *in vitro*. Wang et al[17] observed that CARLo-5 was up-regulated in hepatocellular carcinoma specimens and its high expression was associated with poor prognosis of hepatocellular carcinoma patients. Further function experiments showed that CARLo-5 overexpression significantly enhanced cell proliferation, migration, and invasion *in vitro*. More importantly, Zhang et al[18] reported that CARLo-5 was observed to be up-regulated in GC, and knockdown of CARLo-5 in gastric

**Table III.** Univariate and multivariable Cox regression analysis of RFS.

| Viable | Univariate analysis | | | Multivariate analysis | | |
|---|---|---|---|---|---|---|
| | RR | 95 % CI | *p*-value | RR | 95 % CI | *p*-value |
| T stage | 2.83 | 1.21-4.87 | 0.015 | 2.43 | 0.89-3.83 | 0.016 |
| Lymph node metastasis | 3.44 | 1.32-5.16 | 0.004 | 3.07 | 0.57-4.89 | 0.007 |
| Distant metastasis | 2.45 | 1.17-5.89 | 0.007 | 2.04 | 0.89-4.13 | 0.012 |
| Differentiation | 4.14 | 1.22-6.68 | 0.014 | 2.56 | 1.51-5.55 | 0.006 |
| CARLo-5 expression | 2.12 | 0.77-5.54 | 0.004 | 2.76 | 1.42-6.65 | 0.008 |

cancer cell lines significantly inhibited the cell proliferation via inducing G0/G1 cell-cycle arrest and apoptosis. Based on those results, we hypothesized that CARLo-5 may be associated with the overall survival of GC patients.

## Conclusions

We observed for the first time that the up-regulation of CARLo-5 is a frequent event and predicted poor prognosis in GC patients. However, the precise molecular mechanisms by which CARLo-5 is downregulated in GC need to be further investigation.

### Conflict of Interest

The Authors declare that they have no conflict of interests.

## References


1) Jemal A, Bray F, Center MM, Ferlay J, Ward E, Forman D. Global cancer statistics. CA Cancer J Clin 2011; 61: 69-90.
2) Karimi P, Islami F, Anandasabapathy S, Freedman ND, Kamangar F. Gastric cancer: descriptive epidemiology, risk factors, screening, and prevention. Cancer Epidemiol Biomarkers Prev 2014; 23: 700-713.
3) Terry MB, Gaudet MM, Gammon MD. The epidemiology of gastric cancer. Semin Radiat Oncol 2002; 12: 111-127.
4) Mercer TR, Dinger ME, Mattick JS. Long non-coding RNAs: insights into functions. Nat Rev Genet 2009; 10: 155-159.
5) Wang KC, Chang HY. Molecular mechanisms of long noncoding RNAs. Mol Cell 2011; 43: 904-914.
6) Gibb EA, Brown CJ, Lam WL. The functional role of long non-coding RNA in human carcinomas. Mol Cancer 2011; 10: 38
7) Zhan Y, Lin J, Liu Y, Chen M, Chen X, Zhuang C, Liu L, Xu W, Chen Z, He A, Zhang Q, Sun X, Zhao G, Huang W. Up-regulation of long non-coding RNA PANDAR is associated with poor prognosis and promotes tumorigenesis in bladder cancer. J Exp Clin Cancer Res 2016; 35: 83.
8) Li J, Wang YM, Song YL. Knockdown of long non-coding RNA AB073614 inhibits glioma cell proliferation and migration via affecting epithelial-mesenchymal transition. Eur Rev Med Pharmacol Sci 2016; 20: 3997-4002.
9) Zhang Y, Yang R, Lian J, Xu H. LncRNA Sox2ot overexpression serves as a poor prognostic biomarker in gastric cancer. Am J Transl Res 2016; 8: 5035-5043.
10) Tuo YL, Li XM, Luo J. Long noncoding RNA UCA1 modulates breast cancer cell growth and apoptosis through decreasing tumor suppressive miR-143. Eur Rev Med Pharmacol Sci 2015; 19: 3403-3411.
11) Mattick JS. The genetic signatures of noncoding RNAs. PLoS Genet 2009; 5: e1000459.
12) Gao KT, Lian D. Long non-coding RNA MALAT1 is an independent prognostic factor of osteosarcoma. Eur Rev Med Pharmacol Sci 2016; 20: 3561-3565.
13) Zhang JY, Weng MZ, Song FB, Xu YG, Liu Q, Wu JY, Qin J, Jin T, Xu JM. Long noncoding RNA AFAP1-AS1 indicates a poor prognosis of hepatocellular carcinoma and promotes cell proliferation and invasion via upregulation of the RhoA/Rac2 signaling. Int J Oncol 2016; 48: 1590-1598
14) Peng W, Wu G, Fan H, Wu J, Feng J. Long non-coding RNA SPRY4-IT1 predicts poor patient prognosis and promotes tumorigenesis in gastric cancer. Tumour Biol 2015; 36: 6751-6758.
15) Fei ZH, Yu XJ, Zhou M, Su HF, Zheng Z, Xie CY. Upregulated expression of long non-coding RNA LINC00982 regulates cell proliferation and its clinical relevance in patients with gastric cancer. Tumour Biol 2016; 37: 1983-1993.
16) Luo J, Tang L, Zhang J, Ni J, Zhang HP, Zhang L, Xu JF, Zheng D. Long non-coding RNA CARLo-5 is a negative prognostic factor and exhibits tumor pro-oncogenic activity in non-small cell lung cancer. Tumour Biol 2014; 35: 11541-11549.
17) Wang F, Xie C, Zhao W, Deng Z, Yang H, Fang Q. Long non-coding RNA CARLo-5 expression is associated with disease progression and predicts outcome in hepatocellular carcinoma patients. Clin Exp Med 2015 Oct 3 [Epub ahead of print]
18) Zhang Y, Ma M, Liu W, Ding W, Yu H. Enhanced expression of long noncoding RNA CARLo-5 is associated with the development of gastric cancer. Int J Clin Exp Pathol 2014; 7: 8471-8479.
