# Supplementary material for: Expression of Concern: Prognostic value of long non-coding RNA CCAT1 expression in patients with cancer: A meta-analysis
Source: PLoS One. 2023 Apr 20;18(4):e0284940. doi: 10.1371/journal.pone.0284940 (PMC10118116; doi:10.1371/journal.pone.0284940)
Supplement: S1 File — (ZIP) [file pone.0284940.s001.zip › 3 The 11 included studies in PDF/7.pdf]

结肠癌相关转录因子1表达在结直肠癌早期筛查及预后评估中的作用

崔 戈[1],张 婷[2*],崔 杰[3]

([1]湖州师范学院附属第一医院病理科,浙江 湖州 313000;[2]湖州师范学院医学院病理教研室,浙江 湖州 313000;[3]淄博市中医医院内二科,山东 淄博 255300)

[摘 要] **目的**:分析结肠癌相关转录因子1(CCAT-1)mRNA在结直肠癌患者外周血中的表达水平与临床病理特征及预后生存的关联,探讨CCAT-1在结直肠癌早期筛查及病程预后评估中的作用。**方法**:采用实时定量PCR法(qPCR)检测结直肠癌(60例)、结直肠腺瘤(30例)及健康志愿者(30例)外周血中CCAT-1 mRNA表达,采用ROC曲线判断CCAT-1对结直肠癌的诊断价值,计算Youden指数确定CCAT-1对结直肠癌的诊断阈值;采用双变量相关(bivariate correlation)Pearson检验分析CCAT-1 mRNA表达水平与结直肠癌患者临床病理特征;采用Kaplan-Meier生存分析(Log-rank检验)分析CCAT-1 mRNA表达与结直肠癌患者3年生存率的相关性,绘制生存曲线。**结果**:结直肠癌患者外周血CCAT-1 mRNA水平相对于结直肠腺瘤患者($P < 0.01$)、健康志愿者($P < 0.01$)显著高表达,CCAT-1 mRNA在健康志愿者、结直肠腺瘤及结直肠癌患者外周血中的表达量呈逐渐递增趋势;结直肠癌患者外周血CCAT-1 mRNA表达量与肿瘤直径($P < 0.05$)、分化程度($P < 0.01$)、原发灶浸润深度($P < 0.01$)、淋巴结转移数($P < 0.01$)、远处转移($P < 0.05$)及TNM分期($P < 0.05$)呈显著正相关,与3年生存率呈负相关($P < 0.05$);CCAT-1 mRNA高表达组生存时间显著低于低表达组($P < 0.01$)。**结论**:CCAT-1可作为一种有价值的结直肠癌早期筛查及预后评估的标志物。

[关键词] 结肠癌相关转录因子1(CCAT-1);结直肠癌;早期筛检;病程评估

[中图分类号] R735.34 [文献标志码] A [文章编号] 1007-4368(2015)07-1008-05

doi:10.7655/NYDXBNS20150721

Value of colon cancer associated transcription factor 1 expression in early screening and prognosis assessment for patients with colorectal carcinoma

Cui Ge[1], Zhang Ting[2*], Cui Jie[3]

(*[1]Department of Pathology, the First Affiliated Hospital of Huzhou Teachers College, Huzhou 313000; [2]Department of Pathology, School of Medicine, Huzhou Teachers College, Huzhou 313000; [3]Department of Internal Medicine Ward II, Zibo Hospital of Traditional Chinese Medicine, Zibo 255300, China*)

[Abstract] **Objective**: To investigate the relation between mRNA expression of CCAT-1 in the peripheral blood, clinicopathological features and prognosis of patients with colorectal carcinoma. **Methods**: Real-time quantitative polymerase chain reaction (qPCR) was performed to detect CCAT-1 mRNA expression levels in patients with colorectal carcinoma (60 cases) and colorectal adenoma (30 cases) as well as healthy volunteers (30 cases). The value of CCAT-1 in diagnosis of colorectal carcinoma was evaluated by ROC curve, diagnosis threshold of CCAT-1 mRNA level in colorectal carcinoma was determined by Youden index. Bivariate correlation analysis was performed to analyze the correlation of the expression levels of CCAT-1 and clinicopathological features in patients with colorectal carcinoma. Kaplan-Meier survival curve was performed to analyze the correlation between CCAT-1 expression and survival of patients with colorectal carcinoma. **Results**: CCAT-1 mRNA expression levels in patients with colorectal carcinoma were significantly higher than those in patients with adenoma ($P < 0.01$) and healthy volunteers ($P < 0.01$). CCAT-1 mRNA expression levels were positively correlated with the diameters of tumor, grade of differentiation, invasion depth of primary tumor, the number of lymph node and

[基金项目] 湖州市科技公益性技术应用研究(2013GY19);湖州市自然科学基金(2014YZ04);浙江省医药卫生研究计划(2015KYB372)

*通信作者(Corresponding author),E-mail:grape_chang@163.com

distant metastasis ($P < 0.05$) and TNM stage ($P < 0.01$), and were negatively correlated with survival time ($P < 0.05$). The survival time of patients with colorectal carcinoma with higher-level CCAT-1 expression was significantly shorter than that of the lower-level group. **Conclusion**: CCAT-1 can be considered as a valuable marker for early screening and prognosis assessment of colorectal carcinoma.

[Key words] colon cancer associated transcript 1 (CCAT-1); colorectal carcinoma; early screening; prognosis

[Acta Univ Med Nanjing,2015,35(07):1008-1012]

结直肠癌(colorectal carcinoma,CRC)在我国发病率约(20.10~23.03)/10 万,致死率约(10.1~11.1)/10 万,在我国恶性肿瘤中分别居第 4 位、第 5 位[1-2]。结直肠腺瘤(colorectal adenoma,CRA)被认为是结直肠最主要的癌前病变[3],结直肠腺瘤发展为腺癌的过程被称为“结直肠腺瘤-癌序列”(colorectal adenoma-carcinoma sequence)[4]。因此,研究结直肠腺瘤-癌序列中表达特异、灵敏、可反映病程进展程度的分子标志物,及早发现癌前病变并彻底清除病变组织对于降低结直肠癌的发病率和病死率具有重要意义[4]。

2012 年 Nissan 等[5]发现了一种长链非编码RNA(long noncoding RNA,lncRNA)——结肠癌相关转录因子 1(colon cancer associated transcript 1,CCAT-1),其 RNA 在结直肠癌患者肿瘤组织及外周血中均显著高表达,但在正常结直肠组织中无表达。

为了评估 CCAT-1 在国人“结直肠腺瘤-癌序列”过程中的表达变化,评估 CCAT-1 在结直肠癌早期筛查及病程进展预后中的价值,本研究组采用实时定量 PCR 法(qPCR)检测结直肠癌患者、结直肠腺瘤患者及健康志愿者外周血中 CCAT-1 mRNA 表达,分析 CCAT-1 mRNA 表达水平与患者临床病理特征及预后生存的相关性。

1　对象和方法

1.1　对象

临床样本采集均经样本提供者本人或其家属知情同意,并经主管部门医学伦理委员会审查后实施。采用 EDTA-K_2 抗凝采血管采集湖南师范学院附属第一医院 2009~2010 年首次入院的结直肠癌患者(60 例)、结直肠腺瘤患者(30 例)及健康志愿者(30 例)外周静脉血(>6 mL),离心后吸取血清,置于-80℃冰箱冻存备用。结直肠癌及结直肠腺瘤组织均经病理科确诊,患者未经任何抗肿瘤治疗,排除其他重大或急慢性疾病者。健康志愿者经健康体检无重大急慢性疾病者纳入研究(正常对照组)。

1.2　方法

1.2.1　结直肠癌临床病理特征及预后随访登记与统计

统计所有入组结直肠癌患者临床病理参数:包括性别、年龄、肿瘤部位、大体分型、肿瘤直径、神经脉管累及、分化程度、原发灶浸润深度、淋巴结转移、远处转移。肿瘤病理类型及分化级别诊断标准参照 2010 年 WHO 消化系统肿瘤分类[6]确定。TNM 分期参照 2009 年美国癌症联合委员会(AJCC)/国际抗癌联盟(UICC)结直肠癌 TNM 分期系统(第 7 版)[7]登记。通过电话咨询及公安系统户籍调查,随访入组结直肠癌患者目前生存状态,分别以“死亡=0”、“生存=1”及“失访=2”记录当前生存状态,计算患者 3 年生存率。

1.2.2　qPCR 法检测外周血样本 CCAT-1 mRNA 表达

取上述血清样本经 TRIzol 充分裂解,离心取上清,提取总 RNA,采用 Fermentas RevertAid™ 反转录试剂盒(K1622,Thermo 公司,美国)反转录成 cDNA,使用荧光定量 PCR 仪(ABI 7900T)进行 PCR 扩增。设计并合成 PCR 引物:CCAT-1 上游引物 5′-GAGGTGAGCAGTGGGTGAGT -3′,下游引物 5′-GCTCAGGCAGTAAGGTGAGC-3′,扩增长度 100 bp。以 β-actin 作为内参照基因,β-actin 上游引物为 5′-CCAACTAATGTTCAGCGTTA-3′,下游引物为 5′-TACGATGATAGGAATGGTTG-3′,扩增长度 400 bp。qPCR 反应体系:2 × SYBR mix 7.5 μL,Primer (10 μmol/L) 0.2 μL,cDNA 1.0 μL,ddH_2O 补至 15 μL。反应程序:94℃ 2 min;95℃ 15 s,60℃ 30 s,72℃ 30 s,40 个循环。在 72℃延伸阶段采集荧光,记录 Ct 值。实验重复 3 次。采用 SDS 2.3 软件分析结果,按 $2^{-\Delta Ct}$ 换算成目的基因 mRNA 相对表达量(ΔCt 是同一样本中目的基因 Ct 值减去内参 Ct 值),以正常组织 $2^{-\Delta Ct}$ 为 1 校正各组结果,绘制各组样本 mRNA 相对表达量柱状图,纵坐标以 $2^{-\Delta Ct}$ 表示。

1.3　统计学方法

运用统计软件 SPSS19.0 进行数据分析。计数数据以均数 ± 标准差($\bar{x} \pm s$)表示,多组间计量资料比较用 ANOVA 分析;采用 ROC (receiver operating characteristic)曲线判断 CCAT-1 mRNA 表达对结直

肠癌的诊断价值；计算 Youden 指数(YI)确定结直肠癌诊断阈值；采用双变量相关(bivariate correlation)Pearson 检验分析变量间相关性；结直肠癌患者单因素生存分析采用 Kaplan-Meier 生存分析，Log-rank 检验，绘制生存曲线；以 $P \leq 0.05$ 为差异有统计学意义。

2 结 果

2.1 结直肠癌、结直肠腺瘤患者及健康志愿者外周血 CCAT-1 mRNA 表达

多组间比较 ANOVA 分析显示，结直肠癌患者外周血 CCAT-1 mRNA 相对表达量显著高于结直肠腺瘤患者（均值差 0.001 5 ± 0.000 3，$P < 0.001$，95%可信区间 0.000 9~0.002 2）及健康志愿者（均值差 0.003 2 ± 0.000 3，$P < 0.001$，95%可信区间 0.002 6~0.003 8）；结直肠腺瘤患者外周血 CCAT-1 mRNA 相对表达量亦显著高于健康志愿者(均值差 0.001 7 ± 0.000 4，$P < 0.001$，95%可信区间 0.000 9~0.002 4，图 1)。

2.2 评价 CCAT-1mRNA 表达对结直肠癌的诊断价值并确定诊断临界点

结直肠癌 ROC 曲线下面积（0.793 ± 0.046，上下限 0.703~0.883，图 2A)，与结直肠腺瘤组(0.207 ± 0.046，上下限 0.117~0.297，图 2B)相比有显著差异($P < 0.01$)。通过 ROC 曲线计算 Youden 指数最大值为 0.533 作为诊断临界点，其相应的 CCAT-1 mRNA 表达水平为 0.003 1。

2.3 结直肠癌患者外周血 CCAT-1 mRNA 表达与临床病理特征的相关性

根据诊断临界点将结直肠癌患者分为 CCAT-1 mRNA 低表达组与高表达组。采用双变量相关比较结直肠癌患者外周血 CCAT-1 mRNA 表达量与临床病理特征的相关性，结果发现：结直肠癌患者外周血 CCAT-1 mRNA 相对表达量与肿瘤直径（$P < 0.05$）、分化程度($P < 0.01$)、原发灶浸润深度($P < 0.01$)、淋巴结转移数($P < 0.01$)、远处转移($P < 0.05$)及 TNM 分期($P < 0.05$)呈显著正相关，与 3 年生存率呈负相关($P < 0.05$，表 1)。

2.4 结直肠癌患者外周血 CCAT-1 mRNA 表达与患者生存时间的相关性

以 Youden 指数最大值确定的 CCAT-1 mRNA 表达水平 0.003 1 为诊断临界点，将结直肠癌患者分为 CCAT-1 mRNA 高表达组及低表达组，采用 Kaplan-Meier 生存分析结直肠癌患者外周血 CCAT-1 mRNA 表达与患者生存时间的相关性。结果显示，结直肠癌患者外周血 CCAT-1 mRNA 低表达组患者平均生存时间为 28.114 个月，中位生存时间为 27.000 个月，高表达组患者平均生存时间为 18.606 个月，中位生存时间为 16.100 个月。CCAT-1 mRNA 高表达组生存时间显著低于低表达组（$P < 0.01$），Kaplan-Meier 生存分析(Log-rank 检验)显示 CCAT-

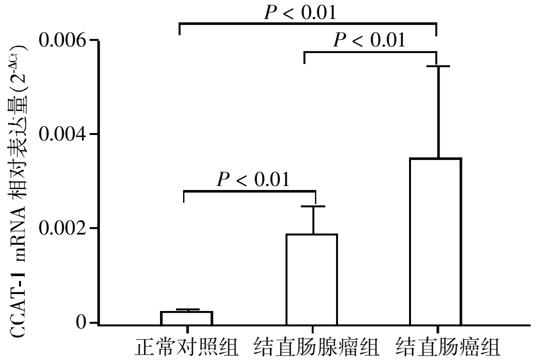

图 1 结直肠癌、结直肠腺瘤患者及健康志愿者外周血 CCAT-1 mRNA 相对表达

Figure 1 Relative expression of CCAT-1 mRNA in the peripheral blood of patients with colorectal carcinoma, colorectal adenoma and healthy volunteer

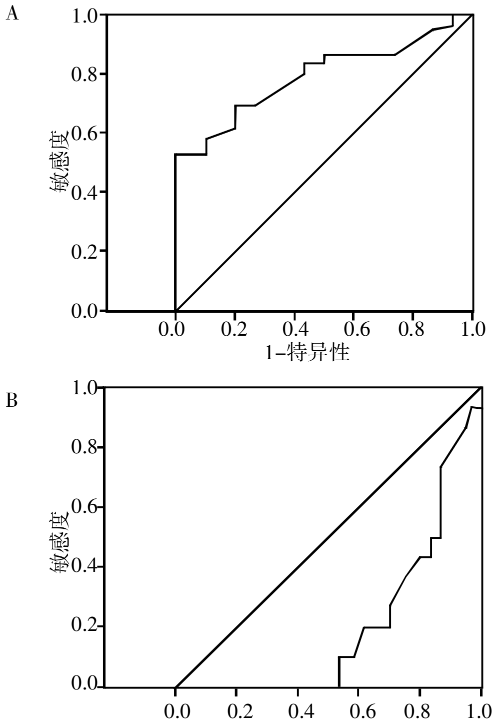

A：结直肠癌组；B：结直肠腺瘤组。

图 2 结直肠癌及结直肠腺瘤患者外周血 CCAT-1 mRNA 表达的 ROC 曲线

Figure 2 ROC Curves of different CCAT-1 mRNA expressions in the peripheral blood of patients with colorectal carcinoma and colorectal adenoma

表 1　结直肠癌患者外周血 CCAT-1 mRNA 表达与临床病理特征及 3 年生存率的相关性

Table 1　Correlation between CCAT-1 mRNA expression in the peripheral blood of patients with colorectal carcinoma and their clinical and pathological features, and 3-year survival rates

临床病理参数	例数	*r*(Pearson)值	*P* 值
性别		−0.135	0.303
男	38		
女	22		
年龄(岁)		0.174	0.184
<60	21		
≥60	39		
肿瘤部位		0.144	0.273
升结肠	15		
横结肠	12		
降结肠	8		
乙状结肠	2		
直肠	23		
大体分型		0.070	0.596
肿块型	12		
浸润型	22		
溃疡型	26		
肿瘤直径(cm)		0.270	0.037
<3	26		
≥3	34		
神经脉管累及		0.022	0.869
未累及	27		
累及	33		
分化程度		0.351	0.006
高分化	14		
中分化	18		
低分化	28		
原发灶浸润深度		0.357	0.005
T_1+T_2	20		
T_3+T_4	40		
淋巴结转移		0.383	0.002
N_0	12		
N_1	13		
N_2	35		
远处转移		0.281	0.030
M_0	43		
M_1	17		
TNM 分期		0.314	0.014
Ⅰ+Ⅱ	25		
Ⅲ+Ⅳ	35		
3 年生存率(月)		−0.571	<0.001
<18	17		
≥18	43		

1 mRNA 高表达组生存时间显著低于低表达组($P < 0.01$,图 3,表 2)。

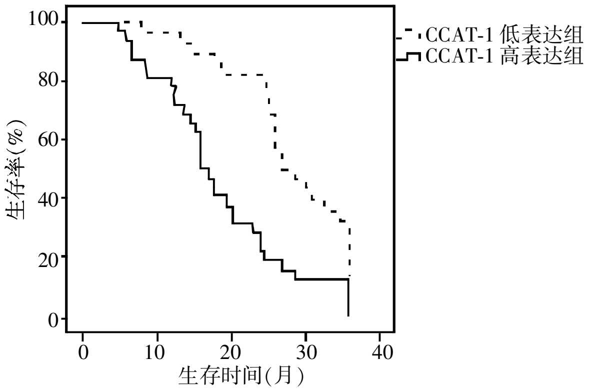

图 3　CCAT-1 mRNA 不同表达水平的结直肠癌患者 Kaplan-Meier 生存曲线

图 3　Kaplan-Meier survival curves of different CCAT-1 mRNA expressions in peripheral blood of patients with colorectal carcinoma

3　讨　论

随着微阵列基因芯片、全基因组测序技术的出现，人们发现一类不编码蛋白质且转录本长度在 200~100 000 个核苷酸(nt)不等的 RNA——lncRNA,可通过调节 DNA 甲基化、组蛋白修饰、染色质重构和作为 miRNA 的前体等,在细胞发育、新陈代谢、肿瘤发生发展中发挥重要生物学作用[8-9]。

2012 年 Nissan 等[5]通过代表性差别分析法发现了一种长链非编码 RNA 转录因子——CCAT-1,定位于染色体组 8q24.21(128.0~128.6 Mb),长度为 5 200 nt,含有 polyA 尾巴,定位在细胞核中其转录位点附近。最近研究发现 CCAT-1 通过参与染色质高级结构维持实现对癌基因 MYC 转录调控，可能参与结直肠癌的发生发展[10]。

为了验证 CCAT-1 在国人结直肠癌及结直肠腺瘤患者外周血中的表达水平,评估 CCAT-1 在“结直肠腺瘤−癌序列”筛检中的价值,本研究采用 qPCR 分别检测了结直肠癌及结直肠腺瘤患者、健康志愿者外周血中 CCAT-1 mRNA 表达,结果发现,结直肠癌患者外周血 CCAT-1 mRNA 水平相对于结直肠腺瘤患者($P < 0.01$)、健康志愿者($P < 0.01$)显著高表达,CCAT-1 mRNA 在健康志愿者、结直肠腺瘤及结直肠癌患者外周血中的表达量呈逐渐递增趋势,结直肠癌组 ROC 曲线下面积与结直肠腺瘤组相比有显著差异($P < 0.01$),表明 CCAT-1 mRNA 表达对结直肠癌有较高的诊断价值,CCAT-1 可作为结直肠癌早期筛查的一种有价值的标志物。

表 2 结直肠癌患者外周血 CCAT-1 mRNA 不同表达分组的生存分析

Table 2 Survival analysis of different CCAT-1 mRNA expressions in the peripheral blood of patients with colorectal carcinoma

分组	平均生存时间			中位生存时间			Log Rank(Mantel-Cox)检验	
	估计	标准误	95%可信区间	估计	标准误	95%可信区间	χ^2 值	P 值
CCAT-1 低表达组	28.114	1.492	25.190~31.039	27.000	2.910	21.296~32.704		
CCAT-1 高表达组	18.606	1.592	15.486~21.727	16.100	1.179	13.790~18.410		
总体估计	23.043	1.252	20.590~25.497	24.000	2.582	18.939~29.061	11.942	0.001

为了进一步分析 CCAT-1 与结直肠癌临床病理参数的关联性，评估 CCAT-1 在结直肠癌病程进展中的价值，本文分别分析了结直肠癌患者外周血 CCAT-1 mRNA 表达水平与结直肠癌患者的临床病理参数及预后的相关性，结果发现，结直肠癌患者外周血 CCAT-1 mRNA 相对表达量与肿瘤直径($P < 0.05$)、分化程度($P < 0.01$)、原发灶浸润深度($P < 0.01$)、淋巴结转移数（$P < 0.01$）、远处转移（$P < 0.05$)及 TNM 分期($P < 0.05$)呈显著正相关，与 3 年生存率呈负相关（$P<0.05$)；Kaplan-Meier 生存分析(Log-rank 检验)显示 CCAT-1 mRNA 高表达组生存时间显著低于低表达组（$P < 0.01$），该结果表明 CCAT-1 可用于结直肠癌患者病程及预后的评估。

综上所述，通过外周血筛查 CCAT-1 的 mRNA 表达对于结直肠癌早期筛查及病程预后评估具有一定价值进而有助于结直肠癌的早诊早治。

[参考文献]

[1] Zheng ZX,Zheng RS,Zhang SW,et al. Colorectal cancer incidence and mortality in China,2010[J]. Asian Pac J Cancer Prev,2014,15(19):8455-8460

[2] Liu S,Zheng R,Zhang M,et al. Incidence and mortality of colorectal cancer in China,2011[J]. Chin J Cancer Res,2015,27(1):22-28

[3] Fearon ER,Vogelstein B. A genetic model for colorectal tumorigenesis[J]. Cell,1990,61(5):759-767

[4] Leslie A,Carey FA,Pratt NR,et al. The colorectal adenoma-carcinoma sequence[J]. Br J Surg,2002,89(7):845-860

[5] Nissan A,Stojadinovic A,Mitrani-Rosenbaum S,et al. Colon cancer associated transcript-1:a novel RNA expressed in malignant and pre-malignant human tissues [J]. Int J Cancer,2012,130(7):1598-1606

[6] Bosman FT. WHO classification of tumours of the digestive system[M]. 4th ed. Lyon,France:IARC Press,2010

[7] Sobin LH GM,Wittekind Ch. TNM Classification of Malignant Tumors[M]. 7th ed. Oxford:Wiley-Blackwell,2009:310

[8] Struhl K. Transcriptional noise and the fidelity of initiation by RNA polymerase Ⅱ[J]. Nat Struct Mol Biol,2007,14(2):103-105

[9] Mercer TR,Dinger ME,Mattick JS. Long non-coding RNAs: insights into functions[J]. Nat Rev Genet,2009,10(3):155-159

[10] Xiang JF,Yin QF,Chen T,et al. Human colorectal cancer-specific CCAT1-L lncRNA regulates long-range chromatin interactions at the MYC locus[J]. Cell Res,2014,24(5):513-531

[收稿日期] 2015-03-07
