# Supplementary material for: Expression of Concern: Prognostic value of long non-coding RNA CCAT1 expression in patients with cancer: A meta-analysis
Source: PLoS One. 2023 Apr 20;18(4):e0284940. doi: 10.1371/journal.pone.0284940 (PMC10118116; doi:10.1371/journal.pone.0284940)
Supplement: S1 File — (ZIP) [file pone.0284940.s001.zip › 3 The 11 included studies in PDF/8.pdf]

Int J Clin Exp Pathol 2015;8(8):9440-9445
www.ijcep.com /ISSN:1936-2625/IJCEP0011501

Original Article

Overexpression of long non-coding RNA CCAT1 is a novel biomarker of poor prognosis in patients with breast cancer

Xin-Feng Zhang[1*], Ting Liu[2*], Yang Li[1], Su Li[2]

[1]*Department of Breast Surgery, Liaoning Cancer Hospital and Institute, Shenyang 110042, China;* [2]*Department of Radiology, First Affiliated Hospital, China Medical University, Shenyang 110001, China.* [*]*Equal contributors.*

Received June 16, 2015; Accepted July 24, 2015; Epub August 1, 2015; Published August 15, 2015

Abstract: Introduction: Recent studies have demonstrated that lncRNA CCAT1 was increased in many types of cancers and was involved in various cellular processes related to carcinogenesis. However, the clinical significance and prognostic value of lncRNA CCAT1 in breast cancer (BC) haven't been investigated. Methods: Expression levels of lncRNA CCAT1 in 92 pairs of BC cancer tissues and adjacent normal tissues were detected by quantitative real-time PCR. In order to determine its prognostic value, overall survival and progression-free survival were evaluated using the Kaplan-Meier method, and multivariate analysis was performed using the Cox proportional hazard analysis. Results: Expression levels of lncRNA CCAT1 in BC tissues were significantly higher than those in adjacent normal tissues. High expression of lncRNA CCAT1 was associated with differentiation grade, TNM stage, and lymph node metastases. Kaplan-Meier analysis with the log-rank test indicated that high expression of lncRNA CCAT1 had a decreased overall survival and progression-free survival. Multivariable analysis was further identified high expression of lncRNA CCAT1 as an independent prognosis factor for overall survival and progression-free survival. Conclusions: Our findings provided that the expression of lncRNA CCAT1 was up-regulated in BC and associated with overall survival as well as progression-free survival, suggesting that lncRNA CCAT1 could be a potential prognostic biomarker for BC progression.

Keywords: lncRNA CCAT1, breast cancer, quantitative real-time PCR, prognosis

Introduction

Breast cancer (BC) is the most prevalent cancer among women and affects approximately one million women worldwide [1]. Although improvements in early detection and treatment have decreased breast cancer mortality rates in recent years, prevention and therapy of breast cancer remain a major public health concern [2]. In China, breast cancer mortality has doubled over the past 30 years [3]. Therefore, a better understanding of the molecular mechanisms involved in BC progression will be helpful to exploit useful prognostic biomarker and therapeutic target for BC therapy.

It is well known that protein-coding genes account for only 2% of the total genome; where as the vast majority of the human genome can be transcripted into non-coding RNAs [4]. Among them there are long non coding RNAs (lncRNAs), which are more than 200 nucleotide in length with limited or no protein-coding capacity [5]. LncRNAs are often expressed in a disease-, tissue- or developmental stage-specific manner making these molecules attractive therapeutic targets and pointing toward specific functions for lncRNAs in development and diseases, in particular human cancer [6-8]. More and more evidence have revealed the contribution of lncRNAs as having oncogenic and tumors suppressor roles in tumorigenesis. Recently, lncRNA CCAT1 has been shown to be up-regulated in some types of cancers, such as colon cancer, gastric cancer, gallbladder cancer and hepatocellular carcinoma [9-12]. However, the clinical significance of lncRNA CCAT1 in BC has not yet been elucidated. Thus, the aim of this study was to investigate the diagnostic and prognostic values of lncRNA CCAT1 in BC.

In the present study, the expression levels of lncRNA CCAT1 in human BC tissues and adjacent normal tissues were examined by quanti-

Table 1. Relationship between lncRNA CCAT1 expression level and clinicopathologic features of BC

Variable	Number	lncRNA CCAT1 expression		*P value*
		Low	High	
Age (years)				0.919
<50	38	18	20	
≥50	54	25	29	
Tumor size (cm)				0.510
<2.5	61	30	31	
≥2.5	31	13	18	
ER				0.492
Positive	59	26	33	
Negative	33	17	16	
PR				0.537
Positive	67	30	37	
Negative	25	13	12	
HER-2				0.224
Positive	49	20	29	
Negative	43	23	20	
Differentiation grade				0.003
G1/G2	63	36	27	
G3	29	7	22	
TNM stage				0.000
I/II	54	35	19	
III	38	8	30	
Lymph nodes metastasis				0.004
No	74	40	34	
Yes	18	3	15	

ER = estrogen receptor; PR = progesterone receptor; HER-2 = c-erb B-2.

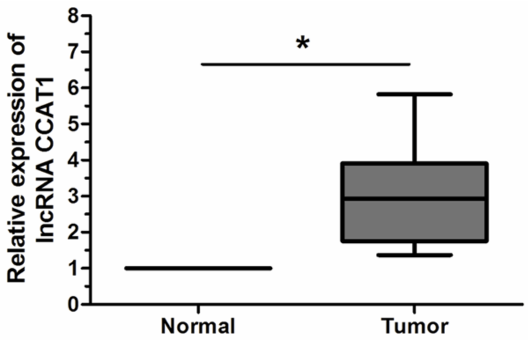

Figure 1. The relative expression level of lncRNA CCAT1 in BC tissues and adjacent normal tissues. lncRNA CCAT1 expression was significantly higher in BC tissues compared with adjacent normal tissues. Results are expressed as mean ± SD for three replicate determination. *P<0.05.

tative real-time PCR (qRT-PCR), and the correlation between lncRNA CCAT1 dysregulation and clinical characteristics and prognosis were analyzed.

Methods

Patients and surgical specimens

92 pairs of BC tissue and matched adjacent normal tissue specimens were collected from patients who underwent surgery between 2005 and 2008 in the Liaoning Cancer Hospital and Institute. The fresh tissue specimens were collected and immediately placed in liquid nitrogen until use. The clinicopathological variables of patients were shown in **Table 1**. None of the patients recruited in this study had undergone preoperative chemotherapy or radiotherapy. This study was approved by the Research Ethics Committee of the Liaoning Cancer Hospital and Institute. Written informed consent was obtained from all patients. All specimens were handled and made anonymous according to the ethical and legal standards.

RNA isolation and cDNA synthesis

Total RNAs were extracted from tumor tissues and adjacent normal tissues using Trizol reagent (Invitrogen) according to the manufacturer's instructions. The yield and quality of total RNA was evaluated by measuring the absorbance at 260 and 280 nm. Only samples with an A260:A280 ratio between 1.8 and 2.1 was considered for further analysis. cDNAs were synthesized using the PrimeScript™ RT reagent kit with gDNA Eraser (Takara) according to the manufacturer's protocol. Briefly, 1 μg total RNA, 2 μl 5× gDNA Eraser Buffer, 1 μl gDNA Eraser and RNase Free dH_2O, were combined in a total reaction volume of 10 μl and incubated at 42°C for 2 min to eliminate the genomic DNA. Ten microliters of reverse-transcription reaction mixture was then added, and the mixture was incubated at 37°C for 15 min, followed by 85°C for 5 s to generate the cDNA.

Quantitative real-time PCR

The expression of lncRNA CCAT1 was quantified using SYBR® Premix Ex Tag™ II (Takara) according to the manufacturer's instructions on the ABI 7500 Real-Time PCR System (Applied Biosystems). Briefly, the 20 μl reaction mixtures

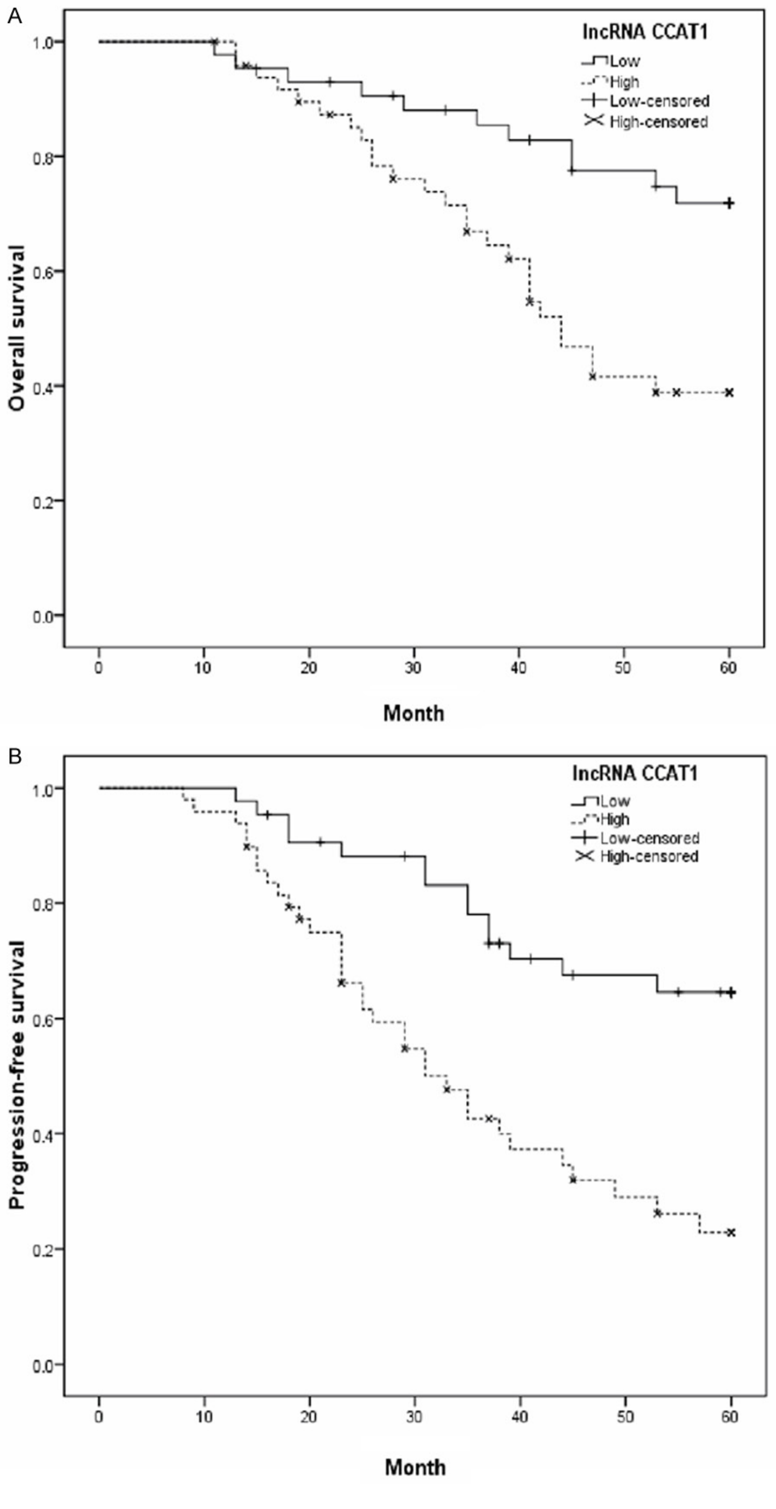

Figure 2. Kaplan-Meier curves for survival time in patients with BC divided according to lncRNA CCAT1 expression. Overall survival and progression-free survival of patients with high vs. low lncRNA CCAT1 expression levels are shown. A. The 5-year overall survival rate of BC patients with high lncRNA CCAT1 was significantly poorer compared to those patients with low lncRNA CCAT1 ($P<0.05$). B. The 5-year progression-free survival survival rate of BC patients with high lncRNA CCAT1 was significantly poorer compared to those patients with low lncRNA CCAT1 ($P<0.05$).

were incubated at 95°C for 30 s for the initial denaturation stage, followed by 40 cycles at 95°C for 5 s and 60°C for 34 s. Small nucleolar RNA U6 was used to normalize the target gene expression. The ΔCt method was used to calculate the relative expression of lncRNA CCAT1 in BC tissues in comparison with paired normal tissues, respectively. Each sample was examined in triplicate. The primers used in this study were synthesized by Invitrogen with the sequences as follows: 5'-TCACTGACAAC-ATCGACTTTGAAG-3' (Forward) and 5'-GGAGAAAACGCTTAGC-CATACAG-3' (Reverse) for CCAT1; 5'-GCGCGTCGTGAAGCGT-TC-3' (Forward) and 5'-GTGC-AGGGTCCGAGGT-3' (Reverse) for U6.

Statistical analysis

Statistical analysis was conducted using the SPSS 18.0 for windows. The correlation between lncRNA CCAT1 expression and clinicopathological features were evaluated by the chi-square test. Overall survival and progression-free survival were calculated by Kaplan-Meier survival analysis and compared by the log rank test. All data were presented as mean ± SD. *P* values less than 0.05 was considered statistically significant.

Results

lncRNA CCAT1 was significantly up-regulated in BC tissues

We analyzed the expression levels of lncRNA CCAT1 in 92 pairs of BC tissues and adjacent normal tissues from BC patients. As revealed by qRT-PCR analysis, lncRNA CCAT1

Table 2. Multivariate Cox proportional hazard model analysis of overall survival and progression-free survival in BC patients

	Overall survival			Progression-free survival		
	Hazard ratio	95% CI	*P*	Hazard ratio	95% CI	*P*
Age (years)	1.318	0.692-2.218	0.352	1.176	0.497-1.865	0.217
Tumor size	1.926	0.615-5.228	0.119	1.685	0.536-4.882	0.086
ER	1.278	0.305-3.138	0.594	1.453	0.799-2.853	0.482
PR	2.037	0.514-3.863	0.085	3.213	0.677-4.947	0.139
HER-2	2.542	0.714-5.125	0.079	2.178	0.743-4.572	0.092
Differentiation grade	3.411	2.366-8.815	0.018	3.704	2.693-9.327	0.013
TNM stage	2.607	1.804-5.312	0.021	3.827	2.016-8.574	0.008
Lymph nodes metastasis	3.174	1.47-6.189	0.031	3.635	1.984-7.823	0.014
CCAT1 expression	2.891	1.483-6.215	0.019	3.593	1.997-7.835	0.005

ER = estrogen receptor; PR = progesterone receptor; HER-2 = c-erb B-2.

expression level was significantly higher in BC tissues compared with adjacent normal tissues (**Figure 1**, P<0.05). The mean lncRNA CCAT1 expression level of all breast cancer cases was 2.94, which was utilized to divide breast cancer patients into two groups. 43 cases were assigned to the low-expression group; the remaining 49 cases were assigned to the high-expression group.

Association between lncRNA CCAT1 expression and the clinicopathological features of BC

Subsequently, the correlation of lncRNA CCAT1 expression with clinicopathological features of BC patients was shown in **Table 1**. By statistical analyses, our results indicated that high lncRNA CCAT1 expression was significantly correlated with differentiation grade, TNM stage, and lymph node metastases ($P<0.05$) of BC patients. However, the expression of lncRNA CCAT1 was not associated with other clinicopathological factors of BC patients, including age, tumor size, ER, PR, as well as HER-2 ($P>0.05$). These data indicated that up-regulation of lncRNA CCAT1 might play a critical role in BC progression.

Association between lncRNA CCAT1 expression and survival in BC patients

To further investigate the correlations of lncRNA CCAT1 expression level with survival of BC patients, Kaplan-Meier analyses were performed. As shown in **Figure 2A**, the 5-year overall survival of high lncRAN CCAT1 expression group was significantly shorter than that of low lncRAN CCAT1 expression group ($P<0.05$). Moreover, the 5-year progression-free survival of high lncRAN CCAT1 expression group was also significantly shorter than that of low lncRAN CCAT1 expression group (**Figure 2B**, $P<0.05$). Furthermore, in a multivariate Cox model, we found that lncRAN CCAT1 expression was an independent poor prognostic factor for both 5-year overall survival (HR=2.891, CI=1.483-6.215, $P=0.019$, **Table 2**) and 5-year progression-free survival (HR=3.593, CI=1.997-7.835, $P=0.005$, **Table 2**) in BC patients.

Discussion

A growing number of novel treatment strategies have been developed for BC, including molecular targeted therapy, gene therapy, and immunotherapy. However, satisfactory therapeutic outcomes have not been achieved, and breast cancer remain a major public health concern. Recently, more and more studies has been intensely focused on the roles of dysregulated lncRNA expression in various human cancer types including human BC [13, 14]. Lots of studies showed that aberrant lncRNAs were associated with proliferation, apoptosis, metastasis, and chemoresistance of breast cancer cells, For example, Shi et al revealed that SPRY4-IT1 was significantly upregulated in breast cancer; the knockdown of SPRY4-IT1 significantly suppressed proliferation and caused apoptosis of breast cancer cells in vitro [15]. Rajnish et al showed that HOTAIR was increased in breast cancer, and HOTAIR expression level in breast cancer is a powerful predictor of eventual metastasis and death [16]. Shi et al found that lncRNA-ATB promotes trastuzumab resistance and invasion metastasis cascade in breast cancer [17]. Thus, identification

of the important lncRNA expression signatures in BC development and progression will be helpful to find potential diagnostic and prognostic markers for BC diagnosis and treatment.

LncRNA colon cancer-associated transcript 1 (CCAT1) is a recently discovered 2628 nucleotide lncRNA, which is located in the vicinity of a well known transcription factor c-Myc [18]. Xiang et al found that CCAT1-L locus was located within a strong super-enhancer and was spatially close to MYC. Knockdown of CCAT1-L reduced long-range interactions between the MYC promoter and its enhancers. These results reveal an important role of lncRNA CCAT1 in gene regulation at the MYC locus [19].

He et al CCAT1 was significantly overexpressed in colon cancer tissues when compared with normal tissues, and its increased expression was correlated with patients' clinical stage, lymph nodes metastasis, and survival time after surgery. Moreover, c-Myc could promote CCAT1 transcription by directly binding to its promoter region, and upregulation of CCAT1 expression in colon cancer cells promoted cell proliferation and invasion [20]. Yang et al showed lncRNA CCAT1 were markedly increased in gastric carcinoma tissues; c-Myc induction of CCAT1 holds an important role in gastric carcinoma and implicate the potential application of CCAT1 in the treatment of gastric carcinoma [10]. However, the role of lncRNA CCAT1 expression in the development and progression of BC is not fully understood.

In the present study, we focused on the expression and clinical significance of lncRNA CCAT1 in BC. Our results showed that lncRNA CCAT1 expression in BC tissues was significantly higher than that in adjacent normal tissues. The relationships of the lncRNA CCAT1 with various clinical features of BC were analyzed. We found that high expression of lncRNA CCAT1 was correlated with differentiation grade, TNM stage, and lymph node metastases, indicating that lncRNA CCAT1 might be involved in the carcinogenesis and metastasis of BC. Furthermore, Kaplan-Meier analysis with the log-rank test indicated that high lncRNA CCAT1 expression had a significant impact on overall survival and progression-free survival. In a multivariate Cox model, our results demonstrated that lncRNA CCAT1 expression was an independent poor prognostic factor for both 5-year overall survival and progression-free survival, suggesting that high lncRNA CCAT1 level might be a promising non-invasive biomarker for prognosis of patients with BC.

In conclusion, we demonstrated that the upregulation of lncRNA CCAT1 may be correlated with aggressive disease progression and poor prognosis of BC patients, suggesting that lncRNA CCAT1 might be involved in breast cancer carcinogenesis and become a potential prognostic marker for BC patients. Further studies of the molecular mechanisms by which lncRNA CCAT1 contributes to the initiation and progression of BC are warranted.

Disclosure of conflict of interest

None.

Address correspondence to: Dr. Ting Liu, Department of Radiology, First Affiliated Hospital, China Medical University, Shenyang 110001, China. E-mail: tliu1973@sina.com

References

[1] DeSantis C, Ma J, Bryan L and Jemal A. Breast cancer statistics, 2013. CA Cancer J Clin 2014; 64: 52-62.
[2] Autier P, Boniol M, Gavin A and Vatten LJ. Breast cancer mortality in neighbouring European countries with different levels of screening but similar access to treatment: trend analysis of WHO mortality database. BMJ 2011; 343: d4411.
[3] Fan L, Strasser-Weippl K, Li JJ, St Louis J, Finkelstein DM, Yu KD, Chen WQ, Shao ZM and Goss PE. Breast cancer in China. Lancet Oncol 2014; 15: e279-289.
[4] Mattick JS and Makunin IV. Non-coding RNA. Hum Mol Genet 2006; 15 Spec No 1: R17-29.
[5] Mercer TR, Dinger ME and Mattick JS. Long non-coding RNAs: insights into functions. Nat Rev Genet 2009; 10: 155-159.
[6] Batista PJ and Chang HY. Long noncoding RNAs: cellular address codes in development and disease. Cell 2013; 152: 1298-1307.
[7] Sanchez Y and Huarte M. Long non-coding RNAs: challenges for diagnosis and therapies. Nucleic Acid Ther 2013; 23: 15-20.
[8] Prensner JR and Chinnaiyan AM. The emergence of lncRNAs in cancer biology. Cancer Discov 2011; 1: 391-407.
[9] Nissan A, Stojadinovic A, MitraniRosenbaum S, Halle D, Grinbaum R, Roistacher M, Bochem A, Dayanc BE, Ritter G and Gomceli I. Colon

cancer associated transcript1: A novel RNA expressed in malignant and premalignant human tissues. Int J Cancer 2012; 130: 1598-1606.
[10] Yang F, Xue X, Bi J, Zheng L, Zhi K, Gu Y and Fang G. Long noncoding RNA CCAT1, which could be activated by c-Myc, promotes the progression of gastric carcinoma. J Cancer Res Clin Oncol 2013; 139: 437-445.
[11] Ma MZ, Chu BF, Zhang Y, Weng MZ, Qin YY, Gong W and Quan ZW. Long non-coding RNA CCAT1 promotes gallbladder cancer development via negative modulation of miRNA-218-5p. Cell Death Dis 2015; 6: e1583.
[12] Deng L, Yang SB, Xu FF and Zhang JH. Long noncoding RNA CCAT1 promotes hepatocellular carcinoma progression by functioning as let-7 sponge. J Exp Clin Cancer Res 2015; 34: 18.
[13] Spizzo R, Almeida MI, Colombatti A and Calin GA. Long non-coding RNAs and cancer: a new frontier of translational research? Oncogene 2012; 31: 4577-4587.
[14] Yang Z, Zhou L, Wu LM, Lai MC, Xie HY, Zhang F and Zheng SS. Overexpression of long non-coding RNA HOTAIR predicts tumor recurrence in hepatocellular carcinoma patients following liver transplantation. Ann Surg Oncol 2011; 18: 1243-1250.
[15] Shi Y, Li J, Liu Y, Ding J, Fan Y, Tian Y, Wang L, Lian Y, Wang K and Shu Y. The long noncoding RNA SPRY4-IT1 increases the proliferation of human breast cancer cells by upregulating ZNF703 expression. Mol Cancer 2015; 14: 51.
[16] Gupta RA, Shah N, Wang KC, Kim J, Horlings HM, Wong DJ, Tsai MC, Hung T, Argani P, Rinn JL, Wang Y, Brzoska P, Kong B, Li R, West RB, van de Vijver MJ, Sukumar S and Chang HY. Long non-coding RNA HOTAIR reprograms chromatin state to promote cancer metastasis. Nature 2010; 464: 1071-1076.
[17] Shi S, Wang L, Yu B, Li Y, Jin Y and Bai X. LncRNA-ATB promotes trastuzumab resistance and invasion-metastasis cascade in breast cancer. Oncotarget 2015; 6: 11652-63.
[18] Alaiyan B, Ilyayev N, Stojadinovic A, Izadjoo M, Roistacher M, Pavlov V, Tzivin V, Halle D, Pan H, Trink B, Gure AO and Nissan A. Differential expression of colon cancer associated transcript1 (CCAT1) along the colonic adenoma-carcinoma sequence. BMC Cancer 2013; 13: 196.
[19] Xiang JF, Yin QF, Chen T, Zhang Y, Zhang X-O, Wu Z, Zhang S, Wang HB, Ge J and Lu X. Human colorectal cancer-specific CCAT1-L lncRNA regulates long-range chromatin interactions at the MYC locus. Cell Res 2014; 24: 513-531.
[20] He X, Tan X, Wang X, Jin H, Liu L, Ma L, Yu H and Fan Z. C-Myc-activated long noncoding RNA CCAT1 promotes colon cancer cell proliferation and invasion. Tumour Biol 2014; 35: 12181-12188.
