# Supplementary material for: Expression of Concern: Prognostic value of long non-coding RNA CCAT1 expression in patients with cancer: A meta-analysis
Source: PLoS One. 2023 Apr 20;18(4):e0284940. doi: 10.1371/journal.pone.0284940 (PMC10118116; doi:10.1371/journal.pone.0284940)
Supplement: S1 File — (ZIP) [file pone.0284940.s001.zip › 3 The 11 included studies in PDF/9.pdf]

Downloaded from http://www.jci.org on February 26, 2017. https://doi.org/10.1172/JCI83265



# CCAT1 is an enhancer-templated RNA that predicts BET sensitivity in colorectal cancer

**Mark L. McCleland,[1] Kathryn Mesh,[1] Edward Lorenzana,[2] Vivek S. Chopra,[1] Ehud Segal,[2] Colin Watanabe,[3] Benjamin Haley,[4] Oleg Mayba,[3] Murat Yaylaoglu,[1] Florian Gnad,[3] and Ron Firestein[1]**

[1]Department of Pathology, [2]Department of Translational Oncology, [3]Department of Bioinformatics and Computational Biology, and [4]Department of Molecular Biology, Genentech Inc., South San Francisco, California, USA.

**Colon tumors arise in a stepwise fashion from either discrete genetic perturbations or epigenetic dysregulation. To uncover the key epigenetic regulators that drive colon cancer growth, we used a CRISPR loss-of-function screen and identified a number of essential genes, including the bromodomain and extraterminal (BET) protein BRD4. We found that BRD4 is critical for colon cancer proliferation, and its knockdown led to differentiation effects in vivo. JQ1, a BET inhibitor, preferentially reduced growth in a subset of epigenetically dysregulated colon cancers characterized by the CpG island methylator phenotype (CIMP). Integrated transcriptomic and genomic analyses defined a distinct superenhancer in CIMP⁺ colon cancers that regulates *cMYC* transcription. We found that the long noncoding RNA colon cancer–associated transcript 1 (CCAT1) is transcribed from this superenhancer and is exquisitely sensitive to BET inhibition. Concordantly, *cMYC* transcription and cell growth were tightly correlated with the presence of CCAT1 RNA in a variety of tumor types. Taken together, we propose that CCAT1 is a clinically tractable biomarker for identifying patients who are likely to benefit from BET inhibitors.**

## Introduction

Colorectal carcinoma (CRC) is one of the most prevalent and fatal types of cancers, accounting for over half a million deaths worldwide annually (1). Genomic analyses of colorectal tumors have uncovered a number of key somatic and germline mutations that drive tumorigenesis at the molecular level and can be linked to well-defined disease stages of tumor progression (2–4). Colorectal tumors can be divided into three main subtypes on the basis of these initiating molecular alterations: (a) chromosomal instability (CIN), (b) CpG island methylator phenotype (CIMP), and (c) microsatellite instability (MSI) (5–7). Sixty percent of colon cancers arise from the CIN pathway and are distinguished by aneuploidy and recurrent chromosomal amplifications at distinct genomic loci. A number of tumor-suppressor genes (*APC*, 5q21; *DCC*, 18q21) and oncogenes (*cMYC*, 8q24; *MET*, 7q; *CDK8*, *CDX2,* 13q; *PRPF6*, 20q) have been shown to reside in these regions of copy number alterations (8–13).

Recent genomic analyses have uncovered epigenetic alterations as major drivers of tumorigenesis (14, 15). In colon cancer, dysregulation of the epigenome has been recognized to occur at both the histone and DNA methylation levels (16, 17). The Wnt effector protein β-catenin associates with a number of histone-modifying enzymes such as the histone acetyltransferases CBP and p300 and the arginine methyltransferase PRMT2 (18, 19). In addition, Wnt pathway genes themselves are repressed via hypermethylation, further implying that Wnt signaling may be disrupted through epigenetic targeting at different levels (20–22). Aberrant DNA hypermethylation has long been recognized as an important etiological cause of tumorigenesis (23, 24). In colon tumors, methylation of the MMR gene *MLH1* has been found as an alternative pathway for the formation of MSI-high colon cancer (25). Widespread CpG island hypermethylation underscores a distinct pathway in colon cancer pathogenesis termed CIMP (7). Tumors arising through the CIMP pathway comprise 20% of colorectal cancers and are characterized by poor patient outcomes. Significant attention has been paid to the role of DNA hypermethylation in epigenetically mediated gene silencing and its significance in colon cancer initiation (26, 27). However, it is not clear whether these epigenetic targets can be harnessed for therapeutic purposes.

With recent findings in epigenetics research, it is now clear that DNA methylation and histone modification are reversible processes that can be targeted for therapeutic intervention using small-molecule inhibitors of the epigenetic writers (methyltransferases, acetyltransferases, kinases), readers (bromodomain- or chromodomain-containing genes), and erasers (demethylases, deacetylases, phosphatases) (28–31). For example, the histone acetyl-lysine reader BRD4 can be targeted for inhibition using drugs that disrupt bromodomain binding to acetylated histones (32, 33). Such drugs are showing promising responses in clinical trials, underscoring the need for additional efforts to identify and characterize epigenetic regulators that may be therapeutically tractable (33).

In this study, we developed an arrayed epigenetic CRISPR library and performed a high-throughput screen to identify epigenetic modulators in colon cancer (34–36). We identified a number of essential epigenetic regulators including BRD4. We show that BRD4 inhibition leads to growth arrest and differentiation in the epigenetically dysregulated CIMP⁺ class of tumors. CIMP⁺ colon cancers were found to be exquisitely dependent on bromodomain and extraterminal (BET) activity for *cMYC* transcription. An integrated transcriptomic and ChIP-sequencing (ChIP-seq)

**Conflict of interest:** All authors were employed by Genentech Inc. during the time this study was conducted.

**Submitted:** June 12, 2015; **Accepted:** November 18, 2015.
**Reference information:** *J Clin Invest*. 2016;126(2):639–652. doi:10.1172/JCI83265.

Downloaded from http://www.jci.org on February 26, 2017. https://doi.org/10.1172/JCI83265



analysis identified colon cancer–associated transcript 1 (CCAT1, also known as LOC100507056) as a distinct long noncoding RNA (lncRNA) transcribed off the *cMYC* superenhancer in colon cancer. Strikingly, we found that CCAT1 expression predicted JQ1 sensitivity and BET-mediated *cMYC* regulation. These results suggest a novel diagnostic methodology to identify *cMYC*-driven tumors that rely on BET for transcription, which could be translated into a promising strategy for patient selection in clinical trials.

## Results

*An arrayed CRISPR loss-of-function screen identifies BRD4 as a critical regulator of colon cancer growth.* To identify epigenetic regulators critical for colon cancer growth, we performed a CRISPR-based loss-of-function screen in the near-diploid RKO colon cancer cell line. Introduction of the Cas9 nuclease and guide RNAs (gRNAs) was performed in a 2-step process (Figure 1A). We used lentivirus to generate RKO cells that stably expressed Cas9 and subsequently transduced cells with relevant gRNAs. To examine the specificity and validate this CRISPR system, RKO (WT) and RKO-Cas9 cells were infected with gRNAs targeting the firefly luciferase gene and the mitotic kinase *PLK1*. Overall, five independent gRNAs targeting *PLK1* consistently reduced cell viability in the Cas9-expressing cells but did not hinder the growth of cells lacking Cas9 (Figure 1B). Notably, 3 nontargeting luciferase gRNAs did not impact cell proliferation.

Given the observed proficiency of gene KOs, a gRNA library targeting the 5′ exons of over 200 genes involved in epigenetic regulation (Epi200 library) was designed with a median coverage of 5 gRNAs per gene (Supplemental Table 1; supplemental material available online with this article; doi:10.1172/JCI83265DS1). High-throughput cloning and viral production were used to construct an arrayed lentiviral library containing over 1,000 gRNAs. The library was subsequently transduced into RKO-Cas9 cells, infected cells were selected with puromycin 48 hours after infection, and cell viability was measured 7 days after transduction (Figure 1, C and D). Our analysis of the CRISPR screen identified 12 genes that affected colon cancer proliferation using the criteria that at least 1 gRNA must have a *Z*-score of less than –2.0, and a second gRNA must have a *Z*-score of less than –1.5 for any given gene (Figure 1D and Supplemental Table 2). Among these genes are known cell-cycle regulators such as *PLK1*, *cMYC*, *AURKB*, and *HDAC1* (37).

In order to validate genetic hits from the screen, we correlated the phenotypic effects with genotypic activity for each set of gRNAs. Robust gRNA-mediated protein depletion was detected for BRD4, KAT8, CHD1, HDAC1, and AURKB by both immunoblot and immunofluorescence microscopy and positively correlated with the observed cell growth effects (Figure 1, E and F, and Supplemental Figure 1, A–D). Strikingly, more than half of all gRNAs tested by immunoblotting ($n$ = 39, from 8 genes) were able to reduce the targeted protein by more than 95% (Supplemental Figure 1E). As previous CRISPR screens have focused on pool-based approaches, these data illustrate the utility of the CRISPR system in an array-based format to provide efficient protein KO and consistent phenotypic effects (38–40).

*The long isoform of BRD4 is critical for colon cancer cell proliferation.* Among the novel candidate therapeutic targets that we identified, BRD4 was particularly attractive to pursue, as BRD4 small-molecule inhibitors have entered clinical trials for several hematological malignancies (41). As investigations of BRD4 in colon tumors have been limited (42, 43), we further examined BRD4 activity in colon cancer. Two alternatively spliced *BRD4* transcript variants have been described: a long-isoform BRD4 (*BRD4-LF*) and a short-isoform BRD4 (*BRD4-SF*) (44, 45). We first characterized BRD4 expression by IHC in colon tissues (normal, premalignant, and carcinoma) using Abs that recognized both BRD4 isoforms or specifically the BRD4-LF variant. We found that overall BRD4 levels remained unchanged at different stages of colonic tumorigenesis, while the BRD4-LF isoform was specifically upregulated during the premalignant-to-malignant transition (adenoma to carcinoma; Figure 2, A and B). Concordantly, BRD4-LF was consistently expressed in colonic cancer cell lines compared with BRD4-SF expression (Figure 2C). While both BRD4 isoforms encode bromodomains, BRD4-LF has been more strongly implicated in transcriptional regulation due to its C-terminal positive transcription elongation factor b–binding (p-TEFb–binding) motif (46, 47).

To further dissect BRD4 function in colon cancer, we used CRISPR to induce N-terminal deletions of BRD4 in RKO and HCT 116 colon cancer cells (Figure 2D). Clonally derived cell lines nullizygous for BRD4 displayed substantial growth retardation in both cancer cell lines (Figure 2, E and F). Consistent with previous findings (48), BRD4-null cells were marked by cell-cycle defects consistent with a G1/S-phase delay (Figure 2G). Since our initial findings showed that isoform-specific BRD4 expression was dysregulated during colon tumorigenesis (Figure 2, A and B), we reconstituted isoform-specific BRD4 expression by delivery of BRD4-LF or BRD4-SF splice variants to BRD4-null colon cancer cells. While BRD4-SF was unable to restore growth, expression of BRD4-LF rescued the growth defect of BRD4-KO cells to WT levels (Figure 2, H and I, and Supplemental Figure 2A). BRD4 constructs containing bromodomain-inactivating mutations failed to rescue the growth defect. To directly test the importance of the p-TEFb–binding C-terminal motif (CTM) of BRD4-LF, we used CRISPR to ablate the BRD4-LF isoform in colon cancer cells. Analysis of 4 independent BRD4-LF–specific nullizygous HCT 116 cell lines showed reduced cell proliferation (Figure 2, J and K). These data underscore an important role for the BRD4-LF in colon cancer cell proliferation.

*BRD4 loss reduces colon tumor growth and induces cellular differentiation in vivo.* BRD4 and, in particular, the BRD4-LF variant have been implicated in maintaining embryonic pluripotency. Likewise, in cancer, BRD4 inhibition has been shown to induce differentiation effects in hematological malignancies (49, 50). To characterize the effect of acute BRD4 knockdown on colon tumor growth in vivo, we used a doxycycline-inducible shRNA system to reduce BRD4 in implanted tumors (51). Efficient BRD4 knockdown and the consequent in vitro growth defects were found with 2 independent BRD4 shRNAs in HT-29 and HCT 116 cell lines (Supplemental Figure 2, B and C). Colon cancer cell lines expressing the more potent shBRD4-2 shRNA were then used for in vivo tumor xenograft efficacy studies. To measure the effects on tumor growth rather than initiation, mice were administered doxycycline only after tumors reached 200 $mm^3$ in size. shBRD4-2 tumors treated

Downloaded from http://www.jci.org on February 26, 2017. https://doi.org/10.1172/JCI83265



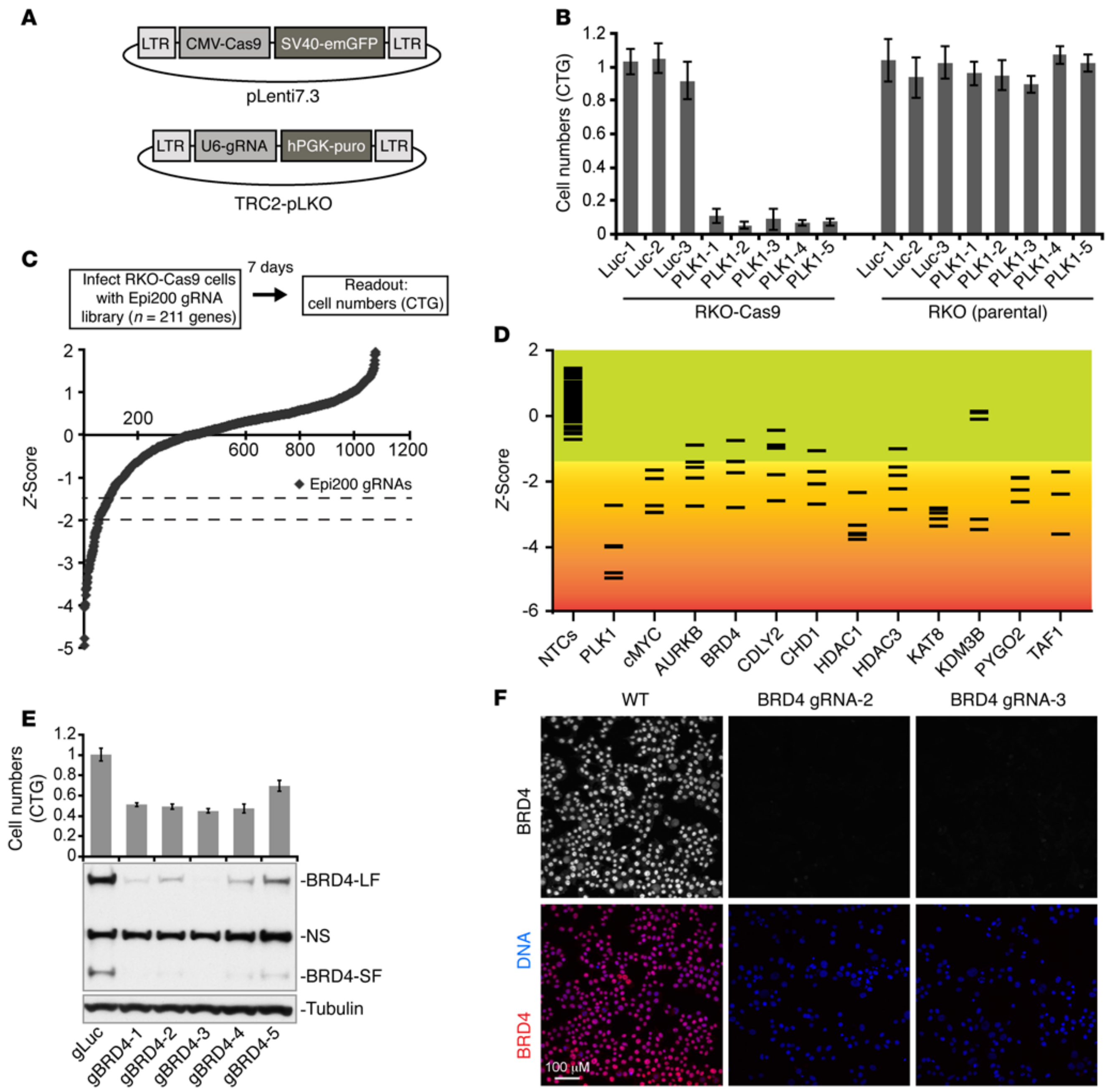


**Figure 1. An arrayed CRISPR screen identifies BRD4 as a regulator of colon cancer.** (**A**) Schematic diagram of lentiviral expression vectors used to express Cas9 and gRNA. (**B**) Cell viability was measured in parental RKO or RKO-Cas9 stably expressing cells 7 days after transduction with gRNAs targeting luciferase or PLK1. Data represent the mean ± SD of 3 replicates. (**C**) Schematic of the CRISPR negative-selection screen conducted in RKO-Cas9 cells using an arrayed gRNA library designed and synthesized to target 211 genes involved in epigenetic regulation and cancer (Epi200). Distribution curve shows *Z*-scores for cell viability for all gRNAs in the Epi200 CRISPR library. Dashed lines (*Z*-scores, –1.5 and –2.0) indicate the cutoffs used for determining whether a gRNA scored as a hit. (**D**) Scatter gram depicts *Z*-score values for each gRNA of the genes that scored as hits. Bars represent individual gRNAs. gRNA NTCs are shown for reference. (**E**) Bar graph shows cell viability effects after BRD4 KO using 5 independent gRNAs. Data represent the mean ± SD of 3 replicates. Accompanying immunoblots show the level of BRD4 depletion 4 days after transduction. (**F**) Validation of gRNA-mediated BRD4 KO using immunofluorescence. Two independent gRNAs are shown. Scale bar: 100 μm.

with doxycycline showed efficient BRD4 depletion in vivo and, importantly, displayed significant tumor regression in both colon tumor xenograft models (Figure 3, A and B, and Supplemental Figure 2D). To characterize the nature of the defect, we performed IHC for cell-cycle (phosphorylated histone H3 [p-H3]) and apoptosis (cleaved caspase 3) markers on tumor xenografts 7 days after doxycycline-induced BRD4 knockdown (Figure 3, C and D). Consistent with a cell-cycle defect, tumors with reduced BRD4 levels showed decreased p-H3 ($P = 0.0065$), with no significant change in cleaved caspase 3 (Figure 3, C and D). We also observed a marked decrease in cMYC ($P < 0.0001$), a direct BRD4 transcriptional target in other cancers (Figure 3E). Histopathological analysis of HT-29

Downloaded from http://www.jci.org on February 26, 2017. https://doi.org/10.1172/JCI83265



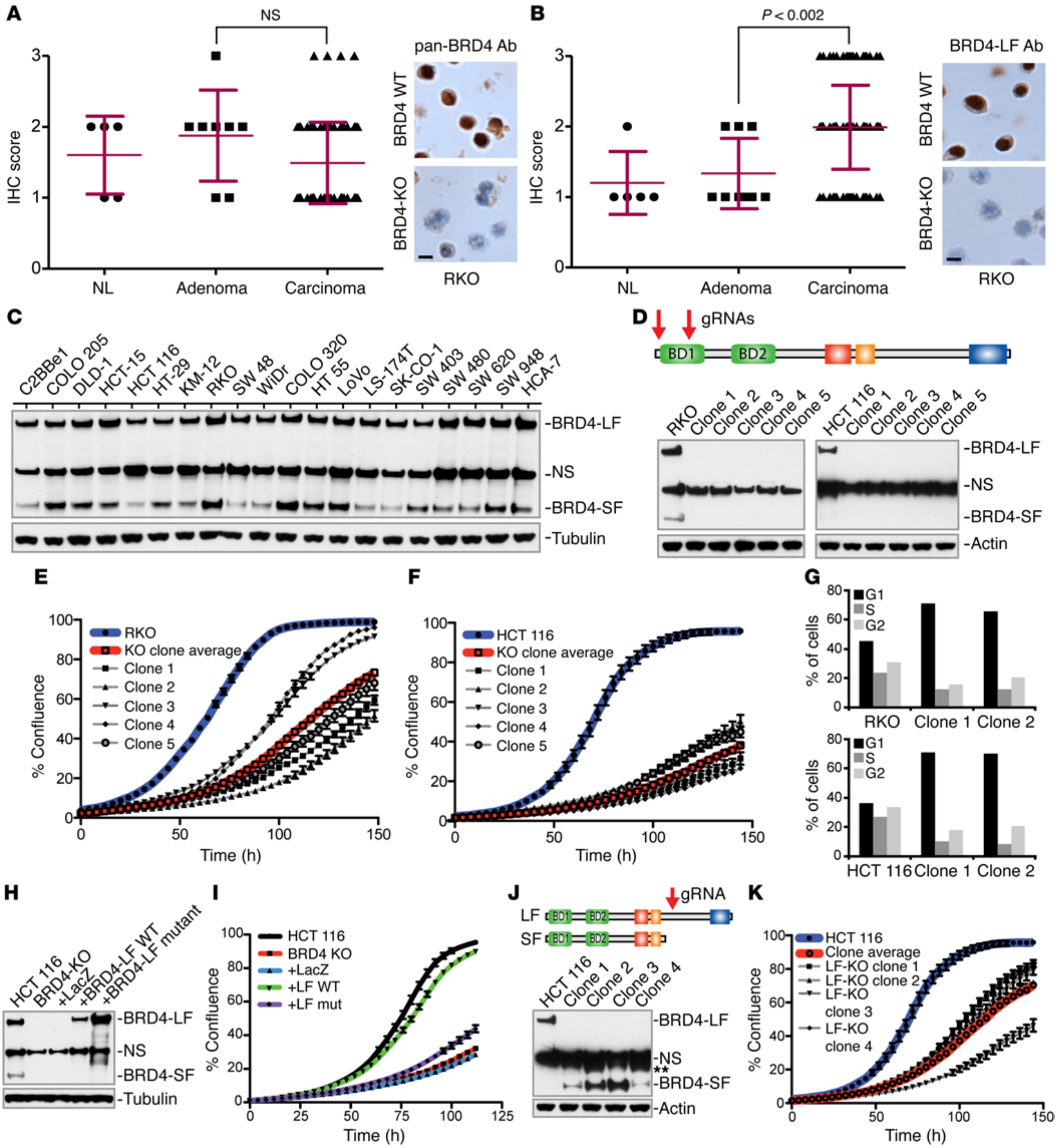


**Figure 2. BRD4-LF is essential for colon cancer growth.** (**A** and **B**) BRD4 IHC of normal colon (NL), colonic adenoma, and colonic carcinoma using either a pan-isoform BRD4 (**A**) or a BRD4-LF–specific (**B**) Ab. Photomicrographs illustrate target specificity using WT or BRD4-KO RKO cells. Data represent the mean ± SD. Statistical comparisons in **A** and **B** were made using a 2-tailed Student's *t* test. Scale bars: 10 μm (**A** and **B**). (**C**) Immunoblot analysis of isoform-specific BRD4 expression in colon cancer cell lines. (**D**) Generation of clonal BRD4-KO RKO and HCT 116 cells. Schematic illustrates the location of the gRNAs. Immunoblot analysis of multiple BRD4-KO clones is shown. (**E** and **F**) IncuCyte analysis was used to quantify cell proliferation of RKO (**E**) and HCT 116 (**F**) BRD4-KO cells. Blue line represents parental cells; gray lines represent an individual clone's growth; and red line represents average clonal growth. Error bars represent the SEM. (**G**) FACS-based cell-cycle analysis of RKO and HCT 116 parental and BRD4-KO cells. (**H** and **I**) Expression of the BRD4-LF–rescued (LF WT) cell proliferation defects in HCT 116 BRD4-KO cells. Expression of LacZ or a BRD4-LF containing inactivating mutations in both bromodomains (LF mut) failed to rescue the phenotype. (**H**) Immunoblot of stably expressing cell lines in **I**. (**J** and **K**) Generation of clonal HCT 116 cells that lacked the C-terminal domain of BRD4. (**J**) Schematic illustrates the location of the gRNA. Immunoblot highlights 4 resulting BRD4 clones with deleted C-termini. BRD4-LF truncated products are highlighted by a double asterisk. (**K**) IncuCyte analysis was used to quantify cell proliferation of HCT 116 cells containing BRD4 C-terminal deletions. Blue line represents parental cells; gray lines represent an individual clone's growth; and red line represents average clonal growth. Error bars represent the SEM.

Downloaded from http://www.jci.org on February 26, 2017. https://doi.org/10.1172/JCI83265



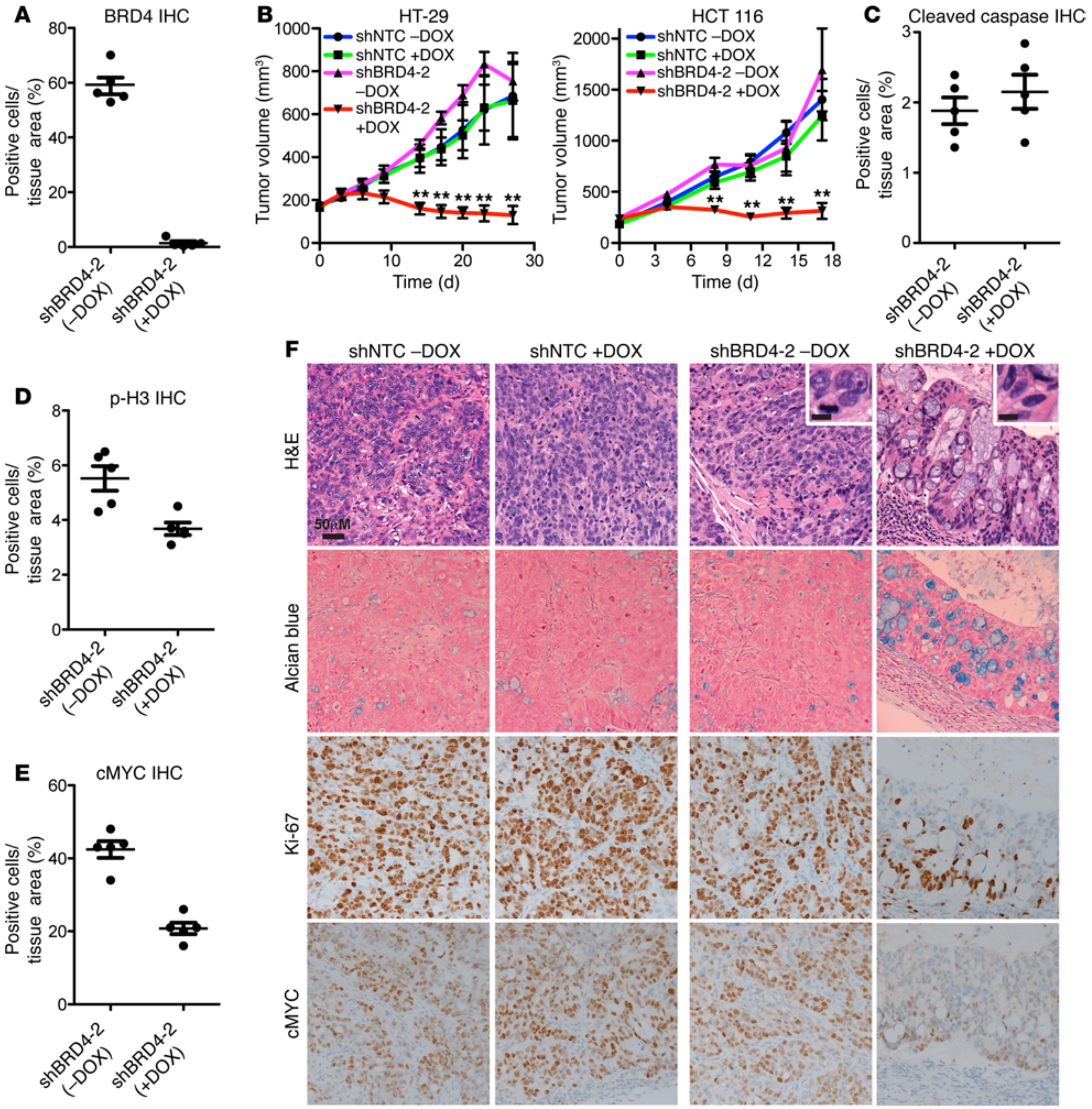


**Figure 3. BRD4 knockdown in colon xenograft reduces tumor growth and induces differentiation.** (**A**) Quantification of BRD4 depletion in xenograft tumors 7 days after doxycycline administration. (**B**) Line graphs show tumor volumes from xenografted HCT 116 and HT-29 cells measured over time ($n$ = 10 mice/group). shRNA expression was induced with doxycycline. $^{**}P < 0.05$, by 2-tailed Student's $t$ test. (**C**) Quantification of apoptosis using cleaved caspase 3 IHC following BRD4 knockdown in HT-29 xenograft tumors. (**D**) Quantification of mitotic index using p-H3 IHC following BRD4 knockdown in HT-29 xenograft tumors. (**E**) Quantification of cMYC by IHC following BRD4 knockdown in HT-29 xenograft tumors. (**F**) Histological analysis of HT-29 tumors following shNTC or shBRD4 induction. H&E, Alcian blue, Ki-67, and cMYC staining are shown. Scale bars: 50 μm and 10 μm (insets). All data represent the mean ± SEM. DOX, doxycycline.

xenograft tumors revealed striking morphological alterations upon BRD4 knockdown. These include the formation of crypt-like structures with goblet cell differentiation and loss of cancer-associated cytological features such as prominent nucleoli and pleomorphic nuclei (Figure 3F; H&E-stained inset images). Immunophenotypical analysis showed that these pseudo-crypts mimic the ordered structure of intestinal epithelium, expressing proliferative markers such as Ki-67 and cMYC in a graded manner (Figure 3F; Ki-67 and cMYC, with higher expression seen at the pseudo-crypt base). Together, these data show that BRD4 is required for tumor growth and maintenance of a dedifferentiated state in vivo.

*BET inhibitors preferentially impair CIMP⁺ colon cancer growth.* To explore the utility of BET inhibition more broadly in colon cancer, we tested the effects of JQ1, a BET small-molecule inhibitor, in a panel of 20 colon cancer cell lines with similar proliferation rates (20–40 hours) (32). We found that a subset of colon cell lines was

Downloaded from http://www.jci.org on February 26, 2017. https://doi.org/10.1172/JCI83265



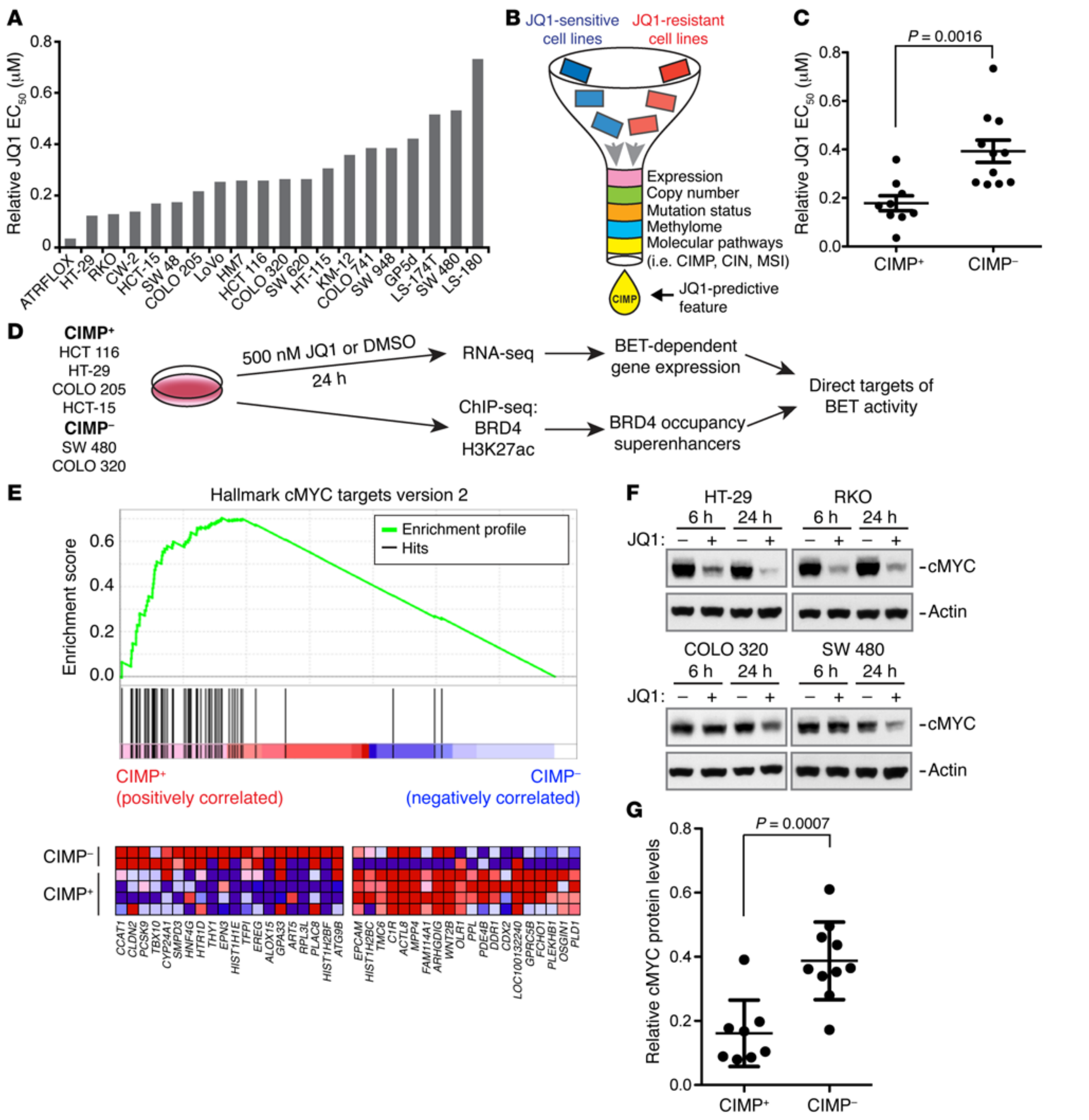


**Figure 4. BET pharmacological inhibition preferentially affects the growth of CIMP⁺ colon cancers by downregulating cMYC expression.** (**A**) Relative $EC_{50}$ values in colon cancer cells following JQ1 treatment for 3 days. (**B**) Schematic illustrating the genomic tools and features used for predicting colon cancer sensitivity to BET inhibition. (**C**) Relative JQ1 $EC_{50}$ values in colon cancer cells sorted by CIMP status. Bars represent the mean, error bars represent the SEM, and each dot represents a single cell line. (**D**) Schematic of the RNA-seq and ChIP-seq experiments and analysis pipeline. (**E**) GSEA analysis of JQ1-regulated genes revealed an enrichment of cMYC targets in CIMP⁺ cell lines. Heatmap of top-20 genes differentially regulated by JQ1 in CIMP⁺ versus CIMP⁻ cell lines. $\log_2$ fold-change values are represented as colors, where the range of colors shows the range of values (red, highest value; blue, lowest value). (**F**) Colon cancer cell lines were treated for 6 or 24 hours with DMSO or 1 μM JQ1, and cMYC levels were subsequently examined by immunoblotting. (**G**) Comparison of cMYC protein depletion in CIMP⁺ and CIMP⁻ colon cell lines following treatment with 1 μM JQ1 for 24 hours. Bars represent the average, error bars represent the SD, and each dot represents an individual cell line. Statistical comparisons were made using a 2-tailed Student's *t* test.

distinctly sensitive to JQ1 (Figure 4A and Supplemental Table 3). To investigate the molecular underpinnings of BET inhibitor sensitivity in these cells, we surveyed genomic features that are predictive of JQ1 sensitivity on the basis of the Lasso shrinkage and selection method for linear regression (Figure 4B, Supplemental Figure 3A, and ref. 52). Using copy number, mutation, DNA methylation, and expression data as well as other classifications such as MSI and CIMP status, we generated 88,723 features for each cell line. The Lasso approach selected CIMP status as the most predictive feature to classify lines as sensitive or resistant (Sup-

Downloaded from http://www.jci.org on February 26, 2017. https://doi.org/10.1172/JCI83265



plemental Figure 3B and ref. 53). Strikingly, the 6 most sensitive cell lines were all CIMP$^+$, while the 6 most resistant cell lines were all CIMP$^-$ (Figure 4, A and C). DNA hypermethylation at promoter CpG islands of *POU3F2*, *FAM65B*, and *GABRB2* was highly correlated with JQ1 sensitivity (Supplemental Figure 3C), and each gene was selected as a predictive feature. Consistent with this finding, repression of *ZNF606*, a known CpG hypermethylated gene (54), was also a predictive feature and exhibited promoter methylation (Supplemental Figure 3D). We confirmed these findings using I-BET-762, an additional BET inhibitor with clinical activity (55). Similar to JQ1, I-BET-762 showed greater activity in CIMP$^+$ cell lines (Supplemental Figure 4, A and B). The JQ1 and I-BET-762 half-maximal effective concentration ($EC_{50}$) values correlated well with each other, implying that these effects are on target (Supplemental Figure 4C). These data suggest that CIMP$^+$ colon cancers are more dependent on BET activity.

To identify direct BET-dependent target genes that may account for the increased sensitivity seen in CIMP$^+$ colon cancer cells, we profiled 4 CIMP$^+$ and 2 CIMP$^-$ cells for both JQ1-dependent gene expression changes and BRD4 and histone H3 lysine 27 acetyl (H3K27ac) genomic enrichment using RNA-seq and ChIP-seq, respectively (Figure 4D, Supplemental Figure 5, and Supplemental Tables 4 and 5). Strikingly, gene set enrichment analysis (GSEA) comparing JQ1 transcriptomic changes across all 6 cell lines revealed significant enrichment of cMYC pathway gene signatures in the CIMP$^+$ cells (FDR < 0.0001) (Figure 4E and Supplemental Figure 6). A number of important colon cancer–associated oncogenic regulators, including *EREG* and *CLDN2*, showed CIMP status–specific JQ1 changes in expression levels (Figure 4E).

As cMYC plays a critical role in colon cancer initiation and progression (56, 57), we examined cMYC protein levels by immunoblot analysis after BET inhibition in colon cancer cell lines. We found that cMYC protein was dramatically reduced in CIMP$^+$ cell lines 24 hours after JQ1 treatment compared with levels in CIMP$^-$ colon cancers (Figure 4, F and G). Furthermore, we found that the kinetics of cMYC reduction also occurred earlier in the CIMP$^+$ cell lines, with the majority of cMYC depleted as early as 6 hours after JQ1 treatment (Supplemental Figure 7, A–C). To determine whether cMYC is a key mediator of BET activity in these cells, we used lentivirus to deliver exogenous cMYC in HCT 116 BRD4-null cells. We found that restoration of cMYC expression in a BRD4-deficient setting led to partial rescue of cell growth (Supplemental Figure 7D). We conclude that CIMP$^+$ cells are sensitive to loss of cMYC in a BET-dependent manner.

*CCAT1 is a BET target gene and marks the colon cMYC superenhancer*. Previous studies have shown that BRD4 preferentially binds at densely occupied enhancer elements termed superenhancers (58), which in some contexts can ramp up the expression of oncogenic factors in a tumor type–specific and lineage-dependent manner (58–60). To identify direct BRD4 targets, we integrated the transcriptomic (RNA-seq) and genomic (ChIP-seq) analyses to identify genes that were both downregulated after JQ1 treatment and marked by an adjacent superenhancer. This integrative approach was conducted for CIMP$^+$ and CIMP$^-$ cell lines independently. Three direct BET targets, *PHF15*, *TRIB3*, and *CCAT1* (Figure 5A), were identified by this analysis in the CIMP$^+$ cells. Of these 3 genes, only *TRIB3* and *CCAT1* were specific to the CIMP$^+$ colon cells (Figure 5A). Notably, the CCAT1 transcript was one of the most highly downregulated genes in CIMP$^+$ cells upon JQ1 treatment (Supplemental Table 4), and its genomic locus ranked as one of the most BRD4-enriched enhancers (i.e., "superenhancer") in CIMP$^+$ colon cancer cell lines (Figure 5B). The CCAT1 transcript is located within a demarcated superenhancer, 500 kb upstream of the *cMYC* promoter. Importantly, both BRD4 binding and H3K27ac levels were enriched at the CCAT1-associated superenhancer in CIMP$^+$ colon cancer cell lines compared with CIMP$^-$ cell lines, suggesting that its activation may be context specific (Figure 5C and Supplemental Figure 8).

CCAT1 is a lncRNA that is expressed in colon, gastric, and gallbladder cancers (61–63). While CCAT1 has been reported to regulate cMYC expression (63), its relationship to BET activity has not been previously reported. We found that basal CCAT1 RNA levels correlated with the amount of BRD4 binding and H3K27ac at the CCAT1-associated superenhancer (Figure 5, C and D, and Supplemental Figure 8, A–D). While CCAT1 expression was lowest in a subset of CIMP$^-$ cells that lacked the CCAT1 superenhancer, CIMP status did not predict CCAT1 expression across a wider panel of colon cancer cell lines ($P$ = 0.4; Supplemental Figure 11A, and Supplemental Table 3). In CCAT1-expressing cell lines, CCAT1 RNA levels were exquisitely sensitive to JQ1, suggesting that it is a direct BET transcriptional target (Figure 5E). Consistent with this superenhancer driving *cMYC* transcription, JQ1 treatment preferentially reduced *cMYC* expression in CCAT1-expressing cells (Figure 5F). Taken together, these results suggest that CCAT1 is a superenhancer template RNA that may serve as a beacon for identifying high BET activity near the *cMYC* locus.

BET inhibitors, including JQ1 and I-BET-762, bind and inhibit the bromodomains of BRD2, BRD3, BRD4, and BRDT. Moreover, genomic profiling suggests that these proteins may be partially redundant, as there is considerable overlap in their genomic binding sites in prostate cancer cells (64). To directly examine BRD4-specific effects as compared with BET-driven changes, we used whole-genome transcriptional profiling in HT-29 and HCT 116 cells after BRD4 knockdown or JQ1 inhibition (Supplemental Figure 9). Pharmacological inhibition with JQ1 induced a significantly greater number of differentially expressed genes compared with that seen with BRD4 knockdown using inducible shRNA constructs. Importantly, CCAT1 was downregulated upon JQ1 treatment, but not upon BRD4 knockdown (Supplemental Figure 9 and Supplemental Figure 10A). To validate that CCAT1 is a direct BET target gene, we examined CCAT1 levels following a dose titration of 2 chemically distinct BET inhibitors. CCAT1 levels exhibited a dose-dependent reduction in the presence of both JQ1 and I-BET-762 that closely mirrored the viability response to inhibitor (Supplemental Figure 10B and C). Taken together, these data suggest that CCAT1 is a direct BET target but is not exclusively regulated by BRD4.

*CCAT1, PCAT1, and LOC728724 are BET transcriptional targets and markers for cells dependent on BET-mediated cMYC transcription*. Growing evidence suggests that enhancers are actively transcribed by RNA polymerase II to produce enhancer-derived lncRNAs (eRNAs) (65, 66). In fact, BRD4 itself has been shown to regulate eRNA expression in murine cells (67). To explore the connection between eRNAs and BET activity at the *cMYC* locus, we asked whether other superenhancers adjacent to the *cMYC* locus express

Downloaded from http://www.jci.org on February 26, 2017. https://doi.org/10.1172/JCI83265



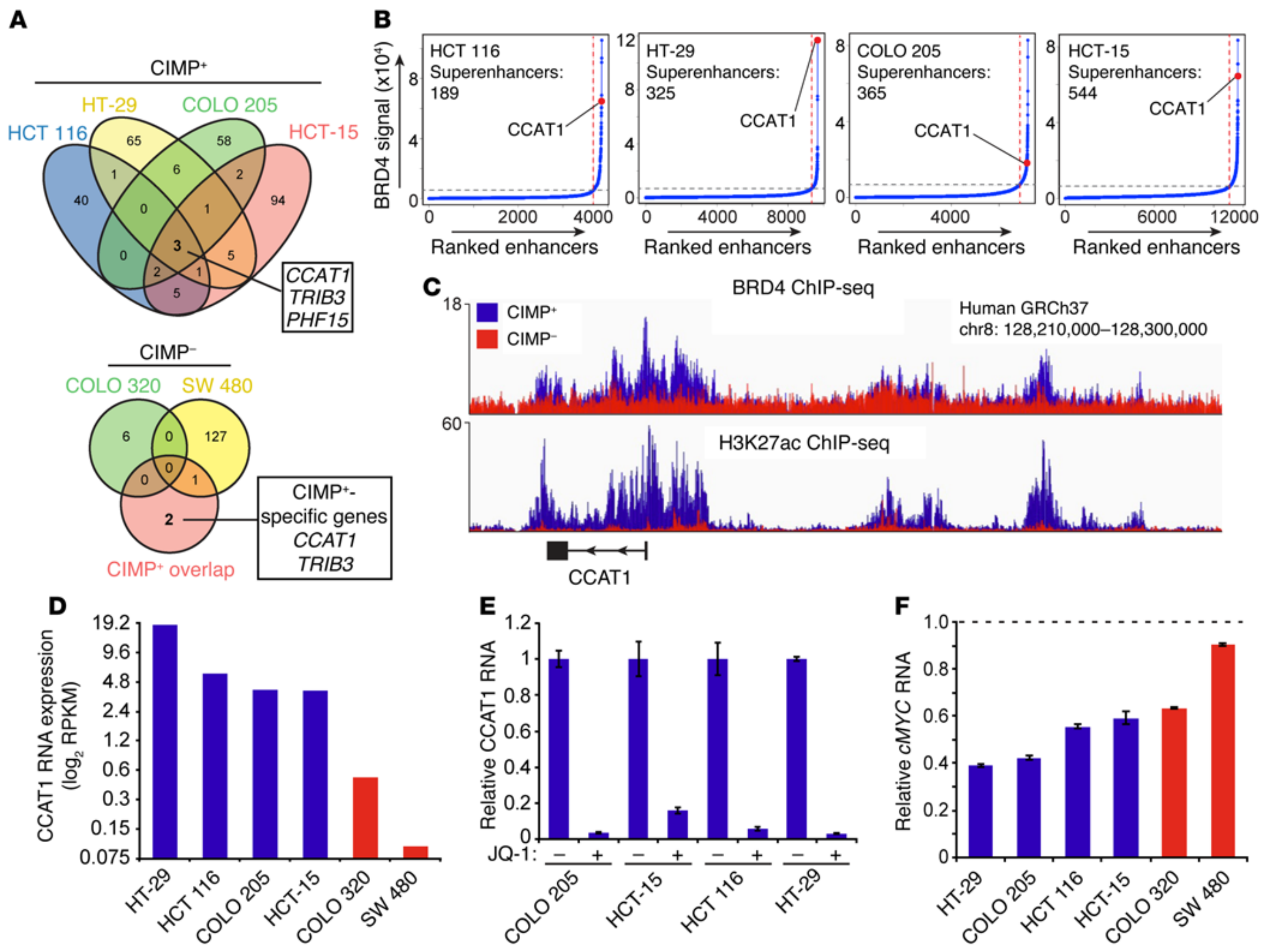


**Figure 5. Genomic approaches uncover CCAT1 as a direct BET transcriptional target and predictor of BET inhibitor sensitivity.** (**A**) Venn diagram shows the overlap of genes downregulated by JQ1 (>2-fold) and associated with a superenhancer across CIMP⁺ and CIMP⁻ cell lines. Superenhancer-associated, JQ1-regulated genes common to all CIMP⁺ cell lines are highlighted. (**B**) Distribution of BRD4 ChIP-seq signal across enhancers in CIMP⁺ cell lines. The *y* axis represents input-subtracted BRD4 signal; the *x* axis represents enhancers ranked by BRD4 signal intensity. Superenhancers were defined as enhancers that surpassed the inflection point. The CCAT1-associated superenhancer is highlighted. (**C**) Genome browser tracks showing input-normalized average BRD4 ChIP-seq signal across the CCAT1 locus for CIMP⁺ (blue) and CIMP⁻ (red) cell lines. The *y* axis represents BRD4/input coverage values averaged for CIMP⁺ (HT-29, COLO 205, HCT 116, HCT 15) or CIMP⁻ (SW 480, COLO 320) cell lines. (**D**) Basal RNA levels of CCAT1 in colon cell lines. RNA was measured by RNA-seq. Blue bars represent CIMP⁺ lines; red bars represent CIMP⁻ lines. (**E**) Relative CCAT1 expression in CIMP⁺ cells following treatment with 500 nM JQ1. Data are from RNA-seq analysis and represent the mean ± SD. (**F**) Relative *cMYC* expression in colon cancer cells following treatment with 500 nM JQ1. Data are from RNA-seq analysis and represent the mean ± SD. Blue bars represent CIMP⁺ lines; red bars represent CIMP⁻ lines.

eRNAs. Indeed, H3K27ac is associated with the presence of 2 distinct eRNAs, prostate cancer–associated transcript 1 (PCAT1) and LOC728724, in prostate and T cell acute lymphoblastic leukemia (T-ALL), respectively (Figure 6A and refs. 60, 64). Notably, both leukemia and prostate cells have been reported to be sensitive to BET inhibition. To determine whether the expression of these eRNAs is restricted to cancer type and superenhancer activation, we examined the expression of CCAT1, PCAT1, and LOC728724 across different cancer indications using RNA-seq data from The Cancer Genome Atlas (TCGA). Strikingly, these eRNAs showed distinct tumor specificity, with LOC728724 expression restricted to leukemia and PCAT1, showing preferential expression in prostatic carcinoma. Interestingly, while CCAT1 expression has been mainly linked to colon cancer, we found that it was also highly expressed in a subset of lung, pancreatic, and gastric tumors (Figure 6B).

To examine whether CCAT1, PCAT1, and LOC728724 are controlled by BRD4-associated superenhancers, we identified cell lines for each tumor type that expressed the CCAT1, PCAT1, or LOC728724 eRNAs (Supplemental Figure 11, A–D, and ref. 68). These cells were treated with JQ1 for 24 hours, and the level of each eRNA was assessed by quantitative PCR (qPCR). Strikingly, BET inhibition led to near-complete RNA reduction for each lineage-specific eRNA (Supplemental Figure 11, E–G). These data demonstrate that BET-dependent eRNAs are distinctly expressed in discrete tumor types and may serve as pharmacodynamic markers of BET inhibition.

While the mechanism by which cancers are dependent on BET for *cMYC* transcription is not well understood, the ability to detect this transcript is of great clinical importance. For example, in hematological malignancies, *cMYC* is an established BET

Downloaded from http://www.jci.org on February 26, 2017. https://doi.org/10.1172/JCI83265



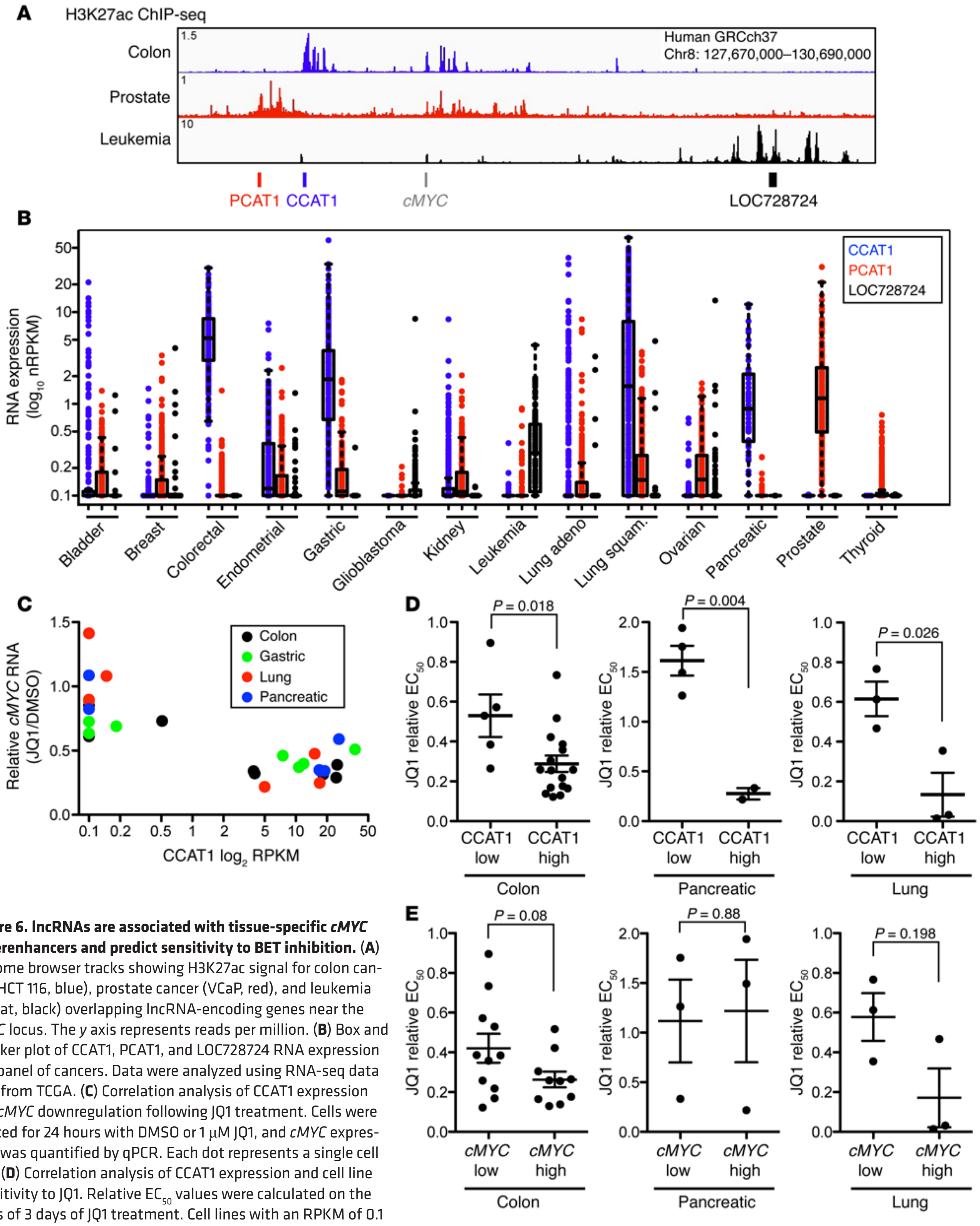


**Figure 6. lncRNAs are associated with tissue-specific *cMYC* superenhancers and predict sensitivity to BET inhibition.** (**A**) Genome browser tracks showing H3K27ac signal for colon cancer (HCT 116, blue), prostate cancer (VCaP, red), and leukemia (Jurkat, black) overlapping lncRNA-encoding genes near the *cMYC* locus. The *y* axis represents reads per million. (**B**) Box and whisker plot of CCAT1, PCAT1, and LOC728724 RNA expression in a panel of cancers. Data were analyzed using RNA-seq data sets from TCGA. (**C**) Correlation analysis of CCAT1 expression and *cMYC* downregulation following JQ1 treatment. Cells were treated for 24 hours with DMSO or 1 μM JQ1, and *cMYC* expression was quantified by qPCR. Each dot represents a single cell line. (**D**) Correlation analysis of CCAT1 expression and cell line sensitivity to JQ1. Relative $EC_{50}$ values were calculated on the basis of 3 days of JQ1 treatment. Cell lines with an RPKM of 0.1 or less were defined as $CCAT1^{lo}$, and cell lines with an RPKM of 1 or more were defined as $CCAT1^{hi}$. Each dot represents a single cell line. Data represent the mean ± SEM. (**E**) Correlation analysis of *cMYC* expression and cell line sensitivity to JQ1. $cMYC^{lo}$ and $cMYC^{hi}$ lines were defined as being above or below the median cMYC RPKM values within each cancer type, respectively. Each dot represents a single cell line. Data represent the mean ± SEM. Statistical comparisons were made using a 2-tailed Student's *t* test.

Downloaded from http://www.jci.org on February 26, 2017. https://doi.org/10.1172/JCI83265



target, while in solid tumor malignancies, BET-dependent *cMYC* transcription is often cell-line dependent (64, 69). Therefore, we asked whether eRNA expression could predict which cells utilize BET to modulate *cMYC* transcription. We measured *cMYC* RNA levels in CCAT1$^{hi}$ and CCAT1$^{lo}$ cell lines following a 24-hour JQ1 treatment and found that 14 of 16 CCAT1$^{hi}$ cell lines (CCAT1 reads per kilobase per million [RPKM] threshold >1.0) showed a 50% or greater reduction in *cMYC* levels. Conversely, JQ1 treatment only moderately affected *cMYC* levels in the 11 cell lines tested that expressed low (≤0.5 RPKM) CCAT1 eRNA levels (Figure 6C and Supplemental Figure 12A). A similar trend was observed in prostate and leukemia cell lines, in which PCAT1 and LOC728724 expression was associated with BET-mediated *cMYC* transcription, respectively (Supplemental Figure 12, B–E).

*cMYC* upregulation in cancer occurs via numerous independent molecular mechanisms, including superenhancer activation, locus amplification, posttranslational modification, and activation and mutation of oncogenic signaling pathways. If BET inhibitors preferentially influence superenhancer activity, we would predict that CCAT1 levels might be more accurate in predicting BET sensitivity than *cMYC* levels. To this end, we tested whether CCAT1 or *cMYC* RNA levels could predict cell-line sensitivity to JQ1. Colon, lung, gastric, and pancreatic cell lines were treated for 3 days with JQ1, and the relative $EC_{50}$ values were calculated. With the exception of gastric cancer, all other tumor types (pancreatic, lung, and colon) exhibited a significant correlation between CCAT1 expression and JQ1 sensitivity (Figure 6D and Supplemental Figure 13, A and B). Similar analyses showed no significant correlation between *cMYC* expression and JQ1 sensitivity (Figure 6E and Supplemental Figure 13C). As these data predicted, there was no significant correlation between CCAT1 and *cMYC* expression in this cell-line panel (Supplemental Figure 13D). While BET inhibitors were more effective in CCAT1-expressing cells, this result was not because CCAT$^+$ cells are more sensitive to *cMYC* downregulation. We knocked down *cMYC* in a panel of CCAT1$^+$ and CCAT1$^-$ cell lines with 2 independent shRNAs and found that both subsets were equally sensitive to *cMYC* reduction (Supplemental Figure 13, E and F). Taken together, we conclude that CCAT1 and other eRNAs may serve as predictive biomarkers to identify tumors that utilize BET-mediated *cMYC* transcription for tumor growth.

*Patients with colon tumors expressing high CCAT1 levels have a poor clinical outcome*. We hypothesize that CCAT1 expression may provide a clinical opportunity to identify tumors that rely on a superenhancer to drive cMYC expression. To address this concept, we developed a CCAT1 ISH assay (Figure 7A and Supplemental Figure 14, A and B) and examined CCAT1 expression directly in colon tumors (Figure 7B). CCAT1 expression was scored in a cohort of normal colon tissues ($n$ = 555) and colon tumors ($n$ = 705) with associated clinicopathological variables. Consistent with previous findings (63) and TCGA data on normal colon cancer (Supplemental Figure 14C), normal colon showed weak to no CCAT1 expression compared with expression levels in colorectal tumors (Figure 7B and Supplemental Figure 14D). In Kaplan-Meier analyses of 638 colon cancers, we found that both overall and colon cancer–specific 5-year patient survival rates were significantly lower in CCAT1$^{hi}$ (ISH score >1) tumors compared with survival rates for patients with CCAT1$^{lo}$ (ISH score 0–1) tumors (Figure 7C and Supplemental Figure 15A). To better understand the implications of CCAT1 expression, we assessed its expression according to clinical, pathological, and molecular characteristics known to be important in colon tumorigenesis (Table 1). We found that CCAT1 expression correlated with tumor grade (poor differentiation), tumor stage (stages III and IV), and nonmucinous histology. Interestingly, CCAT1 was associated with cMYC expression but was inversely correlated with cMYC amplification. We also performed multivariate testing to assess which variables were independently associated with outcomes (Supplemental Table 6). We found that CCAT1 was an independent prognostic indicator (Figure 7C) and was able to predict poor survival, independent of cancer stage (Supplemental Figure 15B). These data suggest that CCAT1 may have utility as an independent prognostic biomarker of patient outcomes. In summary, we propose that CCAT1 and other eRNAs may serve as clinical biomarkers to predict which cancers utilize BET activity to drive *cMYC* transcription and tumor growth (Figure 7D).

## Discussion

The importance of epigenetics in neoplasia has been cemented by the recent discovery that many epigenetic regulators are mutated or dysregulated in human tumors (70) and furthermore can be targeted for therapeutic purposes (71, 72). Here, we used the CRISPR technology to systematically characterize the epigenetic landscape and identify new therapeutic targets in colon cancer. Unlike other CRISPR-based loss-of-function screens that are based on pooled libraries, we developed and utilized an array-based CRISPR library that yielded robust and reproducible target depletion and phenotypic response. While advantages exist for both arrayed and pooled screening platforms (73), our screen represents an important step in characterizing CRISPR technology in an arrayed platform.

We believe that our work identifies a number of novel candidate therapeutic targets. BRD4 was particularly appealing to pursue, as BRD4 inhibitors have been entered into clinical trials for several hematological malignancies (41). Consistent with the oncogenic effects of BRD4 in other malignancies, we found that genetic or pharmacological inhibition of BRD4 reduced cancer proliferation and abrogated tumor growth in colon cancer xenograft models. BRD4 knockdown led to tumor differentiation in vivo, with formation of a crypt-like structure observed in the HT-29 xenografted model. Colon cancer has long been postulated to be a stem cell–driven disease. Consistently, Wnt and Notch pathway inhibitors lead to similar differentiation changes but have been clinically challenging due to accompanying toxicity issues (74). Our findings raise hope that BET inhibitors can offer a clinically tractable path for differentiation therapy in colon cancer.

Colon tumors characterized by a high degree of CIMP are both biologically and clinically distinct (16, 75). Importantly, CIMPness has been correlated with poor patient outcomes and resistance to chemotherapy (76). We uncover a significant correlation between JQ1 sensitivity and CIMP positivity in colon cancer cells. Furthermore, we show that the effects of JQ1 are linked to its ability to inhibit *cMYC* expression specifically in CIMP$^+$ cells. Biologically, CIMP$^+$ tumors have low levels of CIN, and hence disomy, at the *cMYC* locus (77). The preferential effect of JQ1 on *cMYC* in this context suggests that cancers with *cMYC* disomy may be more dependent on superenhancers and other epigenetic mechanisms

Downloaded from http://www.jci.org on February 26, 2017. https://doi.org/10.1172/JCI83265



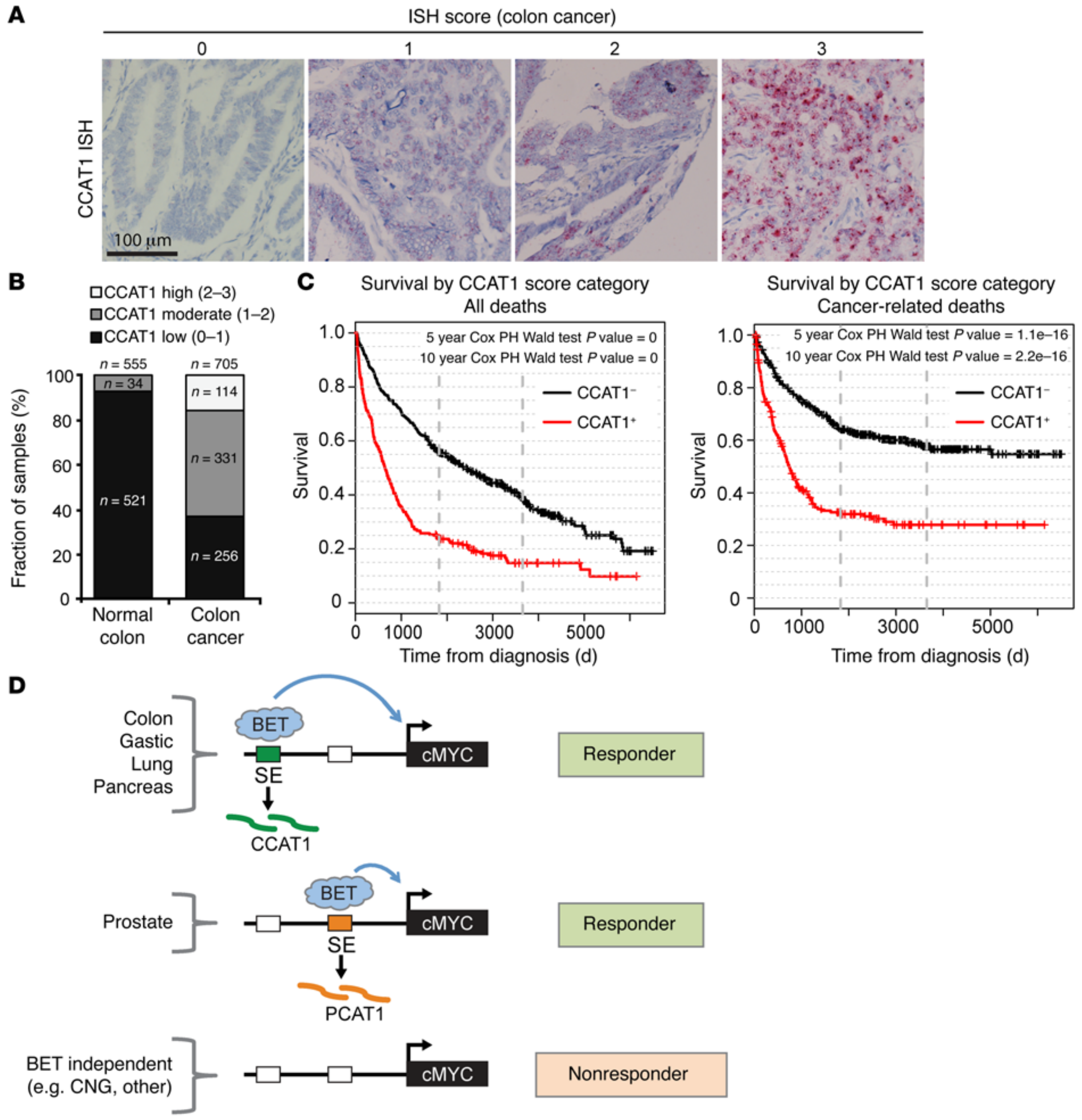


**Figure 7. Patients with colon tumors expressing high CCAT1 levels have a poor clinical outcome.** (**A**) Representative ISH photomicrographs show CCAT1 expression in colon tumors. The ISH scoring system is indicated. Scale bar: 100 μm. (**B**) ISH score breakdown for a panel of normal colon and colon tumors. Each patient sample was scored from triplicate representative tumor cores, and the average CCAT1 ISH score was recorded as low (score of 0–1), moderate (score of 1–2), or high (score of 2–3). (**C**) Kaplan-Meier survival data for patients with colon cancer, separated by CCAT1 ISH score (CCAT1⁻ patients are defined by an ISH score <1, and CCAT1⁺ patients are defined by an ISH score >1). Separate curves are shown for overall survival and colon cancer–specific survival. PH, proportional hazards. (**D**) Model highlighting the utility of lncRNAs in identifying lineage-dependent superenhancers in different cancer types. The model predicts that cMYC-dependent tumors driven by BET and associated superenhancers will be more responsive to BET inhibitors. CNG, copy number gain.

to drive *cMYC* expression. Thus, it would be worth exploring the amplification status of *cMYC* or other relevant oncogenes as correlates of BET activity in clinical trials.

The advent of molecularly targeted cancer therapy has shed light on the critical role that biomarkers play in identifying responsive patients and on the fine-tuning drug-repurposing efforts. Growing evidence in preclinical models shows that BRD4 activity closely associates with the presence of discrete superenhancer elements that direct RNA polymerase II–mediated transcription. Indeed, we found that the p-TEFb interaction domain in BRD4-LF is necessary for BRD4 activity in colon cancer. Unfortunately, superenhancers are variably formed and require sophisticated methodology such as ChIP-seq to detect them. Thus, these elements can be challenging to consistently detect in preserved human tissue and are not amenable as biomarkers in the companion diagnostic setting. Our findings that distinct enhancer template RNAs such as CCAT1 and PCAT1 can be used to uncover superenhancer activity constitute

Downloaded from http://www.jci.org on February 26, 2017. https://doi.org/10.1172/JCI83265



**Table 1. Frequency of CCAT1 expression in colon cancer according to clinical, pathological or molecular feature**

| Feature | Total (*n*) | CCAT1+ | Univariate OR (95% CI) | *P* value |
|---|---|---|---|---|
| All cases | 638 | 202 (32%) | | |
| **Sex** | | | | |
| Male | 326 | 113 (35%) | 1 | |
| Female | 312 | 89 (29%) | 0.75 (0.54–1.04) | |
| **Age at diagnosis (yr)** | | | | |
| <72 | 298 | 94 (32%) | 1 | |
| ≥72 | 340 | 107 (32%) | 1.01 (0.72–1.41) | |
| **Tumor location** | | | | |
| Proximal | 187 | 57 (30%) | 1 | |
| Distal | 305 | 97 (32%) | 1.06 (0.72–1.58) | |
| Rectal | 80 | 27 (34%) | 1.16 (0.66–2.03) | |
| **Tumor stage** | | | | |
| I | 50 | 14 (28%) | 1 | Referent |
| II | 231 | 59 (26%) | 0.88 (0.44–1.75) | |
| III | 198 | 65 (33%) | 1.26 (0.63–2.49) | |
| IV | 84 | 42 (50%) | 2.57 (1.21–5.45) | 0.0182 |
| **Tumor grade** | | | | |
| WD | 94 | 17 (18%) | 1 | Referent |
| MD | 403 | 130 (32%) | 2.16 (1.22–3.80) | 0.0059 |
| PD | 120 | 46 (38%) | 2.82 (1.48–5.35) | 0.0014 |
| **Mucinous component** | | | | |
| Absent | 600 | 196 (33%) | 1 | Referent |
| Present | 38 | 6 (16%) | 0.39 (0.16–0.94) | 0.0309 |
| **cMYC relative copy number** | | | | |
| Low | 66 | 23 (35%) | 1 | Referent |
| Gain | 148 | 47 (32%) | 0.87 (0.47–1.61) | |
| Amplification | 34 | 5 (15%) | 0.32 (0.11–0.95) | 0.037 |
| **cMYC expression** | | | | |
| Low | 103 | 22 (21%) | 1 | Referent |
| Moderate | 297 | 108 (36%) | 2.10 (1.24–3.56) | 0.005 |
| High | 144 | 50 (35%) | 1.96 (1.09–3.51) | 0.0239 |

Only *P* values of less than 0.05 are reported. OR, odds ratio.

an important step forward in the development of biomarkers of BET activity in patients (Figure 7D). Importantly, we show that CCAT1 expression can predict BET inhibitor response in colon, pancreatic, and lung cancers. Our work shows that CCAT1 can be readily detected by either qPCR or an ISH assay, making it an ideal biomarker in formalin-fixed, paraffin-embedded (FFPE) tissues. The CCAT1 eRNA itself has been previously shown to regulate cMYC expression, albeit at lower levels than those seen with BET inhibitors (63, 78). Cumulatively, these data suggest that both BET activity and eRNA expression are necessary to drive *cMYC* transcription in cancers expressing CCAT1. Further work will be necessary to systematically characterize eRNAs and dissect whether they are biomarkers of BET activity or have functional consequences in *cis* or *trans* that impinge on cancer growth and development.

In conclusion, we used an array-based CRISPR screen to identify the BRD4 oncogene as a critical driver of proliferation and the dedifferentiated state in colon cancer. Importantly, we found that CCAT1, a superenhancer RNA, predicts growth sensitivity to BET inhibitors and marks cancer cells using BRD4 to drive *cMYC* expression. Further work will be critical to the translation of these promising findings into a biomarker-driven approach for patient selection in BET inhibitor clinical trials.

## Methods

*Cell culture and generation of stable cell lines expressing Cas9*. All colon, lung, pancreatic, gastric, and blood cell lines were cultured in RPMI-1640 supplemented with 10% FBS, 2 mmol/l L-glutamine (Invitrogen), and 1% penicillin-streptomycin (Invitrogen). 293T cells were grown in DMEM (high-glucose), 10% FBS, 100 μmol/l nonessential amino acids (Invitrogen), 2 mmol/l L-glutamine (Invitrogen), and 1% penicillin-streptomycin. All cell lines were obtained from Genentech's internal cell line repository.

Cas9 was cloned into pLenti7.3 (Invitrogen), and RKO cells were infected at an MOI of greater than 1. Stably expressing Cas9 cells were collected by FACS using the GFP-selectable marker.

*Immunoblot analysis, immunofluorescence, and Abs*. The following Abs were used for immunoblot and immunofluorescence expression

Downloaded from http://www.jci.org on February 26, 2017. https://doi.org/10.1172/JCI83265



analyses: HDAC1 (Cell Signaling Technology; 5356S); CHD1 (Cell Signaling Technology; 4351S); PYGO2 (Epitomics; 3273-1); BRD4 (Epitomics: 5716-1; Bethyl Laboratories: A301-985A100; Cell Signaling Technology: 12183); KAT8 (Bethyl Laboratories; A300-992A); aurora B (BD Biosciences; 611082); KDM3B (Sigma-Aldrich; HPA016610); β-catenin (Cell Signaling Technology; 8480S); NSD2 (Bethyl Laboratories; A303-093A); EZH2 (Cell Signaling Technology; 5246); cMYC (Epitomics; 1472-1); actin (MP Biomedicals; 691002); and tubulin (Sigma-Aldrich; T6074). Immunofluorescence was performed as previously described (79).

*Xenograft studies*. Xenograft tumor studies were performed essentially as described previously (80). Briefly, HT-29 and HCT 116 cells were infected with doxycycline-inducible pHUSH shRNAs targeting BRD4 or nontargeting control (NTC). Target sequences for shBRD4-1 (AGGAAGAGGACAAGTGCAA) and shBRD4-2 (AGAAGGGAGTGAAGAGGAA) were used. For each cell line, $5 \times 10^6$ cells were injected s.c. into the right flank of female NCR nude mice (Taconic). Once tumors reached 200 $mm^3$ in size, hairpin expression was induced with 0.5 mg/ml doxycycline (or sucrose as a control). shRNA efficiency was examined after 7 days of doxycycline treatment in a subset of mice. Tumor volume and body weight were measured in the remaining mice every 3 to 4 days until the end of the study.

*Calculation of relative $EC_{50}$ values*. Cell lines were placed in 96-well plates 12 hours before the addition of drug. Compounds were diluted in DMSO, with final target concentrations ranging from 20 μM to 3 nM (using 3-fold serial dilutions). Cell numbers were quantified 3 days after drug addition with CellTiter-Glo (CTG) (Promega). $EC_{50}$ values were calculated using GraphPad Prism, version 5 (GraphPad Software).

*Transcriptomic and ChIP-seq data sets*. RNA-seq data were deposited in the NCBI's Gene Expression Omnibus (GEO) database (GEO GSE73317 for RNAi experiments and GEO GSE73318 for JQ1 treatments). Also, ChIP-seq data were deposited in the GEO database (GEO GSE73319).

*Statistics*. All IncuCyte (Essen Bioscience) experiments are reported as the mean ± SEM. Results were analyzed using a 2-tailed Students $t$ test when comparisons were made. The Pearson's product-moment correlation coefficient ($r$) was used to measure linear correlations between 2 variables. Statistical significance was defined as $P < 0.05$.

*Study approval*. Approval for use of the Leeds tumor collection was provided by an internal Genentech review and ethics committee. Animal experiments were reviewed and approved by the IACUC of Genentech Inc.

Additional details are provided in the Supplemental Methods.

## Author contributions

MLM, KM, VSC, and RF performed and designed the experiments. FG and OM performed biostatistical analyses. CW, BH, VSC, and RF designed and constructed the gRNA library. MY developed and performed the CCAT1 ISH assay. EL and ES designed and performed the xenograft experiments. MLM, KM, FG, and RF wrote the manuscript.

## Acknowledgments

The authors thank Allison Bruce for the illustrations; Trinna Cuellar, J.P. Stephan, Manuel Viotti, and Katrina Nichols for their assistance with the screen; Christiaan Klijn and Pete Haverty for their bioinformatics work; and Jeff Settleman, William Forrest, Bob Yauch, and Karen Gascoigne for their helpful discussions.

Address correspondence to: Mark McCleland, Genentech, Inc., 1 DNA Way, South San Francisco, California 94080, USA. Phone: 650.225.8211; E-mail: marklm@gene.com. Or to: Ron Firestein, Hudson Institute, 27-31 Wright Street, Clayton, Victoria, Australia. Phone: 61.3.8572.2773; E-mail: ron.firestein@hudson.org.au.

Ron Firestein's present address is: Hudson Institute, Clayton, Victoria, Australia.

1. Haggar FA, Boushey RP. Colorectal cancer epidemiology: incidence, mortality, survival, and risk factors. *Clin Colon Rectal Surg*. 2009;22(4):191–197.
2. Fearon ER. Molecular genetics of colorectal cancer. *Ann N Y Acad Sci*. 1995;768:101–110.
3. Fearon ER, Vogelstein B. A genetic model for colorectal tumorigenesis. *Cell*. 1990;61(5):759–767.
4. Sillars-Hardebol AH, et al. Identification of key genes for carcinogenic pathways associated with colorectal adenoma-to-carcinoma progression. *Tumour Biol*. 2010;31(2):89–96.
5. Issa JP. Colon cancer: it's CIN or CIMP. *Clin Cancer Res*. 2008;14(19):5939–5940.
6. Ogino S, Goel A. Molecular classification and correlates in colorectal cancer. *J Mol Diagn*. 2008;10(1):13–27.
7. Toyota M, Ahuja N, Ohe-Toyota M, Herman JG, Baylin SB, Issa JP. CpG island methylator phenotype in colorectal cancer. *Proc Natl Acad Sci U S A*. 1999;96(15):8681–8686.
8. Adler AS, et al. An integrative analysis of colon cancer identifies an essential function for PRPF6 in tumor growth. *Genes Dev*. 2014;28(10):1068–1084.
9. Salari K, et al. CDX2 is an amplified lineage-survival oncogene in colorectal cancer. *Proc Natl Acad Sci U S A*. 2012;109(46):E3196–E3205.
10. Firestein R, Hahn WC. Revving the Throttle on an oncogene: CDK8 takes the driver seat. *Cancer Res*. 2009;69(20):7899–7901.
11. Dulak AM, et al. Gastrointestinal adenocarcinomas of the esophagus, stomach, and colon exhibit distinct patterns of genome instability and oncogenesis. *Cancer Res*. 2012;72(17):4383–4393.
12. Fearon ER, et al. Identification of a chromosome 18q gene that is altered in colorectal cancers. *Science*. 1990;247(4938):49–56.
13. Powell SM, et al. APC mutations occur early during colorectal tumorigenesis. *Nature*. 1992;359(6392):235–237.
14. Vogelstein B, Papadopoulos N, Velculescu VE, Zhou S, Diaz LA Jr, Kinzler KW. Cancer genome landscapes. *Science*. 2013;339(6127):1546–1558.
15. Garraway LA, Lander ES. Lessons from the cancer genome. *Cell*. 2013;153(1):17–37.
16. Lao VV, Grady WM. Epigenetics and colorectal cancer. *Nat Rev Gastroenterol Hepatol*. 2011;8(12):686–700.
17. Hammoud SS, Cairns BR, Jones DA. Epigenetic regulation of colon cancer and intestinal stem cells. *Curr Opin Cell Biol*. 2013;25(2):177–183.
18. Teo JL, Kahn M. The Wnt signaling pathway in cellular proliferation and differentiation: A tale of two coactivators. *Adv Drug Deliv Rev*. 2010;62(12):1149–1155.
19. Blythe SA, Cha SW, Tadjuidje E, Heasman J, Klein PS. beta-Catenin primes organizer gene expression by recruiting a histone H3 arginine 8 methyltransferase, Prmt2. *Dev Cell*. 2010;19(2):220–231.
20. Ying Y, Tao Q. Epigenetic disruption of the WNT/beta-catenin signaling pathway in human cancers. *Epigenetics*. 2009;4(5):307–312.
21. Jiang X, et al. DACT3 is an epigenetic regulator of Wnt/β-catenin signaling in colorectal cancer and is a therapeutic target of histone modifications. *Cancer Cell*. 2008;13(6):529–541.
22. Suzuki H, et al. Epigenetic inactivation of SFRP genes allows constitutive WNT signaling in colorectal cancer. *Nat Genet*. 2004;36(4):417–422.
23. Baylin SB. DNA methylation and gene silencing in cancer. *Nat Clin Pract Oncol*. 2005; 2(suppl 1):S4–S11.
24. Herman JG, Baylin SB. Gene silencing in cancer in association with promoter hypermethylation. *N Engl J Med*. 2003;349(21):2042–2054.

Downloaded from http://www.jci.org on February 26, 2017. https://doi.org/10.1172/JCI83265




25. Issa JP. The epigenetics of colorectal cancer. *Ann N Y Acad Sci*. 2000;910:140–153.
26. Suzuki H, et al. A genomic screen for genes upregulated by demethylation and histone deacetylase inhibition in human colorectal cancer. *Nat Genet*. 2002;31(2):141–149.
27. Baylin SB. Mechanisms underlying epigenetically mediated gene silencing in cancer. *Semin Cancer Biol*. 2002;12(5):331–337.
28. Wee S, et al. Targeting epigenetic regulators for cancer therapy. *Ann N Y Acad Sci*. 2014;1309:30–36.
29. Ahuja N, Easwaran H, Baylin SB. Harnessing the potential of epigenetic therapy to target solid tumors. *J Clin Invest*. 2014;124(1):56–63.
30. Azad N, Zahnow CA, Rudin CM, Baylin SB. The future of epigenetic therapy in solid tumours — lessons from the past. *Nat Rev Clin Oncol*. 2013;10(5):256–266.
31. Di Costanzo A, Del Gaudio N, Migliaccio A, Altucci L. Epigenetic drugs against cancer: an evolving landscape. *Arch Toxicol*. 2014;88(9):1651–1668.
32. Filippakopoulos P, et al. Selective inhibition of BET bromodomains. *Nature*. 2010;468(7327):1067–1073.
33. Filippakopoulos P, Knapp S. Targeting bromodomains: epigenetic readers of lysine acetylation. *Nat Rev Drug Discov*. 2014;13(5):337–356.
34. Cong L, et al. Multiplex genome engineering using CRISPR/Cas systems. *Science*. 2013;339(6121):819–823.
35. Jinek M, East A, Cheng A, Lin S, Ma E, Doudna J. RNA-programmed genome editing in human cells. *Elife*. 2013;2:e00471.
36. Mali P, et al. RNA-guided human genome engineering via Cas9. *Science*. 2013;339(6121):823–826.
37. Vermeulen K, Van Bockstaele DR, Berneman ZN. The cell cycle: a review of regulation, deregulation and therapeutic targets in cancer. *Cell Prolif*. 2003;36(3):131–149.
38. Wang T, Wei JJ, Sabatini DM, Lander ES. Genetic screens in human cells using the CRISPR-Cas9 system. *Science*. 2014;343(6166):80–84.
39. Shalem O, et al. Genome-scale CRISPR-Cas9 knockout screening in human cells. *Science*. 2014;343(6166):84–87.
40. Koike-Yusa H, Li Y, Tan EP, Velasco-Herrera Mdel C, Yusa K. Genome-wide recessive genetic screening in mammalian cells with a lentiviral CRISPR-guide RNA library. *Nat Biotechnol*. 2014;32(3):267–273.
41. Shi J, Vakoc CR. The mechanisms behind the therapeutic activity of BET bromodomain inhibition. *Mol Cell*. 2014;54(5):728–736.
42. Rodriguez RM, et al. Aberrant epigenetic regulation of bromodomain BRD4 in human colon cancer. *J Mol Med (Berl)*. 2012;90(5):587–595.
43. Hu Y, et al. BRD4 inhibitor inhibits colorectal cancer growth and metastasis. *Int J Mol Sci*. 2015;16(1):1928–1948.
44. Floyd SR, et al. The bromodomain protein Brd4 insulates chromatin from DNA damage signalling. *Nature*. 2013;498(7453):246–250.
45. Wang R, Li Q, Helfer CM, Jiao J, You J. Bromodomain protein Brd4 associated with acetylated chromatin is important for maintenance of higher-order chromatin structure. *J Biol Chem*. 2012;287(14):10738–10752.
46. Yang Z, et al. Recruitment of P-TEFb for stimulation of transcriptional elongation by the bromodomain protein Brd4. *Mol Cell*. 2005;19(4):535–545.
47. Jang MK, Mochizuki K, Zhou M, Jeong HS, Brady JN, Ozato K. The bromodomain protein Brd4 is a positive regulatory component of P-TEFb and stimulates RNA polymerase II-dependent transcription. *Mol Cell*. 2005;19(4):523–534.
48. Mochizuki K, et al. The bromodomain protein Brd4 stimulates G1 gene transcription and promotes progression to S phase. *J Biol Chem*. 2008;283(14):9040–9048.
49. Alsarraj J, et al. Deletion of the proline-rich region of the murine metastasis susceptibility gene Brd4 promotes epithelial-to-mesenchymal transition- and stem cell-like conversion. *Cancer Res*. 2011;71(8):3121–3131.
50. Zuber J, et al. RNAi screen identifies Brd4 as a therapeutic target in acute myeloid leukaemia. *Nature*. 2011;478(7370):524–528.
51. Gray DC, et al. pHUSH: a single vector system for conditional gene expression. *BMC Biotechnol*. 2007;7:61.
52. Ribbing J, Nyberg J, Caster O, Jonsson EN. The lasso — a novel method for predictive covariate model building in nonlinear mixed effects models. *J Pharmacokinet Pharmacodyn*. 2007;34(4):485–517.
53. Ogino S, Kawasaki T, Kirkner GJ, Kraft P, Loda M, Fuchs CS. Evaluation of markers for CpG island methylator phenotype (CIMP) in colorectal cancer by a large population-based sample. *J Mol Diagn*. 2007;9(3):305–314.
54. Lleras RA, et al. Hypermethylation of a cluster of Kruppel-type zinc finger protein genes on chromosome 19q13 in oropharyngeal squamous cell carcinoma. *Am J Pathol*. 2011;178(5):1965–1974.
55. Mirguet O, et al. Discovery of epigenetic regulator I-BET762: lead optimization to afford a clinical candidate inhibitor of the BET bromodomains. *J Med Chem*. 2013;56(19):7501–7515.
56. Fearon ER, Dang CV. Cancer genetics: tumor suppressor meets oncogene. *Curr Biol*. 1999;9(2):R62–R65.
57. Sansom OJ, et al. Myc deletion rescues Apc deficiency in the small intestine. *Nature*. 2007;446(7136):676–679.
58. Whyte WA, et al. Master transcription factors and mediator establish super-enhancers at key cell identity genes. *Cell*. 2013;153(2):307–319.
59. Dowen JM, et al. Control of cell identity genes occurs in insulated neighborhoods in mammalian chromosomes. *Cell*. 2014;159(2):374–387.
60. Mansour MR, et al. Oncogene regulation. *Science*. 2014;346(6215):1373–1377.
61. Ma MZ, et al. Long non-coding RNA CCAT1 promotes gallbladder cancer development via negative modulation of miRNA-218-5p. *Cell Death Dis*. 2015;6:e1583.
62. Mizrahi I, et al. Colon Cancer Associated Transcript-1 (CCAT1) expression in adenocarcinoma of the stomach. *J Cancer*. 2015;6(2):105–110.
63. Xiang JF, et al. Human colorectal cancer-specific CCAT1-L lncRNA regulates long-range chromatin interactions at the MYC locus. *Cell Res*. 2014;24(5):513–531.
64. Asangani IA, et al. Therapeutic targeting of BET bromodomain proteins in castration-resistant prostate cancer. *Nature*. 2014;510(7504):278–282.
65. Kim TK, et al. Widespread transcription at neuronal activity-regulated enhancers. *Nature*. 2010;465(7295):182–187.
66. Wang D, et al. Reprogramming transcription by distinct classes of enhancers functionally defined by eRNA. *Nature*. 2011;474(7351):390–394.
67. Kanno T, et al. BRD4 assists elongation of both coding and enhancer RNAs by interacting with acetylated histones. *Nat Struct Mol Biol*. 2014;21(12):1047–1057.
68. Klijn C, et al. A comprehensive transcriptional portrait of human cancer cell lines. *Nat Biotechnol*. 2015;33(3):306–312.
69. Lockwood WW, Zejnullahu K, Bradner JE, Varmus H. Sensitivity of human lung adenocarcinoma cell lines to targeted inhibition of BET epigenetic signaling proteins. *Proc Natl Acad Sci U S A*. 2012;109(47):19408–19413.
70. Watson IR, Takahashi K, Futreal PA, Chin L. Emerging patterns of somatic mutations in cancer. *Nat Rev Genet*. 2013;14(10):703–718.
71. Dawson MA, Kouzarides T. Cancer epigenetics: from mechanism to therapy. *Cell*. 2012;150(1):12–27.
72. Dawson MA, Kouzarides T, Huntly BJ. Targeting epigenetic readers in cancer. *N Engl J Med*. 2012;367(7):647–657.
73. Boettcher M, Hoheisel JD. Pooled RNAi screens — technical and biological aspects. *Curr Genomics*. 2010;11(3):162–167.
74. Bertrand FE, Angus CW, Partis WJ, Sigounas G. Developmental pathways in colon cancer: crosstalk between WNT, BMP, Hedgehog and Notch. *Cell Cycle*. 2012;11(23):4344–4351.
75. Suzuki H, Yamamoto E, Maruyama R, Niinuma T, Kai M. Biological significance of the CpG island methylator phenotype. *Biochem Biophys Res Commun*. 2014;455(1–2):35–42.
76. Phipps AI, et al. Association between molecular subtypes of colorectal cancer and patient survival. *Gastroenterology*. 2015;148(1):77–87 e72.
77. Cheng YW, et al. CpG island methylator phenotype associates with low-degree chromosomal abnormalities in colorectal cancer. *Clin Cancer Res*. 2008;14(19):6005–6013.
78. Younger ST, Rinn JL. ‘Lnc’-ing enhancers to MYC regulation. *Cell Res*. 2014;24(6):643–644.
79. McCleland ML, et al. Cdk8 deletion in the Apc murine tumour model represses EZH2 activity and accelerates tumourigenesis [published online ahead of print August 3, 2015 *J Pathol*. doi:10.1002/path.4596.
80. Adler AS, et al. CDK8 maintains tumor dedifferentiation and embryonic stem cell pluripotency. *Cancer Res*. 2012;72(8):2129–2139.
