# Supplementary material for: Expression of Concern: Prognostic value of long non-coding RNA CCAT1 expression in patients with cancer: A meta-analysis
Source: PLoS One. 2023 Apr 20;18(4):e0284940. doi: 10.1371/journal.pone.0284940 (PMC10118116; doi:10.1371/journal.pone.0284940)
Supplement: S1 File — (ZIP) [file pone.0284940.s001.zip › 4 Documents/06 Quality assessment of primary Studies under Method.docx]

Quality assessment of primary Studies under Methods should be improved and criteria used should be clearly discussed.

For quality control of the paper, the assessment was performed by three authors (DY Shi, FS Wu, F Gao) according to the Newcastle-Ottawa Scale (NOS) oriented to cohort studies. NOS contains 3 categories including selection (4 items), comparability (2 item) and outcome (3 items). If a study met the following criteria, it can be awarded 1 score for each numbered item. Criteria were as below: In selection category, Item 1: exposed cohort truly represent the cancer patients with high expression level of CCAT1 in the community or somewhat represent those cancer patients in the community. Item 2: the non-exposed cohort, namely the cancer patients with low expression level of CCAT1, were drawn from the same community as the exposed cohort. Item 3: relative expression level of CCAT1 of cancer patients was precise and exact. Item 4: demonstration that outcome of interest was not present at start of study. In Comparability category, Item 1: Study controls for the most important factor: all patients had negative histories of exposure to either chemotherapy or radiotherapy before surgery. Item 2: study controls for any additional factor (This criterion could be modified to indicate specific control for a second important factor). In outcome category, Item 1: Assessment of outcome was independent blind assessment or reliable record. Item 2: Follow-up was long enough for outcomes to occur (>= 30 months). Item 3: Adequacy of follow up of cohorts, complete follow up or subjects lost to follow up unlikely to introduce bias, loss of follow-up was smaller than 20% or description provided of those lost. Disagreements were resolved through discussion with another researcher (XC Qing). The total scores ranges from 0 to 9. The study is considered high quality, if its scores is greater than or equal to 7.

| article | selection | | | | comparability | | outcome | | | NOS scores |
| --- | --- | --- | --- | --- | --- | --- | --- | --- | --- | --- |
|  | Item 1 | Item 2 | Item 3 | Item 4 | Item 1 | Item 2 | Item 1 | Item 2 | Item 3 |  |
| Deng et al. | 0 | 1 | 1 | 1 | 0 | 0 | 1 | 1 | 1 | 6 |
| Zhu et al. | 0 | 1 | 1 | 1 | 1 | 0 | 1 | 1 | 0 | 6 |
| Zhang et al. | 1 | 1 | 1 | 1 | 0 | 0 | 1 | 1 | 1 | 7 |
| He et al. | 1 | 1 | 1 | 1 | 1 | 0 | 1 | 1 | 0 | 7 |
| Zhao et al. | 1 | 1 | 1 | 1 | 1 | 0 | 1 | 1 | 0 | 7 |
| Cui et al. | 1 | 1 | 1 | 1 | 1 | 0 | 1 | 1 | 0 | 7 |
| Zhang et al. | 0 | 1 | 1 | 1 | 1 | 0 | 1 | 1 | 0 | 6 |
| Luo etal. | 0 | 1 | 1 | 1 | 1 | 0 | 1 | 1 | 0 | 6 |
| Wang et al. | 1 | 1 | 1 | 1 | 1 | 0 | 1 | 1 | 0 | 7 |
| McCleland et al. | 1 | 1 | 1 | 1 | 0 | 0 | 1 | 1 | 0 | 6 |
| Liu et al. | 1 | 1 | 1 | 1 | 1 | 0 | 1 | 1 | 0 | 7 |
