# Supplementary material for: Expression of Concern: Prognostic value of long non-coding RNA CCAT1 expression in patients with cancer: A meta-analysis
Source: PLoS One. 2023 Apr 20;18(4):e0284940. doi: 10.1371/journal.pone.0284940 (PMC10118116; doi:10.1371/journal.pone.0284940)
Supplement: S1 File — (ZIP) [file pone.0284940.s001.zip › 4 Documents/Tables.docx]

Records identified through electronic databases Pubmed, Web of Science, OVID and CNKI (n=271; 182 in English,89 in Chinese)

Full-text retrieved for eligibility (n=22; 18 in English, 4 in Chinese)

Studies included in Meta-analysis (n=11; 10 in English, 1 in Chinese)

Excluded by title and abstract information;

(n=169; 84 in English, 85 in Chinese)

Excluded by details;

1)Article did not investigate survival

2)Study without usable data

(n=11; 8 in English, 3 in Chinese)

Articles retrieved from other sources (n=15, in English)

Records after duplicates removed (n=191; 102 in English, 89 in Chinese)

**Figure 1. The flow diagram of the meta analysis**

**Table 1.** Characteristics of studies included in the meta-analysis.

|  |  |  |  |  |  |  |  |  |  |  |  |  |  |  |
| --- | --- | --- | --- | --- | --- | --- | --- | --- | --- | --- | --- | --- | --- | --- |
| Author | year | region | SampleType | tumor type | sample size | preoperative treatment | clinical stage of tumor | method of CCAT1 expression | Elevated CCAT1 | cut-off value | outcome measure | survival analysis | Method^*^ | NOS scores |
| Deng et al. | 2015 | China | Tissue | Hepatocellular carcinoma | 66 | N/A | N/A | qRT-PCR | significant higher(p<0.001) | median | RFS,OS | No | 3 | 7 |
| Zhu et al. | 2015 | China | Tissue | Hepatocellular Carcinoma | 86 | No | N/A | qRT-PCR | N/A | median | RFS,OS | Univariable, multivariable analysis | 1,2 | 7 |
| Zhang et al. | 2016 | China | Tissue | Esophageal squamous cell carcinoma | 90 | N/A | I-IV | qRT-PCR | 80.7%，significant higher(p<0.001) | median | OS | Univariable, multivariable analysis | 1 | 7 |
| He et al. | 2014 | China | Tissue | Colon cancer | 48 | No | I-IV | qRT-PCR | significant higher(p<0.05) | median | OS | No | 3 | 6 |
| Zhao et al. | 2016 | China | Tissue | Endometrial carcinoma | 108 | No | FIGO I-IV | qRT-PCR | 4.77folds，significant higher(p<0.001) | median | OS | No | 3 | 6 |
| Cui et al. | 2015 | China | Plasma | Colorectal cancer | 60 | No | I-IV | qRT-PCR | significant higher(p<0.001) | RQV | OS | No | 3 | 6 |
| Zhang et al. | 2015 | China | Tissue | Breast cancer | 92 | No | I-III | qRT-PCR | significant higher(p<0.05) | median | OS,PFS | Multivariate | 1 | 6 |
| Luo et al. | 2014 | China | Tissue | Non-small cell lung cancer | 62 | No | II-IV | qRT-PCR | significant higher(p<0.0001) | median | OS | No | 3 | 5 |
| Wang et al. | 2015 | China | Tissue | Hepatocellular carcinoma | 97 | No | BCLC 0-C | qRT-PCR | significant higher，more than 2 folds(p<0.05) | 2-fold compared with ANT | OS,DFS,RFS | Univariable, multivariable analysis | 1 | 7 |
| McCleland et al. | 2015 | UK | Tissue | Colorectal cancer | 638 | N/A | I-IV | ISH assay | significant higher(p<0.05) | ISH score = 1 | OS | Multivariate | 1 | 6 |
| Liu et al. | 2017 | China | Tissue | Gastric cancer | 240 | No | I-IV | qRT-PCR | significant higher(p<0.001) | 0.041 times of 2^(-∆∆Ct) | RFS,OS | Univariable, multivariable analysis | 1 | 7 |

OS: overall survival. DFS: disease free survival. PFS: progression free survival. RFS: recurrence free survival.
∆Ct=Ct (CCAT1) - Ct (GAPDH). ANT: adjacent non-tumor tissues. NOS: Newcastle-Ottawa Scale.
ISH: In Situ Hybridization. NA: not available. RQV: risk quotient value
*1 denoted as obtaining HRs directly from publications; 2 denoted as HRs were calculated from the total number of events and its p value; 3 denoted as extracting HRs from Kaplan-Meier curves.

qRT-PCR: quantitative reverse transcription PCR.

**Table 2.** Results of subgroup analysis of pooled hazard ratios of overall survival of different types of cancer with increased CCAT1 expression.

|  |  |  |  | |  |  | |
| --- | --- | --- | --- | --- | --- | --- | --- |
| **Subgroup analysis** | **No. of studies** | **No. of patients** | **Pooled HR(95%CI)** | | **Meta regression (*p* -value)** | **Heterogeneity** | |
|  |  |  | **Fixed** | **Random** |  | **I^2^** | ***p* -value** |
| **Region** |  |  |  |  |  |  |  |
| China | 10 | 949 | 1.057[1.038-1.076] | 2.401[1.483-3.887] | 0.832 | 80.4% | 0.000 |
| UK | 1 | 638 | 2.080[1.564-2.767] | 2.080[1.564-2.767] |  | - | - |
| **Sample size** |  |  |  |  |  |  |  |
| <100 | 8 | 986 | 1.057[1.038-1.076] | 2.393[1.394-4.106] | 0.943 | 83.3% | 0.000 |
| ≥100 | 3 | 601 | 2.113[1.615-2.765] | 2.113[1.615-2.765] |  | 0.0% | 0.880 |
| **Type of cancer** |  |  |  |  |  |  |  |
| Digestive system carcinoma | 8 | 1325 | 1.059[1.040-1.078] | 2.212[1.394-3.510] | 0.525 | 87.2% | 0.000 |
| Non-digestive system carcinoma | 3 | 262 | 2.780[1.645-4.699] | 2.780[1.645-4.699] |  | 0.0% | 0.964 |
| **Preoperative treatment** |  |  |  |  |  |  |  |
| Unclear | 3 | 794 | 1.056[1.037-1.075] | 1.513[0.826-2.772] | 0.025 | 91.1% | 0.000 |
| No | 8 | 793 | 2.889[2.147-3.888] | 2.889[2.147-3.888] |  | 0.0% | 0.993 |
| **NOS score** |  |  |  |  |  |  |  |
| ≥7 | 4 | 579 | 1.055[1.036-1.074] | 2.095[1.061-4.136] | 0.437 | 82.6% | 0.000 |
| <7 | 7 | 1008 | 2.296[1.819-2.897] | 2.296[1.819-2.897] |  | 0.0% | 0.872 |

**Table 3.** Results of subgroup analysis of the independent role of CCAT1 in overall survival/recurrence of different types of cancer.

|  |  |  |  | |  |  | |
| --- | --- | --- | --- | --- | --- | --- | --- |
| **Subgroup analysis** | **No. of studies** | **No. of patients** | **Pooled HR(95%CI)** | | **Meta regression (*p* -value)** | **Heterogeneity** | |
|  |  |  | **Fixed** | **Random** |  | **I^2^** | ***p* -value** |
| **Overall survival** | 6 | 1243 | 1.052 [1.030-1.073] | 2.195[1.316-3.664] |  | 91.3% | 0.000 |
| **Region** |  |  |  |  |  |  |  |
| China | 5 | 605 | 1.048[1.027-1.070] | 2.262[1.165-4.392] | 0.935 | 88.6% | 0.000 |
| UK | 1 | 638 | 2.080[1.564-2.767] | 2.080[1.564-2.767] |  | - | - |
| **Sample size** |  |  |  |  |  |  |  |
| <100 | 4 | 365 | 1.047[1.026-1.069] | 2.240[1.048-4.787] | 0.958 | 90.4% | 0.000 |
| ≥100 | 2 | 878 | 2.113[1.613-2.767] | 2.113[1.613-2.767] |  | 0.0% | 0.742 |
| **Type of cancer** |  |  |  |  |  |  |  |
| Digestive system carcinoma | 5 | 1151 | 1.051[1.029-1.073] | 2.089[1.211-3.606] | 0.611 | 91.9% | 0.000 |
| Non-digestive system carcinoma | 1 | 92 | 2.891[1.412-5.918] | 2.891[1.412-5.918] |  | - | - |
| **Preoperative treatment** |  |  |  |  |  |  |  |
| Unclear | 2 | 728 | 1.048[1.026-1.069] | 1.451[0.739-2.850] | 0.093 | 95.5% | 0.000 |
| No | 4 | 515 | 2.925[2.078-4.116] | 2.925[2.078-4.116] |  | 0.0% | 0.953 |
| **NOS score** |  |  |  |  |  |  |  |
| ≥7 | 4 | 513 | 1.047[1.026-1.069] | 2.137[1.018-4.486] | 0.767 | 89.0% | 0.000 |
| <7 | 2 | 730 | 2.176[1.669-2.836] | 2.176[1.669-2.836] |  | 0.0% | 0.403 |
| **Recurrence** | 4 | 515 | 2.609[1.825-3.728] | 2.609[1.825-3.728] | - | 0.0% | 0.481 |

**Table 4.** Results of meta-analysis of increased CCAT1 expression and clinicopathological features in various cancers.

|  |  |  |  | |  | |
| --- | --- | --- | --- | --- | --- | --- |
| **Cancer types** | **No. of studies** | **No. of patients** | **Pooled OR** | | **Heterogeneity** | |
|  |  |  | **Fixed** | **Random** | **I^2^** | ***p* -value** |
| **TNM stage** |  |  |  |  |  |  |
| Colorectal cancer | 2 | 686 | 1.924[1.365-2.713] | 2.849[0.834-9.733] | 72.1% | 0.058 |
| Esophageal squamous cell carcinoma | 1 | 90 | 2.175[0.869-5.445] | 2.175[0.869-5.445] | - | - |
| Breast cancer | 1 | 92 | 6.908[2.647-18.028] | 6.908[2.647-18.028] | - | - |
| **Tumor Size** |  |  |  |  |  |  |
| Hepatocellular carcinoma | 2 | 163 | 2.664[1.399-5.072] | 2.663[1.396-5.080] | 0.0% | 0.480 |
| Colorectal cancer | 1 | 48 | 5.464[1.627-18.357] | 5.464[1.627-18.357] | - | - |
| Gastric cancer | 1 | 240 | 1.414[0.838-2.387] | 1.414[0.838-2.387] | - | - |
| **Lymph node metastasis** |  |  |  |  |  |  |
| Colorectal cancer | 1 | 48 | 5.000[1.448-17.271] | 5.000[1.448-17.271] | - | - |
| Esophageal squamous cell carcinoma | 1 | 90 | 2.480[1.060-5.803] | 2.480[1.060-5.803] | - | - |
| Endometrial Carcinoma | 1 | 108 | 3.571[1.072-11.901] | 3.571[1.072-11.901] | - | - |
| Breast cancer | 1 | 92 | 5.882[1.569-22.047] | 5.882[1.569-22.047] | - | - |
| Gastric cancer | 1 | 240 | 2.349[1.394-3.956] | 2.349[1.394-3.956] | - | - |
| **Distant metastasis** |  |  |  |  |  |  |
| Gastric cancer | 1 | 240 | 2.345[1.226-4.486] | 2.345[1.226-4.486] | - | - |
| **Microvascular invasion** |  |  |  |  |  |  |
| Hepatocellular carcinoma | 2 | 163 | 4.523[2.157-9.480] | 4.487[2.136-9.426] | 0.0% | 0.674 |
| **Capsular formation** |  |  |  |  |  |  |
| Hepatocellular carcinoma | 2 | 163 | 0.419[0.214-0.818] | 0.417[0.214-0.816] | 0.0% | 0.487 |
