# Supplementary figures and images for: Expression of Concern: Prognostic value of long non-coding RNA CCAT1 expression in patients with cancer: A meta-analysis
Source: PLoS One. 2023 Apr 20;18(4):e0284940. doi: 10.1371/journal.pone.0284940 (PMC10118116; doi:10.1371/journal.pone.0284940)

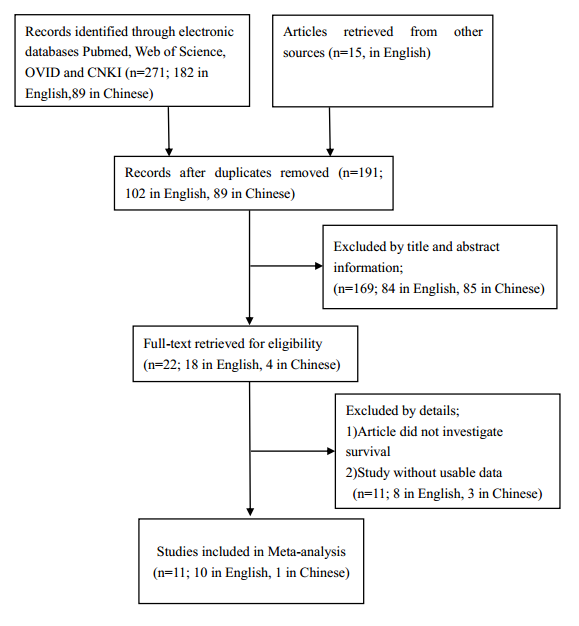

Supplement: S1 File — (ZIP) [file pone.0284940.s001.zip › 2 Figures/Fig 1.tif]

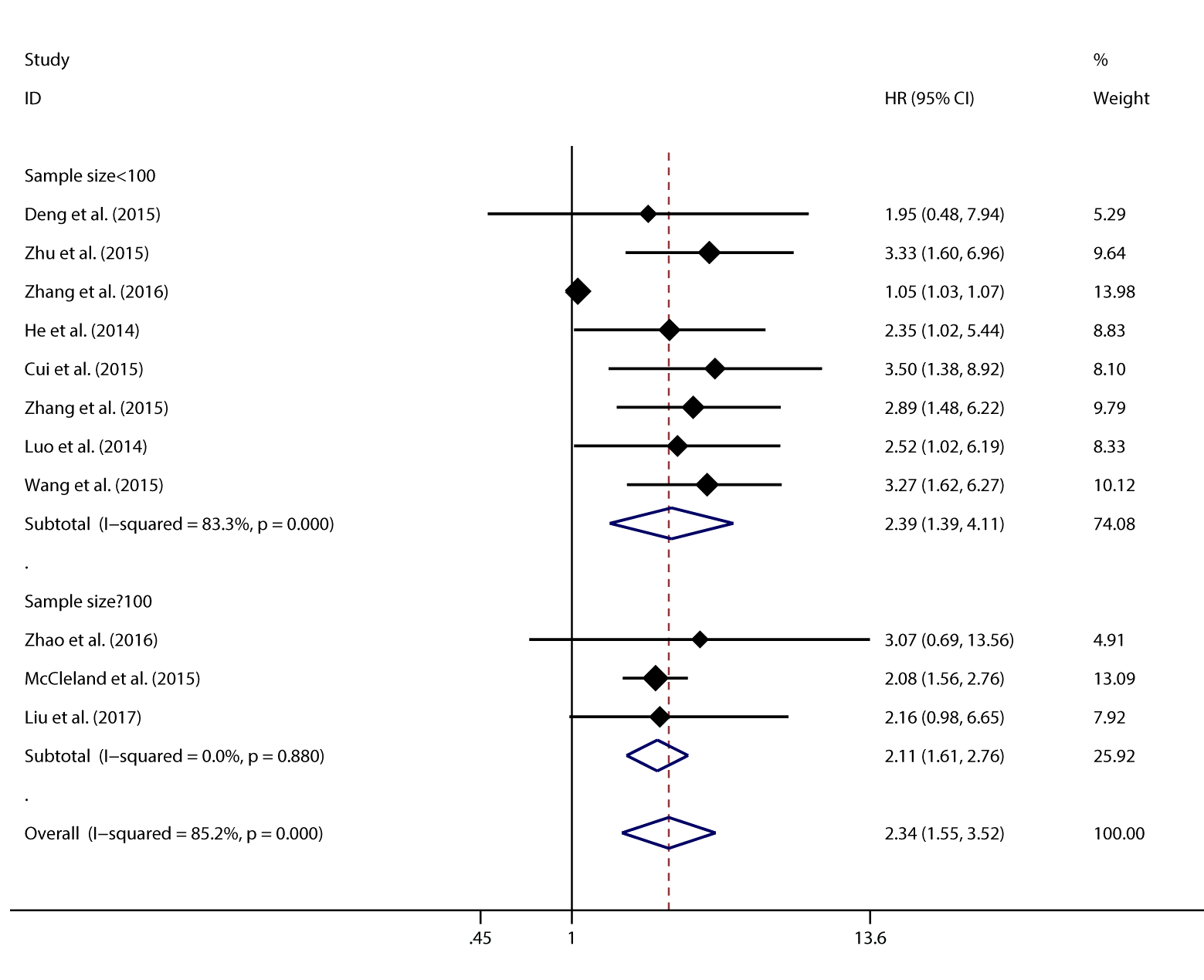

Supplement: S1 File — (ZIP) [file pone.0284940.s001.zip › 2 Figures/Fig 2A.tif]

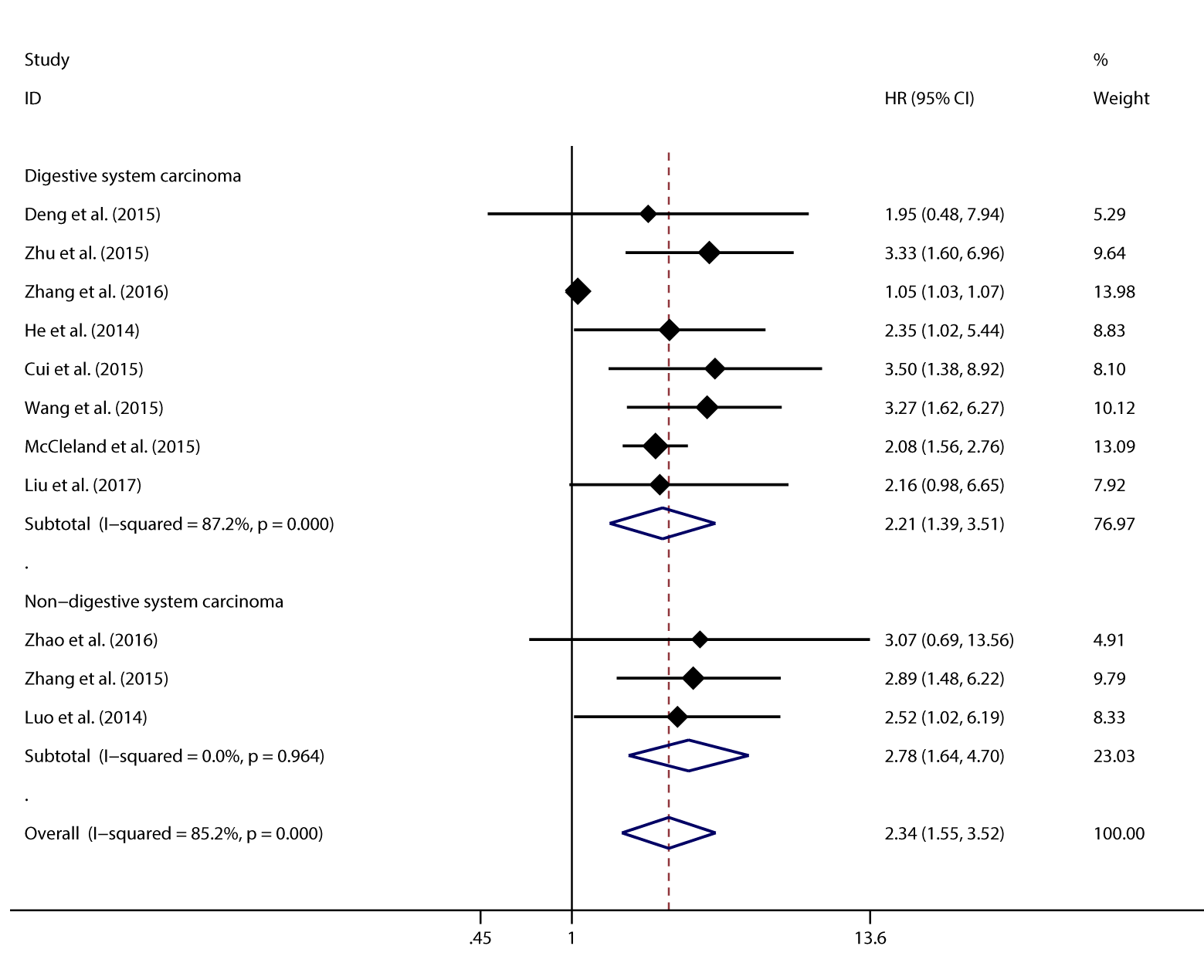

Supplement: S1 File — (ZIP) [file pone.0284940.s001.zip › 2 Figures/Fig 2B.tif]

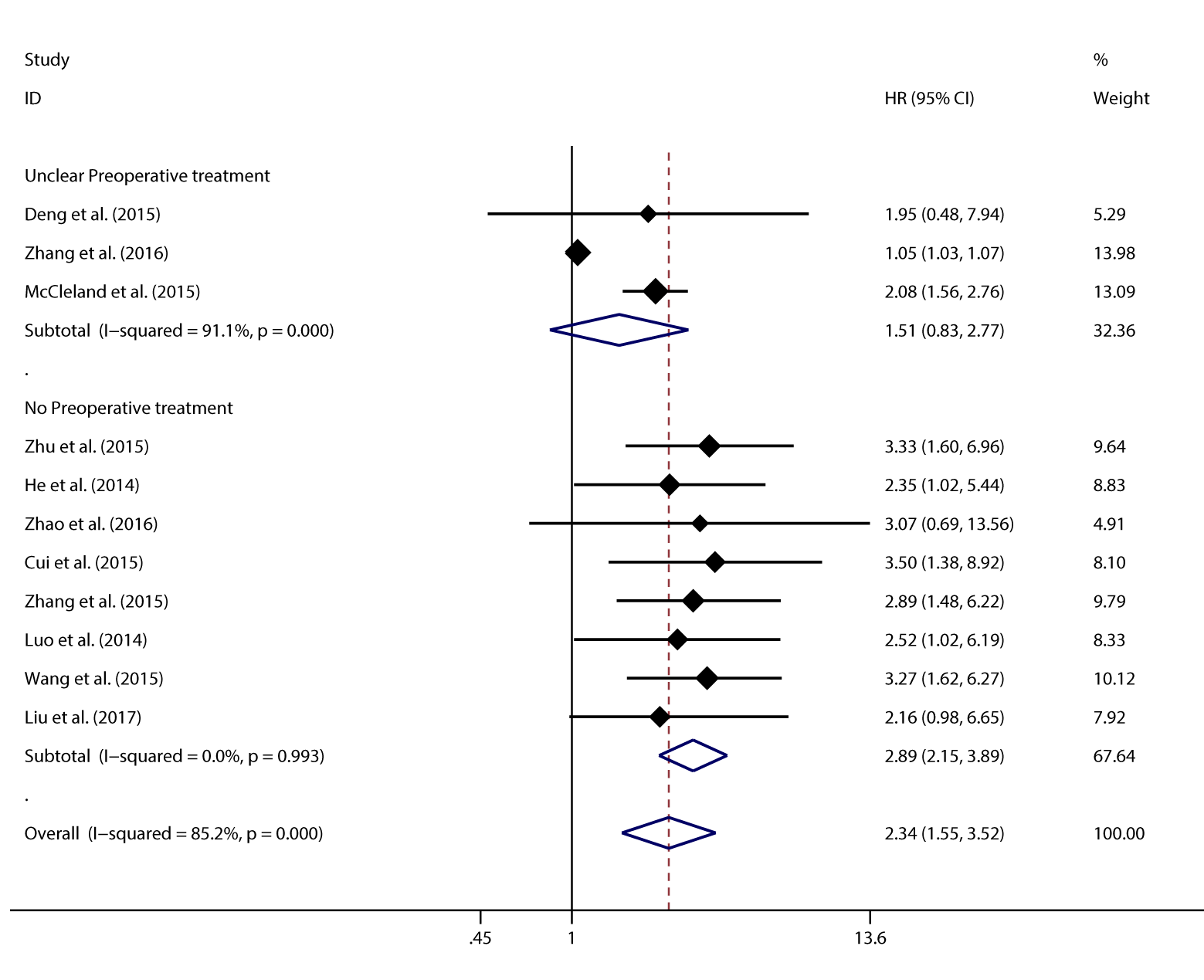

Supplement: S1 File — (ZIP) [file pone.0284940.s001.zip › 2 Figures/Fig 2C.tif]

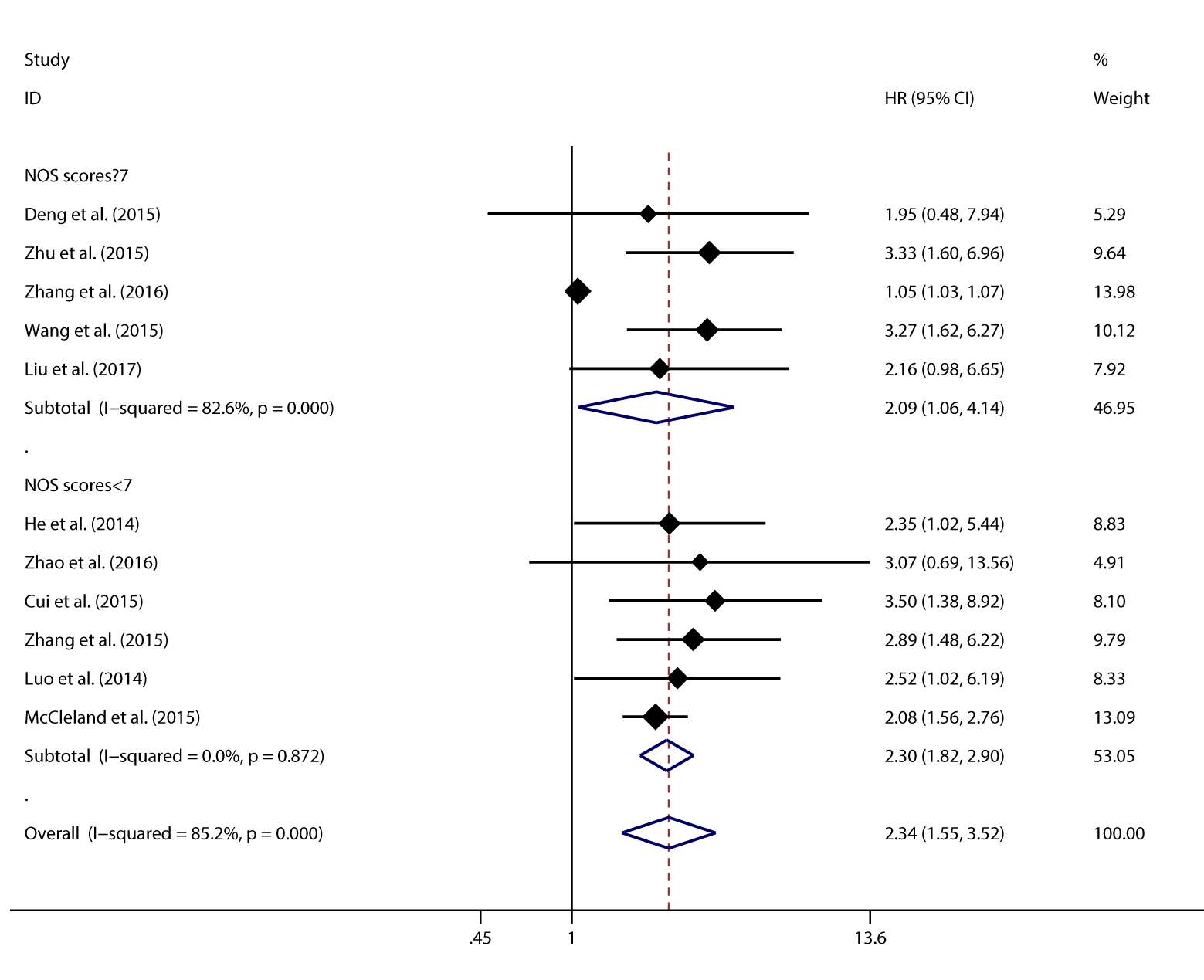

Supplement: S1 File — (ZIP) [file pone.0284940.s001.zip › 2 Figures/Fig 2D.tif]

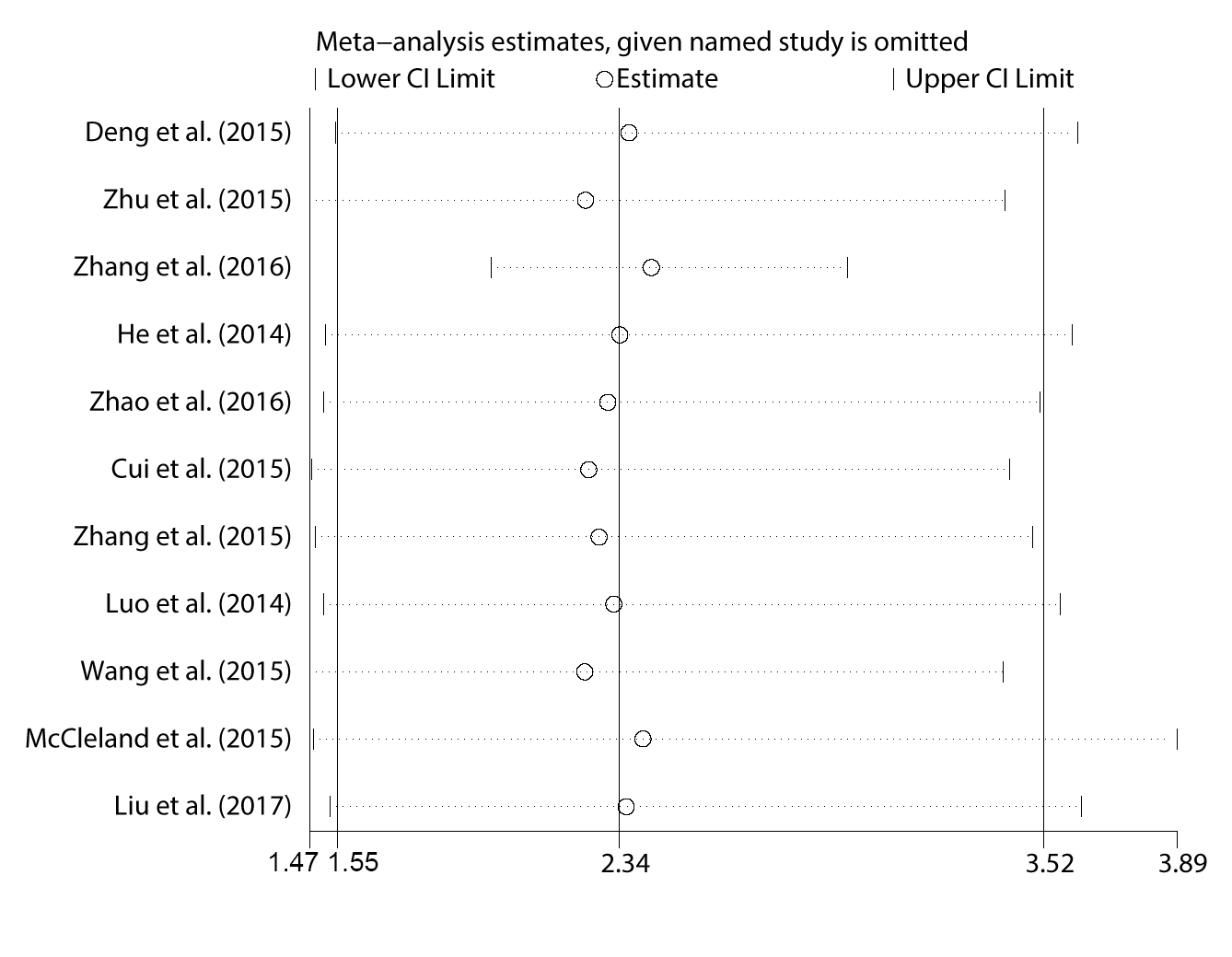

Supplement: S1 File — (ZIP) [file pone.0284940.s001.zip › 2 Figures/Fig 3.tif]

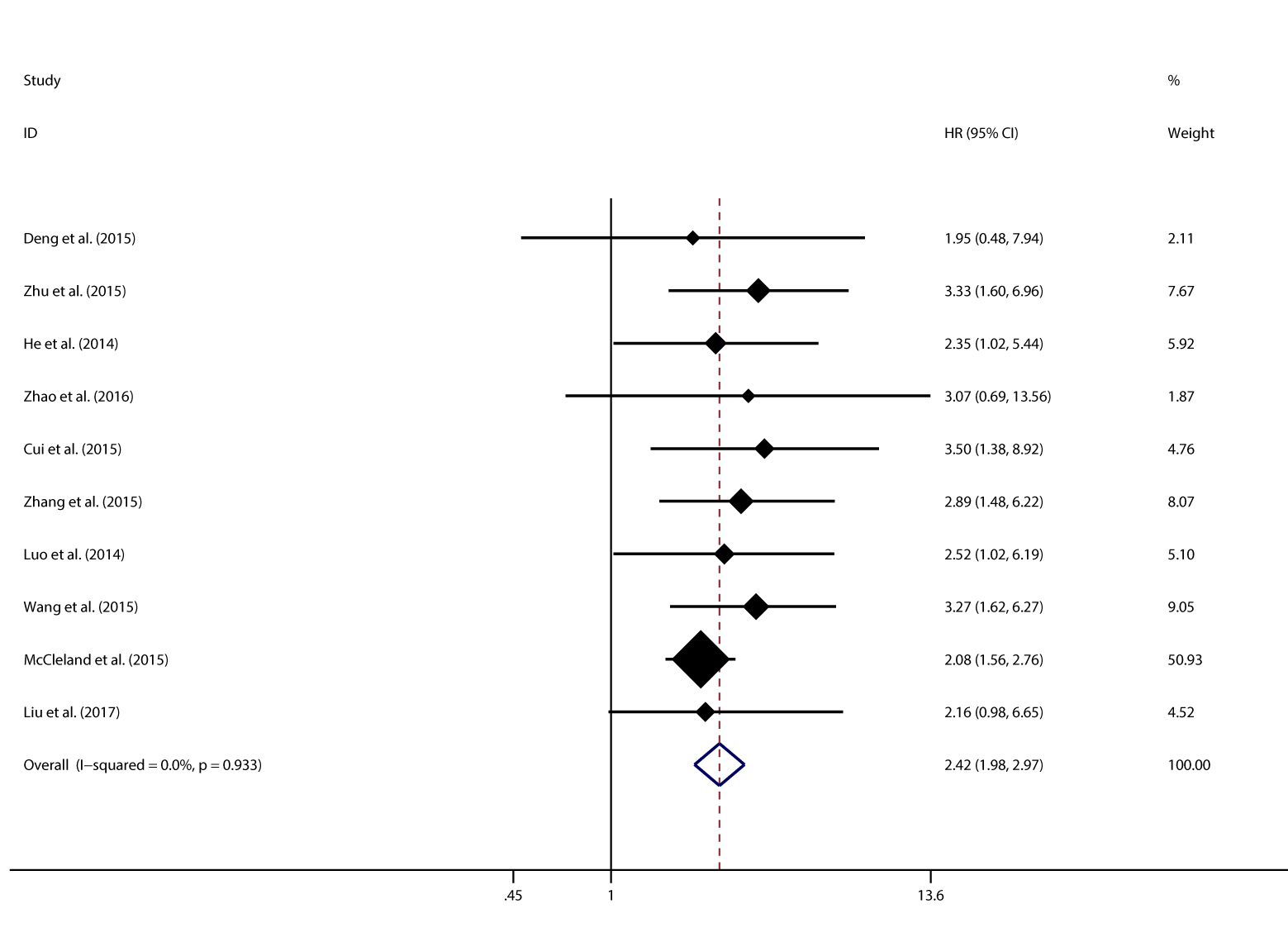

Supplement: S1 File — (ZIP) [file pone.0284940.s001.zip › 2 Figures/Fig 4.tif]

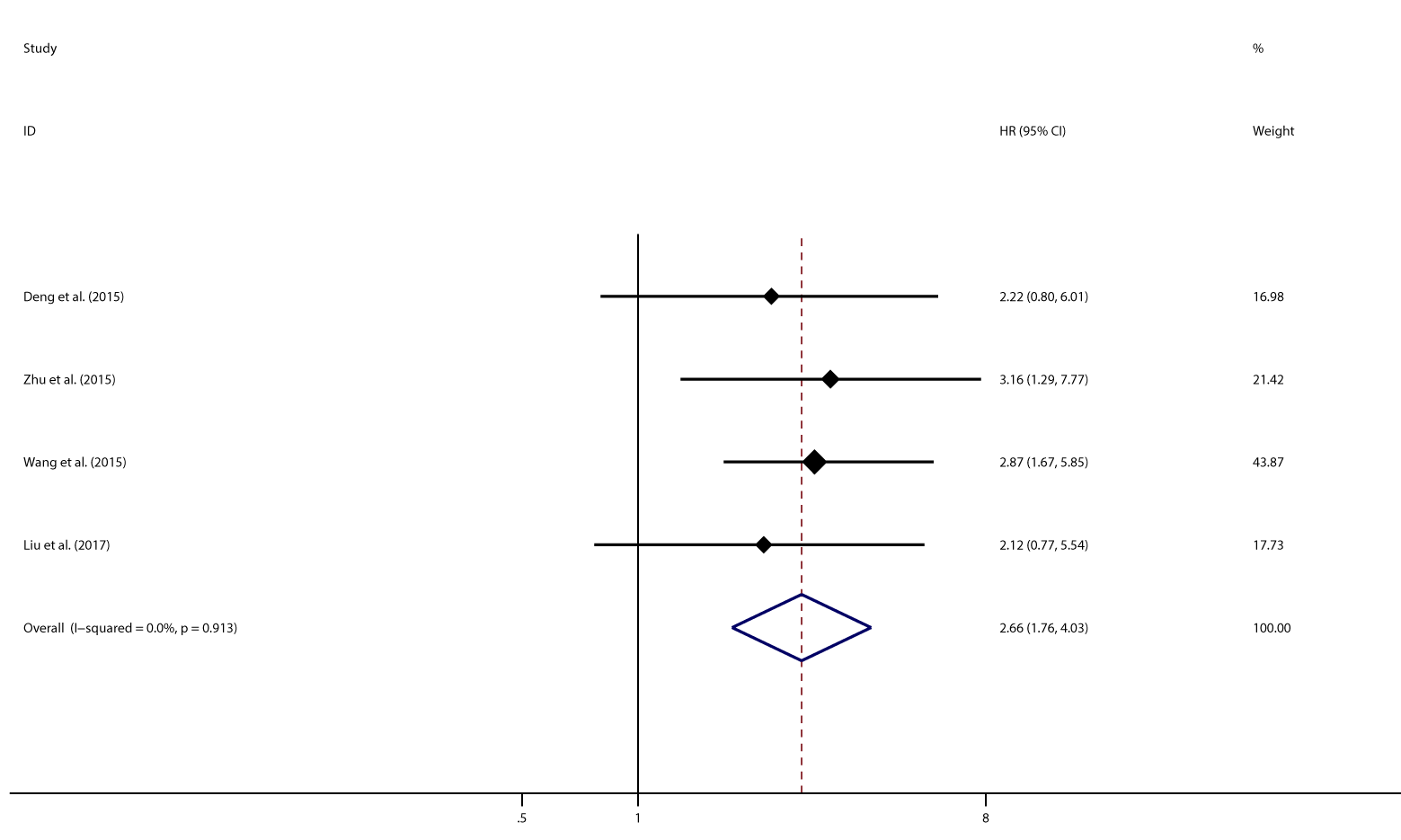

Supplement: S1 File — (ZIP) [file pone.0284940.s001.zip › 2 Figures/Fig 5.tif]

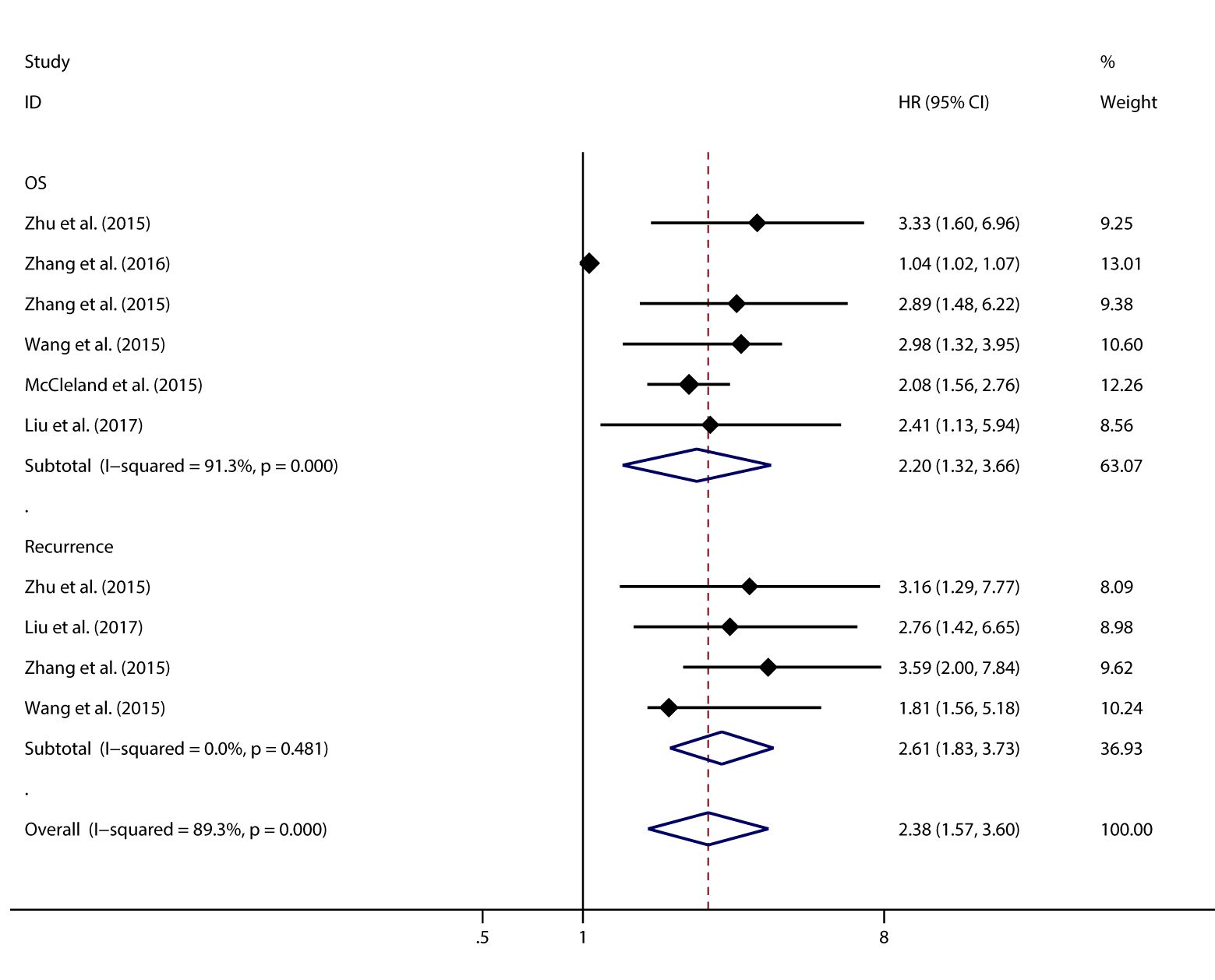

Supplement: S1 File — (ZIP) [file pone.0284940.s001.zip › 2 Figures/Fig 6.tif]

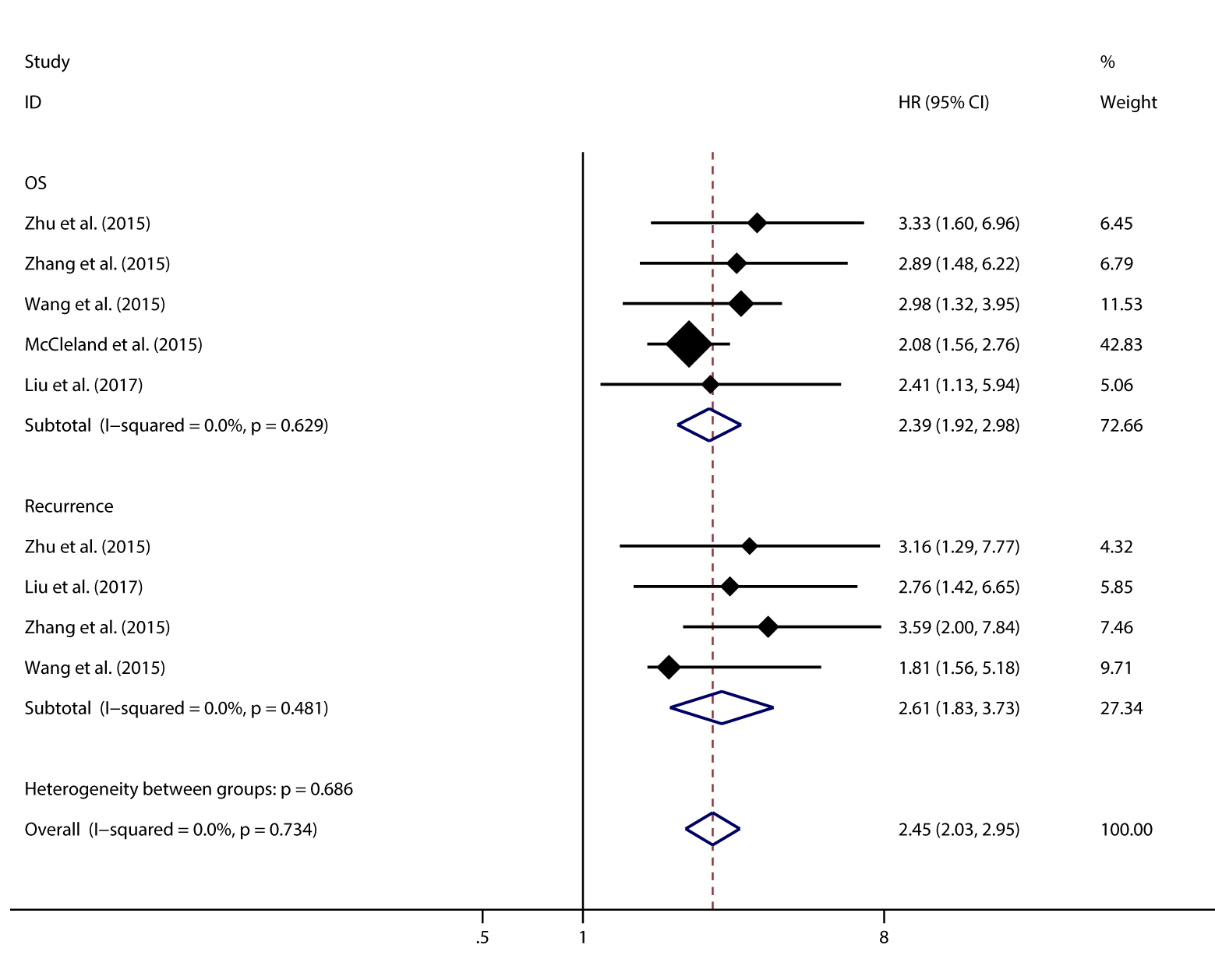

Supplement: S1 File — (ZIP) [file pone.0284940.s001.zip › 2 Figures/Fig 7.tif]

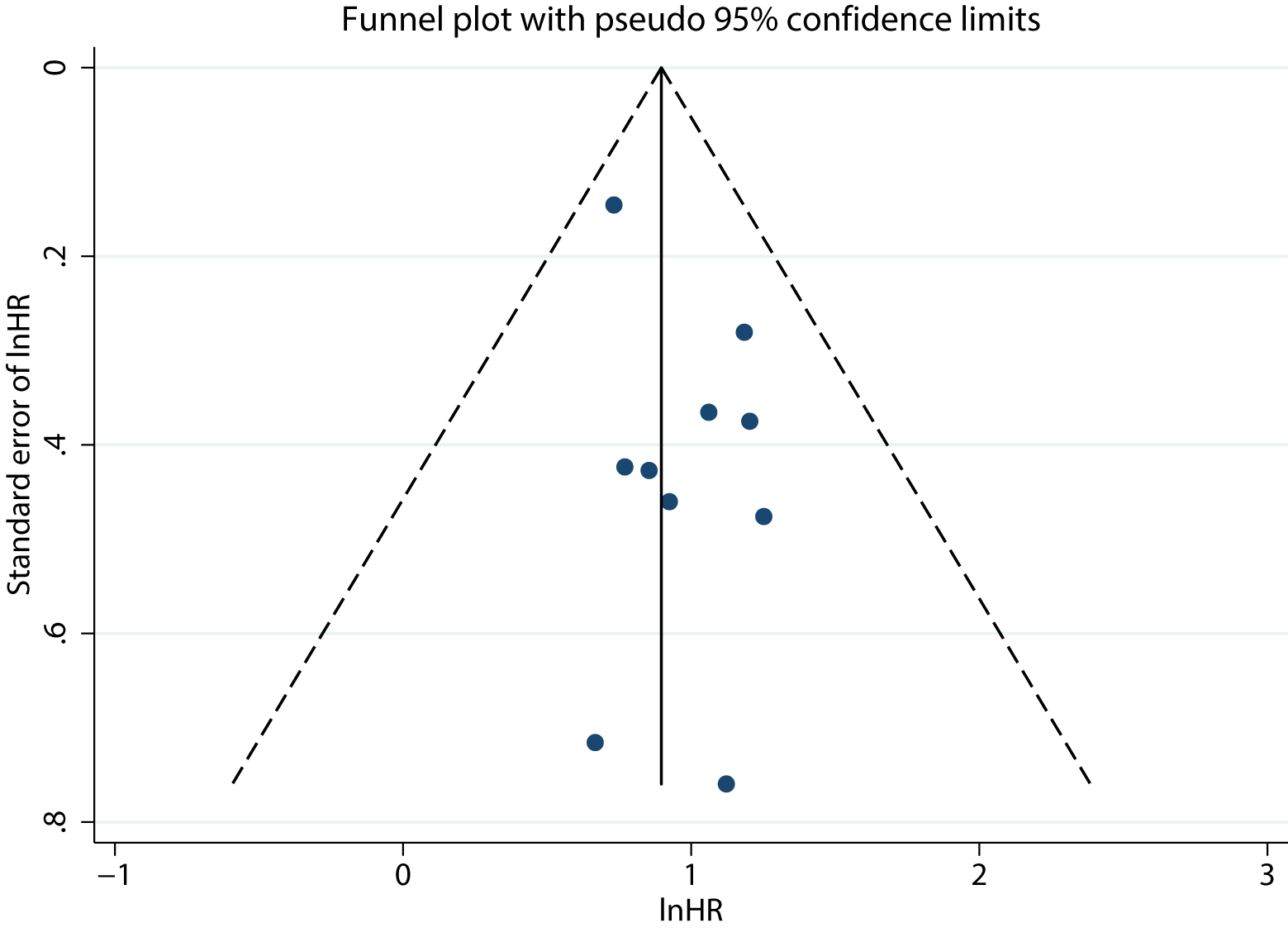

Supplement: S1 File — (ZIP) [file pone.0284940.s001.zip › 2 Figures/S1A_Fig.tif]

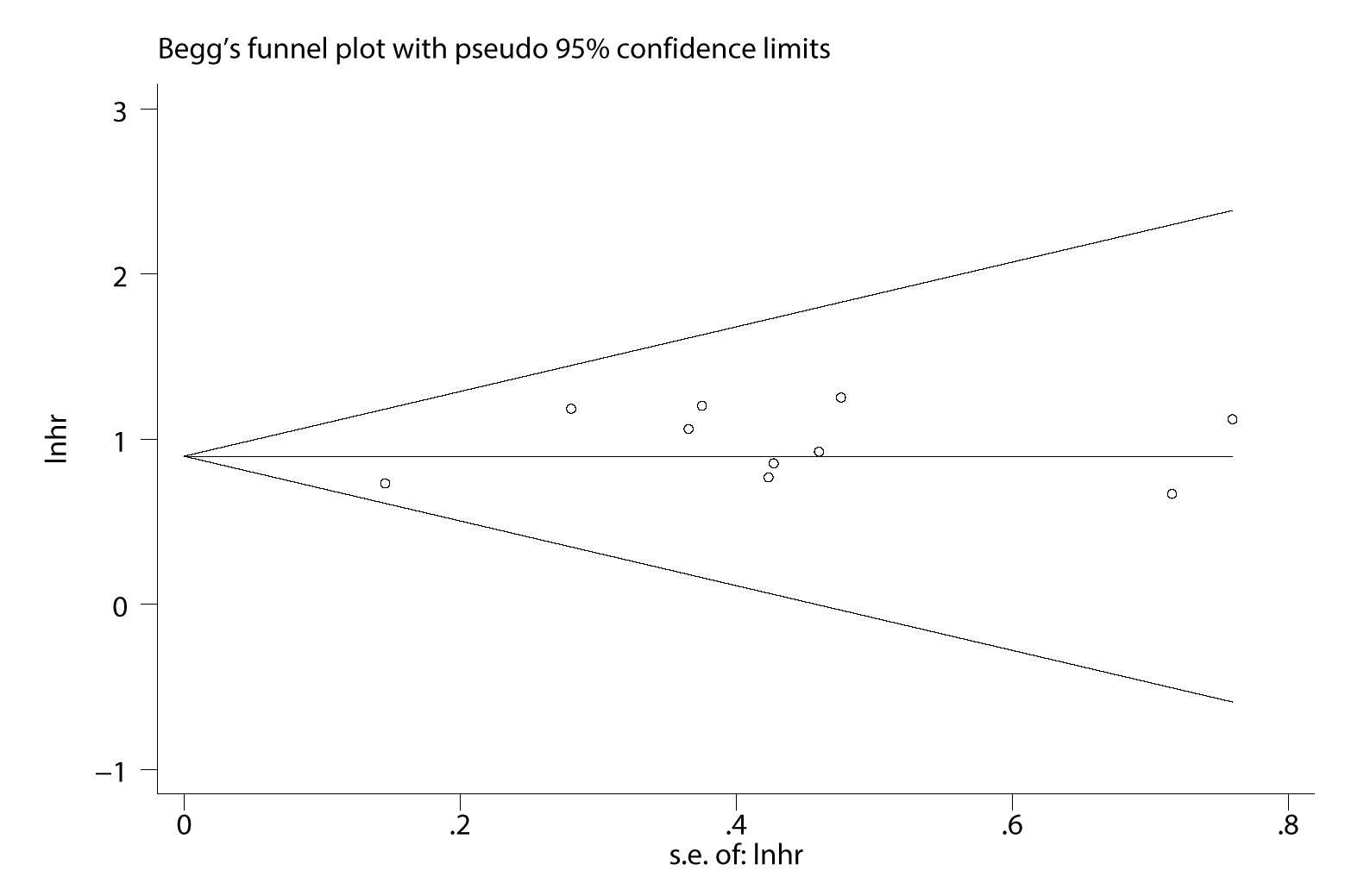

Supplement: S1 File — (ZIP) [file pone.0284940.s001.zip › 2 Figures/S1B_Fig.tif]

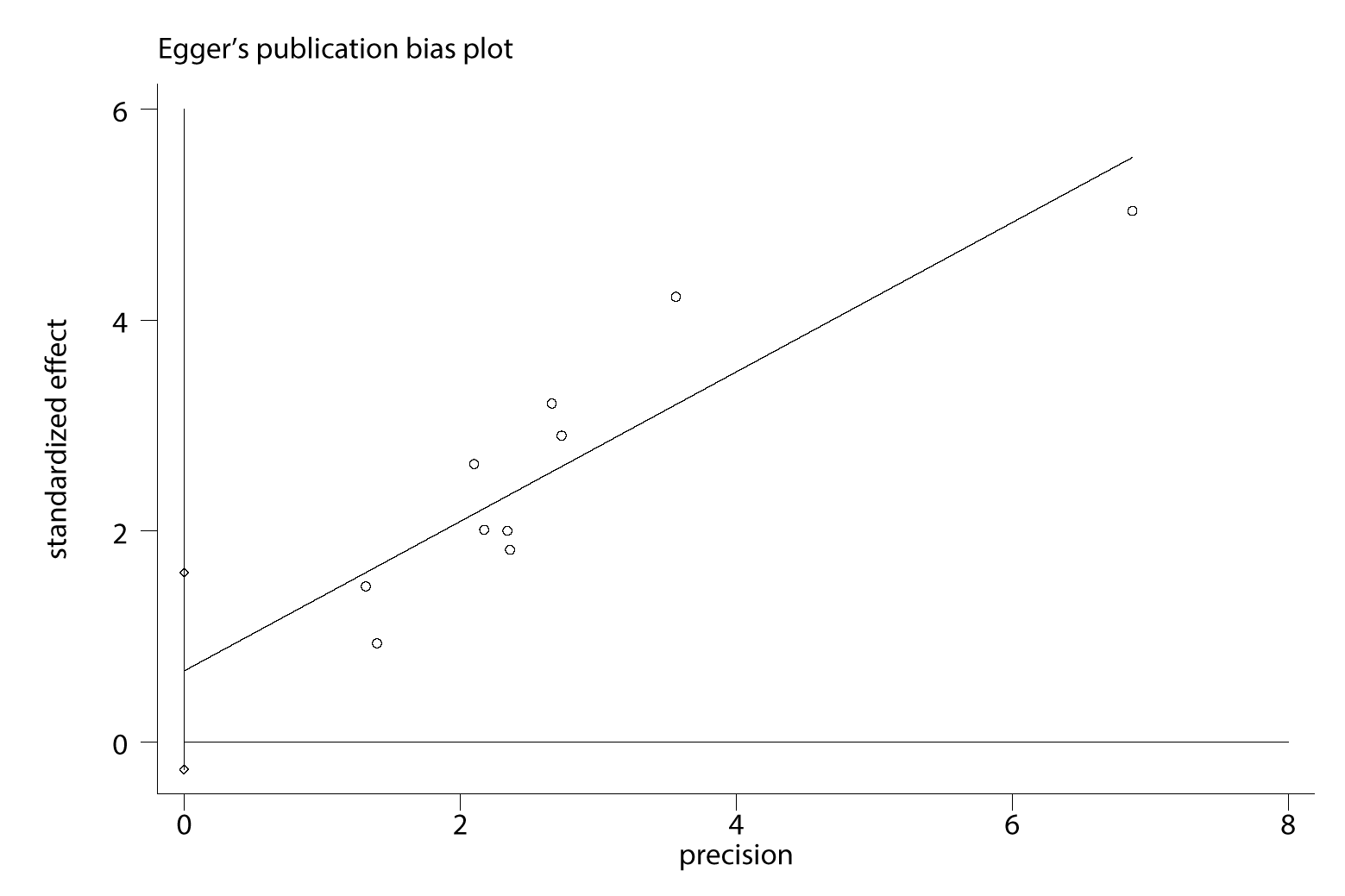

Supplement: S1 File — (ZIP) [file pone.0284940.s001.zip › 2 Figures/S1C_Fig.tif]

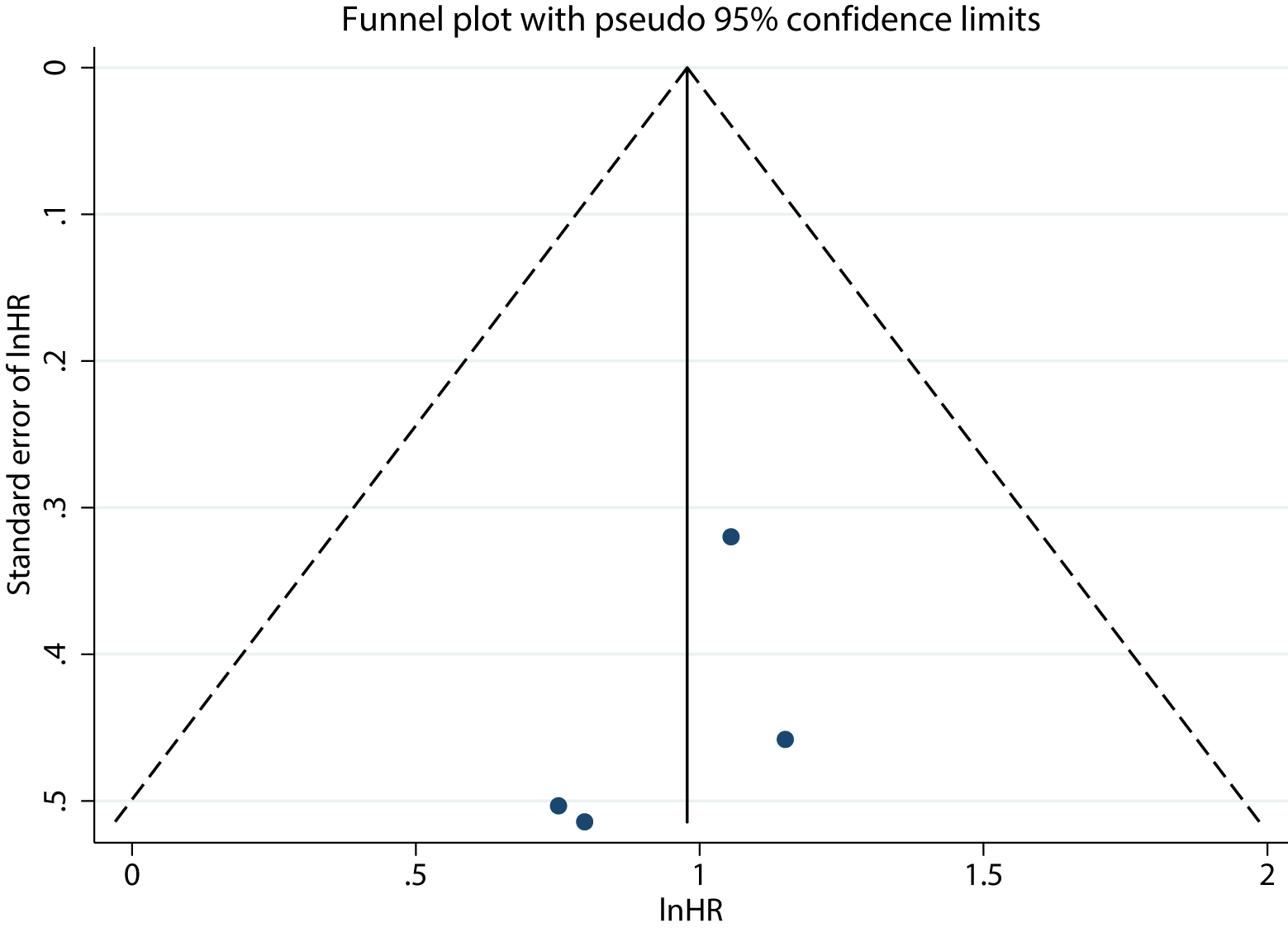

Supplement: S1 File — (ZIP) [file pone.0284940.s001.zip › 2 Figures/S2A_Fig.tif]

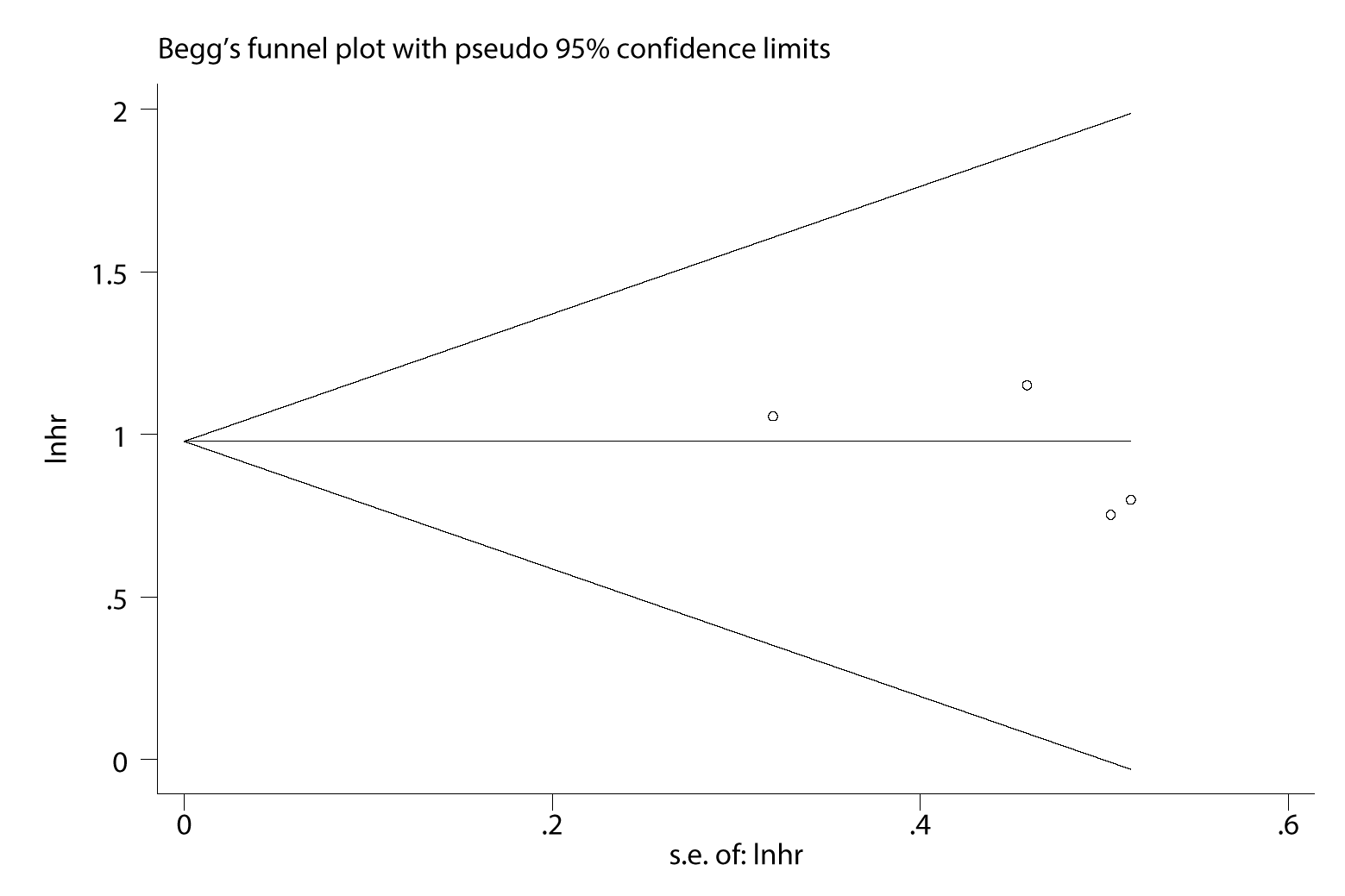

Supplement: S1 File — (ZIP) [file pone.0284940.s001.zip › 2 Figures/S2B_Fig.tif]

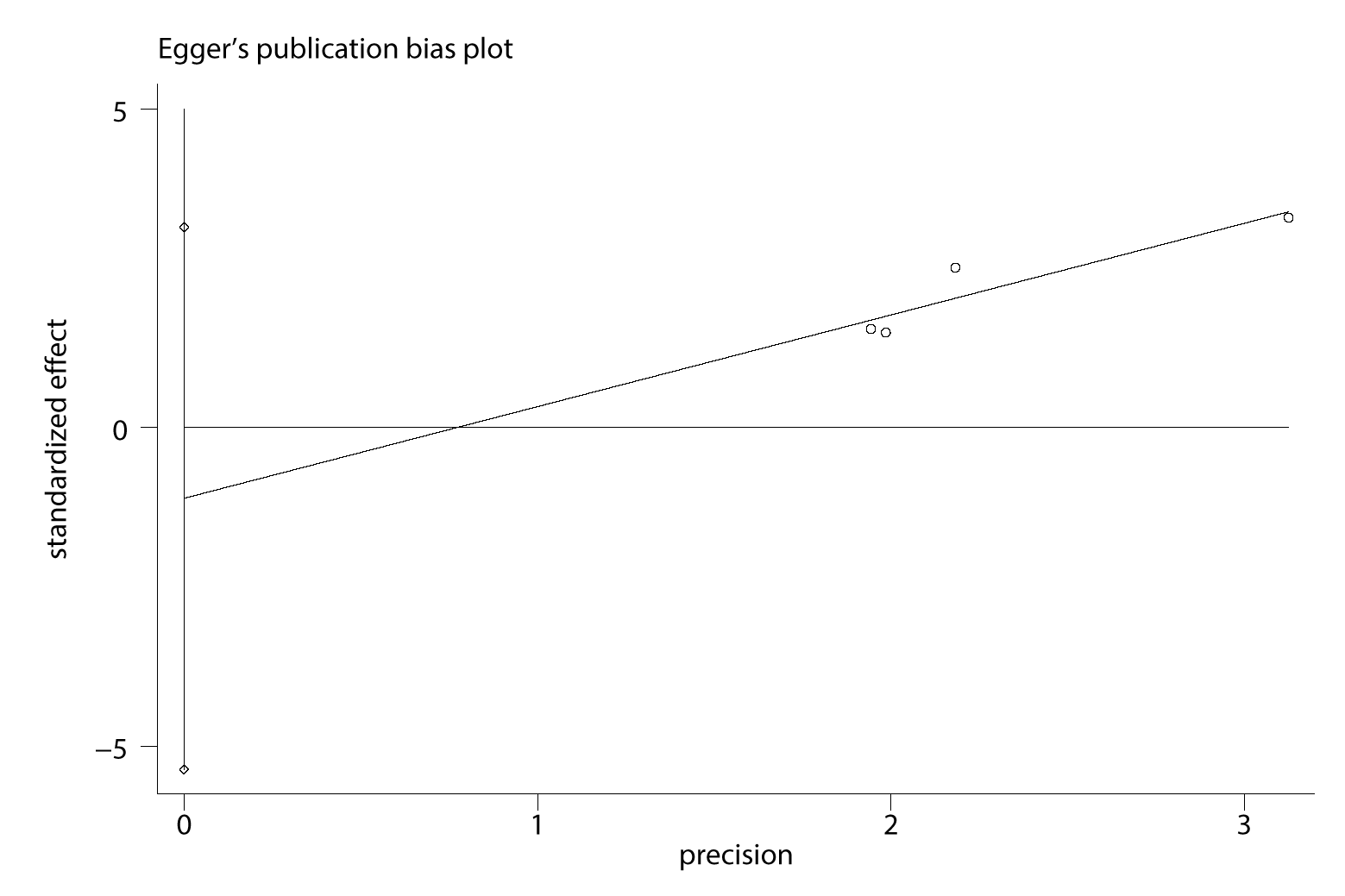

Supplement: S1 File — (ZIP) [file pone.0284940.s001.zip › 2 Figures/S2C_Fig.tif]

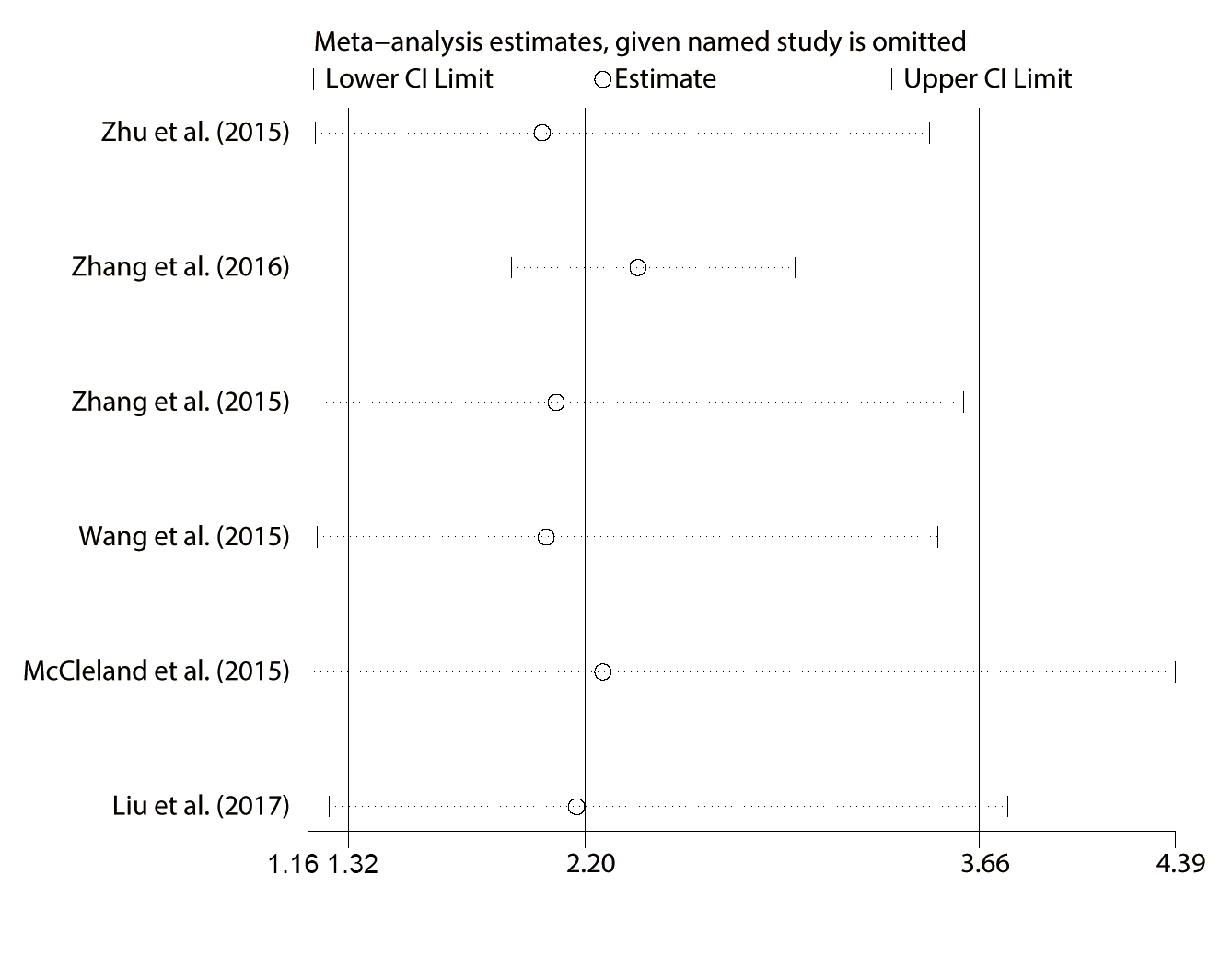

Supplement: S1 File — (ZIP) [file pone.0284940.s001.zip › 2 Figures/S3_Fig.tif]

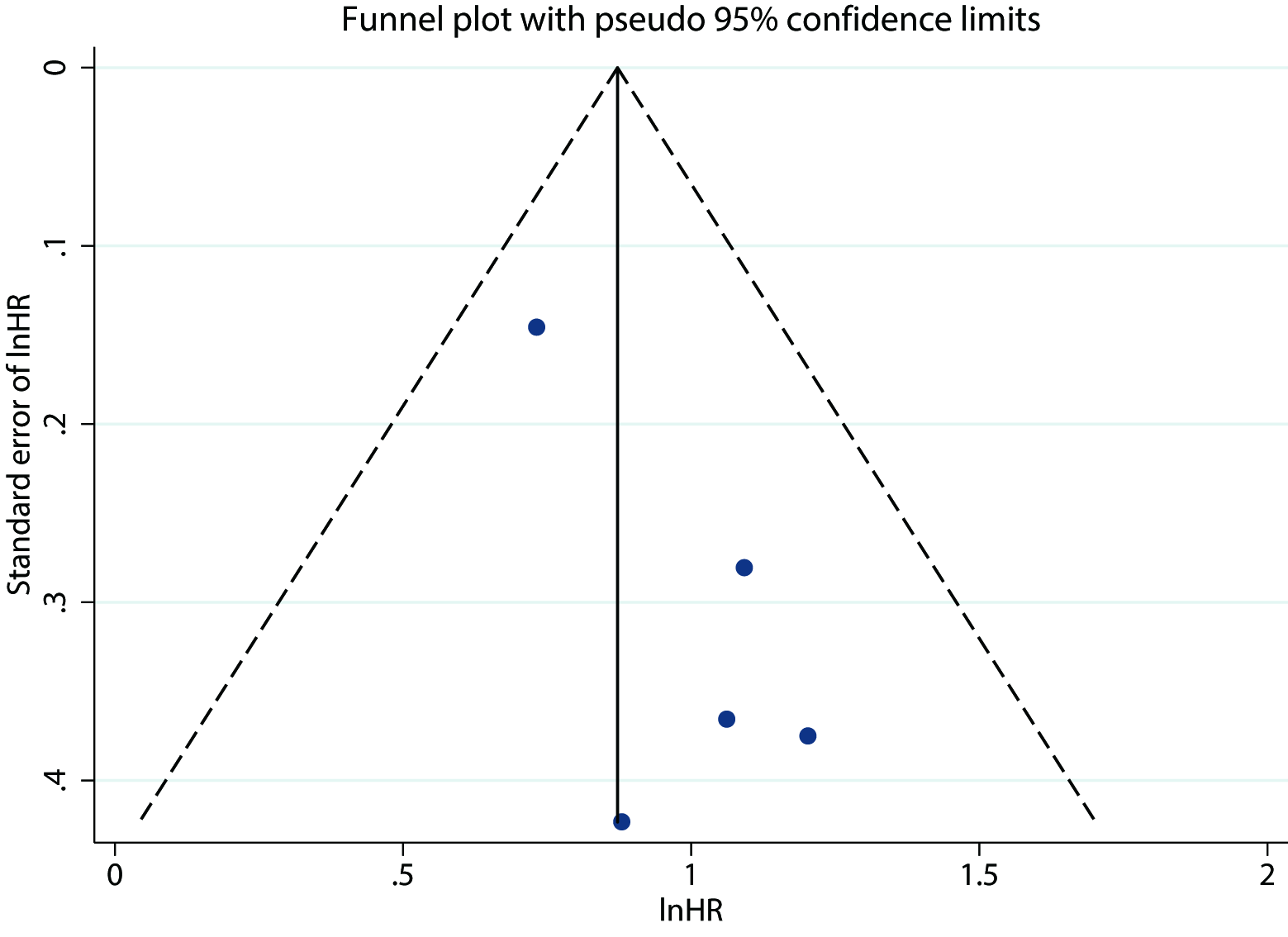

Supplement: S1 File — (ZIP) [file pone.0284940.s001.zip › 2 Figures/S4A_Fig.tif]

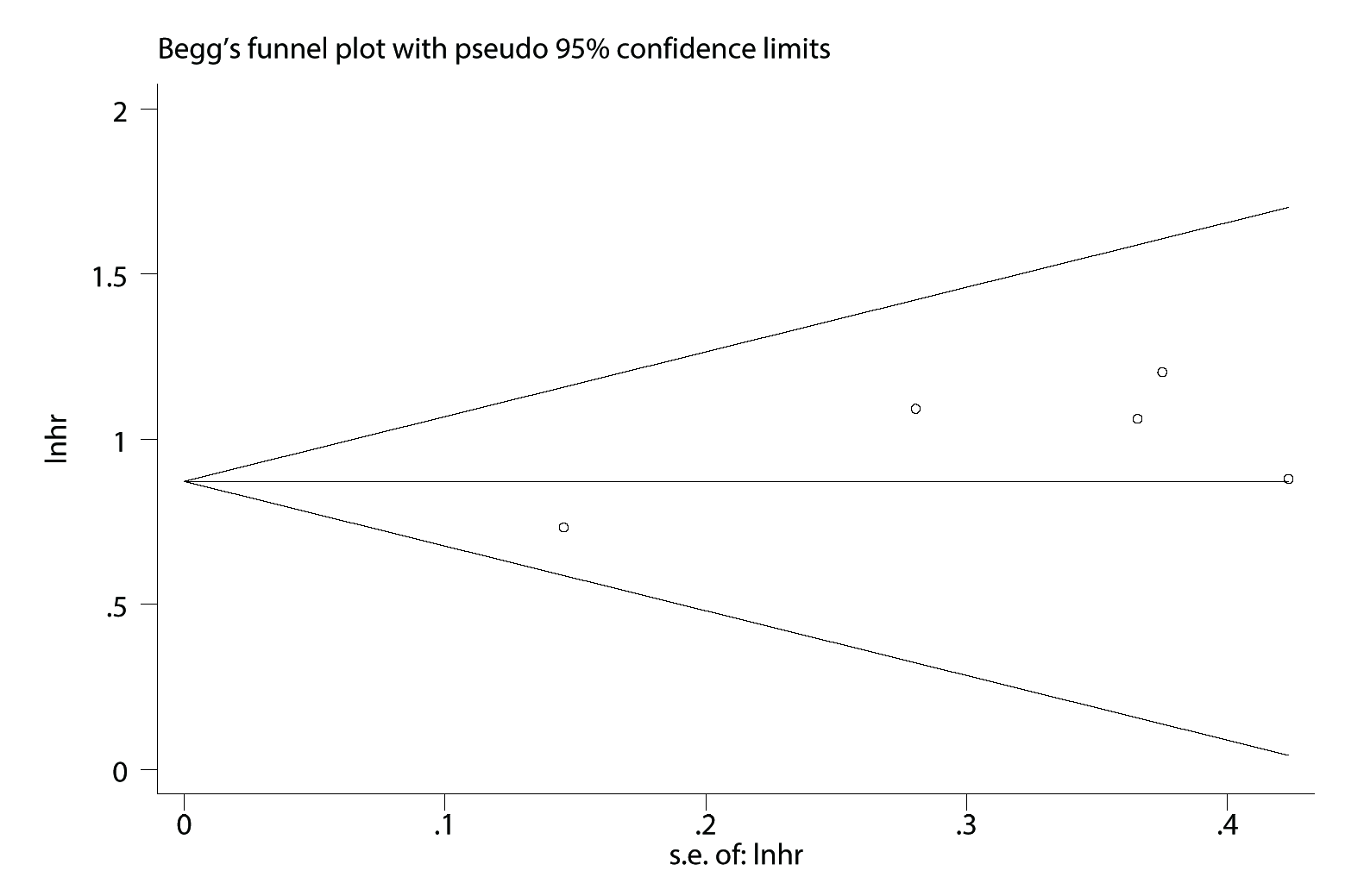

Supplement: S1 File — (ZIP) [file pone.0284940.s001.zip › 2 Figures/S4B_Fig.tif]

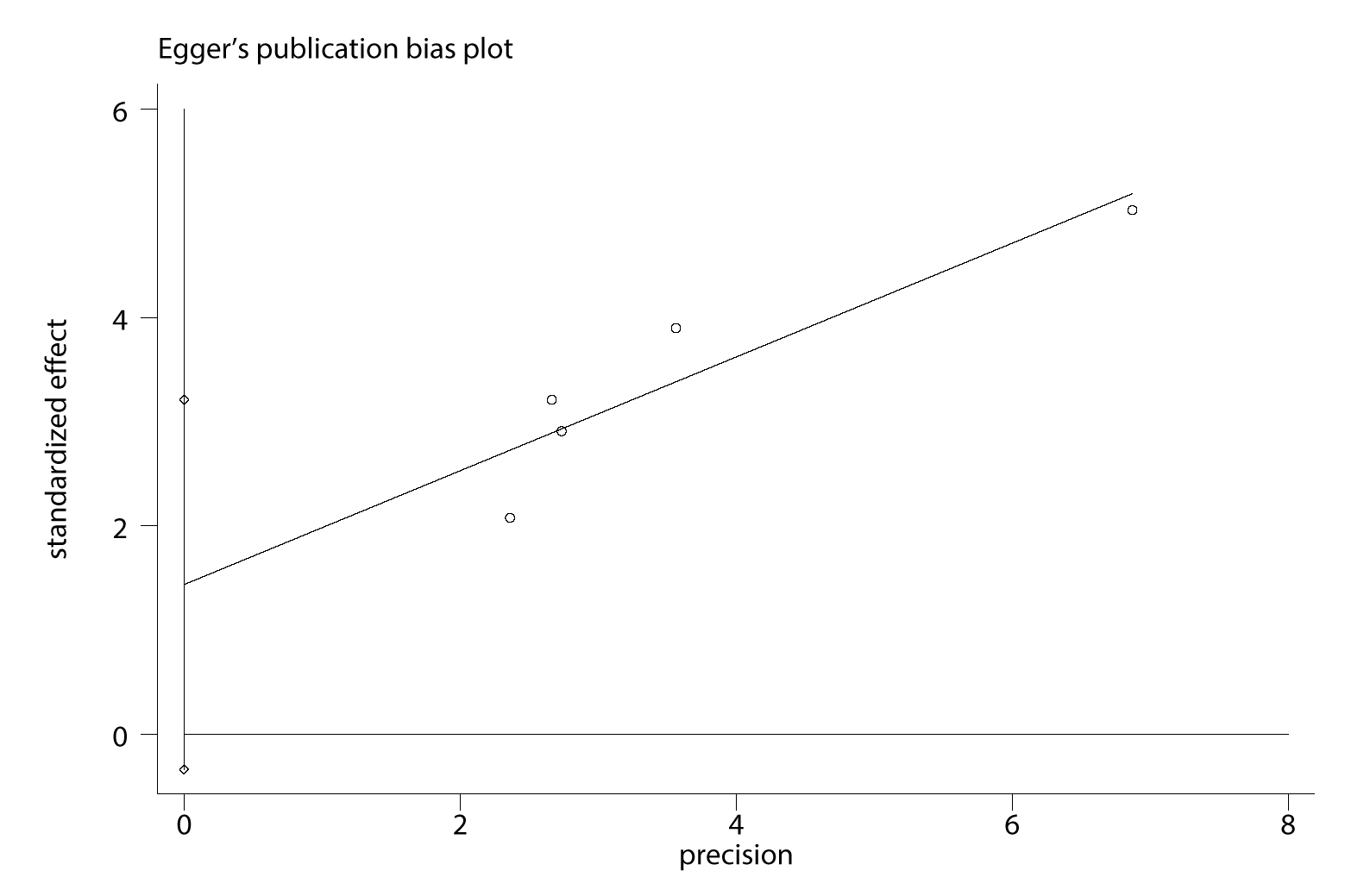

Supplement: S1 File — (ZIP) [file pone.0284940.s001.zip › 2 Figures/S4C_Fig.tif]
