# Supplementary material for: Expression of Concern: Prognostic value of long non-coding RNA CCAT1 expression in patients with cancer: A meta-analysis
Source: PLoS One. 2023 Apr 20;18(4):e0284940. doi: 10.1371/journal.pone.0284940 (PMC10118116; doi:10.1371/journal.pone.0284940)
Supplement: S1 File — (ZIP) [file pone.0284940.s001.zip › 4 Documents/04 A full electronic search strategy and procedure for Pubmed.doc]

**A full electronic search strategy and procedure for Pubmed:**

In this paper, two authors (DY Shi and FS Wu) independently used the following tools: Pubmed, Web of Science, OVID, and CNKI to obtain relevant articles on CCAT1 as a prognostic factor for the survival of patients with any cancer. Here we choose one of the database – Pubmed as example, which is one of the most widely used database among researchers to repeat our search procedure.

The literature search language was limited to English in Pubmed and the last search date was February 27, 2017. To increase the sensitivity of the search, both MeSH terms and free words were used. The search strategy was: “CCAT1 or colon cancer associated transcript 1 or CARLo-5” and “long non-coding RNA or lncRNA or non-coding RNA or RNA long non-coding” and “cancer or sarcoma or carcinoma or neoplasm or malignancy” and “prognosis or mortality of metastasis or progression or development or outcome or survival or recurrence or clinical significance”

In PubMed Advanced Search Builder: we chose “all fields” before entering above searching words, and limits we used included “language: English” and “date publication:1990/01/01-2017/02/27”. The detailed search process, search strategy and items found were as following table. The full search strategy of this paper for Pubmed was as #5.

| No. | Search strategy | Items found |
| --- | --- | --- |
| #1 | (((((CCAT1) OR colon cancer associated transcript 1) OR CARLo-5)) AND English[Language]) AND ("1990/01/01"[Date - Publication] : "2017/02/27"[Date - Publication]) | 173 |
| #2 | (((((long non-coding RNA) OR lncRNA) OR non-coding RNA) OR RNA long non-coding RNA) AND English[Language]) AND ("1990/01/01"[Date - Publication] : "2017/02/27"[Date - Publication]) | 151800 |
| #3 | ((((((cancer) OR sarcoma) OR carcinoma) OR neoplasm) OR malignancy) AND English[Language]) AND ("1990/01/01"[Date - Publication] : "2017/02/27"[Date - Publication]) | 2336844 |
| #4 | ((((((((((prognosis) OR mortality) OR metastasis) OR progression) OR development) OR outcome) OR survival) OR recurrence) OR clinical significance) AND English[Language]) AND ("1990/01/01"[Date - Publication] : "2017/02/27"[Date - Publication]) | 4529787 |
| #5 | #5 = #1 and #2 and #3 and #4  ((((((((((((((prognosis) OR mortality) OR metastasis) OR progression) OR development) OR outcome) OR survival) OR recurrence) OR clinical significance) AND English[Language]) AND ("1990/01/01"[Date - Publication] : "2017/02/27"[Date - Publication]))) AND (((((((cancer) OR sarcoma) OR carcinoma) OR neoplasm) OR malignancy) AND english[Language]) AND ("1990/01/01"[Date - Publication] : "2017/02/27"[Date - Publication]))) AND ((((((long non-coding RNA) OR lncRNA) OR non-coding RNA) OR RNA long non-coding RNA) AND english[Language]) AND ("1990/01/01"[Date - Publication] : "2017/02/27"[Date - Publication]))) AND ((((((CCAT1) OR colon cancer associated transcript 1) OR CARLo-5)) AND English[Language]) AND ("1990/01/01"[Date - Publication] : "2017/02/27"[Date - Publication])) | 42 |
