# Supplementary material for: Expression of Concern: Prognostic value of long non-coding RNA CCAT1 expression in patients with cancer: A meta-analysis
Source: PLoS One. 2023 Apr 20;18(4):e0284940. doi: 10.1371/journal.pone.0284940 (PMC10118116; doi:10.1371/journal.pone.0284940)
Supplement: S1 File — (ZIP) [file pone.0284940.s001.zip › 4 Documents/05 Detailed table of quality assessment for included studies.doc]

| Study authors | Selection | | | | Comparability | | Outcome | | | NOS scores |
| --- | --- | --- | --- | --- | --- | --- | --- | --- | --- | --- |
|  | Item 1 | Item 2 | Item 3 | Item 4 | Item 1 | Item 2 | Item 1 | Item 2 | Item 3 |  |
| Deng et al. | 1 | 1 | 1 | 1 | 1 | 0 | 1 | 1 | 0 | 7 |
| Zhu et al. | 1 | 1 | 1 | 1 | 1 | 0 | 1 | 1 | 0 | 7 |
| Zhang et al. | 1 | 1 | 1 | 1 | 0 | 0 | 1 | 1 | 1 | 7 |
| He et al. | 0 | 1 | 1 | 1 | 1 | 0 | 1 | 1 | 0 | 6 |
| Zhao et al. | 1 | 1 | 1 | 1 | 0 | 0 | 1 | 1 | 0 | 6 |
| Cui et al. | 1 | 1 | 1 | 1 | 0 | 0 | 1 | 1 | 0 | 6 |
| Zhang et al. | 0 | 1 | 1 | 1 | 1 | 0 | 1 | 1 | 0 | 6 |
| Luo et al. | 0 | 1 | 1 | 1 | 1 | 0 | 1 | 1 | 0 | 6 |
| Wang et al. | 1 | 1 | 1 | 1 | 1 | 0 | 1 | 1 | 0 | 7 |
| McCleland et al. | 1 | 1 | 1 | 1 | 0 | 0 | 1 | 1 | 0 | 6 |
| Liu et al. | 1 | 1 | 1 | 1 | 1 | 0 | 1 | 1 | 0 | 7 |
